# Up-Regulation of hsa_circ_0000517 Predicts Adverse Prognosis of Hepatocellular Carcinoma

**DOI:** 10.3389/fonc.2019.01105

**Published:** 2019-10-22

**Authors:** Xicheng Wang, Xining Wang, Wenxin Li, Qi Zhang, Jie Chen, Tao Chen

**Affiliations:** ^1^Department of Hepatobiliary Surgery, Sun Yat-sen Memorial Hospital, Sun Yat-sen University, Guangzhou, China; ^2^Guangdong Provincial Key Laboratory of Malignant Tumor Epigenetics and Gene Regulation, Medical Research Center, Sun Yat-sen Memorial Hospital, Sun Yat-sen University, Guangzhou, China; ^3^Department of Internal Infection, Nanfang Hospital, Southern Medical University, Guangzhou, China

**Keywords:** hsa_circ_0000517, hepatocellular carcinoma, circRNAs, bioinformatics, GEO

## Abstract

Although huge progress has been made in therapeutics against hepatocellular carcinoma (HCC) over the decades, the prognosis of this lethal disease remains poor. To find out risk factors for HCC-related outcome and better predict the prognosis, there is an unmet need to identify novel biomarkers of HCC. Accumulating evidence suggests that circRNAs play pivotal roles in carcinogenesis of several malignancies. In this study, we analyzed two datasets (GSE 94508 and GSE 97332) to examine differentially expressed circRNAs markedly related to HCC pathogenesis. Using Limma package in R and WGCNA analysis, hsa_circ_0000517 was significantly up-regulated in HCC (adjusted *P* < 0.01). Thereafter, a hsa_circ_0000517-related regulatory network was built based on application of databases including CSCD, TargetScan, miRDB, and miRTarBase. We uncovered the potential function of hsa_circ_0000517 through bioinformatics approaches, such as PPI network, GO, and KEGG pathway analyses. Specifically, functional analysis unveiled that hsa_circ_0000517 was likely to regulate the MAPK and Ras pathway through sponging several miRNAs and having an impact on the expression of TP53, MYC, and AKT1. To verify our initial finding, the expression of hsa_circ_0000517 in 60 HCC patients was detected by qRT-PCR and the expression in cancer tissues was higher compared with the paracarcinoma tissues. Survival analysis suggests high hsa_circ_0000517 expression was associated with adverse prognosis in HCC patients. Furthermore, this circRNA was significantly up-regulated in worse TNM stage, consistent with the progressive-stage-specific characteristic of circRNAs. A prognostic nomogram built on AFP and has_circ_0000517 showed significant diagnostic value. In all, we concluded that hsa_circ_0000517, a promising molecular in underlying mechanism of HCC, is a potent valuable biomarker for prognosis prediction.

## Introduction

Hepatocellular carcinoma (HCC) is one of the most fatal carcinomas with relatively high mortality (1). Although there has been exciting development in curative remedies such as liver transplantation and surgical resection (2), the overall prognosis of HCC remains unsatisfactory, as numerous HCC patients are diagnosed with an advanced stage (3). Therefore, there is an unmet need to develop effective biomarkers for diagnosis and prognosis of HCC.

In recent studies, circular RNAs (circRNAs), characterized by stable circular structure and sponge function in most cells, have been identified as one kind of novel and important regulatory non-coding RNAs (ncRNA). Given that circRNA expression shows high heterogeneity among different cells, tissues, and even the stages of disease, this kind of ncRNA has been considered as a latent diagnostic and prognostic biomarker and promising target for developing new therapeutic options against human malignant cancers (4). Prior studies have demonstrated that circRNAs are correlated with cancers, including gastric cancer, colorectal cancer, ovarian cancer, and HCC (5–7). And circRNAs have been reported to function as miRNA sponges with the competence of regulating miRNA via miRNA-targeted mRNAs repression since they were first studied (8). A single circRNA may contain multiple binding sites for various miRNAs, raising the possibility of involving in the regulation of several miRNA-targeted genes. However, the underlying function of most circRNAs and their mechanism in diseases are not completely known (9, 10).

In the current study, we analyzed GSE 97332 and GSE 94508 from the Gene Expression Omnibus (GEO) database to mine differentially expressed circRNA expression data. We selected differentially expressed circRNAs in HCC tissues compared with adjacent normal specimens. Then, we concentrated on the comprehensive analysis of hsa_circ_0000517, the overlapping and up-regulated circRNA between two datasets. Furthermore, in an attempt to better figure out the relationship between hsa_circ_0000517 and prognosis of HCC, quantitative real time-polymerase chain reaction (qRT-PCR) was performed in 60 HCC patients from our center and corresponding clinicopathological characteristics were analyzed by various statistical analyses, involving cox regression analyses and a Least Absolute Shrinkage and Selector Operation (Lasso) regression algorithm.

## Materials and Methods

### GEO Data

GEO (http://www.ncbi.nlm.nih.gov/geo/) is a public database providing functional genomic information from high-throughout gene expression, chips, and microarray data (11). A micro-array search of circular RNA in HCC was conducted in the GEO database with the following keywords: (“circRNA” and “liver cancer”), we could simply find out two datasets: GSE 94508 and GSE 97332. We had no restrictions on language of related studies. Two HCC-related datasets were analyzed in the present study, namely GSE 97332 (12) (data from a total of seven paired malignancies and paracancerous tissues) and GSE 94508 (13) (data from five primary liver tumors and five matched normal tissues). Limma package (version: 3.40.2) of R software was used to study the differential expression of circRNAs (14). The adjusted *P*-value was analyzed to correct for false positive results in GSE 94508 and GSE 97332. “Adjusted *P* < 0.05 and Log (Fold Change) >1 or Log (Fold Change)< −1” were defined as the thresholds for the screening of differential expression of circRNAs. Probe sets without corresponding circbase ID (http://www.circbase.org) were discarded. The circos plot was used to present the chromosomes, hosting genes, average circRNA expression, and the circRNA name (15).

### Venn Plot

Cancer-specific circRNA database (CSCD, http://gb.whu.edu.cn/CSCD) is a database of available RNA sequencing datasets from 87 cancer cell line samples, and 272,152 cancer-specific circRNAs are deposited in the CSCD database, including the corresponding number and position of MRE (microRNA response element), RBP (RNA binding protein), and ORF (open reading frame) elements located in cancer-specific circRNAs (16). In view of the data from CSCD, we identified one overlapping circRNA, hsa_circ_0000517, as a sponge of 73 miRNAs (Table S1). Three online tools, TargetScan (http://www.targetscan.org/vert_72/), miRDB (http://www.mirdb.org/), miRTarBase (http://mirtarbase.mbc.nctu.edu.tw/php/index.php), were then used to predict the potential targets of the miRNAs. With an aim to acquire more trustworthy targets, the Venn plot was performed to collect the consensus genes from the three online databases.

### WGCNA Analysis

We integrated and batched the data from both GSE 97332 and GSE 94508 to perform Weighted Gene Co-expression Network Analysis (WGCNA). The R package “WGCNA” was applied to explore traits-related modules (17). The data matrix, including more than 15 samples, was transformed into topological overlap matrix (TOM). According to TOM-based dissimilarity measure, all circRNAs were divided into different circRNA modules. In present work, we set soft-thresholding power as 18, scale free R2 as >0.80 and minimal module size as 30 to figure out key modules. The modules were then applied to analyze their correlation with HCC using Pearson's correlation test and adjusted *P* < 0.05 was considered significant.

### Construction and Analysis of PPI Networks

A total of 742 potential targets of the miRNAs were imported to the public database STRING (A Search Tool for Known and Predicted Protein-Protein Interactions, version 11.0, https://string-db.org) and a protein network was created. The network edges indicated confidence: the thicker the lines, the more reliabilities they are. Network edges stand for confidence and the custom value of minimum required interaction score was 0.910. Next, a TP53-related network was extracted from the whole PPI network using Cytoscape 3.7.1 (18). Finally, a bar plot was used to elucidate the specific degree of the PPI network.

### Functional Analysis

To further confirm the underlying function of potential targets, the data were analyzed by functional enrichment. Gene Ontology (GO) is a widely-used tool for annotating genes with functions, especially molecular function (MF), biological pathways (BP), and cellular components (CC) (19). Kyoto Encyclopedia of Genes and Genomes (KEGG) Enrichment Analysis is a practical resource for analytical study of gene functions and associated high-level genome functional information (20). To better understand the carcinogenesis of has_circ_0000517, ClusterProfiler package (version: 3.12.0) in R was employed to analyze the GO function of potential targets and enrich the KEGG pathway (21). “*P* < 0.05” was set as the inclusion criterion.

### Clinical Specimen and Cell Line Acquisition

Specimens of tumor and adjacent normal tissues were obtained from 60 HCC patients undergoing surgery at Department of Hepatobiliary Surgery, Sun Yat-sen Memorial Hospital of Sun Yat-Sen University (Guangzhou, China). The patients did not undergo any radiotherapy or chemotherapy before surgical operation. The normal and tumor tissues of 60 HCC patients were frozen immediately in liquid nitrogen and then stored at −80°C for RNA extraction. Detailed HCC patient information is listed in Table S1. The Ethics Committee of Sun Yat-sen Memorial Hospital approved this study. All patients provided written informed consent for all treatments performed and to have their data utilized for research purposes. The human HCC cell line SNU-387 were purchased from the Cell Bank of the Chinese Academy of Sciences (Shanghai, China). The SNU-387 cell line was cultured in Dulbecco's Modified Eagle Medium (Gibco BRL, Grand Island, NY, USA), supplemented with 10% fetal bovine serum (Gibco BRL) and incubated with 5% CO_2_ at 37°C.

### qRT-PCR and Sanger Sequence

Total RNA from tissues was extracted with Trizol reagent based on the manufacturer's instructions (Takara, Shiga, Japan). The mRNA was reverse transcribed using a Prime-Script RT reagent Kit (Takara); cDNA or circRNA were amplified and quantified on CFX96 system (Bio-Rad, Hercules, CA, USA). Briefly, qRT-PCR was performed in a final volume of 10 μL, and the thermal conditions were 95°C for 30 s, and 45 cycles of 95°C for 5 s and 60°C for 20 s. U6 and GAPDH was used as endogenous controls. Expression of hsa_circ_0000517 was calculated using the 2^−ΔΔCT^ method. The primers were as follows: U6, forward CTCGCTTCGGCAGCACA, reverse AACGCTTCACGAATTTGCGT; GAPDH, forward ACAACTTTGGTATCGTGGAAGG, reverse GCCATCACGCCACAGTTTC; hsa_circ_0000517, forward GGGAGGTGAGTTCCCAGAGA, reverse TGGCCCTAGTCTCAGACCTC.

### Statistical Analysis

Data are presented as means ± standard deviation with three independent replications. Statistical analysis was performed using GraphPad Prism (version: 8.0.0) and R software (http://www.R-project.org). In our present study, *P* < 0.05 was considered statistically significant. The Uni-variate cox regression analysis, Lasso regression analysis (22), Multi-variate cox regression analysis and Receiver Operating Characteristic curve (ROC) analysis were conducted in R to evaluate the prognostic potential of the risk factors in HCC patients in our center.

## Results

### Significantly Up-Regulated circRNAs in GSE 97332 and GSE 94508

**Figure 1:**
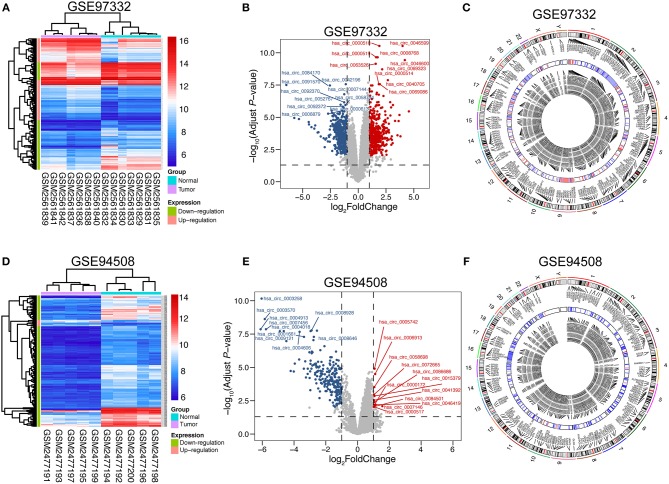
Differential expression of circRNAs in GSE 97332 and GSE 94508. **(A,D)** Hierarchical clustering analysis of circRNAs, which were differentially expressed between tumor and normal tissues; GSE 97332 included seven tumor tissues and seven matched non-tumor tissues, while GSE 94508 included 10 paired samples (>2-fold difference in expression; adjusted *P* < 0.01). Expression values are exhibited in different colors, indicating expression levels above and below the median expression level. **(B,E)** Volcano plots were constructed using fold-change values and adjusted *P*. The red point in the plot represents the over-expressed circRNAs and the green point indicates the down-expressed circRNAs with statistical significance. **(C,F)** Circos plots representing the chromosomal distributions of the differentially expressed circRNAs and their host genes, including the heatmap circle of averaged circRNA expression.

To assess variation in circRNA expression between tumor and normal liver tissues, hierarchical clustering analysis and volcano plots were used. The heat maps of GSE 94508 and GSE 97332 are shown in Figures 1A,D, respectively. To better visualize the differentially expressed circRNAs, volcano plots were performed and various top circrRNAs were labeled in the plot (Figures 1B,E). Notably, there are 235 down-regulated circRNAs and only 12 up-regulated circRNAs in GSE 94508 (detailed information in Table S2). As for GSE 97332, 824 circRNAs displayed significantly differential expression in HCC, including 422 up-regulated circRNAs and 402 down-regulated circRNAs (detailed information in Table S3). Circos diagrams were plotted to exhibit the chromosomal characteristics of the differentially expressed circRNAs in both GSE 97332 and GSE 94508. As shown in Figures 1C,F, these circRNAs and corresponding parental genes were widely apportioned in all chromosomes, including chromosomes X and Y.

### WGCNA Analysis Revealed That has_circ_0000517 Was Closely Relative to HCC

**Figure 2:**
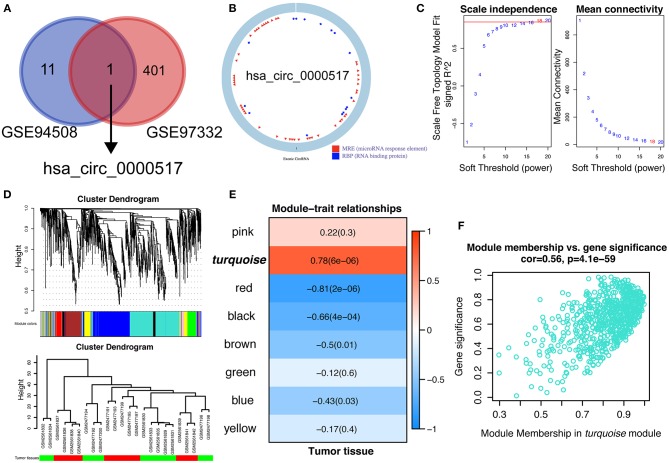
Identification of hub modules correlated with HCC in the datasets via WGCNA. **(A)** Comparison of differentially over-expressed circRNAs in both GSE 94508 and GSE 97332. Only hsa_circ_0000517 was overlapped. **(B)** The structure of hsa_circ_0000517 retrieved from CSCD database (http://gb.whu.edu.cn/CSCD/). **(C)** Analyses of the Scale-Free Topology Model Fit and the Mean Connectivity for different soft threshold powers. **(D)** Clustering dendrograms of circRNAs. The clustering was based on the combined data from GSE 97332 and GSE 94508. **(E)** The correlation between various modules and tumor tissues. The correlation coefficient and *P*-value were presented in the heatmap. **(F)** Scatter plot of module membership (circRNAs) in the turquoise module.

CircRNAs are known to be evolutionally conserved and relatively stable, accounting for the potential as prognostic biomarkers and possible treating targets for precise personalized medicine (23). In our current work, we aimed at exploring specifically up-regulated prognostic biomarkers for HCC patients, which would be much more practical for clinical detection and shed light on future pharmacological therapy. Therefore, in order to figure out the reliable circRNAs for further study, we overlapped the up-regulated circRNAs in GSE 97332 and GSE 94508 using Venn diagram. As shown in Figure 2A, with strict inclusion criteria (adjusted *P* < 0.01), only one circRNA, hsa_circ_0000517, was significantly upregulated in both GSE 94508 and GSE 97332. According to the molecular character of has_circ_0000517 from CSCD database, has_circ_0000517 has numerous microRNA response elements (MRE) and RNA binding protein (RBP) sites (Figure 2B). To further investigate the relationship between has_circ_0000517 and HCC, WGCNA analysis were utilized. Since the sample sizes of each datasets were too small to perform WGCNA analysis, we combined two datasets using batch normalization in R (version: 3.6.0). According to Figures 2C,D, data matrix of 24 samples was used in WGCNA analysis. The data was eligible and the best soft threshold (power) was 18. After analyzing and classifying all circRNAs, eight modules were identified. In view of the heatmap of module–trait relationships (Figure 2E), we could find that turquoise module was positively correlation with tumor tissues (*P* = 6e-06, correlation coefficient = 0.78). The turquoise module contained a total of 701 circRNAs, including has_circ_0000517 (Figure 2F). As presented in Table S4, has_circ_0000517 was closely associated with turquoise module (*P* = 2.43E-09 and the correlation coefficient = 0.90), highlighting the pivotal role of has_circ_0000517 in HCC.

### Sponge Function Exploration of has_circ_0000517 and Its Potential miRNAs and Targets in HCC

Hsa_circ_0000517, as one of the 27 circRNAs derived from Ribonuclease P RNA component H1 (RPPH1), embodies only one exon and is lack of an open reading frame (ORF). However, it consists of several microRNA response elements (MRE) (Figure 2B), indicating the potential miRNA-sponged function of has_circ_0000517. To date, the CSCD database, an open and online database, has identified 9 100 345 MREs in common circRNAs via scanning the junction region for miRNA seeds (7mer-m8, 7mer-1a, and 8mer). Therefore, to predict the sponged miRNAs of hsa_circ_0000517, the CSCD database was in use and 73 miRNAs were acquired from CSCD (Table S5). To develop a reliable prediction of the targets for these miRNAs, we utilized three miRNA-prediction databases, namely TargetScan, miRTarBase, and miRDB. As a result, we figured out that 1327 miRNA-target network edges. Discarding the redundancy, 742 genes were more likely to be targeted by these 60 miRNAs as a result of our study (Table S6).

### Functional Analysis of miRNA-Targeted Genes

**Figure 3:**
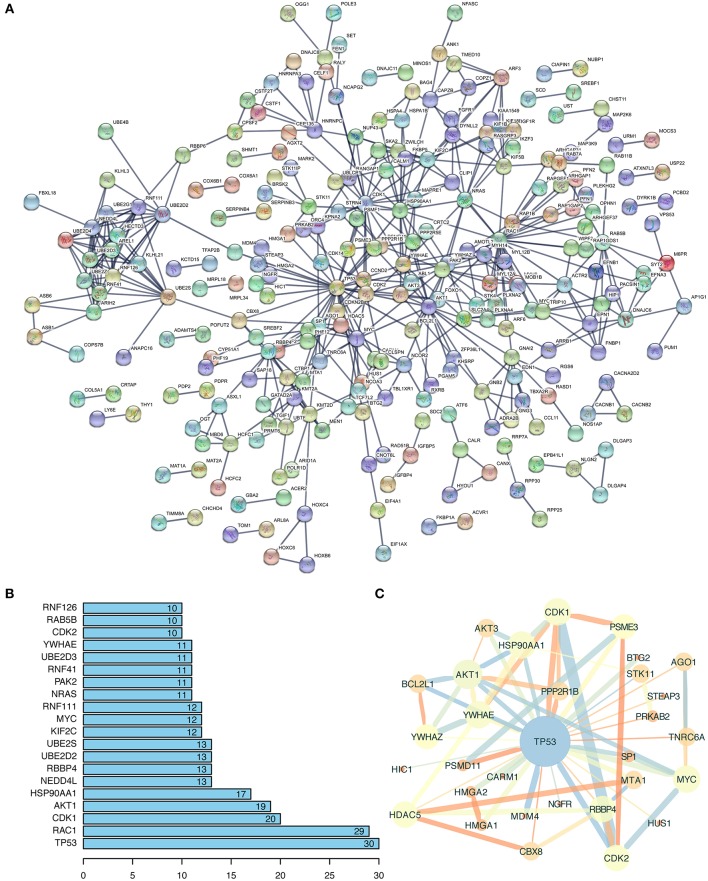
Function annotation of potential targeted genes. **(A)** The analysis results of Protein-Protein Network (PPI) from String Database (https://string-db.org). The disconnected nodes were hided. The line thickness indicated the strength of data support, including experiments, text-mining, databases, co-expression, neighborhood, gene fusion, and co-occurrence. **(B)** The top 20 interactive genes were exhibited, indicating the correlation degree with other genes. **(C)** The TP53-related network extracted from **(A)** using the MCODE plugin in Cytoscape (version: 3.7.1). The wider the edge and circle size, the larger the co-expression, and MCODE-scores are; the brighter the color, the lower the MCODE-score, and combined-score are.

So far, studies have demonstrated that circRNAs mainly exert their function by regulating the targeted miRNAs and genes. Therefore, figuring out the biological function of potential circRNA-related genes could predict the corresponding potential function of circRNAs (24). In the setting of the aforementioned String database, the 742 targets were used to construct a protein-protein network (Figure 3A). This interaction network had 742 nodes and 492 edges. The PPI enrichment *P*-value was smaller than 1.0e-16, and the analysis proved that the network had significantly more interactions than expected. As presented in Figure 3B, the top 20 interactive and important nodes of 742 potential targets were ranked. TP53, as the most interactive genes in top 20 and a member of MAPK pathway, has 30 interactive nodes with other genes. To better visualize the interaction of TP53 and its related genes, we imported the corresponding interactive data downloaded from String database into Cytoscape 3.7.1, and a TP53-related network was constructed (Figure 3C). We concluded that AKT1, CDK1, and MYC, also ranking top 20 interactive targets, were involved in TP53-related network, indicating the possibility that the regulatory relationship of these potential targets might occupy a commanding position in the hepatocarcinogenesis of hsa_circ_0000517.

### Enrichment Analyses to Predict the Probable Functions of Related Targets

**Figure 4:**
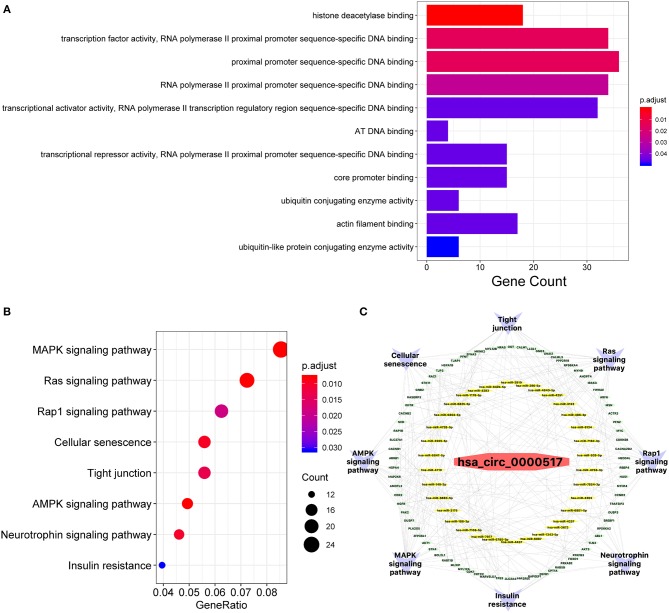
GO and KEGG functional analyses. **(A)** Gene ontology (GO) analysis of potential targets of miRNAs. The biological process (BP), cellular component (CC), and molecular function (MF) of potential targets were clustered based on ClusterProfiler package in R software (version: 3.12.0). The abscissa strands for gene counts and the ordinate means the enriched pathways. **(B)** The enriched KEGG signaling pathways were selected to demonstrate the primary biological actions of major potential targets. The abscissa indicates gene ratio and the enriched pathways were presented in the ordinate. **(C)** The has_circ_0000517 regulatory Network. The pathways, hub genes, miRNAs were presented in outer circle, middle circle and inner circle, respectively.

Given that a better comprehension of has_circ_0000517-related genes might shed light on specific molecular function characterized by has_circ_0000517. The GO enrichment analysis revealed that several targets were involved in “histone deacetylase binding,” “AT DNA binding,” “core promoter binding,” “ubiquitin conjugating enzyme activity,” “actin filament binding,” and “ubiquitin-like protein conjugating enzyme activity” (Figure 4A and Table S7). Importantly, lots of enriched terms were correlated with proximal promoter sequence-specific DNA, consistent with the special formation and structure of circRNAs (25). Therefore, has_circ_0000517 might take part in the carcinogenesis and progression of HCC by modulating transcription and oncogene expression. With respect to their close interactions with miRNAs, circRNAs exert great effect on modulating progression of malignancies and takes part in different signaling pathways in cancers like mitogen-activated protein kinase (MAPK), phosphatidylinositol 3-kinase (PI3K)/protein kinase B (AKT) intracellular pathway and Wnt/β-catenin pathway (26). In our study, with the help of clusterProfiler package in R software, eight signaling pathways of target genes were identified as followed: “MAPK signaling pathway,” “Ras signaling pathway,” “Rap1 signaling pathway,” “Cellular senescence,” “Tight junction,” “AMPK signaling pathway,” “Neurotrophin signaling pathway,” and “insulin resistance” (Figure 4B). Notably, The MAPK pathway included 26 key potential genes, such as TP53, MYC, and AKT1, while Ras signaling pathway included 22 key targets, such as BCL2L1, AKT1, and AKT3. These molecules mentioned above were also identified as top 20 interactive proteins in Figure 3B and those mostly involved in TP53-related network were revealed in Figure 3C. In all, has_circ_0000517 might indirectly regulate these 8-key signaling pathways, especially MAPK pathway. On the other hand, to elucidate the relationship among the upregulated hsa_circ_0000517, key miRNAs, hub genes, and enriched signaling pathways, a network diagram was created (Figure 4C). In this circRNA-miRNA-target-pathway network, we concluded that has_circ_0000517 might be capable of sponging at least one of these 34 miRNAs. Additionally, eight hub signaling pathways contained 78 hub genes that might be regulated by has_circ_0000517.

### Validation of the Differential Expression and Potential Prognostic Value of hsa_circ_0000517 by qRT-PCR

**Figure 5:**
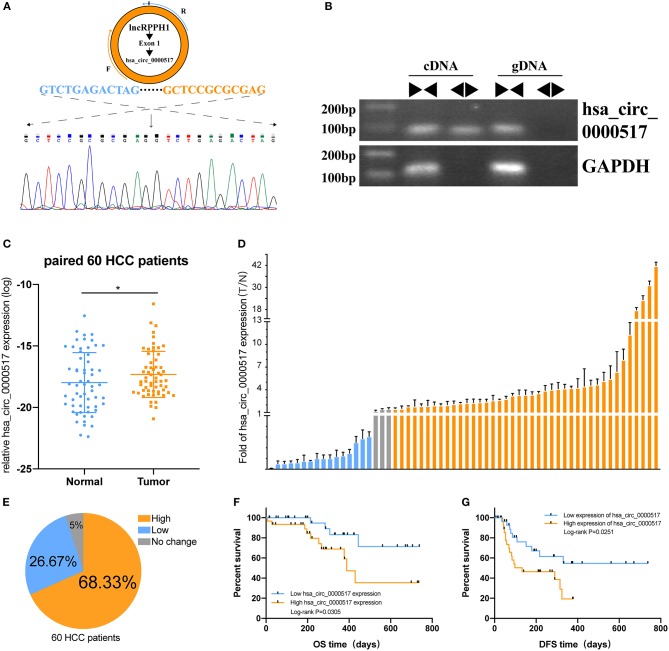
The prognostic value of hsa_circ_0000517 in 60 HCC patients. **(A)** Sanger sequencing was performed following PCR to validate the head-to-tail splicing of has_circ_0000517 in SNU-387 cells. **(B)** The validation of RPPH1 circular exon 1, has_circ_0000517. Divergent and convergent primers were used to perform PCR in both cDNA and gDNA. GAPDH was used as a control. **(C)** The differences in the expression of has_circ_0000517 in 60 tumor and paired normal tissues. *P < 0.05. **(D)** The Fold Changes (FC) of hsa_circ_0000517 expression in 60 HCC patients (tumor vs. matched non-tumor tissue). Data are represented as the mean ± SD of three independent experiments. Different colors stand for three different groups, including “FC > 1.5,” “FC < 0.67,” and “0.67 < FC < 1.5.” **(E)** Distribution of three groups in 60 HCC patients. **(F)** Overall Survival Curve (OS) of has_circ_0000517. **(G)** Disease-free survival (DFS) curve of has_circ_0000517.

Given that has_circ_0000517 has been upregulated in both GSE 97332 and GSE 94508, qRT-PCR experiments were carried out to verify the differential hsa_circ_0000517 expression in 60 tumor and paired non-tumor tissues. First of all, by conducting Electrophoresis and Sanger sequencing of qRT-PCR products, we validated and confirmed the specificity and accuracy of the primers of has_circ_0000517 (Figures 5A,B). Then, as shown in Figure 5C, has_circ_0000517 was significantly up-regulated in tumor tissues compared with paired normal tissues (*P* = 0.0278). Moreover, referring to the criteria [*P* < 0.05, Fold Change (FC) > 1.5 or < 0.67], we separated 60 patients into three groups: High-expression group, No-change group, and Low-expression group. Among 60 HCC patients in our center, 41 (68.33%) patients exhibited high hsa_circ_0000517 expression in tumor tissues compared with non-tumor tissues (Figures 5D,E). The low-expression group accounted for only 26.67% HCC patients and the no-change group was responsible for 5% HCC patients. On the other hand, we could find that high expression of hsa_circ_0000517 resulted in poor prognosis of HCC patients, considering both overall survival (OS, *P* = 0.0305) as well as disease-free survival duration (DFS, *P* = 0.0251) (Figures 5F,G). As shown in Table 1, the patients with high expression of hsa_circ_0000517 were prone to be in a significantly higher TNM stage, another indicator of prognosis prediction. In all, the results demonstrated that hsa_circ_0000517 might be closely associated with the progression of HCC.

**Table 1:** Correlation between hsa_circ_0000517 expression and clinicopathological characteristics of 60 HCC patients.

**Clinicopathological characteristics**	**hsa_circ_0000517 expression**		***P*-value**	**χ^2^**
	**Low (30)**	**High (30)**		
Age (years)			0.26	1.27
≤50	11	7		
>50	19	23		
Gender			0.09	2.96
Male	25	29		
Female	5	1		
Hepatitis virus infection			1	0
HBsAg(+)	24	24		
HBsAg(–)	6	6		
Child–Pugh			0.31	1.02
A	30	29		
B	0	1		
C	0	0		
Cirrhosis			0.80	0.07
Yes	14	15		
No	16	15		
AFP (μg/L)			0.44	0.60
≤400	17	14		
>400	13	16		
TNM stage			0.02*	5.71
I+II	16	7		
III+IV	14	23		
Tumor size (cm)			0.09	2.86
≤5	12	6		
>5	18	24		
Vascular invasion			0.18	1.83
Yes	17	22		
No	13	8		
Histological grade			0.67	0.81
G1	4	2		
G2	13	13		
G3	13	15		

(a) The median expression value of hsa_circ_0000517 was used as the cutoff. Low hsa_circ_0000517 expression in each of the 30 patients was defined as a value below the 50th percentile. (b) χ^2^ test, ^*^P < 0.05. (c) TNM stage: T, Tumor; N, nodes; M, Metastasis. (d) AFP, alpha-fetoprotein; HBsAg, the hepatitis B surface antigen.

### Confirming the Potential Prognostic Value of hsa_circ_0000517 via Cox Regression Analyses

**Figure 6:**
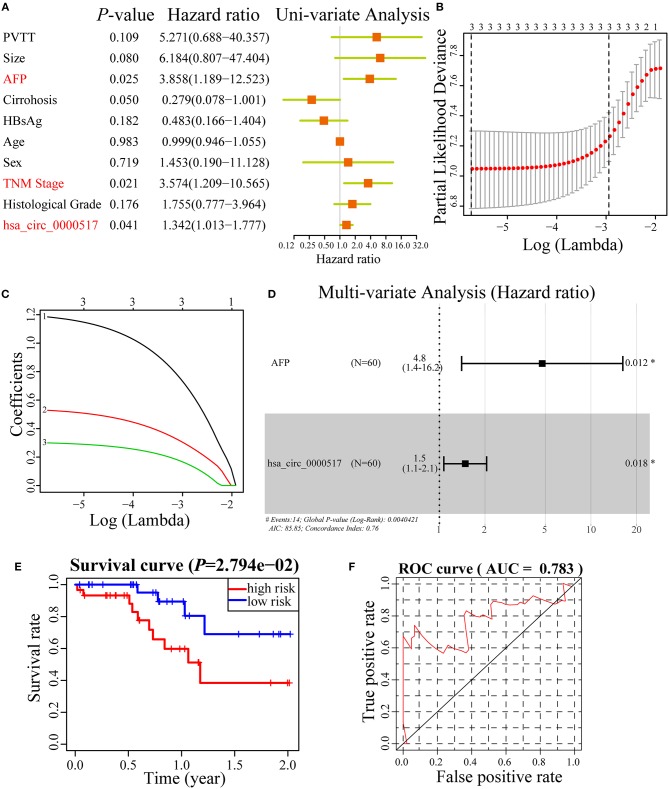
Cox regression analyses in 60 HCC patients. **(A)** Uni-variate analysis of 10 risk factors in HCC patients. “*P* < 0.05” was selected for further study. **(B,C)** Lasso regression analysis of three risk factors. **(D)** Multi-variated analysis of three risk factors and only two was considered significant and used to construct a prognostic model. The formula was as followed: Risk Score = 1.56*AFP + 0.39*has_circ_0000517. The C-index was 0.76. **(E)** Survival curve of high-risk group and low-risk group in 60 HCC patients based on Risk Score model. **(F)** The ROC curve of the Risk Score model.

A good prognostic biomarker is able to construct a signature or nomogram with other risk factors (27). To further study the underlying power of has_circ_0000517 in HCC, we used Uni-variate analysis, Lasso analysis and Multi-variate analysis to testify and verify the risk factors in HCC patients. As depicted in Figure 6A, only alpha-fetoprotein (AFP), TNM stage and has_circ_0000517 were regarded as significant risk factors in 60 HCC patients. With the purpose of obtaining a more precise result, Lasso analysis were performed in three risk factors while none of them were filtered or discarded (Figures 6B,C). Thereafter, we conducted Multi-variate analysis with the three risk factors and as a result, only two were selected to establish a prognostic nomogram: Risk Score = 1.56*AFP + 0.39*has_circ_0000517. The C-index was 0.76 (Figure 6D). Based on the prognostic nomogram, 60 HCC patients were classified into two groups as high-risk group and low-risk group. According to Figure 6E, we could figure out that HCC patients with high-risk worsen the survival status (*P* = 2.794e-02). Moreover, the ROC curve of the prognostic nomogram was plotted in Figure 6F. The AUC of the nomogram was 0.783, indicating the acceptable prognostic value of the nomogram built on has_circ_0000517 and AFP (Figure 6F).

## Discussion

Recent studies have reported that circRNAs could be transcribed and involved in several diseases as the expression of circRNAs varied in neither histologic types nor disease stages (4, 28). In this work, we first thoroughly explored the hsa_circ_0000517-related network of miRNAs, mRNAs, and signaling pathways in HCC. Functional analysis revealed the promising role of hsa_circ_0000517 in hepatocarcinogenesis and development *in silico*. Furthermore, the qRT-PCR result confirmed its differential expression in cancerous and para cancerous tissues. Finally, we validated the prognostic value of has_circ_0000517, via cox and Lasso regression analyses, in HCC patients.

Much attention has been paid to investigate the relation between circRNAs and HCC (29). Qin et al. (30) demonstrated that hsa_circ_0001649 is significantly down-regulated in HCC compared to para cancerous samples. Similarly, Wei et al. (31) revealed that circ-CDYL takes a specific part in the early HCC. Another study reported that circ_0005075 resists mir-335 and enhances HCC progression (32). Furthermore, three isoforms of exosomal circ-PTGR1 were found to induce HCC metastasis through the miR449a–MET pathway (33). Xiong et al. (34) used GSE78520, GSE 94508, and GSE 97332 to unearth new circRNAs in HCC with the RobustRankAggreg method and concluded three differentially expressed circRNAs, namely hsa_circRNA_102166, hsa_circRNA_100291 and hsa_circRNA_104515. Additionally, a more recent study has also revealed that hsa_circ_0000517 was significantly up-regulated in these three datasets while it cannot show significant difference in 50 HCC patients from their center (35). However, we analyzed the data of GSE 97332 and GSE 94508 with a much stricter criteria (adjusted *P* < 0.01) and just intersected up-regulated circRNAs between the datasets. Due to the heterogeneity of HCC patients, we figured out that hsa_circ_0000517 was the single significantly up-regulated molecular, further confirmed by WGCNA analysis. Moreover, a consistent result of 60 HCC patients from our center was validated. Given the samples across studies above were small and bias might be unavoidable, future hsa_circ_0000517-related researches urge to enroll participants with a large scale, and gain stronger evidence in the comparison of tumor and non-tumor HCC tissues.

In this study, we constructed a quadruple network of hsa_circ_0000517 based on bioinformatics analysis. The hsa_circ_0000517 regulatory network was composed of 34 miRNAs, 78 mRNAs, and eight signaling pathways (Figure 5B), and this network vividly exhibited the connection between four parts, indicating the underlying regulatory mechanisms of hsa_circ_0000517. Specifically, pathway analysis revealed that the MAPK, Ras, Rap1, and AMPK signaling pathways, previously associated with the development of HCC, were significantly enriched (36–39). Additionally, the enriched GO terms for the miRNA targets were highly related to carcinogenesis like regulation of transcription and DNA binding. The results of PPI network also highlighted several famous targets, including TP53, MYC, AKT, RAC1, and CDK1, consistent with the eight enriched signaling pathways where MAPK signaling pathway and Ras signaling pathway were the most significant.

Hsa_circ_0000517 might have undiscovered functions due to its several MREs and RBPs. To further verify the changes in hsa_circ_0000517 expression level and its clinical efficacy in patients with HCC, 60 pairs of tumor and adjacent tissues were collected to examine the expression of hsa_circ_0000517 using qRT-PCR assay. Results showed that hsa_circ_0000517 was up-regulated in 41 (68.3%) HCC patients in our center. Moreover, the OS and DFS curves illustrated that hsa_circ_0000517 could account for the poor diagnosis and play a carcinogenic role in the development of HCC (Figures 6B,C). In addition, high hsa_circ_0000517 expression was also related to higher TNM stage among our patients (Table 1), indicating that has_circ_0000517 was characterized by HCC-stage-specific expression. Uni-variate, Lasso and Multi-variate analyses indicated that has_circ_0000517 could be a prognostic indicator for HCC patients. Moreover, we even tested the expression of serum has_circ_0000517 in 5 normal blood samples and 10 HCC blood samples. The result revealed that higher hsa_circ_0000517 expression in the serum of HCC patients than that in normal controls (*P* = 0.0312), indicating the potential prognostic role of the serum hsa_circ_0000517 (Figure S1). However, the precise oncogenic and prognostic mechanism of hsa_circ_0000517 still requires particular investigation *in vitro* and *in vivo* experiments.

On the other hand, RPPH1, as the host gene of has_circ_0000517, is the RNA component of the RNase P ribonucleoprotein (40) and considered as a long non-coding RNA. Previous studies reported that RPPH1 was up-regulated in gastric cancer specimens (41). And lncRNA RPPH1 can promote breast cancer cell proliferation and tumorigenesis via down-regulating miR-122 expression (42). But the regulatory mechanisms of RPPH1 in other malignancies remain largely obscure. has_circ_0000517, derived from RPPH1, might be accountable for the carcinogenesis of RPPH1 owing to the close relationship between circRNAs and host genes. Further validated studies will be taken in our future work.

In summary, hsa_circ_0000517 was highly expressed in HCC, verified *in silico* and in 60 HCC patients of our center. Bioinformatics analysis also suggested that sponging several miRNAs, hsa_circ_0000517 might regulate the expression of TP53, MYC, and AKT1 via MAPK pathway and Ras pathway. Our findings indicated that hsa_circ_0000517 might not only be an undeveloped prognostic biomarker of HCC, but also accountable for hepatocarcinogenesis.

## Data Availability Statement

Publicly available datasets were analyzed in this study. These data can be found here: GSE97332; GSE94508.

## Ethics Statement

The studies involving human participants were reviewed and approved by the Ethics Committee of Sun Yat-Sen Memorial Hospital. The patients/participants provided their written informed consent to participate in this study.

## Author Contributions

TC and JC designed and supervised the study. XicW and XinW performed the specific procedures and wrote the manuscript. WL analyzed the data and made the pictures and graphs. QZ revised the final manuscript. All the authors have read and approved the manuscript.

### Conflict of Interest

The authors declare that the research was conducted in the absence of any commercial or financial relationships that could be construed as a potential conflict of interest.
