# Supplementary material for: Up-Regulation of hsa_circ_0000517 Predicts Adverse Prognosis of Hepatocellular Carcinoma
Source: Front Oncol. 2019 Oct 22;9:1105. doi: 10.3389/fonc.2019.01105 (PMC6842961; doi:10.3389/fonc.2019.01105)
Supplement: Supplementary file 1 [file Table_1.DOCX]

**Table S1 Clinicopathological characteristics of 60 HCC patients.**

| Clinicopathological characteristics | Number of cases(%) |
| --- | --- |
| Age (years) |  |
| ≤ 50 | 18(30%) |
| > 50 | 42(70%) |
| Gender |  |
| Male | 54(90%) |
| Female | 6(10%) |
| Hepatitis virus infection |  |
| HBsAg(+) | 48(80%) |
| HBsAg(-) | 12(20%) |
| Child-Pugh |  |
| A | 59(98.3%) |
| B | 1(1.7%) |
| C |  |
| Cirrhosis |  |
| Yes | 29(48.3%) |
| No | 31(51.7%) |
| AFP (μg/L) |  |
| ≤ 400 | 31(51.7%) |
| > 400 | 29(48.3%) |
| TNM stage |  |
| I | 12(20%) |
| II | 11(18.3%) |
| III | 34(56.7%) |
| IV | 3(5%) |
| Tumor size (cm) |  |
| ≤ 5 | 18(30%) |
| > 5 | 42(70%) |
| Vascular invasion |  |
| Yes | 39(65%) |
| No | 21(35%) |
| Histological grade |  |
| G1 | 6(10%) |
| G2 | 26(43.3%) |
| G3 | 28(46.7) |
