# Supplementary material for: Up-Regulation of hsa_circ_0000517 Predicts Adverse Prognosis of Hepatocellular Carcinoma
Source: Front Oncol. 2019 Oct 22;9:1105. doi: 10.3389/fonc.2019.01105 (PMC6842961; doi:10.3389/fonc.2019.01105)
Supplement: Supplementary file 2 [file Table_2.DOCX]

**Table S2 248 differential expressed circRNAs in GSE 94508.**

| ID | logFC | AveExpr | t | *P*.Value | adj.*P*.Val | B |
| --- | --- | --- | --- | --- | --- | --- |
| hsa_circ_0003258 | -6.04338231 | 10.6716936 | -68.17998702 | 2.68E-14 | 6.78E-11 | 22.19058495 |
| hsa_circ_0003570 | -5.839372336 | 9.727182941 | -43.88494303 | 1.87E-12 | 2.37E-09 | 19.01956433 |
| hsa_circ_0007456 | -5.765255776 | 9.156704997 | -34.29009005 | 2.01E-11 | 1.37E-08 | 16.90981188 |
| hsa_circ_0004913 | -6.098960507 | 9.277582441 | -34.03220908 | 2.16E-11 | 1.37E-08 | 16.8424021 |
| hsa_circ_0008928 | -2.896134236 | 9.430208853 | -32.58789392 | 3.27E-11 | 1.66E-08 | 16.45233635 |
| hsa_circ_0008646 | -2.555168059 | 7.965281352 | -31.76428516 | 4.18E-11 | 1.76E-08 | 16.21992912 |
| hsa_circ_0004018 | -4.643280742 | 8.377422529 | -30.63784831 | 5.91E-11 | 1.87E-08 | 15.88955211 |
| hsa_circ_0001661 | -2.954060372 | 9.881356311 | -30.63033373 | 5.92E-11 | 1.87E-08 | 15.88729768 |
| hsa_circ_0009121 | -4.89195611 | 9.183424258 | -30.17922371 | 6.83E-11 | 1.92E-08 | 15.75069728 |
| hsa_circ_0004606 | -3.65179301 | 9.268074752 | -29.52584049 | 8.42E-11 | 2.13E-08 | 15.54833647 |
| hsa_circ_0074543 | -3.375844343 | 8.659585128 | -26.61141895 | 2.27E-10 | 5.10E-08 | 14.57493691 |
| hsa_circ_0054843 | -2.75277219 | 7.69320642 | -26.44258887 | 2.42E-10 | 5.10E-08 | 14.5147082 |
| hsa_circ_0085045 | -2.780782234 | 8.880713759 | -24.71892621 | 4.60E-10 | 8.95E-08 | 13.87298737 |
| hsa_circ_0043138 | -4.480405136 | 9.78725075 | -24.070198 | 5.92E-10 | 1.07E-07 | 13.61802175 |
| hsa_circ_0001974 | -2.083863618 | 7.437777421 | -21.77868691 | 1.53E-09 | 2.59E-07 | 12.65127384 |
| hsa_circ_0038825 | -2.346806429 | 7.656730376 | -20.1246517 | 3.24E-09 | 5.13E-07 | 11.88105775 |
| hsa_circ_0002504 | -2.880387781 | 8.209390618 | -19.37298757 | 4.65E-09 | 6.55E-07 | 11.5081853 |
| hsa_circ_0062165 | -1.355278476 | 7.730500727 | -19.36752859 | 4.66E-09 | 6.55E-07 | 11.50542128 |
| hsa_circ_0004459 | -3.032309036 | 8.201452621 | -18.95460421 | 5.71E-09 | 7.31E-07 | 11.29392039 |
| hsa_circ_0083035 | -2.836479935 | 8.611352606 | -18.931519 | 5.78E-09 | 7.31E-07 | 11.28195279 |
| hsa_circ_0006960 | -2.88951363 | 8.703427181 | -18.49060398 | 7.21E-09 | 8.69E-07 | 11.05038482 |
| hsa_circ_0023406 | -1.714055133 | 7.274351443 | -17.03776676 | 1.56E-08 | 1.72E-06 | 10.24453082 |
| hsa_circ_0078017 | -1.633920126 | 7.119831365 | -17.03101002 | 1.56E-08 | 1.72E-06 | 10.24061971 |
| hsa_circ_0044949 | -3.679153315 | 8.748554037 | -16.26224351 | 2.41E-08 | 2.54E-06 | 9.78498319 |
| hsa_circ_0027641 | -1.312578577 | 7.799333629 | -16.06446374 | 2.70E-08 | 2.74E-06 | 9.664233267 |
| hsa_circ_0000586 | -1.195180288 | 8.563654995 | -15.80455099 | 3.15E-08 | 3.06E-06 | 9.50325689 |
| hsa_circ_0070805 | -2.953248659 | 7.823058001 | -15.69104278 | 3.37E-08 | 3.09E-06 | 9.432122091 |
| hsa_circ_0008157 | -1.733000582 | 7.397256891 | -15.66351017 | 3.42E-08 | 3.09E-06 | 9.414790102 |
| hsa_circ_0000841 | -3.294740719 | 8.731926276 | -15.39971878 | 4.01E-08 | 3.50E-06 | 9.247174958 |
| hsa_circ_0006248 | -2.484123933 | 8.185461389 | -15.20786914 | 4.51E-08 | 3.80E-06 | 9.123470031 |
| hsa_circ_0045602 | -2.273921195 | 10.88642389 | -15.13735239 | 4.70E-08 | 3.84E-06 | 9.077611827 |
| hsa_circ_0006736 | -1.524691399 | 7.331423103 | -15.00177135 | 5.12E-08 | 4.05E-06 | 8.988845131 |
| hsa_circ_0001601 | -1.752592712 | 7.344197725 | -14.5824538 | 6.66E-08 | 5.11E-06 | 8.709229887 |
| hsa_circ_0061774 | -2.61655357 | 8.349454034 | -14.50990845 | 6.97E-08 | 5.19E-06 | 8.660055967 |
| hsa_circ_0003340 | -3.952334971 | 9.962252425 | -14.45311828 | 7.23E-08 | 5.23E-06 | 8.621393713 |
| hsa_circ_0053394 | -2.96533387 | 8.785286119 | -14.31552063 | 7.90E-08 | 5.37E-06 | 8.527101114 |
| hsa_circ_0088154 | -1.364147347 | 7.221120088 | -14.29440708 | 8.01E-08 | 5.37E-06 | 8.512554481 |
| hsa_circ_0036666 | -2.669586423 | 9.539930263 | -14.28418636 | 8.06E-08 | 5.37E-06 | 8.505505192 |
| hsa_circ_0007395 | -2.754035566 | 8.365972722 | -13.99575822 | 9.74E-08 | 6.32E-06 | 8.304531356 |
| hsa_circ_0038929 | -1.721187334 | 7.994970677 | -13.9417467 | 1.01E-07 | 6.39E-06 | 8.2664518 |
| hsa_circ_0008995 | -2.155057197 | 8.155741602 | -13.7775197 | 1.13E-07 | 6.95E-06 | 8.149787912 |
| hsa_circ_0080451 | -1.50564932 | 7.711251511 | -13.73865462 | 1.16E-07 | 6.97E-06 | 8.121983182 |
| hsa_circ_0000602 | -2.844907099 | 8.206932318 | -13.65200831 | 1.23E-07 | 7.22E-06 | 8.059722217 |
| hsa_circ_0080212 | -2.605424795 | 7.867451747 | -13.54548388 | 1.32E-07 | 7.58E-06 | 7.982656632 |
| hsa_circ_0007402 | -1.22787279 | 6.828695345 | -13.50857941 | 1.35E-07 | 7.60E-06 | 7.955822682 |
| hsa_circ_0005428 | -2.392684546 | 8.469568532 | -13.29067858 | 1.57E-07 | 8.45E-06 | 7.795943244 |
| hsa_circ_0008035 | -2.550828696 | 8.08343532 | -13.15022239 | 1.73E-07 | 9.13E-06 | 7.69156067 |
| hsa_circ_0004194 | -2.451094022 | 7.772533196 | -12.96869504 | 1.97E-07 | 1.00E-05 | 7.555080711 |
| hsa_circ_0042817 | -2.582688922 | 8.690196812 | -12.94673437 | 2.00E-07 | 1.00E-05 | 7.538447638 |
| hsa_circ_0001286 | -1.58007309 | 7.469221953 | -12.93001204 | 2.02E-07 | 1.00E-05 | 7.525764265 |
| hsa_circ_0004148 | -1.13317618 | 6.840816697 | -12.82496149 | 2.18E-07 | 1.06E-05 | 7.44573175 |
| hsa_circ_0008471 | -1.014988675 | 6.873286371 | -12.5801196 | 2.60E-07 | 1.23E-05 | 7.256786058 |
| hsa_circ_0001280 | -1.892807649 | 9.066887844 | -12.56319768 | 2.63E-07 | 1.23E-05 | 7.243600595 |
| hsa_circ_0006677 | -1.089946398 | 7.737702228 | -12.4931635 | 2.77E-07 | 1.24E-05 | 7.188853689 |
| hsa_circ_0073997 | -1.355298291 | 7.446586252 | -12.48676574 | 2.78E-07 | 1.24E-05 | 7.183838211 |
| hsa_circ_0077495 | -1.642336178 | 7.212794735 | -12.45762205 | 2.84E-07 | 1.24E-05 | 7.160960898 |
| hsa_circ_0005417 | -2.901209298 | 8.606890598 | -12.41422751 | 2.94E-07 | 1.24E-05 | 7.126804501 |
| hsa_circ_0067231 | -3.136859478 | 7.878861765 | -12.40335471 | 2.96E-07 | 1.24E-05 | 7.118228995 |
| hsa_circ_0005935 | -1.424069569 | 6.940223294 | -12.40236647 | 2.96E-07 | 1.24E-05 | 7.117449209 |
| hsa_circ_0072788 | -3.381654999 | 8.811820818 | -12.39306821 | 2.98E-07 | 1.24E-05 | 7.110109475 |
| hsa_circ_0005742 | 1.066970882 | 7.505343755 | 12.35675628 | 3.06E-07 | 1.25E-05 | 7.081397017 |
| hsa_circ_0000837 | -1.15982439 | 7.331908395 | -12.31931414 | 3.15E-07 | 1.27E-05 | 7.051708791 |
| hsa_circ_0066151 | -2.171938272 | 8.286652823 | -12.25245171 | 3.31E-07 | 1.31E-05 | 6.998484388 |
| hsa_circ_0092371 | -1.095016846 | 6.84121217 | -12.2009885 | 3.44E-07 | 1.34E-05 | 6.957334881 |
| hsa_circ_0073992 | -1.336598459 | 7.155642595 | -11.91388479 | 4.27E-07 | 1.64E-05 | 6.724791288 |
| hsa_circ_0021553 | -1.796131214 | 7.096332666 | -11.81424587 | 4.61E-07 | 1.74E-05 | 6.64288404 |
| hsa_circ_0080210 | -2.332982156 | 8.809662186 | -11.75194403 | 4.84E-07 | 1.80E-05 | 6.591348096 |
| hsa_circ_0007542 | -1.726798219 | 7.699160691 | -11.710064 | 5.00E-07 | 1.82E-05 | 6.556564843 |
| hsa_circ_0075026 | -2.477230989 | 7.813031619 | -11.70032731 | 5.04E-07 | 1.82E-05 | 6.548461858 |
| hsa_circ_0074146 | -4.208802063 | 8.342724544 | -11.65634401 | 5.21E-07 | 1.86E-05 | 6.511781807 |
| hsa_circ_0037353 | -1.438003803 | 7.925127109 | -11.59454326 | 5.47E-07 | 1.92E-05 | 6.460029729 |
| hsa_circ_0028502 | -2.21199059 | 7.265043539 | -11.5469559 | 5.68E-07 | 1.97E-05 | 6.420009152 |
| hsa_circ_0013617 | -2.986588357 | 8.433778152 | -11.50141612 | 5.89E-07 | 2.00E-05 | 6.381570431 |
| hsa_circ_0004855 | -2.516570061 | 7.468599924 | -11.47538364 | 6.01E-07 | 2.00E-05 | 6.359535326 |
| hsa_circ_0077210 | -2.243347103 | 7.985881347 | -11.47496843 | 6.01E-07 | 2.00E-05 | 6.359183503 |
| hsa_circ_0008356 | -4.01260427 | 8.018449779 | -11.44698272 | 6.15E-07 | 2.02E-05 | 6.335443876 |
| hsa_circ_0001450 | -2.536304465 | 7.901628312 | -11.43357363 | 6.21E-07 | 2.02E-05 | 6.324050678 |
| hsa_circ_0000264 | -2.524861439 | 8.776579033 | -11.36533762 | 6.56E-07 | 2.10E-05 | 6.265885754 |
| hsa_circ_0017979 | -1.925182254 | 7.651211005 | -11.30566597 | 6.88E-07 | 2.15E-05 | 6.214762817 |
| hsa_circ_0008114 | -2.571873321 | 7.753134521 | -11.21923109 | 7.37E-07 | 2.24E-05 | 6.140279267 |
| hsa_circ_0001826 | -1.788816448 | 7.088179074 | -11.14408851 | 7.83E-07 | 2.33E-05 | 6.075107583 |
| hsa_circ_0015438 | -2.667883643 | 9.297820765 | -10.98956901 | 8.88E-07 | 2.61E-05 | 5.939849298 |
| hsa_circ_0003810 | -1.555697959 | 7.22711401 | -10.95579832 | 9.13E-07 | 2.66E-05 | 5.910062906 |
| hsa_circ_0073993 | -1.324058199 | 7.418804901 | -10.94027838 | 9.25E-07 | 2.66E-05 | 5.896346664 |
| hsa_circ_0007591 | -2.313082641 | 9.136709524 | -10.86537417 | 9.84E-07 | 2.80E-05 | 5.829904124 |
| hsa_circ_0044413 | -3.142128948 | 8.698694362 | -10.72933171 | 1.10E-06 | 3.07E-05 | 5.708187131 |
| hsa_circ_0006913 | 1.101243925 | 7.249283107 | 10.68724892 | 1.14E-06 | 3.13E-05 | 5.670260163 |
| hsa_circ_0039844 | -1.604922946 | 7.517339988 | -10.67555719 | 1.15E-06 | 3.13E-05 | 5.659699724 |
| hsa_circ_0092329 | -1.393618808 | 7.219090421 | -10.66672122 | 1.16E-06 | 3.13E-05 | 5.651711971 |
| hsa_circ_0038436 | -2.209313348 | 7.963819552 | -10.63455568 | 1.19E-06 | 3.18E-05 | 5.62258504 |
| hsa_circ_0008838 | -2.062619974 | 9.134992847 | -10.56929299 | 1.26E-06 | 3.32E-05 | 5.563249383 |
| hsa_circ_0053958 | -1.444748739 | 7.612837577 | -10.55772028 | 1.27E-06 | 3.32E-05 | 5.552694207 |
| hsa_circ_0063577 | -2.671803641 | 8.940991013 | -10.49290263 | 1.35E-06 | 3.46E-05 | 5.49338804 |
| hsa_circ_0001214 | -1.785083989 | 7.168259301 | -10.32331973 | 1.56E-06 | 3.94E-05 | 5.336701209 |
| hsa_circ_0085173 | -2.296955022 | 8.145597837 | -10.26352119 | 1.64E-06 | 4.11E-05 | 5.280917118 |
| hsa_circ_0038687 | -1.453206161 | 7.013725444 | -10.23628957 | 1.68E-06 | 4.17E-05 | 5.255420439 |
| hsa_circ_0000296 | -2.547260099 | 8.399081338 | -10.12465248 | 1.85E-06 | 4.55E-05 | 5.15028054 |
| hsa_circ_0084595 | -2.362561429 | 10.12857471 | -10.00780997 | 2.05E-06 | 4.95E-05 | 5.039165908 |
| hsa_circ_0000472 | -1.920666709 | 6.882856038 | -9.965159006 | 2.13E-06 | 5.09E-05 | 4.998329076 |
| hsa_circ_0005442 | -1.424741659 | 7.434981517 | -9.906539898 | 2.25E-06 | 5.32E-05 | 4.941959332 |
| hsa_circ_0017953 | -1.412556133 | 7.368032617 | -9.88569312 | 2.29E-06 | 5.37E-05 | 4.921844026 |
| hsa_circ_0001600 | -1.685081624 | 7.30506102 | -9.839334285 | 2.39E-06 | 5.54E-05 | 4.876982293 |
| hsa_circ_0083299 | -1.197719204 | 6.985635312 | -9.801428238 | 2.47E-06 | 5.67E-05 | 4.840166912 |
| hsa_circ_0076251 | -3.24747799 | 8.19464642 | -9.782798668 | 2.51E-06 | 5.67E-05 | 4.822029147 |
| hsa_circ_0073030 | -1.158801593 | 7.427805817 | -9.775338556 | 2.53E-06 | 5.67E-05 | 4.814757778 |
| hsa_circ_0002862 | -1.629645262 | 7.102454328 | -9.774451775 | 2.53E-06 | 5.67E-05 | 4.813893122 |
| hsa_circ_0005378 | -1.662581511 | 7.044234918 | -9.725573643 | 2.65E-06 | 5.88E-05 | 4.766131579 |
| hsa_circ_0002492 | -3.020091646 | 8.490703913 | -9.656553917 | 2.82E-06 | 6.20E-05 | 4.698342578 |
| hsa_circ_0030292 | -2.200330888 | 8.283296041 | -9.445961204 | 3.42E-06 | 7.28E-05 | 4.48896214 |
| hsa_circ_0000325 | -2.015986026 | 10.34649607 | -9.422404516 | 3.50E-06 | 7.32E-05 | 4.465299419 |
| hsa_circ_0000142 | -1.673143568 | 7.620759938 | -9.287149011 | 3.97E-06 | 7.98E-05 | 4.328477495 |
| hsa_circ_0022723 | -1.589307301 | 10.07039278 | -9.254987106 | 4.10E-06 | 8.01E-05 | 4.29570089 |
| hsa_circ_0007958 | -2.645698578 | 7.905376882 | -9.253468104 | 4.10E-06 | 8.01E-05 | 4.294150535 |
| hsa_circ_0018992 | -2.393449925 | 8.003602781 | -9.250961714 | 4.11E-06 | 8.01E-05 | 4.291591955 |
| hsa_circ_0062760 | -1.726188045 | 7.753929611 | -9.152912966 | 4.52E-06 | 8.54E-05 | 4.191051502 |
| hsa_circ_0090080 | -1.666644629 | 10.63473711 | -9.149141748 | 4.53E-06 | 8.54E-05 | 4.187166832 |
| hsa_circ_0068189 | -1.107347663 | 7.170449205 | -9.14383368 | 4.55E-06 | 8.54E-05 | 4.181696856 |
| hsa_circ_0004313 | -2.325247495 | 8.680363903 | -9.064131856 | 4.92E-06 | 9.08E-05 | 4.099250224 |
| hsa_circ_0088036 | -1.250291931 | 7.059179465 | -9.016453267 | 5.15E-06 | 9.40E-05 | 4.049646677 |
| hsa_circ_0001009 | -1.323956215 | 7.62502473 | -9.013649632 | 5.16E-06 | 9.40E-05 | 4.046723213 |
| hsa_circ_0062397 | -2.034481674 | 9.576035368 | -8.979548066 | 5.34E-06 | 9.65E-05 | 4.011104892 |
| hsa_circ_0007996 | -1.229446715 | 7.469103461 | -8.967582338 | 5.40E-06 | 9.66E-05 | 3.99858095 |
| hsa_circ_0035875 | -1.645433742 | 6.903928266 | -8.963604206 | 5.42E-06 | 9.66E-05 | 3.994414234 |
| hsa_circ_0001172 | -1.883490018 | 7.328058926 | -8.930662005 | 5.60E-06 | 9.84E-05 | 3.959852752 |
| hsa_circ_0001615 | -1.281817912 | 7.294572786 | -8.863538113 | 5.98E-06 | 0.000102897 | 3.889109397 |
| hsa_circ_0008113 | -1.635817582 | 7.458843356 | -8.793310849 | 6.40E-06 | 0.000107393 | 3.814632102 |
| hsa_circ_0007248 | -2.015965589 | 7.7648519 | -8.788628251 | 6.43E-06 | 0.000107393 | 3.809649162 |
| hsa_circ_0049613 | -1.214599135 | 8.110846095 | -8.775050731 | 6.52E-06 | 0.000107393 | 3.795188742 |
| hsa_circ_0055548 | -1.836607008 | 7.367372723 | -8.77322115 | 6.53E-06 | 0.000107393 | 3.79323882 |
| hsa_circ_0032446 | -1.777446643 | 7.822176028 | -8.772943197 | 6.53E-06 | 0.000107393 | 3.792942556 |
| hsa_circ_0003526 | -1.547297219 | 6.968441986 | -8.727462327 | 6.84E-06 | 0.000111622 | 3.744364065 |
| hsa_circ_0076794 | -1.454697604 | 7.579185298 | -8.705235022 | 6.99E-06 | 0.000113386 | 3.720549304 |
| hsa_circ_0072272 | -2.125424823 | 8.79649655 | -8.662426797 | 7.29E-06 | 0.000117579 | 3.674546897 |
| hsa_circ_0050176 | -1.410457953 | 7.506351676 | -8.540762229 | 8.24E-06 | 0.000127776 | 3.542811817 |
| hsa_circ_0002874 | -1.333305924 | 7.477285358 | -8.490932162 | 8.67E-06 | 0.000132978 | 3.48842907 |
| hsa_circ_0069152 | -2.096087925 | 8.686709253 | -8.436602489 | 9.16E-06 | 0.00013803 | 3.428849086 |
| hsa_circ_0008932 | -1.214749797 | 9.61340527 | -8.371953424 | 9.79E-06 | 0.000146122 | 3.357559902 |
| hsa_circ_0089169 | -2.300696319 | 8.113459149 | -8.3636884 | 9.87E-06 | 0.000146122 | 3.348415036 |
| hsa_circ_0004914 | -1.204590698 | 6.923611839 | -8.293654366 | 1.06E-05 | 0.000153662 | 3.270642189 |
| hsa_circ_0006840 | -1.504831582 | 7.168017337 | -8.292455237 | 1.06E-05 | 0.000153662 | 3.269306125 |
| hsa_circ_0000538 | -1.876803965 | 7.256951086 | -8.26549138 | 1.09E-05 | 0.000156208 | 3.239223518 |
| hsa_circ_0001739 | -1.803189575 | 8.536460218 | -8.260179675 | 1.10E-05 | 0.000156208 | 3.233288491 |
| hsa_circ_0059342 | -1.228858118 | 7.410639672 | -8.249943568 | 1.11E-05 | 0.000156254 | 3.221842864 |
| hsa_circ_0024139 | -2.59589571 | 8.41781199 | -8.249131643 | 1.11E-05 | 0.000156254 | 3.220934531 |
| hsa_circ_0002747 | -2.249190268 | 8.558213517 | -8.191872759 | 1.18E-05 | 0.000164032 | 3.156702109 |
| hsa_circ_0086740 | -1.229817289 | 7.414915504 | -8.151303879 | 1.23E-05 | 0.000170209 | 3.110983003 |
| hsa_circ_0001434 | -1.093581901 | 8.162400744 | -8.125052409 | 1.27E-05 | 0.000172416 | 3.081305835 |
| hsa_circ_0001897 | -1.241548305 | 9.778866171 | -8.121758376 | 1.27E-05 | 0.000172416 | 3.077576764 |
| hsa_circ_0009143 | -2.011920623 | 7.115678722 | -8.067769948 | 1.34E-05 | 0.000180724 | 3.01629293 |
| hsa_circ_0033126 | -1.564733134 | 9.975852205 | -8.063702109 | 1.35E-05 | 0.000180724 | 3.011662752 |
| hsa_circ_0001142 | -1.720750862 | 7.16887641 | -7.995784473 | 1.45E-05 | 0.000192185 | 2.934092649 |
| hsa_circ_0011426 | -1.851777514 | 7.555568033 | -7.935504801 | 1.55E-05 | 0.000199905 | 2.86482695 |
| hsa_circ_0002071 | -1.076974607 | 7.027398526 | -7.934828653 | 1.55E-05 | 0.000199905 | 2.864047759 |
| hsa_circ_0001238 | -1.2588632 | 7.715402256 | -7.930805812 | 1.55E-05 | 0.000199905 | 2.859410813 |
| hsa_circ_0007762 | -1.511822197 | 8.661011923 | -7.886162233 | 1.63E-05 | 0.000207401 | 2.807833048 |
| hsa_circ_0078363 | -2.069011191 | 7.9996586 | -7.84709626 | 1.70E-05 | 0.0002131 | 2.762519172 |
| hsa_circ_0007094 | -1.248954116 | 7.283965527 | -7.749926586 | 1.89E-05 | 0.000233308 | 2.649074089 |
| hsa_circ_0007511 | -1.429746569 | 7.727186326 | -7.673287897 | 2.05E-05 | 0.000250017 | 2.558852229 |
| hsa_circ_0003848 | -2.080099063 | 8.433502939 | -7.564729019 | 2.32E-05 | 0.000275231 | 2.429911548 |
| hsa_circ_0067029 | -1.173792054 | 9.957061611 | -7.529715528 | 2.41E-05 | 0.000284809 | 2.388036163 |
| hsa_circ_0059859 | -1.913595475 | 10.24026731 | -7.509070227 | 2.46E-05 | 0.000286087 | 2.363278478 |
| hsa_circ_0070562 | -1.004691116 | 6.994902876 | -7.480574001 | 2.54E-05 | 0.000292651 | 2.329024873 |
| hsa_circ_0056548 | -1.305595588 | 8.372764246 | -7.445581857 | 2.65E-05 | 0.000299853 | 2.286833696 |
| hsa_circ_0058698 | 1.052698582 | 8.333188913 | 7.442786277 | 2.65E-05 | 0.000299853 | 2.283456811 |
| hsa_circ_0000182 | -1.20191278 | 7.131397988 | -7.41985738 | 2.72E-05 | 0.000304958 | 2.255725647 |
| hsa_circ_0083234 | -1.468802931 | 7.175458084 | -7.419848588 | 2.72E-05 | 0.000304958 | 2.255715001 |
| hsa_circ_0006916 | -1.047171247 | 7.092415817 | -7.412327155 | 2.75E-05 | 0.000305577 | 2.246604845 |
| hsa_circ_0005587 | -1.330056496 | 7.945663385 | -7.410216674 | 2.75E-05 | 0.000305577 | 2.244047382 |
| hsa_circ_0000650 | -1.348523649 | 7.032201048 | -7.379108862 | 2.85E-05 | 0.000313733 | 2.206290491 |
| hsa_circ_0043302 | -1.010740552 | 7.666755171 | -7.348806852 | 2.95E-05 | 0.000323266 | 2.169401999 |
| hsa_circ_0005576 | -1.407432772 | 8.553525813 | -7.323321359 | 3.04E-05 | 0.000331312 | 2.138292865 |
| hsa_circ_0083172 | -1.006428982 | 7.385888206 | -7.168687314 | 3.62E-05 | 0.000383403 | 1.947875203 |
| hsa_circ_0055033 | -2.478054458 | 8.747651631 | -7.16609758 | 3.64E-05 | 0.000383403 | 1.944661699 |
| hsa_circ_0001641 | -1.056687426 | 7.082598081 | -7.113160044 | 3.87E-05 | 0.000402217 | 1.878795185 |
| hsa_circ_0006502 | -1.862128345 | 10.74462101 | -7.068125574 | 4.07E-05 | 0.000416973 | 1.822493163 |
| hsa_circ_0001178 | -1.003713124 | 7.297474387 | -7.02356301 | 4.29E-05 | 0.000434344 | 1.766536499 |
| hsa_circ_0066701 | -1.3135443 | 7.817882138 | -6.921454473 | 4.84E-05 | 0.000478278 | 1.637395408 |
| hsa_circ_0058565 | -1.742113524 | 8.672090791 | -6.906534667 | 4.92E-05 | 0.000484899 | 1.618417187 |
| hsa_circ_0006992 | -1.218044973 | 8.114352565 | -6.877784195 | 5.09E-05 | 0.000495921 | 1.581767683 |
| hsa_circ_0067301 | -2.97134691 | 10.44417338 | -6.845620251 | 5.29E-05 | 0.000511923 | 1.54064407 |
| hsa_circ_0005013 | -1.081239856 | 7.046666329 | -6.844634732 | 5.30E-05 | 0.000511923 | 1.539381971 |
| hsa_circ_0016760 | -1.332133951 | 7.50345718 | -6.797718805 | 5.60E-05 | 0.000539208 | 1.479157685 |
| hsa_circ_0008806 | -1.125706738 | 7.263194518 | -6.794782354 | 5.62E-05 | 0.000539208 | 1.475379025 |
| hsa_circ_0092304 | -1.953933921 | 7.263100161 | -6.69495636 | 6.34E-05 | 0.000599819 | 1.346270368 |
| hsa_circ_0003195 | -2.013194824 | 8.350332893 | -6.688717185 | 6.39E-05 | 0.000600401 | 1.338158821 |
| hsa_circ_0026983 | -1.374090994 | 8.517756896 | -6.633924702 | 6.83E-05 | 0.000633165 | 1.266708694 |
| hsa_circ_0078153 | -2.369360238 | 8.032794164 | -6.624081162 | 6.91E-05 | 0.000636138 | 1.25383168 |
| hsa_circ_0001359 | -1.580505005 | 11.75334234 | -6.580272098 | 7.29E-05 | 0.000666241 | 1.196370314 |
| hsa_circ_0061694 | -1.02952606 | 9.347089893 | -6.536142132 | 7.70E-05 | 0.000688377 | 1.138236711 |
| hsa_circ_0000042 | -1.157660817 | 7.427600388 | -6.525380856 | 7.80E-05 | 0.000692756 | 1.124022203 |
| hsa_circ_0064018 | -1.69309169 | 7.589973569 | -6.52525958 | 7.80E-05 | 0.000692756 | 1.123861925 |
| hsa_circ_0067323 | -1.243761682 | 7.460229235 | -6.50722285 | 7.98E-05 | 0.00070584 | 1.100003188 |
| hsa_circ_0008777 | -1.412309937 | 6.941636097 | -6.502270687 | 8.02E-05 | 0.000707688 | 1.09344511 |
| hsa_circ_0078279 | -1.622755092 | 7.123463538 | -6.434681104 | 8.72E-05 | 0.000759712 | 1.003616446 |
| hsa_circ_0077519 | -1.315214667 | 7.134400245 | -6.43120926 | 8.76E-05 | 0.000759712 | 0.998986076 |
| hsa_circ_0046131 | -1.30924384 | 7.009343044 | -6.419015415 | 8.90E-05 | 0.000765878 | 0.982710683 |
| hsa_circ_0007807 | -1.294004132 | 7.113982318 | -6.398935851 | 9.12E-05 | 0.000782605 | 0.955867384 |
| hsa_circ_0007311 | -1.00118663 | 7.577478844 | -6.314469115 | 0.00010139 | 0.000852548 | 0.842364756 |
| hsa_circ_0059703 | -1.036684704 | 7.587214439 | -6.274370809 | 0.000106643 | 0.000887874 | 0.788150896 |
| hsa_circ_0007348 | -1.307686047 | 6.892145179 | -6.226142469 | 0.000113355 | 0.000936705 | 0.722660807 |
| hsa_circ_0016599 | -1.045493463 | 7.335222951 | -6.209549183 | 0.000115769 | 0.000948254 | 0.700056514 |
| hsa_circ_0000478 | -1.130863052 | 7.753995138 | -6.187949385 | 0.000118994 | 0.000968406 | 0.670576725 |
| hsa_circ_0001360 | -1.318699762 | 12.2241123 | -6.129437277 | 0.00012823 | 0.001020598 | 0.590403028 |
| hsa_circ_0008542 | -1.276382454 | 7.268853914 | -6.126516237 | 0.000128711 | 0.001021215 | 0.5863885 |
| hsa_circ_0072665 | 1.028114126 | 7.438501843 | 6.060307541 | 0.000140151 | 0.001098215 | 0.495085039 |
| hsa_circ_0036516 | -1.65074608 | 7.034077671 | -5.996619782 | 0.000152199 | 0.001174435 | 0.406696522 |
| hsa_circ_0017726 | -1.644599478 | 8.362007489 | -5.986165822 | 0.000154281 | 0.001183286 | 0.392135299 |
| hsa_circ_0007582 | -1.258755972 | 7.587526291 | -5.972095721 | 0.000157132 | 0.001197893 | 0.37251364 |
| hsa_circ_0015379 | 1.060444836 | 8.692155515 | 5.820575978 | 0.000191706 | 0.001394277 | 0.159490984 |
| hsa_circ_0059074 | -1.350451652 | 7.947213707 | -5.774780404 | 0.000203708 | 0.001448273 | 0.094485107 |
| hsa_circ_0005360 | -1.079886996 | 7.144436923 | -5.732694825 | 0.000215454 | 0.001518979 | 0.034489992 |
| hsa_circ_0072387 | -1.076615662 | 8.925085295 | -5.715026486 | 0.000220601 | 0.001550944 | 0.00922983 |
| hsa_circ_0005944 | -1.114044357 | 9.066435771 | -5.681054003 | 0.000230873 | 0.001605096 | -0.039461683 |
| hsa_circ_0073518 | -1.130826458 | 7.547243428 | -5.505379263 | 0.000292882 | 0.00194563 | -0.293808769 |
| hsa_circ_0001393 | -1.053452386 | 7.229285959 | -5.500158857 | 0.00029498 | 0.001948813 | -0.301432779 |
| hsa_circ_0001330 | -1.29825628 | 7.649077832 | -5.475611982 | 0.00030506 | 0.001991405 | -0.337332588 |
| hsa_circ_0020048 | -2.395763527 | 11.25426963 | -5.464245432 | 0.000309853 | 0.002005726 | -0.353984639 |
| hsa_circ_0001072 | -1.064686562 | 7.515653547 | -5.400700449 | 0.000338183 | 0.002150604 | -0.447410468 |
| hsa_circ_0004831 | -1.299273251 | 8.21123271 | -5.355198678 | 0.000360172 | 0.002267652 | -0.51465486 |
| hsa_circ_0023642 | -1.045184344 | 7.399261007 | -5.340133875 | 0.000367787 | 0.002300532 | -0.536981922 |
| hsa_circ_0071775 | -1.246909306 | 7.914418104 | -5.339480045 | 0.000368121 | 0.002300532 | -0.537951659 |
| hsa_circ_0045888 | -1.15212316 | 9.86747021 | -5.311262574 | 0.000382868 | 0.002386794 | -0.579859662 |
| hsa_circ_0052867 | -1.199181327 | 8.596392307 | -5.240390966 | 0.000422775 | 0.00256874 | -0.685606489 |
| hsa_circ_0000204 | -1.414623174 | 10.28165322 | -5.17316417 | 0.000464774 | 0.002767868 | -0.78656172 |
| hsa_circ_0002236 | -1.743469999 | 7.239014945 | -5.145855316 | 0.000483094 | 0.0028568 | -0.827751309 |
| hsa_circ_0046215 | -1.259716892 | 8.043815793 | -5.143049163 | 0.00048502 | 0.002861503 | -0.831989664 |
| hsa_circ_0018909 | -1.399644061 | 11.30071176 | -5.138288048 | 0.000488306 | 0.002874192 | -0.839183256 |
| hsa_circ_0041392 | 1.031972405 | 9.565609576 | 5.131281297 | 0.000493186 | 0.002882136 | -0.849775513 |
| hsa_circ_0001251 | -1.137775246 | 9.710557447 | -5.038918496 | 0.000562631 | 0.003171534 | -0.990038509 |
| hsa_circ_0036763 | -1.136805212 | 7.148850186 | -5.034643978 | 0.000566088 | 0.003183933 | -0.996558408 |
| hsa_circ_0086686 | 1.041095945 | 8.613462723 | 4.821068816 | 0.000771404 | 0.004110364 | -1.325517888 |
| hsa_circ_0032253 | -1.093053625 | 8.31960868 | -4.802278101 | 0.000792945 | 0.004216268 | -1.354757884 |
| hsa_circ_0009581 | -1.553458108 | 10.8251981 | -4.768199002 | 0.000833667 | 0.004384999 | -1.40790951 |
| hsa_circ_0072389 | -1.018403558 | 10.29981321 | -4.69567628 | 0.000927938 | 0.004725576 | -1.521538529 |
| hsa_circ_0007540 | -1.104311308 | 10.87143806 | -4.683778217 | 0.00094446 | 0.004790437 | -1.540247464 |
| hsa_circ_0009577 | -1.180548622 | 7.756162782 | -4.618195022 | 0.001041333 | 0.00516787 | -1.64370887 |
| hsa_circ_0000724 | -1.29110004 | 7.195287765 | -4.601561095 | 0.001067549 | 0.005287605 | -1.670039816 |
| hsa_circ_0062239 | -1.319972064 | 9.48126766 | -4.569876319 | 0.001119448 | 0.005501599 | -1.720295688 |
| hsa_circ_0027774 | -1.333769767 | 9.849850601 | -4.447489053 | 0.001346547 | 0.00633478 | -1.915632345 |
| hsa_circ_0046419 | 1.130566465 | 7.244954402 | 4.438305831 | 0.001365457 | 0.006361106 | -1.93036615 |
| hsa_circ_0000172 | 1.04593687 | 8.939012675 | 4.371999578 | 0.001510639 | 0.00687667 | -2.037062625 |
| hsa_circ_0058850 | -1.308045982 | 7.201781408 | -4.328723011 | 0.001614162 | 0.007200672 | -2.106993978 |
| hsa_circ_0009009 | -1.509970448 | 7.471199195 | -4.309143255 | 0.001663444 | 0.007360451 | -2.138708246 |
| hsa_circ_0007146 | 1.559702804 | 9.264686058 | 4.239025465 | 0.001853414 | 0.008115903 | -2.252658744 |
| hsa_circ_0001654 | -1.424902405 | 9.692465592 | -4.153738502 | 0.002115895 | 0.008929297 | -2.392038554 |
| hsa_circ_0000517 | 1.02827997 | 8.176834764 | 4.127069458 | 0.002205817 | 0.009240648 | -2.435792961 |
| hsa_circ_0084501 | 1.024890575 | 8.037381461 | 4.124570608 | 0.002214446 | 0.009248785 | -2.439896775 |
