# Supplementary material for: Up-Regulation of hsa_circ_0000517 Predicts Adverse Prognosis of Hepatocellular Carcinoma
Source: Front Oncol. 2019 Oct 22;9:1105. doi: 10.3389/fonc.2019.01105 (PMC6842961; doi:10.3389/fonc.2019.01105)
Supplement: Supplementary file 3 [file Table_3.DOCX]

**Table S3 824 differentially expressed circRNAs in GSE97332.**

| ID | logFC | AveExpr | t | P.Value | adj.P.Val | B |
| --- | --- | --- | --- | --- | --- | --- |
| hsa_circ_0000518 | 1.896164643 | 7.687308556 | 33.27001824 | 1.26E-14 | 2.89E-11 | 23.432489 |
| hsa_circ_0046599 | 3.987858357 | 7.99322626 | 32.56822732 | 1.69E-14 | 2.89E-11 | 23.18565632 |
| hsa_circ_0008768 | 1.750291592 | 8.511031622 | 26.98552836 | 2.21E-13 | 2.21E-10 | 20.92779002 |
| hsa_circ_0000519 | 1.610866918 | 10.35214124 | 26.6714286 | 2.59E-13 | 2.21E-10 | 20.78296244 |
| hsa_circ_0046600 | 4.191870357 | 8.267620464 | 25.28591697 | 5.36E-13 | 3.66E-10 | 20.11771043 |
| hsa_circ_0063526 | 1.575501194 | 6.430913291 | 23.77686625 | 1.23E-12 | 7.03E-10 | 19.34034406 |
| hsa_circ_0069323 | 2.157598153 | 7.829563464 | 21.85048099 | 3.87E-12 | 1.89E-09 | 18.25806888 |
| hsa_circ_0040705 | 2.660155949 | 7.441211158 | 18.72308038 | 3.08E-11 | 1.32E-08 | 16.2458695 |
| hsa_circ_0069086 | 1.943068112 | 10.45145287 | 17.4341597 | 7.98E-11 | 2.69E-08 | 15.30689121 |
| hsa_circ_0042196 | -1.11275552 | 7.12201825 | -17.22851997 | 9.35E-11 | 2.69E-08 | 15.15030564 |
| hsa_circ_0000514 | 1.849176939 | 10.06148771 | 17.21416258 | 9.45E-11 | 2.69E-08 | 15.13930014 |
| hsa_circ_0091570 | -6.460436209 | 11.13340466 | -16.9210475 | 1.19E-10 | 3.12E-08 | 14.91249959 |
| hsa_circ_0041150 | 1.2775695 | 12.05983523 | 16.71396047 | 1.40E-10 | 3.39E-08 | 14.7497909 |
| hsa_circ_0084170 | -2.54845373 | 7.242317543 | -16.6358234 | 1.49E-10 | 3.39E-08 | 14.68785635 |
| hsa_circ_0072386 | 1.146152755 | 6.318207214 | 16.55052755 | 1.59E-10 | 3.40E-08 | 14.61990429 |
| hsa_circ_0046092 | 1.007552745 | 6.616505332 | 15.71286615 | 3.16E-10 | 6.35E-08 | 13.93289629 |
| hsa_circ_0006248 | 1.062706832 | 6.005118298 | 15.0549144 | 5.55E-10 | 9.81E-08 | 13.3668691 |
| hsa_circ_0033408 | 1.224746939 | 14.32287686 | 15.01473179 | 5.74E-10 | 9.81E-08 | 13.33150759 |
| hsa_circ_0064324 | 1.678169806 | 13.30697746 | 14.70329227 | 7.56E-10 | 1.23E-07 | 13.05422933 |
| hsa_circ_0007144 | -1.25559151 | 11.97516922 | -14.22598443 | 1.16E-09 | 1.63E-07 | 12.61793017 |
| hsa_circ_0031132 | 1.649744327 | 12.33003529 | 14.19930659 | 1.19E-09 | 1.63E-07 | 12.59312732 |
| hsa_circ_0045006 | 1.856554653 | 12.19880073 | 14.06225884 | 1.35E-09 | 1.78E-07 | 12.4649995 |
| hsa_circ_0007099 | 1.075123908 | 6.288516832 | 13.80806684 | 1.71E-09 | 2.09E-07 | 12.22414744 |
| hsa_circ_0060063 | 2.274256449 | 9.035347602 | 13.70638414 | 1.89E-09 | 2.22E-07 | 12.126614 |
| hsa_circ_0040827 | 1.229734235 | 10.71990222 | 13.06393008 | 3.51E-09 | 3.87E-07 | 11.49410708 |
| hsa_circ_0029636 | 1.820097 | 12.14269927 | 12.39412154 | 6.90E-09 | 6.54E-07 | 10.80313481 |
| hsa_circ_0054970 | 2.874732724 | 10.62504575 | 12.34434537 | 7.26E-09 | 6.69E-07 | 10.75043367 |
| hsa_circ_0092370 | -1.264049495 | 6.472237018 | -12.24924654 | 8.01E-09 | 6.86E-07 | 10.64921019 |
| hsa_circ_0001851 | 1.111827495 | 6.294594462 | 12.21543577 | 8.30E-09 | 6.87E-07 | 10.61305105 |
| hsa_circ_0006900 | 1.695992867 | 9.059647301 | 12.1983677 | 8.45E-09 | 6.87E-07 | 10.59476327 |
| hsa_circ_0052767 | -1.587366582 | 8.367043219 | -11.74603646 | 1.37E-08 | 1.06E-06 | 10.10157389 |
| hsa_circ_0050834 | 1.878547561 | 8.018035036 | 11.65885313 | 1.50E-08 | 1.12E-06 | 10.00458396 |
| hsa_circ_0000520 | 3.118940867 | 12.00587852 | 11.65819245 | 1.50E-08 | 1.12E-06 | 10.00384653 |
| hsa_circ_0058794 | -1.148509857 | 9.139039541 | -11.63205818 | 1.55E-08 | 1.12E-06 | 9.974646863 |
| hsa_circ_0039557 | 1.627211816 | 12.27126151 | 11.54972119 | 1.69E-08 | 1.16E-06 | 9.882274527 |
| hsa_circ_0041829 | 1.773497245 | 11.77757808 | 11.51296102 | 1.76E-08 | 1.18E-06 | 9.840847787 |
| hsa_circ_0008583 | 1.561726071 | 13.13383896 | 11.31051806 | 2.20E-08 | 1.45E-06 | 9.610619962 |
| hsa_circ_0092372 | -1.382598495 | 10.07331795 | -11.23171556 | 2.41E-08 | 1.52E-06 | 9.520035644 |
| hsa_circ_0048122 | 1.101348857 | 9.557814153 | 11.18191622 | 2.54E-08 | 1.58E-06 | 9.462507923 |
| hsa_circ_0092273 | 2.161096898 | 12.09064088 | 11.07214129 | 2.88E-08 | 1.72E-06 | 9.334916075 |
| hsa_circ_0051220 | 2.958364214 | 9.969716668 | 11.06867704 | 2.89E-08 | 1.72E-06 | 9.330871954 |
| hsa_circ_0000613 | -1.31695199 | 7.165492301 | -11.06052564 | 2.92E-08 | 1.72E-06 | 9.321351871 |
| hsa_circ_0004837 | 1.004672219 | 5.977294931 | 10.99336236 | 3.15E-08 | 1.82E-06 | 9.242682628 |
| hsa_circ_0065214 | 2.042702929 | 13.32874162 | 10.94014238 | 3.35E-08 | 1.90E-06 | 9.180054227 |
| hsa_circ_0052166 | 1.375336949 | 11.19623833 | 10.80587508 | 3.90E-08 | 2.15E-06 | 9.020895269 |
| hsa_circ_0000453 | 1.131578786 | 13.20892827 | 10.54542867 | 5.29E-08 | 2.82E-06 | 8.7073634 |
| hsa_circ_0006879 | -1.458160173 | 7.622417464 | -10.48454662 | 5.68E-08 | 2.99E-06 | 8.633141942 |
| hsa_circ_0008563 | 1.296137173 | 13.67365369 | 10.46709008 | 5.80E-08 | 3.00E-06 | 8.611794757 |
| hsa_circ_0000708 | -2.39344898 | 12.52776105 | -10.45193423 | 5.91E-08 | 3.01E-06 | 8.593237134 |
| hsa_circ_0044556 | 1.461621051 | 11.53382217 | 10.36859514 | 6.52E-08 | 3.28E-06 | 8.490793637 |
| hsa_circ_0072088 | 4.556802643 | 12.83533646 | 10.30626604 | 7.03E-08 | 3.48E-06 | 8.413732634 |
| hsa_circ_0008153 | -1.24027648 | 7.083219434 | -10.26615317 | 7.38E-08 | 3.53E-06 | 8.363936536 |
| hsa_circ_0000137 | 2.419295684 | 8.421978434 | 10.26453228 | 7.39E-08 | 3.53E-06 | 8.361921021 |
| hsa_circ_0000517 | 1.722260888 | 9.660127311 | 10.25803356 | 7.45E-08 | 3.53E-06 | 8.353837487 |
| hsa_circ_0052001 | -1.382681316 | 10.12038494 | -10.21630611 | 7.83E-08 | 3.61E-06 | 8.301834347 |
| hsa_circ_0001827 | 1.065631643 | 12.10247614 | 10.20720633 | 7.92E-08 | 3.61E-06 | 8.290470677 |
| hsa_circ_0088059 | -1.659232286 | 8.668887633 | -10.09929739 | 9.03E-08 | 3.86E-06 | 8.155083868 |
| hsa_circ_0000871 | 1.05664899 | 11.88812964 | 9.916727809 | 1.13E-07 | 4.50E-06 | 7.923340448 |
| hsa_circ_0003285 | -2.353153918 | 12.0961348 | -9.914678651 | 1.13E-07 | 4.50E-06 | 7.920719997 |
| hsa_circ_0081778 | -1.658004281 | 6.983373916 | -9.864487564 | 1.21E-07 | 4.73E-06 | 7.856400486 |
| hsa_circ_0088222 | 1.413759209 | 6.419159814 | 9.842278399 | 1.24E-07 | 4.76E-06 | 7.827856305 |
| hsa_circ_0049271 | -1.861487918 | 13.29588721 | -9.751569551 | 1.39E-07 | 5.10E-06 | 7.710739415 |
| hsa_circ_0006461 | 1.392741449 | 9.197886531 | 9.75144666 | 1.39E-07 | 5.10E-06 | 7.710580163 |
| hsa_circ_0029596 | -1.674588837 | 8.849651888 | -9.716598783 | 1.45E-07 | 5.22E-06 | 7.6653572 |
| hsa_circ_0002846 | 1.231181918 | 9.260177827 | 9.715587677 | 1.45E-07 | 5.22E-06 | 7.664043153 |
| hsa_circ_0007874 | -2.709516372 | 12.49524123 | -9.707689327 | 1.47E-07 | 5.22E-06 | 7.653774633 |
| hsa_circ_0050532 | -1.368243276 | 7.139857699 | -9.645697321 | 1.58E-07 | 5.49E-06 | 7.572950562 |
| hsa_circ_0002599 | 1.241837735 | 7.296818439 | 9.642387093 | 1.59E-07 | 5.49E-06 | 7.568623277 |
| hsa_circ_0008537 | 1.912444872 | 8.406032084 | 9.558353397 | 1.77E-07 | 5.83E-06 | 7.458378829 |
| hsa_circ_0000204 | 1.249984663 | 10.39384107 | 9.545512715 | 1.80E-07 | 5.83E-06 | 7.441466411 |
| hsa_circ_0045194 | -1.71192899 | 7.046033372 | -9.537058018 | 1.82E-07 | 5.83E-06 | 7.430321077 |
| hsa_circ_0049888 | -2.185282969 | 11.98036233 | -9.533394254 | 1.83E-07 | 5.83E-06 | 7.42548896 |
| hsa_circ_0082917 | 1.668164224 | 6.560420653 | 9.483207085 | 1.95E-07 | 6.16E-06 | 7.359151584 |
| hsa_circ_0014130 | 2.448814959 | 8.272108122 | 9.433232067 | 2.08E-07 | 6.50E-06 | 7.292823496 |
| hsa_circ_0060055 | 1.860452122 | 12.19387756 | 9.427363542 | 2.09E-07 | 6.50E-06 | 7.285016822 |
| hsa_circ_0005397 | 3.707446398 | 13.00162036 | 9.413046534 | 2.13E-07 | 6.53E-06 | 7.265955695 |
| hsa_circ_0015796 | -2.099355306 | 12.77200553 | -9.320186184 | 2.40E-07 | 7.14E-06 | 7.141779945 |
| hsa_circ_0083766 | 2.896339459 | 10.21069568 | 9.288702835 | 2.50E-07 | 7.32E-06 | 7.099463958 |
| hsa_circ_0000715 | -1.057835265 | 9.012452643 | -9.287845512 | 2.51E-07 | 7.32E-06 | 7.098310117 |
| hsa_circ_0041992 | 1.274106816 | 13.48935708 | 9.264022312 | 2.58E-07 | 7.48E-06 | 7.066214761 |
| hsa_circ_0030130 | -2.318251408 | 10.1411588 | -9.216741327 | 2.75E-07 | 7.80E-06 | 7.002329552 |
| hsa_circ_0009150 | 1.48512752 | 10.39553161 | 9.213355891 | 2.76E-07 | 7.80E-06 | 6.997745657 |
| hsa_circ_0046123 | 1.86907302 | 12.76429284 | 9.204577858 | 2.79E-07 | 7.82E-06 | 6.985854198 |
| hsa_circ_0040534 | -1.427532541 | 9.738431852 | -9.156190029 | 2.98E-07 | 8.14E-06 | 6.920149187 |
| hsa_circ_0090518 | 1.341842413 | 8.736839895 | 9.136267701 | 3.05E-07 | 8.19E-06 | 6.893020621 |
| hsa_circ_0000217 | -1.99309951 | 12.03811839 | -9.125515145 | 3.10E-07 | 8.19E-06 | 6.878360123 |
| hsa_circ_0011501 | -1.444518959 | 9.032230694 | -9.120237513 | 3.12E-07 | 8.19E-06 | 6.871159603 |
| hsa_circ_0043001 | 1.431953602 | 10.02840132 | 9.119808905 | 3.12E-07 | 8.19E-06 | 6.870574695 |
| hsa_circ_0004928 | -1.412365622 | 10.31112475 | -9.110189342 | 3.16E-07 | 8.19E-06 | 6.857441728 |
| hsa_circ_0070113 | -2.267436796 | 12.52980284 | -9.07097017 | 3.33E-07 | 8.55E-06 | 6.803790059 |
| hsa_circ_0078738 | 1.064630612 | 14.49691462 | 9.019485178 | 3.56E-07 | 9.09E-06 | 6.733093862 |
| hsa_circ_0009101 | 1.854448286 | 10.68986564 | 8.98871587 | 3.71E-07 | 9.40E-06 | 6.690699017 |
| hsa_circ_0001443 | -1.234593337 | 8.93598874 | -8.97263958 | 3.79E-07 | 9.53E-06 | 6.668505572 |
| hsa_circ_0044922 | 1.254651602 | 6.735484454 | 8.950040768 | 3.91E-07 | 9.68E-06 | 6.637257624 |
| hsa_circ_0000116 | 1.478989189 | 5.855751666 | 8.941514451 | 3.95E-07 | 9.72E-06 | 6.625452831 |
| hsa_circ_0042986 | -1.045175745 | 9.496777985 | -8.933480514 | 4.00E-07 | 9.75E-06 | 6.614322092 |
| hsa_circ_0092368 | -1.950454357 | 9.525808413 | -8.89670174 | 4.20E-07 | 1.01E-05 | 6.563271423 |
| hsa_circ_0087897 | -1.480449398 | 9.235124658 | -8.895966432 | 4.20E-07 | 1.01E-05 | 6.562249189 |
| hsa_circ_0023179 | 1.115475316 | 11.42713677 | 8.888841081 | 4.24E-07 | 1.01E-05 | 6.552340202 |
| hsa_circ_0077929 | 1.002475245 | 7.407716291 | 8.853846912 | 4.45E-07 | 1.05E-05 | 6.503589653 |
| hsa_circ_0005455 | 1.32271648 | 8.713347005 | 8.83278275 | 4.58E-07 | 1.07E-05 | 6.474176549 |
| hsa_circ_0017348 | -1.0224855 | 7.347466056 | -8.811761954 | 4.71E-07 | 1.09E-05 | 6.444772528 |
| hsa_circ_0002980 | -5.75581426 | 8.821418156 | -8.77351654 | 4.96E-07 | 1.12E-05 | 6.391142347 |
| hsa_circ_0040573 | -1.134780776 | 9.347451061 | -8.769337886 | 4.98E-07 | 1.12E-05 | 6.385272403 |
| hsa_circ_0056856 | -1.807345694 | 12.05619421 | -8.740602011 | 5.18E-07 | 1.15E-05 | 6.344850368 |
| hsa_circ_0046580 | 1.022797699 | 6.278394753 | 8.716665423 | 5.35E-07 | 1.18E-05 | 6.311105306 |
| hsa_circ_0024604 | 1.896897602 | 11.61629858 | 8.712025452 | 5.39E-07 | 1.18E-05 | 6.304556214 |
| hsa_circ_0005188 | 2.651862735 | 9.222729184 | 8.705119329 | 5.44E-07 | 1.18E-05 | 6.294803859 |
| hsa_circ_0003838 | 1.095948582 | 9.047047056 | 8.679985814 | 5.63E-07 | 1.21E-05 | 6.259264513 |
| hsa_circ_0004720 | 1.702671612 | 10.74103841 | 8.657712541 | 5.80E-07 | 1.24E-05 | 6.227707185 |
| hsa_circ_0000877 | 1.417812602 | 12.54111904 | 8.633165172 | 6.00E-07 | 1.26E-05 | 6.192859744 |
| hsa_circ_0072627 | -1.067266816 | 7.579149031 | -8.589920289 | 6.37E-07 | 1.31E-05 | 6.131295022 |
| hsa_circ_0049785 | 1.194334541 | 10.46761161 | 8.579465749 | 6.46E-07 | 1.32E-05 | 6.116378156 |
| hsa_circ_0000516 | 1.805930847 | 11.51801924 | 8.550477045 | 6.72E-07 | 1.36E-05 | 6.074947808 |
| hsa_circ_0055161 | 1.513399867 | 6.935068464 | 8.547640445 | 6.75E-07 | 1.36E-05 | 6.070888365 |
| hsa_circ_0000098 | -5.327681367 | 8.298021286 | -8.541145351 | 6.81E-07 | 1.37E-05 | 6.061589638 |
| hsa_circ_0007934 | -1.901687944 | 9.908893895 | -8.495952964 | 7.25E-07 | 1.44E-05 | 5.99674949 |
| hsa_circ_0004726 | -1.92628949 | 8.421410194 | -8.474882707 | 7.46E-07 | 1.46E-05 | 5.966434712 |
| hsa_circ_0001033 | -2.25376102 | 11.1728608 | -8.39657181 | 8.32E-07 | 1.58E-05 | 5.853294281 |
| hsa_circ_0026652 | 2.432758561 | 11.36868698 | 8.388731209 | 8.41E-07 | 1.58E-05 | 5.841925485 |
| hsa_circ_0076798 | -1.628799061 | 11.77562469 | -8.382547655 | 8.49E-07 | 1.58E-05 | 5.83295411 |
| hsa_circ_0005835 | 1.617169194 | 8.607040301 | 8.37779867 | 8.54E-07 | 1.58E-05 | 5.82606091 |
| hsa_circ_0078610 | -1.117743974 | 8.516880339 | -8.364983316 | 8.70E-07 | 1.60E-05 | 5.80744556 |
| hsa_circ_0001282 | -1.103110071 | 8.867586801 | -8.343961934 | 8.96E-07 | 1.62E-05 | 5.776866853 |
| hsa_circ_0005916 | -1.532930704 | 7.85097827 | -8.323759423 | 9.22E-07 | 1.66E-05 | 5.747428371 |
| hsa_circ_0061817 | -2.057075367 | 13.93726661 | -8.31270313 | 9.36E-07 | 1.67E-05 | 5.731296302 |
| hsa_circ_0023685 | -1.600079082 | 9.931092469 | -8.311724818 | 9.37E-07 | 1.67E-05 | 5.729868139 |
| hsa_circ_0007767 | -1.556168184 | 12.32709235 | -8.301846904 | 9.51E-07 | 1.67E-05 | 5.715441551 |
| hsa_circ_0005711 | -1.701789031 | 9.654562699 | -8.25472998 | 1.02E-06 | 1.74E-05 | 5.646462637 |
| hsa_circ_0069559 | -1.179770133 | 10.65968735 | -8.212017368 | 1.08E-06 | 1.82E-05 | 5.583694897 |
| hsa_circ_0058051 | -1.342587337 | 9.519156046 | -8.18987432 | 1.11E-06 | 1.86E-05 | 5.551065964 |
| hsa_circ_0006078 | -1.34253099 | 11.96303535 | -8.18485869 | 1.12E-06 | 1.86E-05 | 5.54366672 |
| hsa_circ_0002881 | -1.148949531 | 9.696907908 | -8.179589764 | 1.13E-06 | 1.86E-05 | 5.535890438 |
| hsa_circ_0001288 | -2.44787798 | 10.76421846 | -8.158867609 | 1.16E-06 | 1.90E-05 | 5.505273616 |
| hsa_circ_0003763 | 3.312282235 | 10.95405448 | 8.156663118 | 1.17E-06 | 1.90E-05 | 5.502013352 |
| hsa_circ_0011385 | 3.634586918 | 10.50327461 | 8.139785072 | 1.20E-06 | 1.94E-05 | 5.477032022 |
| hsa_circ_0060937 | -1.012297 | 8.724242276 | -8.127693944 | 1.22E-06 | 1.95E-05 | 5.459114002 |
| hsa_circ_0003473 | 1.21668151 | 11.05324502 | 8.123575645 | 1.22E-06 | 1.95E-05 | 5.453006866 |
| hsa_circ_0092321 | 2.311202561 | 11.02576758 | 8.089555812 | 1.28E-06 | 2.04E-05 | 5.402476815 |
| hsa_circ_0000919 | -1.537242265 | 13.60140382 | -8.047915013 | 1.36E-06 | 2.16E-05 | 5.340429761 |
| hsa_circ_0088072 | -1.086086638 | 9.017183656 | -8.038118296 | 1.38E-06 | 2.18E-05 | 5.32580045 |
| hsa_circ_0001887 | -1.600746224 | 12.78501028 | -8.034326216 | 1.39E-06 | 2.18E-05 | 5.320134542 |
| hsa_circ_0007518 | -1.822639561 | 12.30577963 | -8.025427003 | 1.41E-06 | 2.20E-05 | 5.306830734 |
| hsa_circ_0003945 | 2.421780847 | 12.18707122 | 8.00146648 | 1.46E-06 | 2.26E-05 | 5.270961484 |
| hsa_circ_0040481 | 1.273469898 | 8.877196408 | 7.979688766 | 1.51E-06 | 2.33E-05 | 5.238297011 |
| hsa_circ_0006302 | -4.452590566 | 7.928878074 | -7.968229617 | 1.53E-06 | 2.35E-05 | 5.221085289 |
| hsa_circ_0083377 | 1.183245332 | 8.017471615 | 7.925838998 | 1.63E-06 | 2.48E-05 | 5.157269519 |
| hsa_circ_0049547 | 2.482780418 | 8.842198801 | 7.917078519 | 1.65E-06 | 2.49E-05 | 5.14405283 |
| hsa_circ_0006665 | -1.893920867 | 9.087926066 | -7.912081004 | 1.66E-06 | 2.50E-05 | 5.136508844 |
| hsa_circ_0008365 | -1.419277551 | 11.70537802 | -7.903874115 | 1.68E-06 | 2.52E-05 | 5.124113254 |
| hsa_circ_0009006 | 1.359975327 | 12.97198363 | 7.900769657 | 1.69E-06 | 2.52E-05 | 5.119422079 |
| hsa_circ_0009020 | -1.195896755 | 8.478266704 | -7.89248409 | 1.71E-06 | 2.53E-05 | 5.106895668 |
| hsa_circ_0008459 | -1.344425959 | 9.100198276 | -7.890297552 | 1.71E-06 | 2.53E-05 | 5.103588522 |
| hsa_circ_0015453 | -1.099260235 | 12.30092803 | -7.878723299 | 1.74E-06 | 2.57E-05 | 5.086072268 |
| hsa_circ_0000189 | -1.902243378 | 10.85156212 | -7.834851306 | 1.86E-06 | 2.71E-05 | 5.019521738 |
| hsa_circ_0007387 | -1.093642816 | 8.685821408 | -7.818821625 | 1.90E-06 | 2.75E-05 | 4.995144413 |
| hsa_circ_0035554 | -1.692478296 | 10.96304125 | -7.761471958 | 2.07E-06 | 2.96E-05 | 4.907658809 |
| hsa_circ_0049356 | -2.028279582 | 12.99767869 | -7.746355047 | 2.12E-06 | 3.01E-05 | 4.884527739 |
| hsa_circ_0021087 | 1.651435735 | 10.63017665 | 7.729532237 | 2.17E-06 | 3.05E-05 | 4.858751698 |
| hsa_circ_0000515 | 1.366084031 | 10.09744942 | 7.729066916 | 2.17E-06 | 3.05E-05 | 4.85803821 |
| hsa_circ_0059151 | -1.699521173 | 10.19314261 | -7.713363578 | 2.22E-06 | 3.11E-05 | 4.833943461 |
| hsa_circ_0019083 | -1.775229786 | 9.055725434 | -7.694363599 | 2.29E-06 | 3.17E-05 | 4.804747747 |
| hsa_circ_0030793 | 2.756470112 | 10.62251487 | 7.690240655 | 2.30E-06 | 3.18E-05 | 4.798406176 |
| hsa_circ_0004808 | -1.101023265 | 8.047617755 | -7.687083925 | 2.31E-06 | 3.18E-05 | 4.793549265 |
| hsa_circ_0088300 | -2.307094939 | 13.1843122 | -7.678141582 | 2.34E-06 | 3.21E-05 | 4.779783651 |
| hsa_circ_0000979 | -1.67827198 | 11.95555474 | -7.667424217 | 2.38E-06 | 3.22E-05 | 4.763271932 |
| hsa_circ_0079480 | -1.840821031 | 12.94778404 | -7.659725808 | 2.41E-06 | 3.22E-05 | 4.751402159 |
| hsa_circ_0079449 | -1.972028082 | 10.99781959 | -7.651620969 | 2.43E-06 | 3.25E-05 | 4.738897404 |
| hsa_circ_0009018 | -1.834206929 | 11.13668468 | -7.624012895 | 2.54E-06 | 3.37E-05 | 4.696237407 |
| hsa_circ_0058055 | -1.332808765 | 9.629090607 | -7.621811935 | 2.55E-06 | 3.37E-05 | 4.692832208 |
| hsa_circ_0048607 | 2.291122 | 10.40215256 | 7.615244587 | 2.57E-06 | 3.39E-05 | 4.682667829 |
| hsa_circ_0036941 | 1.155398796 | 7.900672582 | 7.609182578 | 2.59E-06 | 3.41E-05 | 4.673280576 |
| hsa_circ_0030724 | -1.198807469 | 10.05742032 | -7.592918744 | 2.66E-06 | 3.48E-05 | 4.648071694 |
| hsa_circ_0078362 | -1.611075102 | 9.158729913 | -7.571447879 | 2.74E-06 | 3.57E-05 | 4.614738974 |
| hsa_circ_0008616 | 2.212529 | 11.85494527 | 7.56784247 | 2.76E-06 | 3.57E-05 | 4.609135795 |
| hsa_circ_0067013 | -1.5961275 | 10.22518395 | -7.564426418 | 2.77E-06 | 3.57E-05 | 4.603825325 |
| hsa_circ_0087354 | -1.99312101 | 10.01741842 | -7.549041622 | 2.84E-06 | 3.63E-05 | 4.579889729 |
| hsa_circ_0002811 | -4.429224628 | 7.798868503 | -7.548780223 | 2.84E-06 | 3.63E-05 | 4.579482777 |
| hsa_circ_0005245 | -1.641962469 | 10.11896021 | -7.537051439 | 2.89E-06 | 3.68E-05 | 4.561213934 |
| hsa_circ_0084625 | -2.31725349 | 11.79389363 | -7.534278698 | 2.90E-06 | 3.68E-05 | 4.556892453 |
| hsa_circ_0006276 | 1.109584071 | 10.69457511 | 7.508200894 | 3.02E-06 | 3.79E-05 | 4.516199232 |
| hsa_circ_0038728 | 1.1007735 | 6.810363301 | 7.503602609 | 3.04E-06 | 3.80E-05 | 4.509014546 |
| hsa_circ_0047958 | -2.092741388 | 12.75905299 | -7.47794253 | 3.16E-06 | 3.91E-05 | 4.468870307 |
| hsa_circ_0001191 | -1.95518299 | 10.71133004 | -7.471618002 | 3.19E-06 | 3.92E-05 | 4.458962491 |
| hsa_circ_0000219 | -2.050461224 | 9.132928245 | -7.469146927 | 3.20E-06 | 3.92E-05 | 4.455089945 |
| hsa_circ_0000220 | -2.174042653 | 9.206025786 | -7.445373768 | 3.32E-06 | 4.05E-05 | 4.417792663 |
| hsa_circ_0036750 | -1.074958449 | 8.390855888 | -7.420099445 | 3.45E-06 | 4.17E-05 | 4.378058325 |
| hsa_circ_0028196 | 1.344133469 | 13.40888031 | 7.418457063 | 3.46E-06 | 4.17E-05 | 4.375473374 |
| hsa_circ_0008533 | -1.134546255 | 8.447968148 | -7.40141543 | 3.55E-06 | 4.26E-05 | 4.34863041 |
| hsa_circ_0001605 | -1.688552959 | 12.93612132 | -7.395966755 | 3.57E-06 | 4.27E-05 | 4.340039863 |
| hsa_circ_0027364 | -3.866436633 | 7.531833429 | -7.392067954 | 3.60E-06 | 4.28E-05 | 4.333890479 |
| hsa_circ_0000235 | 1.269115469 | 10.96208603 | 7.384613523 | 3.64E-06 | 4.31E-05 | 4.322127365 |
| hsa_circ_0007009 | 1.173529148 | 6.347714503 | 7.383321438 | 3.64E-06 | 4.31E-05 | 4.320087701 |
| hsa_circ_0001535 | 2.15654148 | 13.12882882 | 7.367156195 | 3.74E-06 | 4.37E-05 | 4.294550789 |
| hsa_circ_0004069 | -1.858329439 | 10.5697397 | -7.345131297 | 3.86E-06 | 4.45E-05 | 4.259701256 |
| hsa_circ_0021773 | 1.302823515 | 6.621637906 | 7.342887033 | 3.88E-06 | 4.45E-05 | 4.256146584 |
| hsa_circ_0007615 | 1.277921755 | 6.199239531 | 7.341585468 | 3.88E-06 | 4.45E-05 | 4.254084737 |
| hsa_circ_0081188 | -2.109731398 | 11.81315331 | -7.334965011 | 3.92E-06 | 4.47E-05 | 4.243593592 |
| hsa_circ_0060035 | 1.339127204 | 9.827314898 | 7.324492628 | 3.99E-06 | 4.51E-05 | 4.226986555 |
| hsa_circ_0018293 | -1.143959582 | 6.628078923 | -7.318704609 | 4.02E-06 | 4.52E-05 | 4.217801685 |
| hsa_circ_0047585 | -1.184802816 | 13.31850358 | -7.312286883 | 4.06E-06 | 4.55E-05 | 4.207612327 |
| hsa_circ_0015358 | -1.232112112 | 10.9291354 | -7.293146162 | 4.18E-06 | 4.65E-05 | 4.177190181 |
| hsa_circ_0001167 | -1.759214694 | 12.96767217 | -7.276386401 | 4.29E-06 | 4.74E-05 | 4.150512162 |
| hsa_circ_0068514 | 1.804444827 | 8.128165974 | 7.264872144 | 4.37E-06 | 4.78E-05 | 4.132162137 |
| hsa_circ_0092350 | 1.215881408 | 11.17067515 | 7.263231414 | 4.38E-06 | 4.78E-05 | 4.129545897 |
| hsa_circ_0000301 | -2.478178367 | 12.49393397 | -7.23848001 | 4.55E-06 | 4.93E-05 | 4.090034649 |
| hsa_circ_0079534 | -1.889368107 | 11.09958774 | -7.224250223 | 4.65E-06 | 5.03E-05 | 4.067282167 |
| hsa_circ_0000305 | -2.175647255 | 12.92546294 | -7.218109282 | 4.69E-06 | 5.05E-05 | 4.057454829 |
| hsa_circ_0026337 | -1.565743571 | 13.36259829 | -7.217170888 | 4.70E-06 | 5.05E-05 | 4.055952673 |
| hsa_circ_0000512 | 1.603441153 | 12.18493108 | 7.212157722 | 4.74E-06 | 5.07E-05 | 4.047925732 |
| hsa_circ_0008801 | -2.254152837 | 12.42687578 | -7.210032122 | 4.75E-06 | 5.07E-05 | 4.044521263 |
| hsa_circ_0030045 | -1.623663694 | 10.63585082 | -7.19311926 | 4.88E-06 | 5.18E-05 | 4.017411157 |
| hsa_circ_0002404 | -1.965793082 | 12.53755392 | -7.192574301 | 4.88E-06 | 5.18E-05 | 4.016536989 |
| hsa_circ_0062162 | 2.212779847 | 6.98202277 | 7.190084009 | 4.90E-06 | 5.18E-05 | 4.012541805 |
| hsa_circ_0005039 | -1.3201735 | 7.737904403 | -7.16653766 | 5.08E-06 | 5.34E-05 | 3.974725116 |
| hsa_circ_0001644 | -1.919693531 | 13.67057678 | -7.13830068 | 5.31E-06 | 5.51E-05 | 3.929276691 |
| hsa_circ_0000953 | 1.878987515 | 7.212210487 | 7.137789804 | 5.31E-06 | 5.51E-05 | 3.92845343 |
| hsa_circ_0008310 | 1.309676112 | 11.99276357 | 7.131223599 | 5.37E-06 | 5.51E-05 | 3.917869051 |
| hsa_circ_0001917 | 1.141463867 | 12.79016961 | 7.118651462 | 5.47E-06 | 5.57E-05 | 3.897587214 |
| hsa_circ_0004639 | -1.942921531 | 11.1749156 | -7.112570088 | 5.53E-06 | 5.60E-05 | 3.887768868 |
| hsa_circ_0015278 | -1.915940306 | 14.34314716 | -7.101961051 | 5.62E-06 | 5.66E-05 | 3.870628692 |
| hsa_circ_0000844 | -1.538403837 | 8.83386349 | -7.092195785 | 5.70E-06 | 5.70E-05 | 3.854838304 |
| hsa_circ_0063756 | -1.122283306 | 7.405069398 | -7.091916298 | 5.71E-06 | 5.70E-05 | 3.854386185 |
| hsa_circ_0088045 | 1.891234949 | 11.34565261 | 7.081768998 | 5.80E-06 | 5.76E-05 | 3.837964024 |
| hsa_circ_0061749 | -1.851183194 | 13.78470963 | -7.073286471 | 5.88E-06 | 5.82E-05 | 3.82422542 |
| hsa_circ_0023984 | -4.204559408 | 7.595066082 | -7.068314323 | 5.92E-06 | 5.84E-05 | 3.81616783 |
| hsa_circ_0023704 | -1.698416867 | 9.812394036 | -7.065626081 | 5.95E-06 | 5.84E-05 | 3.81181002 |
| hsa_circ_0004780 | 2.343839265 | 8.003887878 | 7.062131771 | 5.98E-06 | 5.85E-05 | 3.806144064 |
| hsa_circ_0073805 | -1.320830148 | 8.598531023 | -7.057880247 | 6.02E-06 | 5.87E-05 | 3.799248072 |
| hsa_circ_0006857 | -1.656401714 | 13.84089008 | -7.051307509 | 6.08E-06 | 5.92E-05 | 3.788582249 |
| hsa_circ_0001712 | -1.652129643 | 10.54690582 | -7.042664631 | 6.16E-06 | 5.97E-05 | 3.774548236 |
| hsa_circ_0020390 | -1.991675582 | 12.86540933 | -7.042270891 | 6.17E-06 | 5.97E-05 | 3.773908654 |
| hsa_circ_0011536 | -1.960746112 | 10.5027271 | -7.036088982 | 6.23E-06 | 6.01E-05 | 3.763864147 |
| hsa_circ_0000747 | 1.253737204 | 12.01309754 | 7.029258509 | 6.29E-06 | 6.04E-05 | 3.752759822 |
| hsa_circ_0000876 | 1.144613969 | 7.332569281 | 7.019543078 | 6.39E-06 | 6.10E-05 | 3.73695452 |
| hsa_circ_0000978 | -1.701932071 | 9.472643168 | -7.014127788 | 6.45E-06 | 6.13E-05 | 3.728139243 |
| hsa_circ_0000673 | 2.263811378 | 13.83884527 | 7.003787288 | 6.55E-06 | 6.21E-05 | 3.71129542 |
| hsa_circ_0029426 | -1.819642367 | 14.98478466 | -7.002112836 | 6.57E-06 | 6.21E-05 | 3.708566513 |
| hsa_circ_0005139 | -1.74839898 | 12.52235779 | -6.994862832 | 6.64E-06 | 6.21E-05 | 3.69674656 |
| hsa_circ_0007291 | -1.921047949 | 10.42095801 | -6.99333412 | 6.66E-06 | 6.21E-05 | 3.694253333 |
| hsa_circ_0084443 | 1.691418801 | 7.236459227 | 6.992239112 | 6.67E-06 | 6.21E-05 | 3.692467253 |
| hsa_circ_0001602 | -1.77260802 | 7.551946969 | -6.985645272 | 6.74E-06 | 6.24E-05 | 3.681708529 |
| hsa_circ_0003923 | 1.943537561 | 11.60520118 | 6.977797773 | 6.82E-06 | 6.30E-05 | 3.668896602 |
| hsa_circ_0001861 | -1.034705786 | 8.585710786 | -6.970095784 | 6.91E-06 | 6.36E-05 | 3.656314106 |
| hsa_circ_0092299 | 1.081609337 | 11.86005024 | 6.95889664 | 7.03E-06 | 6.44E-05 | 3.638004044 |
| hsa_circ_0028540 | 1.118161735 | 9.181148102 | 6.952733972 | 7.10E-06 | 6.44E-05 | 3.627921109 |
| hsa_circ_0006421 | 1.268224439 | 9.828683607 | 6.951850244 | 7.11E-06 | 6.44E-05 | 3.626474792 |
| hsa_circ_0003239 | -2.050748327 | 12.61329482 | -6.941829249 | 7.22E-06 | 6.53E-05 | 3.610066898 |
| hsa_circ_0008226 | 1.337341969 | 9.160638015 | 6.939258153 | 7.25E-06 | 6.54E-05 | 3.605854909 |
| hsa_circ_0001874 | -1.43374552 | 9.28102076 | -6.93437275 | 7.31E-06 | 6.55E-05 | 3.597849127 |
| hsa_circ_0004296 | -1.363090214 | 8.622215985 | -6.92490033 | 7.42E-06 | 6.62E-05 | 3.58231728 |
| hsa_circ_0008032 | -1.385113306 | 8.822975347 | -6.91470734 | 7.54E-06 | 6.71E-05 | 3.565590284 |
| hsa_circ_0055033 | 2.569053367 | 8.064071837 | 6.897539709 | 7.75E-06 | 6.84E-05 | 3.537385717 |
| hsa_circ_0006735 | 1.45194151 | 9.325187153 | 6.890246104 | 7.84E-06 | 6.90E-05 | 3.525390959 |
| hsa_circ_0040148 | 2.406350041 | 9.472912755 | 6.885087434 | 7.90E-06 | 6.94E-05 | 3.516902849 |
| hsa_circ_0008305 | -1.413108918 | 12.18407656 | -6.878711126 | 7.98E-06 | 6.97E-05 | 3.506406218 |
| hsa_circ_0007769 | 1.964061663 | 10.44582746 | 6.870879154 | 8.08E-06 | 7.04E-05 | 3.493505697 |
| hsa_circ_0067323 | 1.010902102 | 6.2334935 | 6.857000693 | 8.26E-06 | 7.13E-05 | 3.470625088 |
| hsa_circ_0052863 | 1.315446781 | 6.385575283 | 6.856550399 | 8.27E-06 | 7.13E-05 | 3.469882275 |
| hsa_circ_0067127 | -1.424564867 | 13.68670672 | -6.852358388 | 8.32E-06 | 7.16E-05 | 3.462965725 |
| hsa_circ_0047288 | -1.336255714 | 9.811941173 | -6.847109322 | 8.39E-06 | 7.20E-05 | 3.454301721 |
| hsa_circ_0008514 | 1.957208745 | 8.05768452 | 6.844256189 | 8.43E-06 | 7.20E-05 | 3.449590819 |
| hsa_circ_0023944 | -1.892806286 | 9.915981571 | -6.844156802 | 8.43E-06 | 7.20E-05 | 3.449426699 |
| hsa_circ_0000643 | -1.679153235 | 11.44543772 | -6.841632791 | 8.46E-06 | 7.21E-05 | 3.44525826 |
| hsa_circ_0031570 | 1.702976714 | 7.421262163 | 6.836480426 | 8.53E-06 | 7.23E-05 | 3.43674636 |
| hsa_circ_0002172 | 1.090442444 | 8.287010074 | 6.827634547 | 8.66E-06 | 7.28E-05 | 3.422124182 |
| hsa_circ_0006110 | -2.134605643 | 13.13610838 | -6.825518323 | 8.68E-06 | 7.29E-05 | 3.418624494 |
| hsa_circ_0001936 | -1.777849724 | 13.42906472 | -6.822903254 | 8.72E-06 | 7.30E-05 | 3.414299001 |
| hsa_circ_0001235 | 1.031660617 | 6.702375569 | 6.816288284 | 8.81E-06 | 7.33E-05 | 3.403353241 |
| hsa_circ_0004525 | -1.611178061 | 10.4974984 | -6.815647324 | 8.82E-06 | 7.33E-05 | 3.402292329 |
| hsa_circ_0060158 | -1.043610551 | 6.312374204 | -6.808943412 | 8.92E-06 | 7.39E-05 | 3.39119271 |
| hsa_circ_0050119 | 1.6809605 | 7.13780175 | 6.807617192 | 8.94E-06 | 7.39E-05 | 3.388996169 |
| hsa_circ_0003222 | 1.023815327 | 10.34039038 | 6.804004545 | 8.99E-06 | 7.42E-05 | 3.383011535 |
| hsa_circ_0017636 | 1.548073306 | 11.95750234 | 6.794545284 | 9.13E-06 | 7.50E-05 | 3.367333068 |
| hsa_circ_0092374 | 1.433198184 | 8.1505575 | 6.792376329 | 9.16E-06 | 7.50E-05 | 3.363736361 |
| hsa_circ_0078150 | 1.972267301 | 7.863963278 | 6.784129615 | 9.28E-06 | 7.57E-05 | 3.350055231 |
| hsa_circ_0013339 | -3.950769816 | 8.657519378 | -6.782020687 | 9.31E-06 | 7.57E-05 | 3.346555069 |
| hsa_circ_0004217 | -1.917405337 | 12.47934686 | -6.781158801 | 9.32E-06 | 7.57E-05 | 3.345124432 |
| hsa_circ_0088777 | 1.80073102 | 11.35876935 | 6.780903717 | 9.33E-06 | 7.57E-05 | 3.344701001 |
| hsa_circ_0003497 | -1.388400847 | 12.6103796 | -6.755208077 | 9.72E-06 | 7.83E-05 | 3.30200146 |
| hsa_circ_0001495 | -1.260164163 | 8.680563867 | -6.751802832 | 9.77E-06 | 7.85E-05 | 3.296336038 |
| hsa_circ_0004240 | -1.645984796 | 10.95514956 | -6.744432686 | 9.89E-06 | 7.91E-05 | 3.28406864 |
| hsa_circ_0029346 | 1.143404398 | 6.614518536 | 6.739307295 | 9.97E-06 | 7.94E-05 | 3.275533185 |
| hsa_circ_0018004 | 1.183426949 | 10.0728448 | 6.731359293 | 1.01E-05 | 8.02E-05 | 3.262290046 |
| hsa_circ_0047303 | -1.625302327 | 9.199010459 | -6.724476993 | 1.02E-05 | 8.07E-05 | 3.250815614 |
| hsa_circ_0000861 | -1.095738786 | 7.949694597 | -6.716147858 | 1.03E-05 | 8.16E-05 | 3.236920282 |
| hsa_circ_0008223 | 1.124074755 | 10.92755884 | 6.714386698 | 1.04E-05 | 8.17E-05 | 3.233980957 |
| hsa_circ_0000228 | 1.377037643 | 9.900159026 | 6.710749774 | 1.04E-05 | 8.20E-05 | 3.227909689 |
| hsa_circ_0008301 | 2.312972214 | 9.993911474 | 6.705302336 | 1.05E-05 | 8.25E-05 | 3.218812663 |
| hsa_circ_0044927 | 1.426628582 | 8.004555209 | 6.699514559 | 1.06E-05 | 8.29E-05 | 3.209142827 |
| hsa_circ_0000862 | 1.164815122 | 9.981717337 | 6.685757149 | 1.09E-05 | 8.46E-05 | 3.186139421 |
| hsa_circ_0006097 | -1.408030827 | 8.816734658 | -6.666588073 | 1.12E-05 | 8.66E-05 | 3.154044033 |
| hsa_circ_0000198 | 1.092917204 | 6.816642214 | 6.656465154 | 1.14E-05 | 8.75E-05 | 3.13707457 |
| hsa_circ_0005533 | -2.557494633 | 7.598372418 | -6.638486107 | 1.17E-05 | 8.94E-05 | 3.106900894 |
| hsa_circ_0039943 | -2.1899445 | 11.87637438 | -6.636889797 | 1.18E-05 | 8.95E-05 | 3.104219712 |
| hsa_circ_0036287 | 1.78064776 | 9.353104406 | 6.62569727 | 1.20E-05 | 9.09E-05 | 3.085410779 |
| hsa_circ_0008839 | -1.434973969 | 8.136519679 | -6.622709504 | 1.20E-05 | 9.11E-05 | 3.080386959 |
| hsa_circ_0002041 | -1.641703704 | 10.20272084 | -6.612527677 | 1.22E-05 | 9.22E-05 | 3.063257381 |
| hsa_circ_0003578 | -2.025411449 | 13.00792138 | -6.590589975 | 1.27E-05 | 9.45E-05 | 3.02630171 |
| hsa_circ_0006988 | -1.072314383 | 7.020915594 | -6.588133008 | 1.27E-05 | 9.46E-05 | 3.022158651 |
| hsa_circ_0008383 | 2.718792944 | 11.6823452 | 6.587774191 | 1.27E-05 | 9.46E-05 | 3.021553528 |
| hsa_circ_0037858 | -1.365318133 | 9.074665372 | -6.58175366 | 1.29E-05 | 9.53E-05 | 3.01139761 |
| hsa_circ_0004816 | -1.006287847 | 9.107886077 | -6.577562502 | 1.29E-05 | 9.53E-05 | 3.004324687 |
| hsa_circ_0028198 | 2.329615173 | 12.52163831 | 6.575484851 | 1.30E-05 | 9.54E-05 | 3.000817585 |
| hsa_circ_0009594 | -2.19014451 | 12.69450542 | -6.573565148 | 1.30E-05 | 9.55E-05 | 2.997576576 |
| hsa_circ_0088494 | 2.744531597 | 10.1144005 | 6.570966499 | 1.31E-05 | 9.55E-05 | 2.993188502 |
| hsa_circ_0005562 | -1.793387653 | 11.64236215 | -6.550338884 | 1.35E-05 | 9.80E-05 | 2.958323837 |
| hsa_circ_0030777 | -1.690533122 | 11.03707393 | -6.548504132 | 1.36E-05 | 9.80E-05 | 2.955219918 |
| hsa_circ_0002383 | -1.793144633 | 11.97498247 | -6.54172473 | 1.37E-05 | 9.89E-05 | 2.943746937 |
| hsa_circ_0000508 | -1.534499806 | 11.31545268 | -6.539396174 | 1.38E-05 | 9.91E-05 | 2.939804797 |
| hsa_circ_0003220 | 1.030245327 | 9.461885622 | 6.535214034 | 1.39E-05 | 9.96E-05 | 2.932722748 |
| hsa_circ_0075447 | -1.994886786 | 12.88613664 | -6.530395362 | 1.40E-05 | 9.98E-05 | 2.924559815 |
| hsa_circ_0048129 | -1.097216633 | 8.90893451 | -6.529841829 | 1.40E-05 | 9.98E-05 | 2.923621914 |
| hsa_circ_0074736 | 2.219649821 | 12.5003531 | 6.529653937 | 1.40E-05 | 9.98E-05 | 2.923303542 |
| hsa_circ_0092319 | 1.544635949 | 10.17239881 | 6.524130926 | 1.41E-05 | 0.00010033 | 2.913942955 |
| hsa_circ_0000729 | -1.996309092 | 13.49404866 | -6.509737554 | 1.45E-05 | 0.000102507 | 2.88952887 |
| hsa_circ_0028123 | -1.639380469 | 11.08249052 | -6.498775503 | 1.47E-05 | 0.000103933 | 2.870915895 |
| hsa_circ_0000086 | -1.728803173 | 10.98285138 | -6.49215346 | 1.49E-05 | 0.000104419 | 2.859664016 |
| hsa_circ_0020174 | -1.376601122 | 8.241740857 | -6.483588402 | 1.51E-05 | 0.000105463 | 2.84510171 |
| hsa_circ_0036667 | 1.278501367 | 6.141098724 | 6.479723682 | 1.52E-05 | 0.000105814 | 2.838527611 |
| hsa_circ_0059802 | 2.734861765 | 7.986286658 | 6.473701882 | 1.53E-05 | 0.00010634 | 2.82828011 |
| hsa_circ_0078346 | 1.463621117 | 6.984908895 | 6.47357549 | 1.53E-05 | 0.00010634 | 2.828064971 |
| hsa_circ_0001022 | -1.892705786 | 13.47076129 | -6.4685106 | 1.55E-05 | 0.000107011 | 2.819441928 |
| hsa_circ_0092363 | 1.477057796 | 13.84783957 | 6.462389891 | 1.56E-05 | 0.000107657 | 2.809016632 |
| hsa_circ_0027478 | 2.322658408 | 11.68135188 | 6.446729029 | 1.60E-05 | 0.000109801 | 2.782318303 |
| hsa_circ_0072012 | 1.250793388 | 8.839408418 | 6.440593116 | 1.62E-05 | 0.000110694 | 2.771848723 |
| hsa_circ_0001627 | 1.067826908 | 8.004207291 | 6.435784687 | 1.63E-05 | 0.000111129 | 2.763640584 |
| hsa_circ_0000511 | 1.224503602 | 13.50796547 | 6.429674977 | 1.65E-05 | 0.000111808 | 2.753206529 |
| hsa_circ_0008016 | 1.321317184 | 12.38369523 | 6.427755807 | 1.65E-05 | 0.00011188 | 2.749927944 |
| hsa_circ_0004004 | 1.366411449 | 13.4286678 | 6.414184373 | 1.69E-05 | 0.000113717 | 2.726728943 |
| hsa_circ_0046565 | -1.437740735 | 9.336451673 | -6.412228279 | 1.70E-05 | 0.000113717 | 2.723383109 |
| hsa_circ_0083054 | 1.210176301 | 7.266292186 | 6.410783036 | 1.70E-05 | 0.000113766 | 2.720910731 |
| hsa_circ_0006371 | 1.595259061 | 8.257570643 | 6.384846799 | 1.78E-05 | 0.000118054 | 2.676492757 |
| hsa_circ_0003859 | -1.177730908 | 9.097283362 | -6.367751693 | 1.83E-05 | 0.000121206 | 2.64716539 |
| hsa_circ_0089131 | 2.599511684 | 9.903581321 | 6.365166372 | 1.84E-05 | 0.000121293 | 2.642726666 |
| hsa_circ_0001303 | -1.614060031 | 10.57438231 | -6.360988796 | 1.85E-05 | 0.000121862 | 2.635552266 |
| hsa_circ_0081342 | 1.005808189 | 9.783127742 | 6.348566818 | 1.89E-05 | 0.000123919 | 2.614205093 |
| hsa_circ_0092232 | 2.180027286 | 9.237841102 | 6.330265808 | 1.94E-05 | 0.000126986 | 2.58271616 |
| hsa_circ_0083964 | 2.514398071 | 10.35942469 | 6.329976098 | 1.95E-05 | 0.000126986 | 2.582217312 |
| hsa_circ_0000379 | -2.049387347 | 12.39621585 | -6.319208748 | 1.98E-05 | 0.000128627 | 2.563668948 |
| hsa_circ_0065223 | -1.504000388 | 7.995826153 | -6.315006269 | 1.99E-05 | 0.000129284 | 2.55642523 |
| hsa_circ_0005256 | 1.448627974 | 10.43022726 | 6.313091625 | 2.00E-05 | 0.00012945 | 2.553124198 |
| hsa_circ_0001338 | 1.802419633 | 11.53676907 | 6.311207454 | 2.01E-05 | 0.000129611 | 2.549875212 |
| hsa_circ_0006577 | 1.621859714 | 10.60337267 | 6.30066677 | 2.04E-05 | 0.000131409 | 2.531690297 |
| hsa_circ_0008539 | 1.905073949 | 8.419511954 | 6.292147699 | 2.07E-05 | 0.00013266 | 2.516981943 |
| hsa_circ_0059342 | 1.262959082 | 6.988224735 | 6.291598167 | 2.07E-05 | 0.00013266 | 2.516032822 |
| hsa_circ_0003644 | 1.37156452 | 7.069013801 | 6.285997848 | 2.09E-05 | 0.000133405 | 2.506357901 |
| hsa_circ_0092285 | 1.822929454 | 7.53679264 | 6.277628435 | 2.12E-05 | 0.000134778 | 2.491891161 |
| hsa_circ_0058429 | -1.585680122 | 10.26607872 | -6.275072854 | 2.13E-05 | 0.000134853 | 2.487471858 |
| hsa_circ_0005394 | -1.572286878 | 12.01162496 | -6.264663357 | 2.17E-05 | 0.000136352 | 2.46946171 |
| hsa_circ_0016867 | 2.568506102 | 9.44183302 | 6.264029761 | 2.17E-05 | 0.000136352 | 2.468365003 |
| hsa_circ_0008856 | -1.733486143 | 10.60553787 | -6.262844404 | 2.18E-05 | 0.000136372 | 2.466313094 |
| hsa_circ_0068641 | -1.677030337 | 10.03349183 | -6.257437436 | 2.20E-05 | 0.000137358 | 2.456950926 |
| hsa_circ_0050102 | -1.50211502 | 10.11934142 | -6.252726594 | 2.21E-05 | 0.000137431 | 2.448790832 |
| hsa_circ_0007248 | 1.251798806 | 6.734247046 | 6.251300402 | 2.22E-05 | 0.000137431 | 2.446319788 |
| hsa_circ_0062389 | 1.757280582 | 13.7544393 | 6.250581679 | 2.22E-05 | 0.000137431 | 2.445074412 |
| hsa_circ_0092303 | 1.07362927 | 8.958313319 | 6.247709339 | 2.23E-05 | 0.000137843 | 2.44009662 |
| hsa_circ_0057328 | -2.10028751 | 10.16438713 | -6.244801344 | 2.24E-05 | 0.000138073 | 2.435055884 |
| hsa_circ_0069570 | -1.443977878 | 8.874140612 | -6.244554258 | 2.24E-05 | 0.000138073 | 2.434627531 |
| hsa_circ_0002807 | -1.720739745 | 11.24539247 | -6.241994787 | 2.25E-05 | 0.000138417 | 2.430189881 |
| hsa_circ_0074854 | 2.556964383 | 12.77210379 | 6.219106192 | 2.34E-05 | 0.000143055 | 2.390465346 |
| hsa_circ_0004913 | -3.570311245 | 7.290361408 | -6.209945989 | 2.38E-05 | 0.000144752 | 2.374547131 |
| hsa_circ_0000958 | -1.700125551 | 11.35626243 | -6.202557829 | 2.41E-05 | 0.000145779 | 2.36169992 |
| hsa_circ_0019172 | -1.537374959 | 9.282062173 | -6.199775721 | 2.42E-05 | 0.000146015 | 2.356860196 |
| hsa_circ_0063158 | -1.735634041 | 12.30884205 | -6.174849572 | 2.52E-05 | 0.000150827 | 2.313451629 |
| hsa_circ_0073271 | 1.083340418 | 11.32556028 | 6.173118395 | 2.53E-05 | 0.000150827 | 2.310433645 |
| hsa_circ_0000257 | 1.48735749 | 12.61678872 | 6.170893942 | 2.54E-05 | 0.000150827 | 2.306555126 |
| hsa_circ_0006014 | 1.171389429 | 7.896023347 | 6.17082358 | 2.54E-05 | 0.000150827 | 2.306432433 |
| hsa_circ_0058493 | -3.646951327 | 7.849733357 | -6.168467901 | 2.55E-05 | 0.00015111 | 2.302324349 |
| hsa_circ_0016863 | 2.762647959 | 8.772811388 | 6.165366096 | 2.56E-05 | 0.000151429 | 2.296913931 |
| hsa_circ_0004441 | -1.367274163 | 9.260659276 | -6.163252754 | 2.57E-05 | 0.000151707 | 2.293226917 |
| hsa_circ_0008615 | 1.162346878 | 6.903446061 | 6.133417589 | 2.70E-05 | 0.000158166 | 2.24111016 |
| hsa_circ_0041555 | -1.849752643 | 13.62338227 | -6.132421507 | 2.71E-05 | 0.000158166 | 2.239368081 |
| hsa_circ_0092333 | 1.633761969 | 8.210634842 | 6.130058257 | 2.72E-05 | 0.000158527 | 2.235234375 |
| hsa_circ_0079485 | -1.435479015 | 7.816310584 | -6.128078248 | 2.73E-05 | 0.000158713 | 2.231770433 |
| hsa_circ_0001296 | -1.070311786 | 15.31626732 | -6.127346135 | 2.73E-05 | 0.000158713 | 2.230489496 |
| hsa_circ_0047821 | -1.472675306 | 9.703686898 | -6.1181217 | 2.77E-05 | 0.000160431 | 2.214343745 |
| hsa_circ_0030569 | -1.511152388 | 11.83617647 | -6.116687644 | 2.78E-05 | 0.000160507 | 2.211832637 |
| hsa_circ_0077765 | -1.632596806 | 9.47599923 | -6.102977949 | 2.85E-05 | 0.00016372 | 2.187812061 |
| hsa_circ_0079958 | 1.54092075 | 8.398083538 | 6.094685389 | 2.89E-05 | 0.00016501 | 2.173270316 |
| hsa_circ_0000916 | 1.477558184 | 10.01112027 | 6.094381518 | 2.89E-05 | 0.00016501 | 2.172737273 |
| hsa_circ_0068606 | -1.966024286 | 11.7130596 | -6.088961381 | 2.92E-05 | 0.000166255 | 2.16322728 |
| hsa_circ_0052760 | 1.033933857 | 6.931422122 | 6.075144688 | 2.98E-05 | 0.00016961 | 2.138966834 |
| hsa_circ_0066444 | 2.037121918 | 7.45030198 | 6.071362071 | 3.00E-05 | 0.00016961 | 2.132320474 |
| hsa_circ_0008144 | -1.340594959 | 8.472357571 | -6.071304209 | 3.00E-05 | 0.00016961 | 2.132218791 |
| hsa_circ_0070934 | 2.509775276 | 8.583686832 | 6.067320708 | 3.02E-05 | 0.000170479 | 2.125217322 |
| hsa_circ_0008419 | 2.317070602 | 9.286610679 | 6.064890138 | 3.04E-05 | 0.000170902 | 2.120944247 |
| hsa_circ_0059545 | -1.177645622 | 7.386150668 | -6.050028729 | 3.11E-05 | 0.000174697 | 2.094799569 |
| hsa_circ_0055412 | -1.709586296 | 12.09197452 | -6.045642633 | 3.14E-05 | 0.000175716 | 2.087077653 |
| hsa_circ_0042458 | 1.089889204 | 9.29984202 | 6.04093713 | 3.16E-05 | 0.00017655 | 2.078790494 |
| hsa_circ_0002696 | -1.903628367 | 14.09807403 | -6.039818841 | 3.17E-05 | 0.000176598 | 2.076820561 |
| hsa_circ_0006686 | 1.279710153 | 6.661108954 | 6.037884916 | 3.18E-05 | 0.000176891 | 2.073413436 |
| hsa_circ_0007503 | 1.291409949 | 8.337141301 | 6.035070733 | 3.19E-05 | 0.000177452 | 2.068454591 |
| hsa_circ_0000208 | -1.054920092 | 5.993633434 | -6.017025058 | 3.29E-05 | 0.000181966 | 2.036630863 |
| hsa_circ_0071989 | -1.530722867 | 14.42133983 | -6.016520203 | 3.30E-05 | 0.000181966 | 2.035739908 |
| hsa_circ_0000059 | -1.693322378 | 12.43982513 | -6.011104208 | 3.33E-05 | 0.000183211 | 2.026179734 |
| hsa_circ_0074903 | 1.061602582 | 13.46351138 | 6.00486379 | 3.36E-05 | 0.000184428 | 2.015159365 |
| hsa_circ_0084984 | -1.453074571 | 13.01752281 | -6.003215823 | 3.37E-05 | 0.000184651 | 2.012248228 |
| hsa_circ_0000788 | -1.376984929 | 14.18629566 | -5.998175635 | 3.40E-05 | 0.000185652 | 2.003342434 |
| hsa_circ_0068462 | -1.320974347 | 11.92985095 | -5.993432422 | 3.43E-05 | 0.000186766 | 1.99495823 |
| hsa_circ_0088046 | 1.884923204 | 10.21922013 | 5.992803816 | 3.43E-05 | 0.000186766 | 1.993846863 |
| hsa_circ_0022505 | 1.035142071 | 8.313377832 | 5.989876762 | 3.45E-05 | 0.000187106 | 1.988671166 |
| hsa_circ_0000291 | 1.034140102 | 9.868163551 | 5.983460714 | 3.49E-05 | 0.000188571 | 1.977322065 |
| hsa_circ_0001892 | 1.718345551 | 10.44906308 | 5.979350593 | 3.51E-05 | 0.000189601 | 1.970048893 |
| hsa_circ_0008351 | -1.082376153 | 6.457991128 | -5.97280678 | 3.55E-05 | 0.000191432 | 1.95846439 |
| hsa_circ_0003162 | -1.630506969 | 14.49297886 | -5.963503295 | 3.61E-05 | 0.000194199 | 1.941984447 |
| hsa_circ_0000999 | -1.971746316 | 10.28761561 | -5.960880489 | 3.63E-05 | 0.00019446 | 1.937336357 |
| hsa_circ_0092308 | 1.030239959 | 9.214778051 | 5.959024196 | 3.64E-05 | 0.000194774 | 1.934046106 |
| hsa_circ_0047744 | -1.829401612 | 11.76498351 | -5.953145165 | 3.67E-05 | 0.000195835 | 1.923622529 |
| hsa_circ_0089852 | -1.132952189 | 6.359335115 | -5.953115475 | 3.67E-05 | 0.000195835 | 1.923569878 |
| hsa_circ_0029976 | -1.773848561 | 11.70327435 | -5.941755422 | 3.75E-05 | 0.000198448 | 1.903415093 |
| hsa_circ_0005480 | -1.814754796 | 13.06054972 | -5.929927315 | 3.82E-05 | 0.00020187 | 1.882411335 |
| hsa_circ_0092283 | 2.275079923 | 14.03618322 | 5.929090224 | 3.83E-05 | 0.00020187 | 1.880924154 |
| hsa_circ_0007763 | -1.432238561 | 8.867204454 | -5.928157982 | 3.84E-05 | 0.000201882 | 1.879267816 |
| hsa_circ_0004223 | 1.075263367 | 7.58258449 | 5.921406589 | 3.88E-05 | 0.000203648 | 1.867268934 |
| hsa_circ_0001301 | -1.269173735 | 7.072475867 | -5.921294264 | 3.88E-05 | 0.000203648 | 1.867069252 |
| hsa_circ_0042819 | 1.87921852 | 8.622126801 | 5.915783615 | 3.92E-05 | 0.000204956 | 1.857270827 |
| hsa_circ_0004606 | 1.105830378 | 7.047218056 | 5.89378877 | 4.07E-05 | 0.000210284 | 1.818121182 |
| hsa_circ_0001901 | 1.149950878 | 9.170764724 | 5.877004569 | 4.19E-05 | 0.000215477 | 1.788202308 |
| hsa_circ_0002490 | -1.174559388 | 9.689873153 | -5.872905477 | 4.22E-05 | 0.000216354 | 1.780889656 |
| hsa_circ_0004053 | 1.238223367 | 6.855899469 | 5.870509866 | 4.24E-05 | 0.000216623 | 1.776614913 |
| hsa_circ_0008184 | -1.302398112 | 9.387338219 | -5.870447812 | 4.24E-05 | 0.000216623 | 1.776504173 |
| hsa_circ_0003098 | 1.489223071 | 8.519907005 | 5.866744712 | 4.26E-05 | 0.000217685 | 1.769894787 |
| hsa_circ_0001056 | 1.203372633 | 11.1726195 | 5.865255025 | 4.27E-05 | 0.000217783 | 1.767235436 |
| hsa_circ_0022249 | -1.245622255 | 9.854223066 | -5.86475567 | 4.28E-05 | 0.000217783 | 1.766343936 |
| hsa_circ_0004207 | -1.416270745 | 10.40971095 | -5.846892928 | 4.41E-05 | 0.000224272 | 1.73443138 |
| hsa_circ_0000730 | -2.501146541 | 6.625536791 | -5.844369444 | 4.43E-05 | 0.000224806 | 1.72991961 |
| hsa_circ_0056285 | -1.50883351 | 10.82393361 | -5.843799818 | 4.44E-05 | 0.000224806 | 1.72890105 |
| hsa_circ_0052318 | -1.792416776 | 14.39298265 | -5.838041947 | 4.48E-05 | 0.000226719 | 1.718602834 |
| hsa_circ_0062022 | 1.784911888 | 10.91060072 | 5.830972827 | 4.54E-05 | 0.000228831 | 1.705953306 |
| hsa_circ_0070933 | 1.582698408 | 7.623670286 | 5.824576079 | 4.59E-05 | 0.000230992 | 1.694501143 |
| hsa_circ_0000191 | -1.238497765 | 10.5776263 | -5.823780532 | 4.59E-05 | 0.000230992 | 1.693076483 |
| hsa_circ_0038773 | 1.105929592 | 7.543133776 | 5.822169149 | 4.60E-05 | 0.000230992 | 1.690190572 |
| hsa_circ_0008942 | 1.046433888 | 7.778829281 | 5.822140415 | 4.60E-05 | 0.000230992 | 1.690139107 |
| hsa_circ_0007178 | -1.719392633 | 9.579022735 | -5.821131632 | 4.61E-05 | 0.000231057 | 1.688332243 |
| hsa_circ_0003952 | -1.107023745 | 7.756869209 | -5.808559245 | 4.71E-05 | 0.000235112 | 1.665801982 |
| hsa_circ_0010486 | 1.125439408 | 11.96217113 | 5.790004773 | 4.87E-05 | 0.000241044 | 1.632512912 |
| hsa_circ_0059175 | 1.058633765 | 8.895335995 | 5.78233038 | 4.93E-05 | 0.000243925 | 1.618730623 |
| hsa_circ_0003503 | -1.704931888 | 8.358498066 | -5.780130854 | 4.95E-05 | 0.000244506 | 1.614779086 |
| hsa_circ_0083294 | -1.824905449 | 13.78680374 | -5.776570713 | 4.98E-05 | 0.000245668 | 1.608381784 |
| hsa_circ_0008274 | 3.53036851 | 12.43154557 | 5.767063502 | 5.07E-05 | 0.000249249 | 1.591289758 |
| hsa_circ_0075825 | -1.723606378 | 10.9901748 | -5.758357758 | 5.14E-05 | 0.000251636 | 1.575628035 |
| hsa_circ_0029642 | -1.320818408 | 7.928232582 | -5.75618191 | 5.16E-05 | 0.000251993 | 1.571712086 |
| hsa_circ_0020926 | -1.819148949 | 12.91244415 | -5.751921404 | 5.20E-05 | 0.000253146 | 1.564042476 |
| hsa_circ_0000434 | 1.51120899 | 7.636510168 | 5.743967578 | 5.27E-05 | 0.000256094 | 1.549717817 |
| hsa_circ_0007345 | 2.03419898 | 10.00040929 | 5.739340362 | 5.32E-05 | 0.00025729 | 1.541380434 |
| hsa_circ_0069249 | -2.641974694 | 11.79482973 | -5.733879986 | 5.37E-05 | 0.000258967 | 1.531538191 |
| hsa_circ_0008234 | 2.113333653 | 7.877339449 | 5.733543528 | 5.37E-05 | 0.000258967 | 1.530931601 |
| hsa_circ_0068075 | 1.777909071 | 10.06422797 | 5.733183291 | 5.37E-05 | 0.000258967 | 1.530282125 |
| hsa_circ_0002158 | 1.888924893 | 7.724583339 | 5.730344978 | 5.40E-05 | 0.000259762 | 1.52516428 |
| hsa_circ_0011950 | 1.94197798 | 7.661507704 | 5.727608445 | 5.43E-05 | 0.000260031 | 1.520228948 |
| hsa_circ_0000549 | -1.613534235 | 11.65564647 | -5.721172784 | 5.49E-05 | 0.000261867 | 1.508618321 |
| hsa_circ_0064288 | 1.077650469 | 12.3174014 | 5.715452833 | 5.54E-05 | 0.000263026 | 1.498294299 |
| hsa_circ_0001834 | 1.656934 | 12.53743533 | 5.710881832 | 5.59E-05 | 0.000264767 | 1.490040915 |
| hsa_circ_0006517 | 1.106312714 | 14.74225243 | 5.70734001 | 5.62E-05 | 0.000266042 | 1.483643909 |
| hsa_circ_0041811 | 1.185939888 | 7.84648124 | 5.70036104 | 5.69E-05 | 0.000267824 | 1.471034088 |
| hsa_circ_0008797 | -1.523085316 | 13.43073367 | -5.698078523 | 5.71E-05 | 0.000268015 | 1.466908566 |
| hsa_circ_0035381 | -1.376943245 | 14.98891605 | -5.696989291 | 5.73E-05 | 0.000268015 | 1.464939597 |
| hsa_circ_0056264 | 1.018343531 | 6.878246153 | 5.695873439 | 5.74E-05 | 0.000268015 | 1.462922344 |
| hsa_circ_0053967 | -1.696363 | 11.3999214 | -5.688402914 | 5.81E-05 | 0.00027002 | 1.449412786 |
| hsa_circ_0003056 | -1.242472551 | 8.902086673 | -5.68709952 | 5.83E-05 | 0.00027002 | 1.447054997 |
| hsa_circ_0004539 | -1.786127388 | 10.52022317 | -5.682347435 | 5.87E-05 | 0.000271907 | 1.438456757 |
| hsa_circ_0070659 | -1.825924684 | 11.93736481 | -5.672690669 | 5.97E-05 | 0.000275799 | 1.420974988 |
| hsa_circ_0001513 | 1.385106622 | 10.02661968 | 5.671269177 | 5.99E-05 | 0.00027594 | 1.418400602 |
| hsa_circ_0002348 | -1.64722301 | 10.53685126 | -5.670859591 | 5.99E-05 | 0.00027594 | 1.417658775 |
| hsa_circ_0057553 | -1.142285184 | 6.598346827 | -5.66047509 | 6.10E-05 | 0.000279504 | 1.398843341 |
| hsa_circ_0088807 | 1.173462026 | 8.837621804 | 5.640766029 | 6.32E-05 | 0.000288185 | 1.363093901 |
| hsa_circ_0054254 | -1.023838388 | 7.520003765 | -5.635423001 | 6.38E-05 | 0.000289743 | 1.353393606 |
| hsa_circ_0000507 | -1.276879622 | 10.19960304 | -5.631051068 | 6.43E-05 | 0.000291591 | 1.34545355 |
| hsa_circ_0034762 | -1.073674694 | 15.39591966 | -5.624186117 | 6.51E-05 | 0.000293962 | 1.332980755 |
| hsa_circ_0007396 | 1.634478816 | 7.213564296 | 5.622305795 | 6.53E-05 | 0.000294546 | 1.329563357 |
| hsa_circ_0009076 | -1.993463173 | 13.54283658 | -5.614264954 | 6.62E-05 | 0.000297957 | 1.314944271 |
| hsa_circ_0000630 | -1.058014923 | 7.375320467 | -5.60790012 | 6.69E-05 | 0.000300268 | 1.303366337 |
| hsa_circ_0001955 | 3.803487959 | 13.02129466 | 5.606149438 | 6.72E-05 | 0.000300268 | 1.300180835 |
| hsa_circ_0069104 | 3.022454531 | 11.61646991 | 5.597802138 | 6.82E-05 | 0.000303695 | 1.284986758 |
| hsa_circ_0008661 | -1.269328694 | 7.952598684 | -5.597779065 | 6.82E-05 | 0.000303695 | 1.284944748 |
| hsa_circ_0092310 | 1.011365541 | 12.97716029 | 5.597487078 | 6.82E-05 | 0.000303695 | 1.284413097 |
| hsa_circ_0000223 | -1.413312357 | 8.499105709 | -5.595201013 | 6.85E-05 | 0.000303871 | 1.28025023 |
| hsa_circ_0008139 | -1.525101199 | 7.916948742 | -5.594969491 | 6.85E-05 | 0.000303871 | 1.279828597 |
| hsa_circ_0043947 | -1.99484749 | 13.0397347 | -5.594943286 | 6.85E-05 | 0.000303871 | 1.279780873 |
| hsa_circ_0002374 | -1.422789847 | 8.325959505 | -5.594111195 | 6.86E-05 | 0.000303923 | 1.278265459 |
| hsa_circ_0047151 | -1.572086 | 12.67507557 | -5.59085242 | 6.90E-05 | 0.000305279 | 1.27232967 |
| hsa_circ_0006168 | -3.374717327 | 7.093582765 | -5.586217864 | 6.96E-05 | 0.000307387 | 1.263885537 |
| hsa_circ_0002513 | 1.712493031 | 7.86872774 | 5.583380619 | 6.99E-05 | 0.000307735 | 1.258714713 |
| hsa_circ_0018529 | -1.68503748 | 10.77573281 | -5.580585794 | 7.03E-05 | 0.000308797 | 1.25362017 |
| hsa_circ_0028135 | 1.057652816 | 8.834015092 | 5.579322185 | 7.04E-05 | 0.000308797 | 1.251316469 |
| hsa_circ_0009456 | 1.119993694 | 10.43511793 | 5.579244245 | 7.04E-05 | 0.000308797 | 1.251174369 |
| hsa_circ_0000560 | 1.768712459 | 7.792177658 | 5.577889332 | 7.06E-05 | 0.000309139 | 1.248703964 |
| hsa_circ_0063329 | -1.113002663 | 7.741133352 | -5.571715215 | 7.14E-05 | 0.000312128 | 1.237443709 |
| hsa_circ_0007372 | 2.038448388 | 9.996493714 | 5.557362217 | 7.32E-05 | 0.000317753 | 1.211247779 |
| hsa_circ_0000670 | -1.949912429 | 14.2480743 | -5.557279899 | 7.32E-05 | 0.000317753 | 1.211097461 |
| hsa_circ_0073736 | -1.765225985 | 11.10846938 | -5.553251249 | 7.37E-05 | 0.000319214 | 1.203739841 |
| hsa_circ_0052523 | 1.534782776 | 7.559483929 | 5.54765617 | 7.45E-05 | 0.000321713 | 1.193517919 |
| hsa_circ_0000662 | -1.596363449 | 14.086256 | -5.542829467 | 7.51E-05 | 0.000323925 | 1.18469652 |
| hsa_circ_0072758 | 2.147274776 | 8.471635204 | 5.539548094 | 7.55E-05 | 0.000324583 | 1.178697678 |
| hsa_circ_0072389 | 1.083980327 | 8.037081224 | 5.534133119 | 7.63E-05 | 0.000326649 | 1.168795244 |
| hsa_circ_0092316 | 1.352657745 | 8.556180719 | 5.53383871 | 7.63E-05 | 0.000326649 | 1.168256746 |
| hsa_circ_0016873 | 1.31905198 | 8.288929918 | 5.530668961 | 7.67E-05 | 0.000327668 | 1.162458303 |
| hsa_circ_0008666 | -2.62289751 | 6.786324102 | -5.511734271 | 7.94E-05 | 0.000336691 | 1.127793908 |
| hsa_circ_0073568 | -2.094215163 | 6.416648434 | -5.509521849 | 7.97E-05 | 0.000336822 | 1.123740532 |
| hsa_circ_0001845 | -1.689341622 | 9.373932219 | -5.494875331 | 8.18E-05 | 0.000343572 | 1.096890753 |
| hsa_circ_0076092 | -1.759616296 | 13.31899681 | -5.485445293 | 8.32E-05 | 0.000348096 | 1.079589147 |
| hsa_circ_0000321 | -2.160942561 | 6.879774842 | -5.471463889 | 8.52E-05 | 0.000355729 | 1.053915992 |
| hsa_circ_0031017 | -1.284999449 | 11.01059302 | -5.47119047 | 8.53E-05 | 0.000355729 | 1.053413682 |
| hsa_circ_0020117 | 1.559084082 | 9.808007663 | 5.463183242 | 8.65E-05 | 0.000359959 | 1.038698964 |
| hsa_circ_0001497 | -1.163991582 | 9.709005332 | -5.44990837 | 8.86E-05 | 0.000365903 | 1.014285952 |
| hsa_circ_0004033 | -1.519432357 | 10.9228174 | -5.439764349 | 9.02E-05 | 0.000371685 | 0.995615541 |
| hsa_circ_0005777 | -1.569072622 | 8.914114311 | -5.427868498 | 9.21E-05 | 0.000378294 | 0.973704138 |
| hsa_circ_0068176 | 1.227670765 | 9.146948444 | 5.425105518 | 9.26E-05 | 0.000378827 | 0.96861233 |
| hsa_circ_0092328 | -1.662564372 | 11.26982382 | -5.424395406 | 9.27E-05 | 0.000378827 | 0.967303532 |
| hsa_circ_0008472 | 1.326420622 | 9.038582709 | 5.421056809 | 9.33E-05 | 0.000380638 | 0.961149343 |
| hsa_circ_0000322 | 2.393824704 | 9.616499107 | 5.4133652 | 9.46E-05 | 0.000384073 | 0.946965667 |
| hsa_circ_0036282 | -1.703582765 | 10.43381334 | -5.409767408 | 9.52E-05 | 0.000386093 | 0.940328602 |
| hsa_circ_0007961 | -1.17433048 | 7.037319413 | -5.401554241 | 9.66E-05 | 0.000390106 | 0.925171136 |
| hsa_circ_0004587 | 1.909412128 | 8.187444212 | 5.401515945 | 9.66E-05 | 0.000390106 | 0.92510044 |
| hsa_circ_0000550 | -1.544562714 | 10.05769971 | -5.400988114 | 9.67E-05 | 0.000390106 | 0.92412603 |
| hsa_circ_0043278 | -1.187831939 | 14.96587276 | -5.400678742 | 9.67E-05 | 0.000390106 | 0.923554892 |
| hsa_circ_0015928 | 1.252291643 | 8.978439056 | 5.39948055 | 9.69E-05 | 0.000390482 | 0.921342772 |
| hsa_circ_0003611 | -1.284649347 | 9.540866265 | -5.393365095 | 9.80E-05 | 0.000392976 | 0.91004951 |
| hsa_circ_0045128 | 1.026666459 | 9.101948903 | 5.38833252 | 9.89E-05 | 0.000395557 | 0.900752436 |
| hsa_circ_0000034 | -1.06284401 | 9.317461658 | -5.381582088 | 0.000100093 | 0.000399902 | 0.888276815 |
| hsa_circ_0002078 | 1.000392357 | 8.523381158 | 5.37696173 | 0.000100925 | 0.000402288 | 0.879734523 |
| hsa_circ_0071865 | 1.367415041 | 8.781947776 | 5.373033835 | 0.000101639 | 0.000404514 | 0.872470372 |
| hsa_circ_0007772 | 1.993519949 | 8.809697474 | 5.372583752 | 0.000101721 | 0.000404514 | 0.871637874 |
| hsa_circ_0068631 | -1.528350949 | 10.95488078 | -5.371536208 | 0.000101912 | 0.000404804 | 0.869700187 |
| hsa_circ_0071834 | -1.344461724 | 8.36124026 | -5.367260183 | 0.000102697 | 0.000407447 | 0.861789201 |
| hsa_circ_0001359 | 1.799896122 | 10.12435364 | 5.36520322 | 0.000103077 | 0.000408108 | 0.857982841 |
| hsa_circ_0002069 | -1.278798286 | 13.46884429 | -5.365064393 | 0.000103102 | 0.000408108 | 0.857725926 |
| hsa_circ_0074817 | 2.588260704 | 10.10409592 | 5.356008275 | 0.000104792 | 0.00041336 | 0.840961324 |
| hsa_circ_0001561 | -1.684628214 | 10.20364122 | -5.342240809 | 0.000107417 | 0.000422534 | 0.81545545 |
| hsa_circ_0063853 | -3.109287847 | 9.789233689 | -5.340117888 | 0.000107827 | 0.000423378 | 0.81152038 |
| hsa_circ_0017639 | 2.214901969 | 10.27138698 | 5.338990694 | 0.000108046 | 0.00042375 | 0.809430769 |
| hsa_circ_0003028 | 1.66907498 | 8.633190061 | 5.332849106 | 0.000109246 | 0.000427907 | 0.798042618 |
| hsa_circ_0036044 | -2.867449306 | 8.325410082 | -5.332287348 | 0.000109357 | 0.000427907 | 0.797000733 |
| hsa_circ_0004599 | 2.375028439 | 11.25912849 | 5.318005807 | 0.000112204 | 0.000437546 | 0.770499767 |
| hsa_circ_0019627 | 1.391167878 | 7.71757549 | 5.308311601 | 0.000114181 | 0.000444048 | 0.752496666 |
| hsa_circ_0008594 | -1.633755276 | 10.60004115 | -5.30728971 | 0.000114392 | 0.000444048 | 0.750598235 |
| hsa_circ_0077179 | -1.044217969 | 8.040201444 | -5.30432776 | 0.000115004 | 0.000445919 | 0.745094903 |
| hsa_circ_0007018 | 2.933842163 | 10.76745131 | 5.299946047 | 0.000115916 | 0.000448438 | 0.736951645 |
| hsa_circ_0051637 | -1.187434214 | 8.825304648 | -5.288711485 | 0.00011829 | 0.000456537 | 0.716061766 |
| hsa_circ_0001204 | -1.352598143 | 12.14356686 | -5.288144672 | 0.000118411 | 0.000456537 | 0.715007404 |
| hsa_circ_0003528 | 2.406922061 | 12.7969679 | 5.287397885 | 0.000118571 | 0.000456637 | 0.713618201 |
| hsa_circ_0038646 | -1.01322752 | 7.50263724 | -5.28481427 | 0.000119125 | 0.000458255 | 0.708811528 |
| hsa_circ_0065299 | -1.183536071 | 6.791970566 | -5.283643264 | 0.000119377 | 0.000458453 | 0.706632666 |
| hsa_circ_0000699 | -1.036407143 | 6.778403061 | -5.283329335 | 0.000119445 | 0.000458453 | 0.706048518 |
| hsa_circ_0002454 | 1.943229 | 10.17438652 | 5.282641254 | 0.000119593 | 0.000458508 | 0.704768117 |
| hsa_circ_0002828 | -2.209111847 | 10.87431542 | -5.281831202 | 0.000119768 | 0.000458664 | 0.703260673 |
| hsa_circ_0070033 | -1.373436214 | 8.32865377 | -5.279122204 | 0.000120356 | 0.000460196 | 0.698218854 |
| hsa_circ_0070348 | 1.46740852 | 7.796354015 | 5.275539186 | 0.000121137 | 0.000462352 | 0.691548977 |
| hsa_circ_0072255 | 1.66280648 | 7.524192301 | 5.261825885 | 0.000124177 | 0.000471847 | 0.666006752 |
| hsa_circ_0031554 | -1.249404337 | 11.64703727 | -5.252511538 | 0.000126288 | 0.0004788 | 0.648644817 |
| hsa_circ_0013561 | -1.951422673 | 7.459141204 | -5.245317296 | 0.000127943 | 0.000483739 | 0.635227494 |
| hsa_circ_0028048 | -1.332421429 | 10.76073839 | -5.245301312 | 0.000127947 | 0.000483739 | 0.635197679 |
| hsa_circ_0092235 | 1.176813418 | 7.58717223 | 5.243489967 | 0.000128367 | 0.000483739 | 0.631818504 |
| hsa_circ_0042823 | 2.151845806 | 8.978570056 | 5.243188715 | 0.000128437 | 0.000483739 | 0.631256461 |
| hsa_circ_0082564 | 1.282529 | 14.26621432 | 5.242133664 | 0.000128683 | 0.000484119 | 0.629287977 |
| hsa_circ_0000331 | 1.008172551 | 8.761245561 | 5.23992082 | 0.0001292 | 0.000485529 | 0.625158874 |
| hsa_circ_0040414 | -1.041104827 | 6.296181097 | -5.235575486 | 0.000130221 | 0.000488577 | 0.617048872 |
| hsa_circ_0016726 | -1.783405112 | 11.12857326 | -5.231522774 | 0.000131181 | 0.000490381 | 0.609482937 |
| hsa_circ_0006553 | 2.196873347 | 12.73989551 | 5.231405879 | 0.000131208 | 0.000490381 | 0.609264678 |
| hsa_circ_0072264 | -1.755097959 | 7.855080908 | -5.209460424 | 0.000136537 | 0.00050532 | 0.568260159 |
| hsa_circ_0048259 | 1.102617041 | 12.5052207 | 5.205606389 | 0.000137496 | 0.000508318 | 0.561052973 |
| hsa_circ_0078155 | 1.029714658 | 9.256690773 | 5.204664405 | 0.000137731 | 0.000508638 | 0.559291155 |
| hsa_circ_0004370 | 1.69631252 | 7.13938576 | 5.203410798 | 0.000138045 | 0.000509247 | 0.556946335 |
| hsa_circ_0074816 | 1.625215612 | 8.531120663 | 5.19963312 | 0.000138996 | 0.000511648 | 0.549879197 |
| hsa_circ_0004976 | 1.932707622 | 12.29455805 | 5.197705884 | 0.000139483 | 0.00051289 | 0.546273135 |
| hsa_circ_0009022 | 1.110779765 | 6.815170311 | 5.193468522 | 0.000140562 | 0.000516299 | 0.538343005 |
| hsa_circ_0003214 | -1.392433867 | 7.207816413 | -5.185941957 | 0.000142498 | 0.000522289 | 0.524251877 |
| hsa_circ_0000045 | -1.333758469 | 9.464316102 | -5.184455283 | 0.000142884 | 0.000523142 | 0.521467743 |
| hsa_circ_0008682 | -1.439378469 | 8.361879827 | -5.17409018 | 0.000145604 | 0.000532531 | 0.502049361 |
| hsa_circ_0047688 | 1.840748306 | 6.676186531 | 5.171672553 | 0.000146246 | 0.000534307 | 0.497518236 |
| hsa_circ_0067997 | -1.384870046 | 7.105934651 | -5.168627361 | 0.000147059 | 0.000536704 | 0.491809929 |
| hsa_circ_0002320 | 1.358485316 | 9.258786862 | 5.168025788 | 0.000147221 | 0.000536719 | 0.49068213 |
| hsa_circ_0004315 | 2.743755949 | 10.74256787 | 5.16633968 | 0.000147673 | 0.000537795 | 0.487520871 |
| hsa_circ_0041382 | -1.882914898 | 13.49433677 | -5.160812887 | 0.000149167 | 0.000542081 | 0.477156386 |
| hsa_circ_0032683 | -2.955215827 | 10.36601741 | -5.15890729 | 0.000149686 | 0.000543388 | 0.473581943 |
| hsa_circ_0043428 | 1.16388949 | 6.600016531 | 5.15624682 | 0.000150414 | 0.000545449 | 0.468590817 |
| hsa_circ_0036005 | 1.604090755 | 13.51953479 | 5.151729974 | 0.000151657 | 0.000548794 | 0.460115139 |
| hsa_circ_0059859 | 1.496019133 | 9.887858281 | 5.138358559 | 0.000155401 | 0.000560561 | 0.435010007 |
| hsa_circ_0032704 | 3.027867092 | 11.43472697 | 5.135703011 | 0.000156156 | 0.00056269 | 0.430021632 |
| hsa_circ_0005513 | -1.566805776 | 14.02631873 | -5.122262451 | 0.000160036 | 0.000572444 | 0.404761111 |
| hsa_circ_0005358 | -1.719423408 | 7.725610316 | -5.099943354 | 0.000166702 | 0.000594421 | 0.362767036 |
| hsa_circ_0002372 | -1.455993 | 9.452472 | -5.098723166 | 0.000167075 | 0.000595128 | 0.360469529 |
| hsa_circ_0003839 | 1.163169398 | 11.13995172 | 5.095851381 | 0.000167955 | 0.000597019 | 0.355061522 |
| hsa_circ_0008657 | 1.815973949 | 6.827385944 | 5.070606939 | 0.000175906 | 0.0006214 | 0.307481007 |
| hsa_circ_0003006 | 2.599076959 | 11.06759282 | 5.062015549 | 0.0001787 | 0.000629317 | 0.291271138 |
| hsa_circ_0051042 | 1.755574204 | 8.66162101 | 5.052051584 | 0.000181998 | 0.00063634 | 0.272460861 |
| hsa_circ_0005785 | 1.920484418 | 12.84376681 | 5.048816316 | 0.000183082 | 0.000639477 | 0.266350762 |
| hsa_circ_0026911 | -1.350052296 | 6.693870505 | -5.047268225 | 0.000183603 | 0.000640643 | 0.263426625 |
| hsa_circ_0002522 | -1.167942143 | 11.68018596 | -5.032663064 | 0.000188598 | 0.000653398 | 0.235825866 |
| hsa_circ_0024553 | 1.205016745 | 7.834052393 | 5.031251189 | 0.000189088 | 0.000653405 | 0.233156415 |
| hsa_circ_0036117 | 1.200273378 | 6.936084138 | 5.031004772 | 0.000189174 | 0.000653405 | 0.232690489 |
| hsa_circ_0068850 | 1.417954204 | 8.65200799 | 5.028683113 | 0.000189983 | 0.000655538 | 0.228300336 |
| hsa_circ_0011692 | 1.305236969 | 13.04071464 | 5.027325358 | 0.000190458 | 0.000655852 | 0.225732597 |
| hsa_circ_0038608 | -1.755293071 | 11.85724777 | -5.016854725 | 0.000194163 | 0.000666493 | 0.205923831 |
| hsa_circ_0045601 | -1.142677122 | 8.528034133 | -4.993186848 | 0.000202816 | 0.000689923 | 0.161102071 |
| hsa_circ_0013048 | 1.769979122 | 11.57506864 | 4.992753512 | 0.000202978 | 0.000689923 | 0.160280838 |
| hsa_circ_0015454 | -1.24900548 | 11.88799374 | -4.982465912 | 0.000206866 | 0.000700351 | 0.140778137 |
| hsa_circ_0012077 | 2.027725551 | 12.43891863 | 4.981878251 | 0.00020709 | 0.000700416 | 0.139663722 |
| hsa_circ_0006837 | 2.004868929 | 9.197923168 | 4.979152829 | 0.000208134 | 0.00070268 | 0.134494832 |
| hsa_circ_0001953 | -1.021698429 | 7.085238173 | -4.979057884 | 0.000208171 | 0.00070268 | 0.134314749 |
| hsa_circ_0003892 | 1.859769561 | 13.72211311 | 4.970144143 | 0.000211625 | 0.000711525 | 0.117403513 |
| hsa_circ_0040777 | 1.003752883 | 6.502733661 | 4.966464622 | 0.000213068 | 0.000715601 | 0.110420097 |
| hsa_circ_0087493 | 1.265794495 | 8.742536161 | 4.96275788 | 0.000214532 | 0.000719176 | 0.103383489 |
| hsa_circ_0007196 | 1.697840429 | 12.20345504 | 4.958012266 | 0.000216421 | 0.000724799 | 0.094372526 |
| hsa_circ_0004071 | 1.482867816 | 9.447004235 | 4.952953222 | 0.000218454 | 0.000730892 | 0.08476366 |
| hsa_circ_0048937 | 1.559437939 | 13.5208229 | 4.947687942 | 0.000220592 | 0.0007366 | 0.074760063 |
| hsa_circ_0007798 | -1.616306061 | 10.37292347 | -4.943668036 | 0.000222238 | 0.000739927 | 0.067120503 |
| hsa_circ_0032664 | -2.728087204 | 9.20527852 | -4.938963846 | 0.000224181 | 0.000744944 | 0.058178237 |
| hsa_circ_0002675 | 1.08793773 | 8.810958242 | 4.938406394 | 0.000224412 | 0.000744988 | 0.057118406 |
| hsa_circ_0080251 | -1.478735944 | 6.505786196 | -4.925494157 | 0.000229842 | 0.000760794 | 0.032559994 |
| hsa_circ_0005869 | -2.560697847 | 7.827062536 | -4.906536678 | 0.000238061 | 0.000783446 | -0.003529321 |
| hsa_circ_0007618 | 1.227217173 | 8.574049199 | 4.898242732 | 0.000241753 | 0.000794709 | -0.019330824 |
| hsa_circ_0007933 | -1.287886969 | 5.886111541 | -4.895658808 | 0.000242915 | 0.000796511 | -0.024255207 |
| hsa_circ_0072430 | 1.43748648 | 8.776083372 | 4.889475931 | 0.000245719 | 0.000803999 | -0.036041326 |
| hsa_circ_0006109 | 1.553290138 | 9.277988686 | 4.88272324 | 0.00024882 | 0.000810497 | -0.048918379 |
| hsa_circ_0000253 | -1.465544673 | 14.1437978 | -4.882569272 | 0.000248891 | 0.000810497 | -0.049212047 |
| hsa_circ_0070049 | 1.991445296 | 11.09664964 | 4.868439763 | 0.000255513 | 0.00082969 | -0.076172464 |
| hsa_circ_0002056 | 1.810231857 | 9.080920071 | 4.856455016 | 0.000261274 | 0.000845181 | -0.09905725 |
| hsa_circ_0006088 | 2.232265342 | 11.94093634 | 4.855192923 | 0.000261888 | 0.000845567 | -0.101468101 |
| hsa_circ_0012107 | 1.434049347 | 12.69023232 | 4.853747679 | 0.000262593 | 0.000847043 | -0.104229014 |
| hsa_circ_0043256 | -1.818914214 | 11.11609565 | -4.848140671 | 0.000265349 | 0.000851637 | -0.114942431 |
| hsa_circ_0007113 | -1.774964592 | 7.887258602 | -4.845876986 | 0.000266469 | 0.000853902 | -0.119268644 |
| hsa_circ_0002606 | -1.30281351 | 8.791427602 | -4.84434978 | 0.000267228 | 0.000854793 | -0.12218765 |
| hsa_circ_0067434 | -1.120981724 | 7.426112393 | -4.830520726 | 0.000274204 | 0.000872141 | -0.148630882 |
| hsa_circ_0051260 | 1.03485951 | 10.1679273 | 4.814243916 | 0.000282655 | 0.000895253 | -0.179780374 |
| hsa_circ_0029325 | 1.521646235 | 13.61384693 | 4.813945209 | 0.000282813 | 0.000895253 | -0.18035228 |
| hsa_circ_0020749 | 1.638847847 | 7.466819781 | 4.81351078 | 0.000283042 | 0.000895253 | -0.181184055 |
| hsa_circ_0045905 | 1.092609765 | 7.117430577 | 4.809684209 | 0.000285071 | 0.000900003 | -0.188511414 |
| hsa_circ_0019390 | -1.540908878 | 10.56451485 | -4.809104129 | 0.00028538 | 0.000900146 | -0.189622321 |
| hsa_circ_0003831 | -1.435830204 | 10.88913166 | -4.805603106 | 0.000287251 | 0.000902715 | -0.19632785 |
| hsa_circ_0005651 | -1.461019102 | 8.348200082 | -4.792911448 | 0.000294143 | 0.000920139 | -0.220646917 |
| hsa_circ_0051861 | 1.207790357 | 7.291286791 | 4.790819578 | 0.000295295 | 0.000922054 | -0.224656846 |
| hsa_circ_0031431 | 1.250075878 | 10.28877696 | 4.783906499 | 0.000299136 | 0.000932343 | -0.237911813 |
| hsa_circ_0058189 | 1.347833469 | 8.342104735 | 4.773709102 | 0.000304896 | 0.000946226 | -0.25747302 |
| hsa_circ_0092357 | -1.491777214 | 9.171236138 | -4.760893865 | 0.000312298 | 0.000964567 | -0.282070986 |
| hsa_circ_0082356 | 1.372737444 | 7.365619411 | 4.757779581 | 0.000314125 | 0.000968458 | -0.288051164 |
| hsa_circ_0045038 | -1.837832561 | 6.264223087 | -4.755716194 | 0.000315342 | 0.000971332 | -0.292013908 |
| hsa_circ_0008529 | -1.426310592 | 10.29167331 | -4.738015554 | 0.000325979 | 0.000999591 | -0.326025738 |
| hsa_circ_0024731 | 1.206868265 | 8.189989143 | 4.73350847 | 0.000328746 | 0.00100627 | -0.334691146 |
| hsa_circ_0079385 | -1.406599878 | 13.09098396 | -4.732422156 | 0.000329417 | 0.00100742 | -0.336780018 |
| hsa_circ_0007991 | 3.092749755 | 11.62524258 | 4.726385081 | 0.00033317 | 0.001017076 | -0.348390856 |
| hsa_circ_0006944 | -1.553417398 | 13.62276733 | -4.721039126 | 0.00033653 | 0.001026416 | -0.358675526 |
| hsa_circ_0087072 | -1.764730316 | 6.068435842 | -4.716329763 | 0.000339519 | 0.001032766 | -0.367737859 |
| hsa_circ_0041050 | 1.602126224 | 7.832212531 | 4.714454556 | 0.000340717 | 0.001035123 | -0.371346973 |
| hsa_circ_0001828 | 1.541872653 | 11.77830083 | 4.714168697 | 0.0003409 | 0.001035123 | -0.371897182 |
| hsa_circ_0001806 | 2.172266541 | 13.88573121 | 4.713460607 | 0.000341353 | 0.00103558 | -0.373260118 |
| hsa_circ_0003141 | 1.354627949 | 14.33265313 | 4.711837907 | 0.000342395 | 0.00103782 | -0.376383685 |
| hsa_circ_0001360 | 2.24803852 | 11.75739346 | 4.707360492 | 0.000345288 | 0.001044733 | -0.385003701 |
| hsa_circ_0008832 | -1.734407408 | 8.640897684 | -4.705408526 | 0.000346557 | 0.001047644 | -0.388762287 |
| hsa_circ_0006446 | -1.289418357 | 14.62637311 | -4.672499414 | 0.00036869 | 0.00110284 | -0.452186182 |
| hsa_circ_0003520 | -1.333708806 | 8.911743321 | -4.669177924 | 0.000371003 | 0.00110879 | -0.458593346 |
| hsa_circ_0001184 | -1.218642112 | 14.34176902 | -4.660799186 | 0.000376906 | 0.001122504 | -0.474760674 |
| hsa_circ_0055377 | 1.503915255 | 7.753518464 | 4.652077141 | 0.000383154 | 0.001137146 | -0.491597579 |
| hsa_circ_0003958 | 1.451141102 | 11.90996714 | 4.610297123 | 0.000414601 | 0.001225154 | -0.572348874 |
| hsa_circ_0000069 | 2.547995255 | 11.66381009 | 4.602661267 | 0.000420631 | 0.001240825 | -0.587124863 |
| hsa_circ_0031851 | -1.014438602 | 8.224095026 | -4.59827473 | 0.000424135 | 0.001250083 | -0.595615593 |
| hsa_circ_0005830 | -1.806302827 | 8.367335383 | -4.591696288 | 0.000429448 | 0.001260304 | -0.60835236 |
| hsa_circ_0064136 | 1.507582571 | 8.138501663 | 4.572888673 | 0.000445018 | 0.001303239 | -0.644788246 |
| hsa_circ_0004788 | 1.483487173 | 10.54487398 | 4.572645941 | 0.000445223 | 0.001303239 | -0.645258699 |
| hsa_circ_0027774 | 1.60901201 | 10.47675348 | 4.556092145 | 0.000459415 | 0.001337904 | -0.677355043 |
| hsa_circ_0013062 | 2.270715571 | 8.59652252 | 4.545098153 | 0.000469098 | 0.001360872 | -0.698684922 |
| hsa_circ_0054302 | 2.9779085 | 9.869190709 | 4.54404944 | 0.000470033 | 0.001361859 | -0.700720131 |
| hsa_circ_0002820 | -1.168273541 | 5.929094964 | -4.534104265 | 0.000478991 | 0.001384294 | -0.720025272 |
| hsa_circ_0084429 | 1.759752607 | 8.086240314 | 4.529115985 | 0.000483551 | 0.001392497 | -0.729711567 |
| hsa_circ_0006958 | -1.122576694 | 11.23863441 | -4.528771001 | 0.000483868 | 0.001392497 | -0.730381543 |
| hsa_circ_0070649 | -1.279193918 | 6.06468702 | -4.517711951 | 0.000494144 | 0.001420873 | -0.751864182 |
| hsa_circ_0005418 | -2.067427658 | 6.390428712 | -4.507887078 | 0.000503462 | 0.001442808 | -0.770958249 |
| hsa_circ_0004656 | -1.654150643 | 6.086709077 | -4.506810395 | 0.000504494 | 0.001444553 | -0.773051223 |
| hsa_circ_0000727 | 1.238598786 | 7.81451324 | 4.503093178 | 0.000508074 | 0.001451155 | -0.780277919 |
| hsa_circ_0004789 | -1.710703459 | 9.566804719 | -4.497761687 | 0.000513255 | 0.001459848 | -0.790645009 |
| hsa_circ_0074269 | 1.085454816 | 8.504238224 | 4.497085806 | 0.000513915 | 0.001460511 | -0.791959433 |
| hsa_circ_0009581 | 2.478608133 | 10.27061827 | 4.495383492 | 0.000515583 | 0.001464033 | -0.795270191 |
| hsa_circ_0004099 | -2.152788286 | 10.50878451 | -4.490862255 | 0.00052004 | 0.001473424 | -0.804064544 |
| hsa_circ_0008664 | -1.194284735 | 6.723822265 | -4.485916322 | 0.00052496 | 0.00148449 | -0.813686959 |
| hsa_circ_0000559 | 1.836956194 | 8.803630566 | 4.484828142 | 0.000526049 | 0.001486339 | -0.815804312 |
| hsa_circ_0002702 | 2.13013152 | 9.839240362 | 4.482625104 | 0.000528261 | 0.001491026 | -0.820091232 |
| hsa_circ_0009792 | 1.217761378 | 9.125458821 | 4.475971144 | 0.000535 | 0.001506645 | -0.833041727 |
| hsa_circ_0092367 | -1.501533357 | 12.59889532 | -4.468557533 | 0.000542613 | 0.001525569 | -0.847475067 |
| hsa_circ_0005336 | 1.169567347 | 8.393916643 | 4.458281168 | 0.00055335 | 0.001552067 | -0.867489338 |
| hsa_circ_0040994 | -1.394748071 | 8.636479393 | -4.45723852 | 0.000554452 | 0.001553739 | -0.869520488 |
| hsa_circ_0008654 | 1.273043546 | 7.857489901 | 4.443666156 | 0.000568999 | 0.001591894 | -0.895968475 |
| hsa_circ_0005252 | -1.454247429 | 10.98986774 | -4.441564412 | 0.000571287 | 0.00159568 | -0.900065408 |
| hsa_circ_0005273 | 1.931592566 | 11.17231388 | 4.438942074 | 0.000574155 | 0.001599765 | -0.905177636 |
| hsa_circ_0004228 | 1.052380071 | 7.882712393 | 4.435312821 | 0.000578148 | 0.001609578 | -0.912253751 |
| hsa_circ_0000357 | 2.345245867 | 9.860708138 | 4.425387231 | 0.000589215 | 0.001636388 | -0.931611506 |
| hsa_circ_0000053 | 1.298952408 | 7.549911327 | 4.418316623 | 0.000597231 | 0.001653276 | -0.945406009 |
| hsa_circ_0046292 | 1.719956837 | 8.271337276 | 4.415680032 | 0.000600249 | 0.001659832 | -0.950550916 |
| hsa_circ_0053907 | 1.422185878 | 8.767665745 | 4.411980125 | 0.000604511 | 0.001668021 | -0.957771647 |
| hsa_circ_0006030 | -2.077801204 | 7.916681347 | -4.405372045 | 0.0006122 | 0.001683797 | -0.970670634 |
| hsa_circ_0010433 | -1.394539867 | 6.368036597 | -4.396720391 | 0.000622419 | 0.001709151 | -0.987563826 |
| hsa_circ_0092297 | 1.694183781 | 8.594246355 | 4.39568972 | 0.000623648 | 0.001709777 | -0.989576697 |
| hsa_circ_0003045 | -1.29467401 | 9.584162077 | -4.386782484 | 0.000634375 | 0.001733619 | -1.006975689 |
| hsa_circ_0003832 | 1.205618235 | 8.852622668 | 4.373718117 | 0.000650451 | 0.001770471 | -1.032505997 |
| hsa_circ_0007061 | 1.043098071 | 10.14357182 | 4.367167701 | 0.000658669 | 0.001788564 | -1.045311641 |
| hsa_circ_0003541 | -1.715473571 | 6.288771173 | -4.353725975 | 0.000675869 | 0.001826556 | -1.071599397 |
| hsa_circ_0031897 | -1.297476255 | 10.74840587 | -4.348432097 | 0.000682768 | 0.001839382 | -1.081956216 |
| hsa_circ_0008342 | 1.237080133 | 7.634051964 | 4.341954308 | 0.00069131 | 0.001857998 | -1.094632007 |
| hsa_circ_0078522 | 1.999025128 | 9.956891946 | 4.341954275 | 0.00069131 | 0.001857998 | -1.094632072 |
| hsa_circ_0004291 | 1.40384802 | 8.918632724 | 4.331010736 | 0.00070599 | 0.00188706 | -1.116053412 |
| hsa_circ_0000714 | -1.036216122 | 13.0943944 | -4.32057022 | 0.000720295 | 0.001917791 | -1.136498143 |
| hsa_circ_0008667 | -1.397978571 | 7.411820306 | -4.318566768 | 0.000723073 | 0.00192369 | -1.140422213 |
| hsa_circ_0060558 | 1.505004235 | 9.39594775 | 4.315137862 | 0.000727855 | 0.001932753 | -1.147138914 |
| hsa_circ_0044468 | -1.081763102 | 7.858952459 | -4.31248008 | 0.000731583 | 0.001938734 | -1.152345673 |
| hsa_circ_0076125 | -1.547842694 | 7.91981198 | -4.312281509 | 0.000731863 | 0.001938734 | -1.152734705 |
| hsa_circ_0005918 | -1.577650663 | 13.71970036 | -4.310645022 | 0.000734169 | 0.001941115 | -1.155940955 |
| hsa_circ_0000723 | 1.232466398 | 7.721080495 | 4.287923159 | 0.000766974 | 0.002016924 | -1.200477481 |
| hsa_circ_0009065 | 1.667515714 | 8.847527541 | 4.281701637 | 0.000776217 | 0.00203631 | -1.212678307 |
| hsa_circ_0049997 | 1.576165582 | 13.55215295 | 4.272867473 | 0.000789537 | 0.002065129 | -1.230007192 |
| hsa_circ_0059369 | -3.015493255 | 9.342516556 | -4.266828761 | 0.000798777 | 0.00208451 | -1.241855592 |
| hsa_circ_0082141 | 1.152027684 | 7.832294577 | 4.265654909 | 0.000800586 | 0.002087636 | -1.244159059 |
| hsa_circ_0034972 | -1.019260439 | 12.28554986 | -4.263463515 | 0.000803975 | 0.002094873 | -1.248459505 |
| hsa_circ_0005556 | -1.501921163 | 14.00939941 | -4.256119232 | 0.000815438 | 0.002115063 | -1.262874416 |
| hsa_circ_0009098 | -2.014718454 | 6.748347742 | -4.253206606 | 0.000820031 | 0.002125361 | -1.268592119 |
| hsa_circ_0055054 | 1.315496449 | 7.684222561 | 4.251922433 | 0.000822064 | 0.002128756 | -1.271113221 |
| hsa_circ_0040625 | -1.781362449 | 7.168244357 | -4.249936741 | 0.000825219 | 0.002132335 | -1.275011767 |
| hsa_circ_0041506 | 1.054507643 | 8.140078464 | 4.241563105 | 0.000838657 | 0.002165422 | -1.291454675 |
| hsa_circ_0092301 | -1.64414798 | 6.610999005 | -4.228987835 | 0.000859262 | 0.002208607 | -1.316156549 |
| hsa_circ_0072008 | 1.873696163 | 8.131080102 | 4.226317303 | 0.000863704 | 0.002215025 | -1.321403618 |
| hsa_circ_0052455 | 1.40557451 | 8.765780051 | 4.222495059 | 0.000870103 | 0.002229762 | -1.328914351 |
| hsa_circ_0046572 | -1.19029149 | 6.384911143 | -4.205205297 | 0.000899661 | 0.002294567 | -1.362900122 |
| hsa_circ_0085154 | -1.988861051 | 7.527439311 | -4.192871771 | 0.000921374 | 0.002340083 | -1.387154726 |
| hsa_circ_0001516 | -1.448470755 | 7.713713214 | -4.190702524 | 0.000925248 | 0.002346433 | -1.391421618 |
| hsa_circ_0001221 | -1.72264901 | 10.21389361 | -4.187021571 | 0.000931859 | 0.002359698 | -1.398662661 |
| hsa_circ_0003930 | -1.145248214 | 11.05848356 | -4.180504215 | 0.000943685 | 0.002382579 | -1.411485329 |
| hsa_circ_0001568 | -1.176944561 | 15.16015538 | -4.1715414 | 0.0009602 | 0.002418911 | -1.429123388 |
| hsa_circ_0005348 | -1.373177622 | 6.292687372 | -4.168711506 | 0.000965476 | 0.002430409 | -1.43469334 |
| hsa_circ_0058427 | -1.641502673 | 5.997347122 | -4.154910897 | 0.000991632 | 0.002483442 | -1.46186296 |
| hsa_circ_0004673 | -1.805029944 | 6.827401166 | -4.115782057 | 0.001069809 | 0.002663607 | -1.538953747 |
| hsa_circ_0011159 | -1.039669827 | 8.187181995 | -4.115297447 | 0.001070816 | 0.002664172 | -1.539909025 |
| hsa_circ_0075504 | -1.57309002 | 13.45554663 | -4.102605685 | 0.001097532 | 0.002720732 | -1.564931685 |
| hsa_circ_0000851 | -1.497077592 | 8.810524551 | -4.10046319 | 0.001102109 | 0.002726143 | -1.569156564 |
| hsa_circ_0003757 | -2.075710255 | 7.353495801 | -4.090557381 | 0.001123524 | 0.00276909 | -1.588693237 |
| hsa_circ_0078279 | -1.533671515 | 8.309641982 | -4.069183974 | 0.001171193 | 0.002878271 | -1.630863094 |
| hsa_circ_0001942 | -1.310677153 | 7.679075699 | -4.066299473 | 0.001177783 | 0.002892384 | -1.636555894 |
| hsa_circ_0034326 | 1.846081949 | 9.980802556 | 4.061316158 | 0.001189257 | 0.002914277 | -1.646391785 |
| hsa_circ_0008160 | -1.208326398 | 7.098865179 | -4.043848328 | 0.001230387 | 0.002997861 | -1.680878103 |
| hsa_circ_0017069 | -1.033709531 | 6.728177051 | -4.04069185 | 0.001237973 | 0.003012047 | -1.687111313 |
| hsa_circ_0036751 | -1.602581296 | 8.63745974 | -4.03560202 | 0.001250306 | 0.003037727 | -1.6971633 |
| hsa_circ_0065301 | -1.618921122 | 6.588957439 | -4.03276899 | 0.001257225 | 0.003049639 | -1.702758782 |
| hsa_circ_0001837 | -1.402537092 | 7.34443173 | -4.028409 | 0.001267949 | 0.003067018 | -1.711370814 |
| hsa_circ_0049392 | 1.081073092 | 8.649303362 | 4.027763812 | 0.001269544 | 0.003067018 | -1.712645285 |
| hsa_circ_0046557 | 1.537426367 | 9.073277337 | 4.026323214 | 0.001273113 | 0.003073465 | -1.715491031 |
| hsa_circ_0001796 | -1.298382342 | 7.708813028 | -4.021100069 | 0.001286138 | 0.00309834 | -1.72580952 |
| hsa_circ_0005687 | 1.650701112 | 9.580249638 | 4.017143275 | 0.001296095 | 0.003115736 | -1.733627047 |
| hsa_circ_0000231 | 1.39314099 | 7.740404485 | 4.016394965 | 0.001297987 | 0.00311809 | -1.735105573 |
| hsa_circ_0049998 | 1.746532704 | 8.914783332 | 4.015673435 | 0.001299814 | 0.003120284 | -1.736531209 |
| hsa_circ_0056548 | -1.675553459 | 8.833864138 | -4.011557099 | 0.001310287 | 0.003141011 | -1.744664876 |
| hsa_circ_0016404 | 2.004281776 | 12.38185706 | 4.007192723 | 0.001321486 | 0.003163416 | -1.753289408 |
| hsa_circ_0086563 | 1.144829776 | 7.139704301 | 4.000279411 | 0.001339424 | 0.003199965 | -1.766952499 |
| hsa_circ_0036627 | 1.011065969 | 7.336910781 | 4.000226026 | 0.001339564 | 0.003199965 | -1.767058016 |
| hsa_circ_0038718 | 2.189652786 | 13.22559851 | 3.973165371 | 0.001412213 | 0.003354319 | -1.820556932 |
| hsa_circ_0000688 | 1.04431902 | 8.036278459 | 3.951552653 | 0.001473116 | 0.003470457 | -1.863304358 |
| hsa_circ_0007888 | -1.26761148 | 10.57338549 | -3.950320636 | 0.001476667 | 0.003476426 | -1.865741626 |
| hsa_circ_0065220 | -1.252221857 | 6.72379951 | -3.94424946 | 0.001494295 | 0.003508256 | -1.877752818 |
| hsa_circ_0043785 | -1.038424429 | 8.475909071 | -3.934814816 | 0.001522116 | 0.003566221 | -1.89642066 |
| hsa_circ_0005203 | 1.174494592 | 7.300292673 | 3.929658967 | 0.001537543 | 0.003599894 | -1.906623463 |
| hsa_circ_0034325 | 1.884533602 | 8.612973903 | 3.920691351 | 0.001564754 | 0.003650284 | -1.92437124 |
| hsa_circ_0007733 | 1.411349684 | 8.583476852 | 3.918294355 | 0.001572111 | 0.003660756 | -1.929115536 |
| hsa_circ_0002981 | -1.175283969 | 10.87834442 | -3.899867805 | 0.00162985 | 0.003777183 | -1.965592135 |
| hsa_circ_0034786 | 1.580120776 | 10.62908097 | 3.890852312 | 0.001658882 | 0.003824055 | -1.983442328 |
| hsa_circ_0006896 | 2.535333719 | 12.51448319 | 3.874967552 | 0.001711323 | 0.003931324 | -2.014898408 |
| hsa_circ_0001120 | -1.082026276 | 6.850373668 | -3.857361324 | 0.001771421 | 0.004050317 | -2.049770535 |
| hsa_circ_0022033 | -1.064647061 | 5.937701571 | -3.855376785 | 0.001778328 | 0.004063391 | -2.053701689 |
| hsa_circ_0008193 | -2.340242036 | 9.016393941 | -3.855015973 | 0.001779587 | 0.004063549 | -2.054416428 |
| hsa_circ_0003270 | 1.548463276 | 10.3848692 | 3.853801191 | 0.001783832 | 0.004070521 | -2.056822829 |
| hsa_circ_0042521 | 1.372587031 | 8.337379077 | 3.851750798 | 0.00179102 | 0.004084195 | -2.060884592 |
| hsa_circ_0007217 | -1.022178959 | 6.493782383 | -3.85039552 | 0.001795787 | 0.004092334 | -2.063569403 |
| hsa_circ_0092348 | -1.326949429 | 9.231466214 | -3.847767459 | 0.001805068 | 0.004108004 | -2.068775709 |
| hsa_circ_0005514 | -1.90032099 | 6.905007934 | -3.844873036 | 0.001815347 | 0.004128645 | -2.074509857 |
| hsa_circ_0062545 | -1.366534362 | 6.586807074 | -3.834620533 | 0.001852236 | 0.004201353 | -2.094822454 |
| hsa_circ_0007112 | -1.244658893 | 6.582252441 | -3.812319119 | 0.001935131 | 0.004360427 | -2.139013412 |
| hsa_circ_0020246 | 1.166497357 | 8.208737495 | 3.807995726 | 0.001951633 | 0.004394711 | -2.147581313 |
| hsa_circ_0000848 | -1.384538959 | 9.822906214 | -3.806629551 | 0.001956877 | 0.004401532 | -2.150288795 |
| hsa_circ_0092337 | 2.484700439 | 11.9441862 | 3.806535427 | 0.001957239 | 0.004401532 | -2.150475332 |
| hsa_circ_0022587 | 1.638594439 | 8.931205128 | 3.796037926 | 0.001998022 | 0.004478505 | -2.171280193 |
| hsa_circ_0002003 | 1.245845418 | 11.18221069 | 3.793848635 | 0.002006635 | 0.004494863 | -2.175619317 |
| hsa_circ_0080712 | 1.454684439 | 12.50002181 | 3.789368798 | 0.002024379 | 0.004531637 | -2.184498446 |
| hsa_circ_0008253 | -1.571445612 | 9.113670449 | -3.781658014 | 0.002055293 | 0.004577608 | -2.199781977 |
| hsa_circ_0040719 | -1.3745195 | 7.634174148 | -3.781180265 | 0.002057224 | 0.004577608 | -2.200728946 |
| hsa_circ_0001336 | 1.227325837 | 6.081419133 | 3.780910551 | 0.002058315 | 0.004577608 | -2.201263562 |
| hsa_circ_0000370 | -1.010035939 | 8.127498776 | -3.778640958 | 0.002067519 | 0.004590514 | -2.205762274 |
| hsa_circ_0069340 | -1.091973296 | 6.009553577 | -3.778485755 | 0.00206815 | 0.004590514 | -2.206069914 |
| hsa_circ_0078768 | 1.106523602 | 7.851996189 | 3.773504353 | 0.002088505 | 0.004629677 | -2.215944094 |
| hsa_circ_0087104 | -1.879384107 | 8.205313462 | -3.764801897 | 0.002124553 | 0.004697394 | -2.233194804 |
| hsa_circ_0092325 | -1.192989643 | 6.93417026 | -3.752844634 | 0.002175116 | 0.00479677 | -2.256898604 |
| hsa_circ_0023696 | 1.193807112 | 7.854268342 | 3.749909456 | 0.002187714 | 0.004815225 | -2.262717411 |
| hsa_circ_0070396 | 1.283504806 | 8.185962526 | 3.739481361 | 0.002233071 | 0.004905577 | -2.283390945 |
| hsa_circ_0051732 | 2.064620469 | 13.22535754 | 3.734691433 | 0.002254224 | 0.00493301 | -2.292887127 |
| hsa_circ_0076767 | -1.127270745 | 9.747486015 | -3.708211043 | 0.002374884 | 0.005163974 | -2.34538704 |
| hsa_circ_0008309 | -1.051863806 | 9.433342138 | -3.699906357 | 0.002414057 | 0.005245814 | -2.361852201 |
| hsa_circ_0005456 | -1.170383117 | 6.169418768 | -3.683498524 | 0.002493388 | 0.005401024 | -2.394382899 |
| hsa_circ_0069248 | -1.338889602 | 8.769194536 | -3.679603987 | 0.002512603 | 0.005435752 | -2.402104267 |
| hsa_circ_0008773 | 1.098156704 | 8.885605332 | 3.663089033 | 0.002595763 | 0.005608556 | -2.434846484 |
| hsa_circ_0080000 | -1.155041286 | 9.400813918 | -3.657753917 | 0.002623219 | 0.005653575 | -2.445423501 |
| hsa_circ_0008784 | 1.642612612 | 8.526692041 | 3.642836812 | 0.002701556 | 0.005804097 | -2.474996077 |
| hsa_circ_0002637 | -1.131728459 | 7.184307964 | -3.637072532 | 0.002732457 | 0.005852083 | -2.486423064 |
| hsa_circ_0000683 | -1.833862888 | 8.062986311 | -3.628697085 | 0.002777994 | 0.005927313 | -2.503025815 |
| hsa_circ_0004552 | -1.114953786 | 14.33757006 | -3.62072134 | 0.002822071 | 0.006002612 | -2.51883554 |
| hsa_circ_0004166 | 1.168978643 | 8.669829893 | 3.619252102 | 0.002830268 | 0.0060163 | -2.521747823 |
| hsa_circ_0063604 | -1.240690337 | 6.380432638 | -3.61576825 | 0.002849799 | 0.006047361 | -2.52865331 |
| hsa_circ_0002224 | 2.094544653 | 10.53476097 | 3.613936269 | 0.002860124 | 0.006064671 | -2.532284495 |
| hsa_circ_0082139 | 1.17000323 | 8.662726008 | 3.612785445 | 0.00286663 | 0.006074695 | -2.53456553 |
| hsa_circ_0001888 | -1.435801602 | 9.536438474 | -3.605601169 | 0.002907581 | 0.006146224 | -2.548805002 |
| hsa_circ_0009910 | 1.585596408 | 13.08155264 | 3.601997959 | 0.002928342 | 0.006186281 | -2.555946411 |
| hsa_circ_0007762 | -1.589147128 | 7.236110217 | -3.599544489 | 0.002942564 | 0.006208647 | -2.560808975 |
| hsa_circ_0001489 | 1.435307827 | 14.32901944 | 3.589582733 | 0.00300103 | 0.006317252 | -2.580551345 |
| hsa_circ_0001789 | -1.507525918 | 11.25565998 | -3.572207566 | 0.003105826 | 0.006485026 | -2.614981659 |
| hsa_circ_0065217 | -1.005615786 | 7.078110719 | -3.567947133 | 0.003132082 | 0.006531863 | -2.623423186 |
| hsa_circ_0003110 | 1.353581918 | 9.94123001 | 3.56326095 | 0.00316122 | 0.00658459 | -2.632707861 |
| hsa_circ_0000422 | -1.141054071 | 8.209966321 | -3.556410569 | 0.003204308 | 0.00665001 | -2.646279597 |
| hsa_circ_0006135 | -1.187061939 | 9.455715735 | -3.55267098 | 0.003228079 | 0.006691167 | -2.653687907 |
| hsa_circ_0001293 | -1.062289306 | 7.048529429 | -3.552367467 | 0.003230016 | 0.006691167 | -2.654289167 |
| hsa_circ_0046523 | 1.00787901 | 6.460780781 | 3.542417549 | 0.003294172 | 0.006803441 | -2.6739988 |
| hsa_circ_0086419 | -1.062817821 | 7.521569273 | -3.541869241 | 0.003297745 | 0.006806704 | -2.675084866 |
| hsa_circ_0009524 | -1.949701847 | 8.395664005 | -3.452393647 | 0.003936283 | 0.007932945 | -2.852195416 |
| hsa_circ_0006629 | 1.17706651 | 9.060500041 | 3.442696313 | 0.00401257 | 0.008058165 | -2.871373642 |
| hsa_circ_0067934 | 2.543754704 | 13.20194572 | 3.433995254 | 0.004082284 | 0.008158083 | -2.888578225 |
| hsa_circ_0001212 | 1.239638735 | 9.475348112 | 3.405799917 | 0.004316659 | 0.008583067 | -2.944305716 |
| hsa_circ_0008662 | -1.057869918 | 5.871960847 | -3.404393761 | 0.004328696 | 0.008601992 | -2.947083983 |
| hsa_circ_0082182 | 2.169051321 | 10.70936766 | 3.399143203 | 0.004373938 | 0.008681796 | -2.957457119 |
| hsa_circ_0003568 | -1.164660816 | 8.10543798 | -3.385454917 | 0.004494128 | 0.00888937 | -2.984493553 |
| hsa_circ_0084666 | -1.056406934 | 6.447371013 | -3.347291024 | 0.004847013 | 0.009488479 | -3.059819942 |
| hsa_circ_0046555 | 1.098021 | 8.534636439 | 3.326598124 | 0.005049827 | 0.009834782 | -3.100627322 |
