# Supplementary material for: Up-Regulation of hsa_circ_0000517 Predicts Adverse Prognosis of Hepatocellular Carcinoma
Source: Front Oncol. 2019 Oct 22;9:1105. doi: 10.3389/fonc.2019.01105 (PMC6842961; doi:10.3389/fonc.2019.01105)
Supplement: Supplementary file 4 [file Table_4.DOCX]

**Table S4 701 hub genes in tuiquoise module.**

| gene | R | P.value |
| --- | --- | --- |
| hsa_circ_0007018 | 0.995501053 | 5.16E-24 |
| hsa_circ_0074736 | 0.993432272 | 3.28E-22 |
| hsa_circ_0027478 | 0.992733682 | 9.93E-22 |
| hsa_circ_0062022 | 0.98926381 | 7.16E-20 |
| hsa_circ_0032704 | 0.987912746 | 2.62E-19 |
| hsa_circ_0008274 | 0.987881179 | 2.70E-19 |
| hsa_circ_0048607 | 0.985273283 | 2.27E-18 |
| hsa_circ_0070348 | 0.985263428 | 2.29E-18 |
| hsa_circ_0002702 | 0.983139936 | 9.97E-18 |
| hsa_circ_0070049 | 0.983023354 | 1.08E-17 |
| hsa_circ_0001955 | 0.982612415 | 1.40E-17 |
| hsa_circ_0004780 | 0.982068607 | 1.95E-17 |
| hsa_circ_0069104 | 0.981627868 | 2.55E-17 |
| hsa_circ_0004788 | 0.981041972 | 3.59E-17 |
| hsa_circ_0088777 | 0.980684155 | 4.40E-17 |
| hsa_circ_0007769 | 0.979730914 | 7.44E-17 |
| hsa_circ_0034326 | 0.979546808 | 8.21E-17 |
| hsa_circ_0003923 | 0.979103141 | 1.04E-16 |
| hsa_circ_0012077 | 0.97849953 | 1.42E-16 |
| hsa_circ_0007196 | 0.978185457 | 1.66E-16 |
| hsa_circ_0005397 | 0.977665948 | 2.14E-16 |
| hsa_circ_0072088 | 0.977645249 | 2.17E-16 |
| hsa_circ_0003528 | 0.977068444 | 2.86E-16 |
| hsa_circ_0008383 | 0.976674905 | 3.44E-16 |
| hsa_circ_0002056 | 0.976160757 | 4.36E-16 |
| hsa_circ_0088807 | 0.974940105 | 7.51E-16 |
| hsa_circ_0011385 | 0.974067819 | 1.09E-15 |
| hsa_circ_0026652 | 0.972913232 | 1.75E-15 |
| hsa_circ_0013062 | 0.972583897 | 2.00E-15 |
| hsa_circ_0007991 | 0.972348286 | 2.19E-15 |
| hsa_circ_0000560 | 0.97224065 | 2.29E-15 |
| hsa_circ_0051220 | 0.972004514 | 2.51E-15 |
| hsa_circ_0017636 | 0.971900268 | 2.61E-15 |
| hsa_circ_0089131 | 0.971685895 | 2.83E-15 |
| hsa_circ_0006553 | 0.970818169 | 3.93E-15 |
| hsa_circ_0028198 | 0.969875691 | 5.56E-15 |
| hsa_circ_0006088 | 0.969843393 | 5.62E-15 |
| hsa_circ_0009006 | 0.96978506 | 5.74E-15 |
| hsa_circ_0052523 | 0.96926718 | 6.91E-15 |
| hsa_circ_0003763 | 0.968648413 | 8.57E-15 |
| hsa_circ_0062389 | 0.96854285 | 8.89E-15 |
| hsa_circ_0052455 | 0.968315623 | 9.61E-15 |
| hsa_circ_0003098 | 0.968028491 | 1.06E-14 |
| hsa_circ_0001892 | 0.967496656 | 1.27E-14 |
| hsa_circ_0001535 | 0.966702682 | 1.65E-14 |
| hsa_circ_0040148 | 0.966045343 | 2.04E-14 |
| hsa_circ_0000673 | 0.965623155 | 2.33E-14 |
| hsa_circ_0041050 | 0.964530995 | 3.27E-14 |
| hsa_circ_0009792 | 0.963371756 | 4.63E-14 |
| hsa_circ_0017454 | 0.963267064 | 4.78E-14 |
| hsa_circ_0000520 | 0.962688527 | 5.66E-14 |
| hsa_circ_0000069 | 0.961612057 | 7.70E-14 |
| hsa_circ_0092232 | 0.959291883 | 1.45E-13 |
| hsa_circ_0021087 | 0.958360128 | 1.85E-13 |
| hsa_circ_0001806 | 0.958349597 | 1.86E-13 |
| hsa_circ_0004976 | 0.95772481 | 2.18E-13 |
| hsa_circ_0004315 | 0.957069258 | 2.58E-13 |
| hsa_circ_0005835 | 0.956183528 | 3.21E-13 |
| hsa_circ_0051732 | 0.956104752 | 3.28E-13 |
| hsa_circ_0092283 | 0.955981622 | 3.38E-13 |
| hsa_circ_0078522 | 0.955833558 | 3.50E-13 |
| hsa_circ_0038718 | 0.955738615 | 3.58E-13 |
| hsa_circ_0005785 | 0.955281609 | 4.00E-13 |
| hsa_circ_0043001 | 0.955225245 | 4.06E-13 |
| hsa_circ_0068850 | 0.953707636 | 5.82E-13 |
| hsa_circ_0000255 | 0.951802609 | 8.98E-13 |
| hsa_circ_0016867 | 0.950191407 | 1.28E-12 |
| hsa_circ_0007733 | 0.949467669 | 1.50E-12 |
| hsa_circ_0049997 | 0.948999663 | 1.65E-12 |
| hsa_circ_0036005 | 0.948761317 | 1.74E-12 |
| hsa_circ_0055377 | 0.948643027 | 1.78E-12 |
| hsa_circ_0008342 | 0.948380231 | 1.88E-12 |
| hsa_circ_0038821 | 0.947962634 | 2.05E-12 |
| hsa_circ_0074817 | 0.947255643 | 2.37E-12 |
| hsa_circ_0001828 | 0.947129999 | 2.43E-12 |
| hsa_circ_0000231 | 0.946684462 | 2.66E-12 |
| hsa_circ_0001834 | 0.946103582 | 2.99E-12 |
| hsa_circ_0078155 | 0.945902889 | 3.11E-12 |
| hsa_circ_0001513 | 0.944981706 | 3.73E-12 |
| hsa_circ_0048937 | 0.94483709 | 3.84E-12 |
| hsa_circ_0078150 | 0.943604873 | 4.87E-12 |
| hsa_circ_0004004 | 0.942433285 | 6.07E-12 |
| hsa_circ_0031431 | 0.941819671 | 6.80E-12 |
| hsa_circ_0004599 | 0.941735473 | 6.90E-12 |
| hsa_circ_0002675 | 0.940372038 | 8.85E-12 |
| hsa_circ_0001348 | 0.940271942 | 9.01E-12 |
| hsa_circ_0072255 | 0.939923696 | 9.59E-12 |
| hsa_circ_0031570 | 0.939510433 | 1.03E-11 |
| hsa_circ_0000956 | 0.939463885 | 1.04E-11 |
| hsa_circ_0082182 | 0.939159454 | 1.10E-11 |
| hsa_circ_0002454 | 0.939019162 | 1.13E-11 |
| hsa_circ_0003839 | 0.938500911 | 1.23E-11 |
| hsa_circ_0029325 | 0.938387562 | 1.26E-11 |
| hsa_circ_0092273 | 0.93794966 | 1.36E-11 |
| hsa_circ_0000516 | 0.937147659 | 1.56E-11 |
| hsa_circ_0006276 | 0.93658927 | 1.71E-11 |
| hsa_circ_0008501 | 0.936213137 | 1.82E-11 |
| hsa_circ_0071865 | 0.936057448 | 1.87E-11 |
| hsa_circ_0092333 | 0.935952559 | 1.90E-11 |
| hsa_circ_0009910 | 0.935754816 | 1.97E-11 |
| hsa_circ_0000257 | 0.935327927 | 2.11E-11 |
| hsa_circ_0016404 | 0.935229118 | 2.15E-11 |
| hsa_circ_0006896 | 0.935071996 | 2.20E-11 |
| hsa_circ_0074854 | 0.934469411 | 2.43E-11 |
| hsa_circ_0041992 | 0.933937743 | 2.65E-11 |
| hsa_circ_0041506 | 0.93372813 | 2.74E-11 |
| hsa_circ_0006913 | 0.933382892 | 2.90E-11 |
| hsa_circ_0024604 | 0.932760012 | 3.20E-11 |
| hsa_circ_0008301 | 0.931676727 | 3.80E-11 |
| hsa_circ_0009150 | 0.931468056 | 3.92E-11 |
| hsa_circ_0028135 | 0.931224467 | 4.07E-11 |
| hsa_circ_0012107 | 0.929859544 | 5.02E-11 |
| hsa_circ_0000554 | 0.929696746 | 5.15E-11 |
| hsa_circ_0042819 | 0.929258915 | 5.50E-11 |
| hsa_circ_0044158 | 0.929085265 | 5.65E-11 |
| hsa_circ_0003222 | 0.928913735 | 5.80E-11 |
| hsa_circ_0003945 | 0.927955291 | 6.68E-11 |
| hsa_circ_0008472 | 0.927096062 | 7.58E-11 |
| hsa_circ_0070190 | 0.926650262 | 8.09E-11 |
| hsa_circ_0083766 | 0.925959782 | 8.94E-11 |
| hsa_circ_0015928 | 0.924797748 | 1.06E-10 |
| hsa_circ_0054302 | 0.924745335 | 1.06E-10 |
| hsa_circ_0007713 | 0.924459529 | 1.11E-10 |
| hsa_circ_0000357 | 0.924442708 | 1.11E-10 |
| hsa_circ_0019627 | 0.923681734 | 1.23E-10 |
| hsa_circ_0004587 | 0.92347877 | 1.27E-10 |
| hsa_circ_0007246 | 0.923470564 | 1.27E-10 |
| hsa_circ_0006837 | 0.923323624 | 1.30E-10 |
| hsa_circ_0092316 | 0.923169868 | 1.33E-10 |
| hsa_circ_0046580 | 0.923106221 | 1.34E-10 |
| hsa_circ_0001901 | 0.92264206 | 1.43E-10 |
| hsa_circ_0073271 | 0.92261297 | 1.43E-10 |
| hsa_circ_0055054 | 0.922362026 | 1.48E-10 |
| hsa_circ_0000235 | 0.921553933 | 1.65E-10 |
| hsa_circ_0080712 | 0.921514739 | 1.66E-10 |
| hsa_circ_0007345 | 0.92137221 | 1.70E-10 |
| hsa_circ_0024731 | 0.920956217 | 1.79E-10 |
| hsa_circ_0001338 | 0.920386624 | 1.94E-10 |
| hsa_circ_0000453 | 0.919740357 | 2.11E-10 |
| hsa_circ_0048259 | 0.9197322 | 2.11E-10 |
| hsa_circ_0064136 | 0.919706858 | 2.12E-10 |
| hsa_circ_0003006 | 0.919486824 | 2.18E-10 |
| hsa_circ_0020749 | 0.918808096 | 2.38E-10 |
| hsa_circ_0041829 | 0.918342524 | 2.53E-10 |
| hsa_circ_0008784 | 0.91778913 | 2.72E-10 |
| hsa_circ_0031132 | 0.917529981 | 2.81E-10 |
| hsa_circ_0006735 | 0.916639695 | 3.15E-10 |
| hsa_circ_0092374 | 0.916566911 | 3.18E-10 |
| hsa_circ_0002191 | 0.916475174 | 3.22E-10 |
| hsa_circ_0087493 | 0.916367169 | 3.26E-10 |
| hsa_circ_0003110 | 0.915746553 | 3.53E-10 |
| hsa_circ_0003958 | 0.915579518 | 3.60E-10 |
| hsa_circ_0002782 | 0.915418323 | 3.68E-10 |
| hsa_circ_0018004 | 0.915374591 | 3.70E-10 |
| hsa_circ_0005873 | 0.912860269 | 5.04E-10 |
| hsa_circ_0000747 | 0.912625606 | 5.19E-10 |
| hsa_circ_0058988 | 0.912505245 | 5.27E-10 |
| hsa_circ_0008226 | 0.91232094 | 5.38E-10 |
| hsa_circ_0084443 | 0.912257729 | 5.43E-10 |
| hsa_circ_0003473 | 0.91182444 | 5.72E-10 |
| hsa_circ_0012283 | 0.911686451 | 5.81E-10 |
| hsa_circ_0029855 | 0.910956466 | 6.34E-10 |
| hsa_circ_0052166 | 0.910875469 | 6.40E-10 |
| hsa_circ_0007361 | 0.910517345 | 6.68E-10 |
| hsa_circ_0023179 | 0.909853127 | 7.22E-10 |
| hsa_circ_0000291 | 0.908872005 | 8.10E-10 |
| hsa_circ_0000862 | 0.908461779 | 8.49E-10 |
| hsa_circ_0004565 | 0.907602677 | 9.37E-10 |
| hsa_circ_0017639 | 0.907321119 | 9.68E-10 |
| hsa_circ_0003745 | 0.907201141 | 9.81E-10 |
| hsa_circ_0022587 | 0.906964909 | 1.01E-09 |
| hsa_circ_0054656 | 0.906855791 | 1.02E-09 |
| hsa_circ_0007396 | 0.905835506 | 1.14E-09 |
| hsa_circ_0089974 | 0.905627051 | 1.17E-09 |
| hsa_circ_0066444 | 0.90557833 | 1.18E-09 |
| hsa_circ_0003759 | 0.905067985 | 1.25E-09 |
| hsa_circ_0048492 | 0.904992844 | 1.26E-09 |
| hsa_circ_0000322 | 0.904669823 | 1.30E-09 |
| hsa_circ_0060558 | 0.904361495 | 1.35E-09 |
| hsa_circ_0044556 | 0.903419457 | 1.49E-09 |
| hsa_circ_0084677 | 0.903224437 | 1.53E-09 |
| hsa_circ_0004366 | 0.903219283 | 1.53E-09 |
| hsa_circ_0006577 | 0.903079805 | 1.55E-09 |
| hsa_circ_0007772 | 0.902744169 | 1.61E-09 |
| hsa_circ_0034325 | 0.902715435 | 1.61E-09 |
| hsa_circ_0008563 | 0.902243274 | 1.70E-09 |
| hsa_circ_0014132 | 0.902110621 | 1.72E-09 |
| hsa_circ_0082564 | 0.901798092 | 1.78E-09 |
| hsa_circ_0081342 | 0.901248618 | 1.89E-09 |
| hsa_circ_0039914 | 0.901056628 | 1.93E-09 |
| hsa_circ_0001489 | 0.900944031 | 1.95E-09 |
| hsa_circ_0008758 | 0.900892088 | 1.96E-09 |
| hsa_circ_0042521 | 0.900446232 | 2.06E-09 |
| hsa_circ_0040827 | 0.899273291 | 2.33E-09 |
| hsa_circ_0000517 | 0.898861762 | 2.43E-09 |
| hsa_circ_0007503 | 0.898781174 | 2.45E-09 |
| hsa_circ_0023696 | 0.89850437 | 2.52E-09 |
| hsa_circ_0024553 | 0.898298974 | 2.57E-09 |
| hsa_circ_0049547 | 0.897128829 | 2.90E-09 |
| hsa_circ_0028540 | 0.897058691 | 2.92E-09 |
| hsa_circ_0060063 | 0.896962354 | 2.95E-09 |
| hsa_circ_0024895 | 0.894873012 | 3.65E-09 |
| hsa_circ_0003892 | 0.894757533 | 3.69E-09 |
| hsa_circ_0091331 | 0.894680512 | 3.72E-09 |
| hsa_circ_0008942 | 0.894679463 | 3.72E-09 |
| hsa_circ_0082139 | 0.894279726 | 3.87E-09 |
| hsa_circ_0001394 | 0.894181601 | 3.91E-09 |
| hsa_circ_0004380 | 0.893988854 | 3.98E-09 |
| hsa_circ_0092303 | 0.893038945 | 4.37E-09 |
| hsa_circ_0028196 | 0.892662851 | 4.54E-09 |
| hsa_circ_0003270 | 0.891593553 | 5.03E-09 |
| hsa_circ_0061137 | 0.891277906 | 5.19E-09 |
| hsa_circ_0008946 | 0.891106305 | 5.27E-09 |
| hsa_circ_0083861 | 0.890987846 | 5.34E-09 |
| hsa_circ_0000228 | 0.890901202 | 5.38E-09 |
| hsa_circ_0075736 | 0.890889564 | 5.39E-09 |
| hsa_circ_0001827 | 0.890378309 | 5.66E-09 |
| hsa_circ_0004228 | 0.887795994 | 7.22E-09 |
| hsa_circ_0065964 | 0.887571471 | 7.37E-09 |
| hsa_circ_0092308 | 0.885298852 | 9.08E-09 |
| hsa_circ_0072386 | 0.883919527 | 1.03E-08 |
| hsa_circ_0054970 | 0.883157891 | 1.10E-08 |
| hsa_circ_0008419 | 0.881209768 | 1.31E-08 |
| hsa_circ_0007705 | 0.881184558 | 1.31E-08 |
| hsa_circ_0049392 | 0.880472713 | 1.40E-08 |
| hsa_circ_0045006 | 0.879255001 | 1.55E-08 |
| hsa_circ_0000137 | 0.878955268 | 1.59E-08 |
| hsa_circ_0003141 | 0.878865426 | 1.61E-08 |
| hsa_circ_0003478 | 0.878567881 | 1.65E-08 |
| hsa_circ_0000981 | 0.878526531 | 1.65E-08 |
| hsa_circ_0064288 | 0.878456517 | 1.66E-08 |
| hsa_circ_0002320 | 0.877444031 | 1.81E-08 |
| hsa_circ_0082140 | 0.87686589 | 1.90E-08 |
| hsa_circ_0092369 | 0.876658577 | 1.94E-08 |
| hsa_circ_0085459 | 0.876397525 | 1.98E-08 |
| hsa_circ_0060035 | 0.876343534 | 1.99E-08 |
| hsa_circ_0035292 | 0.87631719 | 1.99E-08 |
| hsa_circ_0007140 | 0.87584115 | 2.07E-08 |
| hsa_circ_0062682 | 0.874450853 | 2.33E-08 |
| hsa_circ_0008255 | 0.874232793 | 2.37E-08 |
| hsa_circ_0001749 | 0.873771294 | 2.46E-08 |
| hsa_circ_0030428 | 0.870955003 | 3.10E-08 |
| hsa_circ_0040921 | 0.869386254 | 3.51E-08 |
| hsa_circ_0090518 | 0.86862152 | 3.73E-08 |
| hsa_circ_0000053 | 0.86839461 | 3.80E-08 |
| hsa_circ_0008768 | 0.867104059 | 4.20E-08 |
| hsa_circ_0078738 | 0.867076336 | 4.21E-08 |
| hsa_circ_0008234 | 0.867075678 | 4.21E-08 |
| hsa_circ_0070700 | 0.866898062 | 4.27E-08 |
| hsa_circ_0079958 | 0.866107437 | 4.54E-08 |
| hsa_circ_0004166 | 0.865332003 | 4.82E-08 |
| hsa_circ_0008773 | 0.864532924 | 5.12E-08 |
| hsa_circ_0009904 | 0.86448962 | 5.14E-08 |
| hsa_circ_0089172 | 0.864378149 | 5.18E-08 |
| hsa_circ_0002224 | 0.864301319 | 5.21E-08 |
| hsa_circ_0001360 | 0.864016342 | 5.33E-08 |
| hsa_circ_0006109 | 0.863900881 | 5.38E-08 |
| hsa_circ_0000519 | 0.863635581 | 5.48E-08 |
| hsa_circ_0001917 | 0.863547078 | 5.52E-08 |
| hsa_circ_0000644 | 0.862315426 | 6.06E-08 |
| hsa_circ_0060055 | 0.862183068 | 6.12E-08 |
| hsa_circ_0049998 | 0.861562073 | 6.41E-08 |
| hsa_circ_0088222 | 0.861147293 | 6.61E-08 |
| hsa_circ_0007061 | 0.859411594 | 7.52E-08 |
| hsa_circ_0092289 | 0.859184454 | 7.64E-08 |
| hsa_circ_0064651 | 0.858224892 | 8.20E-08 |
| hsa_circ_0007481 | 0.856794799 | 9.09E-08 |
| hsa_circ_0074816 | 0.8564552 | 9.31E-08 |
| hsa_circ_0008263 | 0.856346816 | 9.39E-08 |
| hsa_circ_0020117 | 0.854703011 | 1.06E-07 |
| hsa_circ_0042823 | 0.854509079 | 1.07E-07 |
| hsa_circ_0008583 | 0.854485933 | 1.07E-07 |
| hsa_circ_0077929 | 0.854458657 | 1.07E-07 |
| hsa_circ_0014879 | 0.854273808 | 1.09E-07 |
| hsa_circ_0005336 | 0.854266471 | 1.09E-07 |
| hsa_circ_0022591 | 0.85382763 | 1.12E-07 |
| hsa_circ_0006789 | 0.853705321 | 1.13E-07 |
| hsa_circ_0006421 | 0.853118982 | 1.18E-07 |
| hsa_circ_0006900 | 0.85157845 | 1.31E-07 |
| hsa_circ_0001727 | 0.851130974 | 1.35E-07 |
| hsa_circ_0092313 | 0.850457415 | 1.42E-07 |
| hsa_circ_0053907 | 0.850358519 | 1.43E-07 |
| hsa_circ_0006608 | 0.85023117 | 1.44E-07 |
| hsa_circ_0043438 | 0.850075275 | 1.46E-07 |
| hsa_circ_0034786 | 0.849980081 | 1.47E-07 |
| hsa_circ_0092297 | 0.849523884 | 1.51E-07 |
| hsa_circ_0001851 | 0.848848312 | 1.58E-07 |
| hsa_circ_0003502 | 0.848789086 | 1.59E-07 |
| hsa_circ_0000518 | 0.848329307 | 1.64E-07 |
| hsa_circ_0088865 | 0.845605931 | 1.97E-07 |
| hsa_circ_0004071 | 0.845227943 | 2.02E-07 |
| hsa_circ_0092350 | 0.844291366 | 2.15E-07 |
| hsa_circ_0092319 | 0.844217969 | 2.16E-07 |
| hsa_circ_0008539 | 0.843375889 | 2.28E-07 |
| hsa_circ_0005939 | 0.843229018 | 2.30E-07 |
| hsa_circ_0008616 | 0.84259193 | 2.40E-07 |
| hsa_circ_0003645 | 0.841969239 | 2.50E-07 |
| hsa_circ_0001658 | 0.841727526 | 2.54E-07 |
| hsa_circ_0030793 | 0.841019332 | 2.66E-07 |
| hsa_circ_0086694 | 0.840806954 | 2.69E-07 |
| hsa_circ_0011279 | 0.839734896 | 2.88E-07 |
| hsa_circ_0002078 | 0.839679235 | 2.89E-07 |
| hsa_circ_0079675 | 0.839045046 | 3.01E-07 |
| hsa_circ_0036287 | 0.836958479 | 3.44E-07 |
| hsa_circ_0001056 | 0.836291753 | 3.58E-07 |
| hsa_circ_0009142 | 0.836155789 | 3.61E-07 |
| hsa_circ_0007099 | 0.836083047 | 3.63E-07 |
| hsa_circ_0003472 | 0.836065841 | 3.63E-07 |
| hsa_circ_0023182 | 0.835886342 | 3.67E-07 |
| hsa_circ_0033408 | 0.835702898 | 3.72E-07 |
| hsa_circ_0058058 | 0.835294472 | 3.81E-07 |
| hsa_circ_0005687 | 0.835213966 | 3.83E-07 |
| hsa_circ_0082917 | 0.83514481 | 3.85E-07 |
| hsa_circ_0041150 | 0.835018027 | 3.88E-07 |
| hsa_circ_0092327 | 0.834443863 | 4.02E-07 |
| hsa_circ_0044396 | 0.832537745 | 4.51E-07 |
| hsa_circ_0088045 | 0.832353641 | 4.56E-07 |
| hsa_circ_0002513 | 0.832198952 | 4.61E-07 |
| hsa_circ_0007608 | 0.83131211 | 4.86E-07 |
| hsa_circ_0067934 | 0.830568515 | 5.08E-07 |
| hsa_circ_0001346 | 0.828137054 | 5.87E-07 |
| hsa_circ_0023404 | 0.8264316 | 6.49E-07 |
| hsa_circ_0001500 | 0.825809264 | 6.73E-07 |
| hsa_circ_0003770 | 0.824931661 | 7.08E-07 |
| hsa_circ_0091710 | 0.824174308 | 7.40E-07 |
| hsa_circ_0069752 | 0.822592035 | 8.10E-07 |
| hsa_circ_0004006 | 0.82015484 | 9.30E-07 |
| hsa_circ_0087640 | 0.820107997 | 9.33E-07 |
| hsa_circ_0023033 | 0.81810795 | 1.04E-06 |
| hsa_circ_0010486 | 0.818039535 | 1.05E-06 |
| hsa_circ_0002003 | 0.817516282 | 1.08E-06 |
| hsa_circ_0012300 | 0.817150518 | 1.10E-06 |
| hsa_circ_0084615 | 0.814435073 | 1.28E-06 |
| hsa_circ_0004946 | 0.813885779 | 1.31E-06 |
| hsa_circ_0085711 | 0.812847727 | 1.39E-06 |
| hsa_circ_0003220 | 0.81220247 | 1.44E-06 |
| hsa_circ_0070396 | 0.811735172 | 1.48E-06 |
| hsa_circ_0002153 | 0.80970486 | 1.64E-06 |
| hsa_circ_0000721 | 0.807704159 | 1.83E-06 |
| hsa_circ_0008514 | 0.80643618 | 1.95E-06 |
| hsa_circ_0050834 | 0.80632669 | 1.96E-06 |
| hsa_circ_0092363 | 0.805942696 | 2.00E-06 |
| hsa_circ_0092310 | 0.805868668 | 2.01E-06 |
| hsa_circ_0005742 | 0.804607472 | 2.14E-06 |
| hsa_circ_0045862 | 0.804537072 | 2.15E-06 |
| hsa_circ_0001054 | 0.804004229 | 2.21E-06 |
| hsa_circ_0055734 | 0.803797772 | 2.23E-06 |
| hsa_circ_0092337 | 0.803485885 | 2.27E-06 |
| hsa_circ_0000463 | 0.803396647 | 2.28E-06 |
| hsa_circ_0039053 | 0.802193216 | 2.42E-06 |
| hsa_circ_0018814 | 0.80180619 | 2.47E-06 |
| hsa_circ_0009456 | 0.801771939 | 2.48E-06 |
| hsa_circ_0067716 | 0.800955613 | 2.58E-06 |
| hsa_circ_0046123 | 0.800773201 | 2.60E-06 |
| hsa_circ_0092331 | 0.797996209 | 2.99E-06 |
| hsa_circ_0005435 | 0.797822087 | 3.02E-06 |
| hsa_circ_0001359 | 0.796792642 | 3.17E-06 |
| hsa_circ_0007427 | 0.796546756 | 3.21E-06 |
| hsa_circ_0007001 | 0.795976828 | 3.30E-06 |
| hsa_circ_0046882 | 0.793914067 | 3.65E-06 |
| hsa_circ_0006371 | 0.793075366 | 3.80E-06 |
| hsa_circ_0000434 | 0.792529301 | 3.90E-06 |
| hsa_circ_0000514 | 0.792505841 | 3.91E-06 |
| hsa_circ_0068563 | 0.792151819 | 3.97E-06 |
| hsa_circ_0070382 | 0.791728469 | 4.05E-06 |
| hsa_circ_0007527 | 0.789782968 | 4.45E-06 |
| hsa_circ_0065214 | 0.789617536 | 4.48E-06 |
| hsa_circ_0016979 | 0.788827521 | 4.65E-06 |
| hsa_circ_0001627 | 0.788719875 | 4.68E-06 |
| hsa_circ_0041732 | 0.787432188 | 4.97E-06 |
| hsa_circ_0002039 | 0.786842413 | 5.11E-06 |
| hsa_circ_0056139 | 0.786612939 | 5.16E-06 |
| hsa_circ_0026143 | 0.786076947 | 5.29E-06 |
| hsa_circ_0008310 | 0.785982418 | 5.31E-06 |
| hsa_circ_0000331 | 0.785973941 | 5.32E-06 |
| hsa_circ_0008719 | 0.785313404 | 5.48E-06 |
| hsa_circ_0009581 | 0.785161826 | 5.52E-06 |
| hsa_circ_0007409 | 0.784744389 | 5.63E-06 |
| hsa_circ_0072012 | 0.784590441 | 5.67E-06 |
| hsa_circ_0000916 | 0.783690224 | 5.91E-06 |
| hsa_circ_0011692 | 0.783276226 | 6.02E-06 |
| hsa_circ_0039557 | 0.782818416 | 6.15E-06 |
| hsa_circ_0092299 | 0.78277193 | 6.16E-06 |
| hsa_circ_0088046 | 0.782613671 | 6.20E-06 |
| hsa_circ_0007676 | 0.782574027 | 6.21E-06 |
| hsa_circ_0080425 | 0.780291675 | 6.89E-06 |
| hsa_circ_0005699 | 0.779219972 | 7.23E-06 |
| hsa_circ_0001343 | 0.778610849 | 7.43E-06 |
| hsa_circ_0058189 | 0.777676879 | 7.75E-06 |
| hsa_circ_0092277 | 0.775394238 | 8.57E-06 |
| hsa_circ_0001279 | 0.774840454 | 8.78E-06 |
| hsa_circ_0082141 | 0.773500365 | 9.31E-06 |
| hsa_circ_0036768 | 0.773370406 | 9.36E-06 |
| hsa_circ_0003695 | 0.772139702 | 9.87E-06 |
| hsa_circ_0013048 | 0.770522873 | 1.06E-05 |
| hsa_circ_0092275 | 0.769147599 | 1.12E-05 |
| hsa_circ_0007646 | 0.768799719 | 1.14E-05 |
| hsa_circ_0068176 | 0.767420582 | 1.21E-05 |
| hsa_circ_0004291 | 0.767294385 | 1.21E-05 |
| hsa_circ_0070934 | 0.765760992 | 1.29E-05 |
| hsa_circ_0002406 | 0.76469892 | 1.35E-05 |
| hsa_circ_0009022 | 0.764342515 | 1.37E-05 |
| hsa_circ_0003738 | 0.763302882 | 1.43E-05 |
| hsa_circ_0085803 | 0.760649134 | 1.60E-05 |
| hsa_circ_0000740 | 0.759856716 | 1.65E-05 |
| hsa_circ_0024609 | 0.758363591 | 1.76E-05 |
| hsa_circ_0042415 | 0.758294046 | 1.76E-05 |
| hsa_circ_0003832 | 0.756895165 | 1.86E-05 |
| hsa_circ_0000682 | 0.756017735 | 1.93E-05 |
| hsa_circ_0008284 | 0.753998788 | 2.09E-05 |
| hsa_circ_0078165 | 0.753449143 | 2.14E-05 |
| hsa_circ_0000723 | 0.749289952 | 2.51E-05 |
| hsa_circ_0006827 | 0.748911281 | 2.55E-05 |
| hsa_circ_0001436 | 0.748891301 | 2.55E-05 |
| hsa_circ_0038648 | 0.747773029 | 2.67E-05 |
| hsa_circ_0006055 | 0.746703779 | 2.78E-05 |
| hsa_circ_0006376 | 0.746513291 | 2.80E-05 |
| hsa_circ_0087564 | 0.745863375 | 2.87E-05 |
| hsa_circ_0022505 | 0.745508639 | 2.91E-05 |
| hsa_circ_0084162 | 0.742721757 | 3.23E-05 |
| hsa_circ_0004720 | 0.742332454 | 3.28E-05 |
| hsa_circ_0022015 | 0.740944617 | 3.45E-05 |
| hsa_circ_0001998 | 0.740900061 | 3.46E-05 |
| hsa_circ_0003247 | 0.740877743 | 3.46E-05 |
| hsa_circ_0070933 | 0.738038455 | 3.85E-05 |
| hsa_circ_0037078 | 0.73721705 | 3.96E-05 |
| hsa_circ_0046760 | 0.735540945 | 4.21E-05 |
| hsa_circ_0088485 | 0.735365767 | 4.24E-05 |
| hsa_circ_0008654 | 0.734190933 | 4.43E-05 |
| hsa_circ_0029636 | 0.732552298 | 4.69E-05 |
| hsa_circ_0092317 | 0.731905656 | 4.81E-05 |
| hsa_circ_0082356 | 0.728913123 | 5.35E-05 |
| hsa_circ_0036683 | 0.728888784 | 5.35E-05 |
| hsa_circ_0006232 | 0.728870804 | 5.35E-05 |
| hsa_circ_0043812 | 0.728053161 | 5.51E-05 |
| hsa_circ_0023180 | 0.727410615 | 5.64E-05 |
| hsa_circ_0064416 | 0.725771785 | 5.97E-05 |
| hsa_circ_0006461 | 0.725661226 | 5.99E-05 |
| hsa_circ_0089153 | 0.722389205 | 6.71E-05 |
| hsa_circ_0003655 | 0.722241463 | 6.75E-05 |
| hsa_circ_0027774 | 0.721605361 | 6.90E-05 |
| hsa_circ_0084429 | 0.720073695 | 7.27E-05 |
| hsa_circ_0070421 | 0.719174129 | 7.49E-05 |
| hsa_circ_0072430 | 0.718788471 | 7.59E-05 |
| hsa_circ_0008016 | 0.718443435 | 7.68E-05 |
| hsa_circ_0036113 | 0.717853972 | 7.84E-05 |
| hsa_circ_0007551 | 0.717612696 | 7.90E-05 |
| hsa_circ_0049700 | 0.714724766 | 8.70E-05 |
| hsa_circ_0006566 | 0.714228508 | 8.85E-05 |
| hsa_circ_0073027 | 0.713097435 | 9.19E-05 |
| hsa_circ_0000877 | 0.712458173 | 9.38E-05 |
| hsa_circ_0002945 | 0.709411723 | 0.000103715 |
| hsa_circ_0002733 | 0.709042729 | 0.000104971 |
| hsa_circ_0038872 | 0.707239798 | 0.000111298 |
| hsa_circ_0002172 | 0.706103862 | 0.000115453 |
| hsa_circ_0007796 | 0.704411757 | 0.000121895 |
| hsa_circ_0079380 | 0.701428911 | 0.000134018 |
| hsa_circ_0054598 | 0.700351819 | 0.000138648 |
| hsa_circ_0007404 | 0.693341493 | 0.000172343 |
| hsa_circ_0007528 | 0.692407327 | 0.000177332 |
| hsa_circ_0000633 | 0.692070945 | 0.000179159 |
| hsa_circ_0008223 | 0.68901917 | 0.000196502 |
| hsa_circ_0078357 | 0.686205362 | 0.000213771 |
| hsa_circ_0007378 | 0.685965604 | 0.000215301 |
| hsa_circ_0018824 | 0.684548308 | 0.000224547 |
| hsa_circ_0001854 | 0.68419925 | 0.000226876 |
| hsa_circ_0087636 | 0.682351184 | 0.000239562 |
| hsa_circ_0006370 | 0.679607175 | 0.000259538 |
| hsa_circ_0048807 | 0.670883571 | 0.000332983 |
| hsa_circ_0076054 | 0.669879727 | 0.000342493 |
| hsa_circ_0046523 | 0.66850031 | 0.000355944 |
| hsa_circ_0076412 | 0.664069506 | 0.000402302 |
| hsa_circ_0005038 | 0.657202238 | 0.000484488 |
| hsa_circ_0014130 | 0.656760946 | 0.000490233 |
| hsa_circ_0044185 | 0.655320061 | 0.000509404 |
| hsa_circ_0080635 | 0.653334678 | 0.00053688 |
| hsa_circ_0007146 | 0.648985501 | 0.000601583 |
| hsa_circ_0051657 | 0.647540569 | 0.00062452 |
| hsa_circ_0000915 | 0.646919488 | 0.000634608 |
| hsa_circ_0006089 | 0.640251671 | 0.000752098 |
| hsa_circ_0014624 | 0.632660988 | 0.000908234 |
| hsa_circ_0000515 | 0.632009508 | 0.000922847 |
| hsa_circ_0011002 | 0.628834407 | 0.000996995 |
| hsa_circ_0034044 | 0.628690666 | 0.00100047 |
| hsa_circ_0002311 | 0.624742633 | 0.001100057 |
| hsa_circ_0007277 | 0.624452508 | 0.001107699 |
| hsa_circ_0079492 | 0.623967214 | 0.001120585 |
| hsa_circ_0000524 | 0.622797149 | 0.001152182 |
| hsa_circ_0001542 | 0.62208719 | 0.001171725 |
| hsa_circ_0008261 | 0.618924315 | 0.001262281 |
| hsa_circ_0084678 | 0.612098436 | 0.001478345 |
| hsa_circ_0092278 | 0.60747378 | 0.001642074 |
| hsa_circ_0009117 | 0.605866215 | 0.001702502 |
| hsa_circ_0002754 | 0.605065002 | 0.001733322 |
| hsa_circ_0009131 | 0.604214296 | 0.001766565 |
| hsa_circ_0000512 | 0.601557837 | 0.001873899 |
| hsa_circ_0007540 | 0.601161857 | 0.001890365 |
| hsa_circ_0002780 | 0.599484138 | 0.001961506 |
| hsa_circ_0042498 | 0.588841 | 0.002468139 |
| hsa_circ_0063534 | 0.587738271 | 0.002526471 |
| hsa_circ_0002111 | 0.580990354 | 0.002909538 |
| hsa_circ_0082352 | 0.579578913 | 0.002995596 |
| hsa_circ_0038306 | 0.57772911 | 0.003111639 |
| hsa_circ_0000929 | 0.565244525 | 0.003998771 |
| hsa_circ_0001361 | 0.559637486 | 0.004461659 |
| hsa_circ_0020934 | 0.558211189 | 0.004586345 |
| hsa_circ_0001212 | 0.551684541 | 0.005194944 |
| hsa_circ_0005813 | 0.551128879 | 0.005249748 |
| hsa_circ_0066147 | 0.547714735 | 0.005597236 |
| hsa_circ_0006462 | 0.547523362 | 0.00561727 |
| hsa_circ_0051042 | 0.545572823 | 0.005824921 |
| hsa_circ_0050745 | 0.543185872 | 0.006087738 |
| hsa_circ_0006010 | 0.541708367 | 0.006255337 |
| hsa_circ_0083126 | 0.536591762 | 0.006865843 |
| hsa_circ_0002289 | 0.536075924 | 0.006930059 |
| hsa_circ_0006570 | 0.530439424 | 0.007664995 |
| hsa_circ_0015936 | 0.527818756 | 0.008028165 |
| hsa_circ_0000940 | 0.521762563 | 0.008922452 |
| hsa_circ_0032858 | 0.520530477 | 0.009114142 |
| hsa_circ_0011636 | 0.511737467 | 0.010583724 |
| hsa_circ_0000936 | 0.510139005 | 0.010870822 |
| hsa_circ_0006209 | 0.506383937 | 0.011570633 |
| hsa_circ_0057552 | 0.506049141 | 0.01163479 |
| hsa_circ_0000033 | 0.496574669 | 0.013575635 |
| hsa_circ_0000511 | 0.492995348 | 0.014374729 |
| hsa_circ_0001667 | 0.491379799 | 0.014747817 |
| hsa_circ_0092291 | 0.488694082 | 0.015385569 |
| hsa_circ_0048687 | 0.469688359 | 0.020571999 |
| hsa_circ_0001171 | 0.447885183 | 0.028178181 |
| hsa_circ_0004096 | 0.440470754 | 0.031225692 |
| hsa_circ_0001265 | 0.436971577 | 0.032752396 |
| hsa_circ_0008729 | 0.42744118 | 0.037213237 |
| hsa_circ_0024143 | 0.425323337 | 0.038266903 |
| hsa_circ_0003001 | 0.378912562 | 0.067855626 |
| hsa_circ_0006614 | 0.341736062 | 0.10217 |
| hsa_circ_0035560 | 0.295627526 | 0.160754435 |
| hsa_circ_0092284 | -0.294152912 | 0.162951251 |
| hsa_circ_0038082 | -0.378082921 | 0.068509654 |
| hsa_circ_0092287 | -0.426347986 | 0.037754217 |
| hsa_circ_0049586 | -0.445333234 | 0.02919896 |
| hsa_circ_0000040 | -0.451616236 | 0.026737393 |
| hsa_circ_0002286 | -0.464865717 | 0.022091433 |
| hsa_circ_0002080 | -0.476347903 | 0.018614117 |
| hsa_circ_0002380 | -0.493213369 | 0.014324978 |
| hsa_circ_0036231 | -0.501852877 | 0.012464081 |
| hsa_circ_0000866 | -0.52601287 | 0.008286661 |
| hsa_circ_0008887 | -0.527344316 | 0.008095419 |
| hsa_circ_0004711 | -0.557130582 | 0.00468275 |
| hsa_circ_0030764 | -0.558907584 | 0.004525105 |
| hsa_circ_0003421 | -0.593932701 | 0.002213405 |
| hsa_circ_0020336 | -0.600930708 | 0.001900034 |
| hsa_circ_0023988 | -0.606934646 | 0.001662132 |
| hsa_circ_0008628 | -0.610082528 | 0.001547911 |
| hsa_circ_0087234 | -0.612476187 | 0.001465612 |
| hsa_circ_0043497 | -0.621769762 | 0.001180554 |
| hsa_circ_0057319 | -0.629016177 | 0.000992616 |
| hsa_circ_0006419 | -0.635615668 | 0.00084443 |
| hsa_circ_0061035 | -0.648209824 | 0.000613804 |
| hsa_circ_0007167 | -0.664026229 | 0.000402779 |
| hsa_circ_0002318 | -0.665746253 | 0.000384178 |
| hsa_circ_0080797 | -0.666452027 | 0.000376764 |
| hsa_circ_0045306 | -0.667347973 | 0.000367531 |
| hsa_circ_0004795 | -0.670370766 | 0.000337812 |
| hsa_circ_0059151 | -0.672001932 | 0.000322659 |
| hsa_circ_0037955 | -0.67300338 | 0.000313652 |
| hsa_circ_0071869 | -0.676166791 | 0.000286618 |
| hsa_circ_0081683 | -0.679085378 | 0.000263496 |
| hsa_circ_0008253 | -0.684081619 | 0.000227666 |
| hsa_circ_0058792 | -0.688229716 | 0.000201219 |
| hsa_circ_0000292 | -0.689616638 | 0.000192996 |
| hsa_circ_0038090 | -0.690530126 | 0.000187741 |
| hsa_circ_0032391 | -0.693307172 | 0.000172524 |
| hsa_circ_0002981 | -0.695443142 | 0.000161563 |
| hsa_circ_0004463 | -0.700919211 | 0.000136192 |
| hsa_circ_0087232 | -0.702083862 | 0.00013127 |
| hsa_circ_0000246 | -0.706683992 | 0.000113314 |
| hsa_circ_0082142 | -0.707548699 | 0.00011019 |
| hsa_circ_0062577 | -0.709268368 | 0.000104201 |
| hsa_circ_0002077 | -0.711408577 | 9.71E-05 |
| hsa_circ_0008153 | -0.712270949 | 9.44E-05 |
| hsa_circ_0032202 | -0.713034944 | 9.21E-05 |
| hsa_circ_0090429 | -0.717041284 | 8.05E-05 |
| hsa_circ_0007845 | -0.718124115 | 7.77E-05 |
| hsa_circ_0000715 | -0.720162285 | 7.25E-05 |
| hsa_circ_0053967 | -0.722357087 | 6.72E-05 |
| hsa_circ_0051637 | -0.724562351 | 6.23E-05 |
| hsa_circ_0038844 | -0.728575803 | 5.41E-05 |
| hsa_circ_0004816 | -0.729172728 | 5.30E-05 |
| hsa_circ_0005029 | -0.730867675 | 4.99E-05 |
| hsa_circ_0001874 | -0.731412028 | 4.89E-05 |
| hsa_circ_0006128 | -0.732365042 | 4.73E-05 |
| hsa_circ_0008160 | -0.734233044 | 4.42E-05 |
| hsa_circ_0039857 | -0.735825978 | 4.17E-05 |
| hsa_circ_0072932 | -0.736443245 | 4.08E-05 |
| hsa_circ_0024162 | -0.736655943 | 4.05E-05 |
| hsa_circ_0025135 | -0.738879537 | 3.73E-05 |
| hsa_circ_0003016 | -0.740904065 | 3.46E-05 |
| hsa_circ_0000708 | -0.741300971 | 3.41E-05 |
| hsa_circ_0029634 | -0.743344197 | 3.16E-05 |
| hsa_circ_0004001 | -0.750184579 | 2.43E-05 |
| hsa_circ_0007707 | -0.75061143 | 2.39E-05 |
| hsa_circ_0015449 | -0.757298889 | 1.83E-05 |
| hsa_circ_0092293 | -0.757614277 | 1.81E-05 |
| hsa_circ_0005542 | -0.758172395 | 1.77E-05 |
| hsa_circ_0088072 | -0.763645296 | 1.41E-05 |
| hsa_circ_0041252 | -0.764235821 | 1.38E-05 |
| hsa_circ_0043691 | -0.764935142 | 1.34E-05 |
| hsa_circ_0008839 | -0.769131116 | 1.12E-05 |
| hsa_circ_0006958 | -0.769197402 | 1.12E-05 |
| hsa_circ_0079540 | -0.769574276 | 1.10E-05 |
| hsa_circ_0003859 | -0.771544881 | 1.01E-05 |
| hsa_circ_0008043 | -0.772898727 | 9.55E-06 |
| hsa_circ_0001942 | -0.775122891 | 8.67E-06 |
| hsa_circ_0000844 | -0.776264699 | 8.24E-06 |
| hsa_circ_0005218 | -0.77797015 | 7.65E-06 |
| hsa_circ_0058794 | -0.778745074 | 7.39E-06 |
| hsa_circ_0078051 | -0.781284012 | 6.59E-06 |
| hsa_circ_0007765 | -0.781450691 | 6.54E-06 |
| hsa_circ_0008334 | -0.783285201 | 6.02E-06 |
| hsa_circ_0092362 | -0.787974131 | 4.84E-06 |
| hsa_circ_0001221 | -0.794432319 | 3.56E-06 |
| hsa_circ_0017348 | -0.79536381 | 3.40E-06 |
| hsa_circ_0005231 | -0.796963689 | 3.15E-06 |
| hsa_circ_0007179 | -0.798612648 | 2.90E-06 |
| hsa_circ_0054263 | -0.801935193 | 2.46E-06 |
| hsa_circ_0023704 | -0.802168383 | 2.43E-06 |
| hsa_circ_0063329 | -0.805615133 | 2.03E-06 |
| hsa_circ_0003568 | -0.807652228 | 1.83E-06 |
| hsa_circ_0004840 | -0.80772217 | 1.82E-06 |
| hsa_circ_0008496 | -0.808064349 | 1.79E-06 |
| hsa_circ_0041915 | -0.809874767 | 1.63E-06 |
| hsa_circ_0007330 | -0.811074644 | 1.53E-06 |
| hsa_circ_0004494 | -0.813151567 | 1.37E-06 |
| hsa_circ_0069248 | -0.814807846 | 1.25E-06 |
| hsa_circ_0001443 | -0.814851748 | 1.25E-06 |
| hsa_circ_0005830 | -0.817240738 | 1.09E-06 |
| hsa_circ_0007888 | -0.81834474 | 1.03E-06 |
| hsa_circ_0052767 | -0.818386701 | 1.03E-06 |
| hsa_circ_0007875 | -0.82362841 | 7.64E-07 |
| hsa_circ_0001861 | -0.824760636 | 7.15E-07 |
| hsa_circ_0001516 | -0.828584254 | 5.72E-07 |
| hsa_circ_0050278 | -0.829651499 | 5.37E-07 |
| hsa_circ_0001301 | -0.830031127 | 5.25E-07 |
| hsa_circ_0004660 | -0.831707697 | 4.74E-07 |
| hsa_circ_0009590 | -0.836489413 | 3.54E-07 |
| hsa_circ_0004630 | -0.836602412 | 3.51E-07 |
| hsa_circ_0045194 | -0.838137925 | 3.19E-07 |
| hsa_circ_0043302 | -0.841335349 | 2.60E-07 |
| hsa_circ_0029961 | -0.841796543 | 2.53E-07 |
| hsa_circ_0038350 | -0.842341394 | 2.44E-07 |
| hsa_circ_0007113 | -0.844041857 | 2.18E-07 |
| hsa_circ_0003273 | -0.84476173 | 2.08E-07 |
| hsa_circ_0005982 | -0.846561764 | 1.85E-07 |
| hsa_circ_0007180 | -0.847436273 | 1.74E-07 |
| hsa_circ_0058805 | -0.848711433 | 1.60E-07 |
| hsa_circ_0005651 | -0.850266262 | 1.44E-07 |
| hsa_circ_0092372 | -0.850397818 | 1.42E-07 |
| hsa_circ_0011159 | -0.852630891 | 1.22E-07 |
| hsa_circ_0008533 | -0.854132092 | 1.10E-07 |
| hsa_circ_0009343 | -0.856867607 | 9.04E-08 |
| hsa_circ_0042472 | -0.860549158 | 6.91E-08 |
| hsa_circ_0062426 | -0.866001187 | 4.58E-08 |
| hsa_circ_0065217 | -0.867041301 | 4.22E-08 |
| hsa_circ_0001837 | -0.868553635 | 3.75E-08 |
| hsa_circ_0032664 | -0.868570436 | 3.75E-08 |
| hsa_circ_0063853 | -0.870865908 | 3.12E-08 |
| hsa_circ_0080000 | -0.873515145 | 2.52E-08 |
| hsa_circ_0011929 | -0.875136682 | 2.20E-08 |
| hsa_circ_0004673 | -0.875852292 | 2.07E-08 |
| hsa_circ_0006797 | -0.876189074 | 2.01E-08 |
| hsa_circ_0032683 | -0.87996153 | 1.46E-08 |
| hsa_circ_0001579 | -0.880797355 | 1.36E-08 |
| hsa_circ_0065220 | -0.882611404 | 1.16E-08 |
| hsa_circ_0003315 | -0.885041471 | 9.30E-09 |
| hsa_circ_0002637 | -0.885980095 | 8.54E-09 |
| hsa_circ_0014292 | -0.886939517 | 7.81E-09 |
| hsa_circ_0008832 | -0.888728349 | 6.61E-09 |
| hsa_circ_0006352 | -0.888899991 | 6.51E-09 |
| hsa_circ_0065223 | -0.88936363 | 6.23E-09 |
| hsa_circ_0062545 | -0.889370259 | 6.22E-09 |
| hsa_circ_0019083 | -0.895900485 | 3.29E-09 |
| hsa_circ_0050102 | -0.897812554 | 2.71E-09 |
| hsa_circ_0070040 | -0.90205686 | 1.73E-09 |
| hsa_circ_0005358 | -0.904935946 | 1.27E-09 |
| hsa_circ_0037858 | -0.91077528 | 6.48E-10 |
| hsa_circ_0008557 | -0.911297396 | 6.09E-10 |
| hsa_circ_0047303 | -0.911407267 | 6.01E-10 |
| hsa_circ_0091570 | -0.913199392 | 4.84E-10 |
| hsa_circ_0085154 | -0.915445706 | 3.67E-10 |
| hsa_circ_0059369 | -0.918191724 | 2.58E-10 |
| hsa_circ_0036751 | -0.921800164 | 1.60E-10 |
| hsa_circ_0013561 | -0.926537996 | 8.23E-11 |
| hsa_circ_0008351 | -0.927703736 | 6.94E-11 |
| hsa_circ_0040994 | -0.928602707 | 6.07E-11 |
| hsa_circ_0004099 | -0.930330485 | 4.68E-11 |
| hsa_circ_0008193 | -0.931160484 | 4.11E-11 |
| hsa_circ_0058493 | -0.943189522 | 5.26E-12 |
| hsa_circ_0006302 | -0.949879792 | 1.37E-12 |
| hsa_circ_0020174 | -0.952668868 | 7.39E-13 |
| hsa_circ_0013339 | -0.955759741 | 3.57E-13 |
| hsa_circ_0036044 | -0.96509978 | 2.74E-14 |
