# Supplementary material for: Up-Regulation of hsa_circ_0000517 Predicts Adverse Prognosis of Hepatocellular Carcinoma
Source: Front Oncol. 2019 Oct 22;9:1105. doi: 10.3389/fonc.2019.01105 (PMC6842961; doi:10.3389/fonc.2019.01105)
Supplement: Supplementary file 5 [file Table_5.DOCX]

**Table S5 The hsa_circ_0000517-targeted miRNAs predicted on CSCD Database.**

| CircRNA ID | Family | MSA Start | MSA End | Site Type |
| --- | --- | --- | --- | --- |
| chr14:20811404\|20811492 | miR-1178-5p | 43 | 48 | 6mer |
| chr14:20811404\|20811492 | miR-1182 | 42 | 48 | 7mer-m8 |
| chr14:20811404\|20811492 | miR-1199-3p | 20 | 25 | 6mer |
| chr14:20811404\|20811492 | miR-1291/6775-3p | 18 | 23 | 6mer |
| chr14:20811404\|20811492 | miR-1296-5p | 30 | 36 | 7mer-m8 |
| chr14:20811404\|20811492 | miR-146-5p/7153-5p | 62 | 67 | 6mer |
| chr14:20811404\|20811492 | miR-146b-3p | 32 | 37 | 6mer |
| chr14:20811404\|20811492 | miR-149-3p/4728-5p/6785-5p/6883-5p | 78 | 83 | 6mer |
| chr14:20811404\|20811492 | miR-186-3p | 16 | 21 | 6mer |
| chr14:20811404\|20811492 | miR-18b-3p | 16 | 22 | 7mer-m8 |
| chr14:20811404\|20811492 | miR-193a-5p | 42 | 47 | 6mer |
| chr14:20811404\|20811492 | miR-296-5p | 30 | 36 | 7mer-1a |
| chr14:20811404\|20811492 | miR-3123 | 63 | 68 | 6mer |
| chr14:20811404\|20811492 | miR-3175 | 79 | 84 | 6mer |
| chr14:20811404\|20811492 | miR-3197 | 23 | 28 | 6mer |
| chr14:20811404\|20811492 | miR-3672/6864-3p | 36 | 42 | 7mer-m8 |
| chr14:20811404\|20811492 | miR-3689/6851-5p | 76 | 82 | 7mer-m8 |
| chr14:20811404\|20811492 | miR-381-5p | 46 | 51 | 6mer |
| chr14:20811404\|20811492 | miR-3919 | 63 | 69 | 7mer-m8 |
| chr14:20811404\|20811492 | miR-3925-5p | 62 | 68 | 7mer-m8 |
| chr14:20811404\|20811492 | miR-4251 | 38 | 44 | 7mer-1a |
| chr14:20811404\|20811492 | miR-4257 | 25 | 30 | 6mer |
| chr14:20811404\|20811492 | miR-4283 | 55 | 61 | 7mer-m8 |
| chr14:20811404\|20811492 | miR-4303 | 39 | 44 | 6mer |
| chr14:20811404\|20811492 | miR-4329 | 37 | 44 | 8mer-1a |
| chr14:20811404\|20811492 | miR-4437 | 55 | 60 | 6mer |
| chr14:20811404\|20811492 | miR-4467 | 21 | 26 | 6mer |
| chr14:20811404\|20811492 | miR-4489 | 56 | 61 | 6mer |
| chr14:20811404\|20811492 | miR-4508 | 56 | 62 | 7mer-m8 |
| chr14:20811404\|20811492 | miR-4512 | 30 | 36 | 7mer-1a |
| chr14:20811404\|20811492 | miR-4527/6503-5p | 41 | 46 | 6mer |
| chr14:20811404\|20811492 | miR-4640-5p/4726-5p | 28 | 34 | 7mer-m8 |
| chr14:20811404\|20811492 | miR-4710 | 74 | 79 | 6mer |
| chr14:20811404\|20811492 | miR-4756-3p | 64 | 69 | 6mer |
| chr14:20811404\|20811492 | miR-4792 | 74 | 79 | 6mer |
| chr14:20811404\|20811492 | miR-486-3p | 57 | 62 | 6mer |
| chr14:20811404\|20811492 | miR-5090/6775-5p | 57 | 62 | 6mer |
| chr14:20811404\|20811492 | miR-542-5p | 80 | 87 | 8mer-1a |
| chr14:20811404\|20811492 | miR-589-5p | 62 | 67 | 6mer |
| chr14:20811404\|20811492 | miR-6075 | 19 | 24 | 6mer |
| chr14:20811404\|20811492 | miR-6087 | 22 | 28 | 7mer-m8 |
| chr14:20811404\|20811492 | miR-6134 | 76 | 81 | 6mer |
| chr14:20811404\|20811492 | miR-642b-5p | 69 | 74 | 6mer |
| chr14:20811404\|20811492 | miR-6508-3p | 29 | 34 | 6mer |
| chr14:20811404\|20811492 | miR-6727-5p | 81 | 87 | 7mer-1a |
| chr14:20811404\|20811492 | miR-6753-3p/7107-3p | 41 | 46 | 6mer |
| chr14:20811404\|20811492 | miR-6761-5p | 38 | 44 | 7mer-1a |
| chr14:20811404\|20811492 | miR-6777-5p/6889-5p | 79 | 86 | 8mer-1a |
| chr14:20811404\|20811492 | miR-6799-5p | 77 | 83 | 7mer-m8 |
| chr14:20811404\|20811492 | miR-6804-5p | 44 | 49 | 6mer |
| chr14:20811404\|20811492 | miR-6825-5p | 78 | 84 | 7mer-m8 |
| chr14:20811404\|20811492 | miR-6847-5p | 25 | 30 | 6mer |
| chr14:20811404\|20811492 | miR-6851-3p | 18 | 23 | 6mer |
| chr14:20811404\|20811492 | miR-6895-5p | 29 | 36 | 8mer-1a |
| chr14:20811404\|20811492 | miR-7106-5p | 78 | 83 | 6mer |
| chr14:20811404\|20811492 | miR-7160-3p | 30 | 36 | 7mer-1a |
| chr14:20811404\|20811492 | miR-7854-3p | 75 | 81 | 7mer-m8 |
| chr14:20811404\|20811492 | miR-7977 | 67 | 73 | 7mer-1a |
| chr14:20811404\|20811492 | miR-937-3p | 50 | 56 | 7mer-m8 |
| chr14:20811404\|20811492 | miR-939-5p/1343-5p | 79 | 84 | 6mer |
