# Supplementary material for: Up-Regulation of hsa_circ_0000517 Predicts Adverse Prognosis of Hepatocellular Carcinoma
Source: Front Oncol. 2019 Oct 22;9:1105. doi: 10.3389/fonc.2019.01105 (PMC6842961; doi:10.3389/fonc.2019.01105)
Supplement: Supplementary file 6 [file Table_6.DOCX]

**Table S6 The overlapping results of 60 miRNAs in three online databases.**

| miRNA | Gene | miRDB | miRTarBase | TargetScan | Sum | entrezID |
| --- | --- | --- | --- | --- | --- | --- |
| hsa-miR-7977 | ARHGEF37 | 1 | 1 | 1 | 3 | 389337 |
| hsa-miR-149-3p | MSI1 | 1 | 1 | 1 | 3 | 4440 |
| hsa-miR-4710 | EN2 | 1 | 1 | 1 | 3 | 2020 |
| hsa-miR-4508 | ASB6 | 1 | 1 | 1 | 3 | 140459 |
| hsa-miR-6883-5p | FAM83H | 1 | 1 | 1 | 3 | 286077 |
| hsa-miR-6799-5p | RAB11FIP4 | 1 | 1 | 1 | 3 | 84440 |
| hsa-miR-6883-5p | COL5A1 | 1 | 1 | 1 | 3 | 1289 |
| hsa-miR-6799-5p | SLC30A7 | 1 | 1 | 1 | 3 | 148867 |
| hsa-miR-7160-3p | RNF44 | 1 | 1 | 1 | 3 | 22838 |
| hsa-miR-4756-3p | TNRC6A | 1 | 1 | 1 | 3 | 27327 |
| hsa-miR-6883-5p | NAV1 | 1 | 1 | 1 | 3 | 89796 |
| hsa-miR-3175 | DAZAP2 | 1 | 1 | 1 | 3 | 9802 |
| hsa-miR-4728-5p | ASPH | 1 | 1 | 1 | 3 | 444 |
| hsa-miR-7106-5p | SAP18 | 1 | 1 | 1 | 3 | 10284 |
| hsa-miR-7977 | ZNF703 | 1 | 1 | 1 | 3 | 80139 |
| hsa-miR-6785-5p | NAV2 | 1 | 1 | 1 | 3 | 89797 |
| hsa-miR-4728-5p | SLC29A4 | 1 | 1 | 1 | 3 | 222962 |
| hsa-miR-4257 | NAT8L | 1 | 1 | 1 | 3 | 339983 |
| hsa-miR-7106-5p | CANX | 1 | 1 | 1 | 3 | 821 |
| hsa-miR-6825-5p | NOS1AP | 1 | 1 | 1 | 3 | 9722 |
| hsa-miR-4437 | PLAGL2 | 1 | 1 | 1 | 3 | 5326 |
| hsa-miR-4710 | GBA2 | 1 | 1 | 1 | 3 | 57704 |
| hsa-miR-6134 | CIAPIN1 | 1 | 1 | 1 | 3 | 57019 |
| hsa-miR-4512 | ALG9 | 1 | 1 | 1 | 3 | 79796 |
| hsa-miR-4728-5p | TAGLN | 1 | 1 | 1 | 3 | 6876 |
| hsa-miR-3672 | AGXT2 | 1 | 1 | 1 | 3 | 64902 |
| hsa-miR-4756-3p | GGA2 | 1 | 1 | 1 | 3 | 23062 |
| hsa-miR-6785-5p | TP53 | 1 | 1 | 1 | 3 | 7157 |
| hsa-miR-6851-5p | KLHDC3 | 1 | 1 | 1 | 3 | 116138 |
| hsa-miR-186-3p | TNFSF15 | 1 | 1 | 1 | 3 | 9966 |
| hsa-miR-6134 | DNAJC11 | 1 | 1 | 1 | 3 | 55735 |
| hsa-miR-4728-5p | PRICKLE1 | 1 | 1 | 1 | 3 | 144165 |
| hsa-miR-7977 | MARVELD2 | 1 | 1 | 1 | 3 | 153562 |
| hsa-miR-6883-5p | CPM | 1 | 1 | 1 | 3 | 1368 |
| hsa-miR-7106-5p | CRTC2 | 1 | 1 | 1 | 3 | 200186 |
| hsa-miR-4756-3p | KAT7 | 1 | 1 | 1 | 3 | 11143 |
| hsa-miR-3175 | UBTF | 1 | 1 | 1 | 3 | 7343 |
| hsa-miR-3175 | NFAT5 | 1 | 1 | 1 | 3 | 10725 |
| hsa-miR-6851-5p | RXRB | 1 | 1 | 1 | 3 | 6257 |
| hsa-miR-6799-5p | EPN1 | 1 | 1 | 1 | 3 | 29924 |
| hsa-miR-18b-3p | ANKRD52 | 1 | 1 | 1 | 3 | 283373 |
| hsa-miR-1343-5p | NFASC | 1 | 1 | 1 | 3 | 23114 |
| hsa-miR-7106-5p | OPA3 | 1 | 1 | 1 | 3 | 80207 |
| hsa-miR-6883-5p | ANKRD45 | 1 | 1 | 1 | 3 | 339416 |
| hsa-miR-4508 | PRX | 1 | 1 | 1 | 3 | 57716 |
| hsa-miR-4283 | TRIP10 | 1 | 1 | 1 | 3 | 9322 |
| hsa-miR-7977 | ATP1B4 | 1 | 1 | 1 | 3 | 23439 |
| hsa-miR-6508-3p | ITPRIP | 1 | 1 | 1 | 3 | 85450 |
| hsa-miR-4710 | DHDDS | 1 | 1 | 1 | 3 | 79947 |
| hsa-miR-6864-3p | AHCYL2 | 1 | 1 | 1 | 3 | 23382 |
| hsa-miR-642b-5p | BTG2 | 1 | 1 | 1 | 3 | 7832 |
| hsa-miR-4728-5p | KCNA5 | 1 | 1 | 1 | 3 | 3741 |
| hsa-miR-6883-5p | RNF157 | 1 | 1 | 1 | 3 | 114804 |
| hsa-miR-6775-5p | HNRNPC | 1 | 1 | 1 | 3 | 3183 |
| hsa-miR-6851-5p | MTMR10 | 1 | 1 | 1 | 3 | 54893 |
| hsa-miR-6883-5p | HOXC4 | 1 | 1 | 1 | 3 | 3221 |
| hsa-miR-6785-5p | CHRDL1 | 1 | 1 | 1 | 3 | 91851 |
| hsa-miR-6785-5p | AGBL5 | 1 | 1 | 1 | 3 | 60509 |
| hsa-miR-296-5p | MTSS1L | 1 | 1 | 1 | 3 | 92154 |
| hsa-miR-939-5p | SLC9A3R2 | 1 | 1 | 1 | 3 | 9351 |
| hsa-miR-6785-5p | NLGN2 | 1 | 1 | 1 | 3 | 57555 |
| hsa-miR-6847-5p | SDC2 | 1 | 1 | 1 | 3 | 6383 |
| hsa-miR-486-3p | PLXNA4 | 1 | 1 | 1 | 3 | 91584 |
| hsa-miR-7106-5p | MLLT1 | 1 | 1 | 1 | 3 | 4298 |
| hsa-miR-186-3p | CDK1 | 1 | 1 | 1 | 3 | 983 |
| hsa-miR-6883-5p | NRGN | 1 | 1 | 1 | 3 | 4900 |
| hsa-miR-3175 | MYL12A | 1 | 1 | 1 | 3 | 10627 |
| hsa-miR-6825-5p | EPB41L1 | 1 | 1 | 1 | 3 | 2036 |
| hsa-miR-149-3p | GCDH | 1 | 1 | 1 | 3 | 2639 |
| hsa-miR-149-3p | TBXA2R | 1 | 1 | 1 | 3 | 6915 |
| hsa-miR-6825-5p | MOB3A | 1 | 1 | 1 | 3 | 126308 |
| hsa-miR-3919 | AGO1 | 1 | 1 | 1 | 3 | 26523 |
| hsa-miR-6134 | BCL2L13 | 1 | 1 | 1 | 3 | 23786 |
| hsa-miR-6825-5p | EPN1 | 1 | 1 | 1 | 3 | 29924 |
| hsa-miR-4640-5p | OTOF | 1 | 1 | 1 | 3 | 9381 |
| hsa-miR-149-3p | WARS | 1 | 1 | 1 | 3 | 7453 |
| hsa-miR-7106-5p | RNF157 | 1 | 1 | 1 | 3 | 114804 |
| hsa-miR-4640-5p | SAMD4B | 1 | 1 | 1 | 3 | 55095 |
| hsa-miR-6883-5p | MLXIP | 1 | 1 | 1 | 3 | 22877 |
| hsa-miR-6847-5p | ZBTB4 | 1 | 1 | 1 | 3 | 57659 |
| hsa-miR-6825-5p | KIAA1161 | 1 | 1 | 1 | 3 | NA |
| hsa-miR-3175 | RANBP10 | 1 | 1 | 1 | 3 | 57610 |
| hsa-miR-4710 | AREL1 | 1 | 1 | 1 | 3 | 9870 |
| hsa-miR-149-3p | DYRK1B | 1 | 1 | 1 | 3 | 9149 |
| hsa-miR-4251 | STK11IP | 1 | 1 | 1 | 3 | 114790 |
| hsa-miR-149-3p | KCNA5 | 1 | 1 | 1 | 3 | 3741 |
| hsa-miR-6825-5p | USP22 | 1 | 1 | 1 | 3 | 23326 |
| hsa-miR-7977 | YIPF4 | 1 | 1 | 1 | 3 | 84272 |
| hsa-miR-4251 | CLIC5 | 1 | 1 | 1 | 3 | 53405 |
| hsa-miR-149-3p | RAB11B | 1 | 1 | 1 | 3 | 9230 |
| hsa-miR-939-5p | HAND2 | 1 | 1 | 1 | 3 | 9464 |
| hsa-miR-7977 | SLC35E2 | 1 | 1 | 1 | 3 | NA |
| hsa-miR-486-3p | ZFHX3 | 1 | 1 | 1 | 3 | 463 |
| hsa-miR-7106-5p | SNX1 | 1 | 1 | 1 | 3 | 6642 |
| hsa-miR-4728-5p | NEUROD2 | 1 | 1 | 1 | 3 | 4761 |
| hsa-miR-6825-5p | GATAD2A | 1 | 1 | 1 | 3 | 54815 |
| hsa-miR-149-3p | SLC29A1 | 1 | 1 | 1 | 3 | 2030 |
| hsa-miR-6804-5p | ZNF641 | 1 | 1 | 1 | 3 | 121274 |
| hsa-miR-3672 | POLI | 1 | 1 | 1 | 3 | 11201 |
| hsa-miR-6847-5p | HMGA1 | 1 | 1 | 1 | 3 | 3159 |
| hsa-miR-7106-5p | RRP7A | 1 | 1 | 1 | 3 | 27341 |
| hsa-miR-7106-5p | RPP30 | 1 | 1 | 1 | 3 | 10556 |
| hsa-miR-186-3p | SMIM7 | 1 | 1 | 1 | 3 | 79086 |
| hsa-miR-149-3p | CCL22 | 1 | 1 | 1 | 3 | 6367 |
| hsa-miR-6883-5p | PLLP | 1 | 1 | 1 | 3 | 51090 |
| hsa-miR-3925-5p | PHF12 | 1 | 1 | 1 | 3 | 57649 |
| hsa-miR-7977 | FUT2 | 1 | 1 | 1 | 3 | 2524 |
| hsa-miR-4710 | BCL2L1 | 1 | 1 | 1 | 3 | 598 |
| hsa-miR-6799-5p | RTKN | 1 | 1 | 1 | 3 | 6242 |
| hsa-miR-7977 | STK4 | 1 | 1 | 1 | 3 | 6789 |
| hsa-miR-149-3p | MLXIP | 1 | 1 | 1 | 3 | 22877 |
| hsa-miR-6883-5p | AGBL5 | 1 | 1 | 1 | 3 | 60509 |
| hsa-miR-149-3p | AKT1 | 1 | 1 | 1 | 3 | 207 |
| hsa-miR-4251 | CD300E | 1 | 1 | 1 | 3 | 342510 |
| hsa-miR-149-3p | KRT80 | 1 | 1 | 1 | 3 | 144501 |
| hsa-miR-149-3p | PPFIA3 | 1 | 1 | 1 | 3 | 8541 |
| hsa-miR-7977 | PCBD2 | 1 | 1 | 1 | 3 | 84105 |
| hsa-miR-6883-5p | SLC29A4 | 1 | 1 | 1 | 3 | 222962 |
| hsa-miR-149-3p | SREBF2 | 1 | 1 | 1 | 3 | 6721 |
| hsa-miR-6785-5p | UBE2S | 1 | 1 | 1 | 3 | 27338 |
| hsa-miR-6883-5p | NOVA2 | 1 | 1 | 1 | 3 | 4858 |
| hsa-miR-6883-5p | ZFP36L1 | 1 | 1 | 1 | 3 | 677 |
| hsa-miR-149-3p | HAP1 | 1 | 1 | 1 | 3 | 9001 |
| hsa-miR-6883-5p | CAPZB | 1 | 1 | 1 | 3 | 832 |
| hsa-miR-6825-5p | KCTD2 | 1 | 1 | 1 | 3 | 23510 |
| hsa-miR-4728-5p | PRR12 | 1 | 1 | 1 | 3 | 57479 |
| hsa-miR-6825-5p | BTG2 | 1 | 1 | 1 | 3 | 7832 |
| hsa-miR-4728-5p | RPRD2 | 1 | 1 | 1 | 3 | 23248 |
| hsa-miR-542-5p | TBR1 | 1 | 1 | 1 | 3 | 10716 |
| hsa-miR-6883-5p | CARM1 | 1 | 1 | 1 | 3 | 10498 |
| hsa-miR-6785-5p | RNF157 | 1 | 1 | 1 | 3 | 114804 |
| hsa-miR-7106-5p | PACSIN1 | 1 | 1 | 1 | 3 | 29993 |
| hsa-miR-6825-5p | YWHAZ | 1 | 1 | 1 | 3 | 7534 |
| hsa-miR-4728-5p | CDK14 | 1 | 1 | 1 | 3 | 5218 |
| hsa-miR-486-3p | UNC119B | 1 | 1 | 1 | 3 | 84747 |
| hsa-miR-6799-5p | PLAGL2 | 1 | 1 | 1 | 3 | 5326 |
| hsa-miR-6785-5p | IGFBP4 | 1 | 1 | 1 | 3 | 3487 |
| hsa-miR-6785-5p | TMEM63C | 1 | 1 | 1 | 3 | 57156 |
| hsa-miR-6847-5p | PLA2G5 | 1 | 1 | 1 | 3 | 5322 |
| hsa-miR-7854-3p | CCDC6 | 1 | 1 | 1 | 3 | 8030 |
| hsa-miR-149-3p | DPYSL5 | 1 | 1 | 1 | 3 | 56896 |
| hsa-miR-7106-5p | CSTF1 | 1 | 1 | 1 | 3 | 1477 |
| hsa-miR-1296-5p | SFPQ | 1 | 1 | 1 | 3 | 6421 |
| hsa-miR-1296-5p | HYOU1 | 1 | 1 | 1 | 3 | 10525 |
| hsa-miR-1343-5p | PGPEP1 | 1 | 1 | 1 | 3 | 54858 |
| hsa-miR-3175 | SLC26A9 | 1 | 1 | 1 | 3 | 115019 |
| hsa-miR-3919 | TNRC6A | 1 | 1 | 1 | 3 | 27327 |
| hsa-miR-6785-5p | DUSP7 | 1 | 1 | 1 | 3 | 1849 |
| hsa-miR-6785-5p | NOVA2 | 1 | 1 | 1 | 3 | 4858 |
| hsa-miR-6825-5p | ITGA3 | 1 | 1 | 1 | 3 | 3675 |
| hsa-miR-4710 | WIPF2 | 1 | 1 | 1 | 3 | 147179 |
| hsa-miR-6761-5p | PRMT8 | 1 | 1 | 1 | 3 | 56341 |
| hsa-miR-3925-5p | PAK2 | 1 | 1 | 1 | 3 | 5062 |
| hsa-miR-4728-5p | NGFR | 1 | 1 | 1 | 3 | 4804 |
| hsa-miR-7106-5p | CLSTN1 | 1 | 1 | 1 | 3 | 22883 |
| hsa-miR-6785-5p | IFFO2 | 1 | 1 | 1 | 3 | 126917 |
| hsa-miR-1291 | PPM1F | 1 | 1 | 1 | 3 | 9647 |
| hsa-miR-486-3p | POFUT2 | 1 | 1 | 1 | 3 | 23275 |
| hsa-miR-6825-5p | SLITRK5 | 1 | 1 | 1 | 3 | 26050 |
| hsa-miR-149-3p | MOB3A | 1 | 1 | 1 | 3 | 126308 |
| hsa-miR-4710 | VMA21 | 1 | 1 | 1 | 3 | 203547 |
| hsa-miR-6799-5p | SLC35E2 | 1 | 1 | 1 | 3 | NA |
| hsa-miR-4728-5p | MEX3A | 1 | 1 | 1 | 3 | 92312 |
| hsa-miR-3919 | CTDSP1 | 1 | 1 | 1 | 3 | 58190 |
| hsa-miR-4792 | RNF185 | 1 | 1 | 1 | 3 | 91445 |
| hsa-miR-939-5p | UNC13A | 1 | 1 | 1 | 3 | 23025 |
| hsa-miR-6134 | TMEM50B | 1 | 1 | 1 | 3 | 757 |
| hsa-miR-6804-5p | CDK2 | 1 | 1 | 1 | 3 | 1017 |
| hsa-miR-6825-5p | SPATA6 | 1 | 1 | 1 | 3 | 54558 |
| hsa-miR-6134 | C16orf52 | 1 | 1 | 1 | 3 | NA |
| hsa-miR-3919 | GTPBP2 | 1 | 1 | 1 | 3 | 54676 |
| hsa-miR-3123 | BLOC1S6 | 1 | 1 | 1 | 3 | 26258 |
| hsa-miR-6134 | MTMR10 | 1 | 1 | 1 | 3 | 54893 |
| hsa-miR-1178-5p | PRDM10 | 1 | 1 | 1 | 3 | 56980 |
| hsa-miR-186-3p | KIF2C | 1 | 1 | 1 | 3 | 11004 |
| hsa-miR-6799-5p | GCFC2 | 1 | 1 | 1 | 3 | 6936 |
| hsa-miR-6785-5p | SAMD10 | 1 | 1 | 1 | 3 | 140700 |
| hsa-miR-4728-5p | C10orf76 | 1 | 1 | 1 | 3 | NA |
| hsa-miR-6825-5p | NRGN | 1 | 1 | 1 | 3 | 4900 |
| hsa-miR-186-3p | EMP2 | 1 | 1 | 1 | 3 | 2013 |
| hsa-miR-6785-5p | MARK2 | 1 | 1 | 1 | 3 | 2011 |
| hsa-miR-7106-5p | TOB2 | 1 | 1 | 1 | 3 | 10766 |
| hsa-miR-6785-5p | CTDSP2 | 1 | 1 | 1 | 3 | 10106 |
| hsa-miR-1291 | SLC12A7 | 1 | 1 | 1 | 3 | 10723 |
| hsa-miR-7106-5p | CCL11 | 1 | 1 | 1 | 3 | 6356 |
| hsa-miR-6847-5p | AMOTL2 | 1 | 1 | 1 | 3 | 51421 |
| hsa-miR-6785-5p | MSI1 | 1 | 1 | 1 | 3 | 4440 |
| hsa-miR-4303 | PKP1 | 1 | 1 | 1 | 3 | 5317 |
| hsa-miR-6825-5p | TP53 | 1 | 1 | 1 | 3 | 7157 |
| hsa-miR-4251 | ZSWIM6 | 1 | 1 | 1 | 3 | 57688 |
| hsa-miR-6825-5p | RAP1GAP2 | 1 | 1 | 1 | 3 | 23108 |
| hsa-miR-4728-5p | KRT80 | 1 | 1 | 1 | 3 | 144501 |
| hsa-miR-1178-5p | MAP2K6 | 1 | 1 | 1 | 3 | 5608 |
| hsa-miR-149-3p | MNT | 1 | 1 | 1 | 3 | 4335 |
| hsa-miR-149-3p | SLC29A4 | 1 | 1 | 1 | 3 | 222962 |
| hsa-miR-6883-5p | IQSEC3 | 1 | 1 | 1 | 3 | 440073 |
| hsa-miR-4710 | HSPA4 | 1 | 1 | 1 | 3 | 3308 |
| hsa-miR-186-3p | ATCAY | 1 | 1 | 1 | 3 | 85300 |
| hsa-miR-4728-5p | ASB6 | 1 | 1 | 1 | 3 | 140459 |
| hsa-miR-149-3p | NLGN2 | 1 | 1 | 1 | 3 | 57555 |
| hsa-miR-7854-3p | PPP6R3 | 1 | 1 | 1 | 3 | 55291 |
| hsa-miR-6785-5p | PDE7A | 1 | 1 | 1 | 3 | 5150 |
| hsa-miR-7106-5p | RAP1GAP2 | 1 | 1 | 1 | 3 | 23108 |
| hsa-miR-149-3p | PIGR | 1 | 1 | 1 | 3 | 5284 |
| hsa-miR-3123 | ZNF587 | 1 | 1 | 1 | 3 | 84914 |
| hsa-miR-7106-5p | PDE4A | 1 | 1 | 1 | 3 | 5141 |
| hsa-miR-7977 | POU2F3 | 1 | 1 | 1 | 3 | 25833 |
| hsa-miR-6883-5p | PNMA2 | 1 | 1 | 1 | 3 | 10687 |
| hsa-miR-6825-5p | SREBF2 | 1 | 1 | 1 | 3 | 6721 |
| hsa-miR-6851-5p | RAP1GDS1 | 1 | 1 | 1 | 3 | 5910 |
| hsa-miR-7106-5p | ARRB1 | 1 | 1 | 1 | 3 | 408 |
| hsa-miR-6825-5p | NFASC | 1 | 1 | 1 | 3 | 23114 |
| hsa-miR-3919 | TCF7L2 | 1 | 1 | 1 | 3 | 6934 |
| hsa-miR-3919 | UBE2G1 | 1 | 1 | 1 | 3 | 7326 |
| hsa-miR-3175 | CARD10 | 1 | 1 | 1 | 3 | 29775 |
| hsa-miR-7106-5p | ARHGAP31 | 1 | 1 | 1 | 3 | 57514 |
| hsa-miR-4728-5p | MINK1 | 1 | 1 | 1 | 3 | 50488 |
| hsa-miR-7106-5p | GCNT4 | 1 | 1 | 1 | 3 | 51301 |
| hsa-miR-6825-5p | PDE4A | 1 | 1 | 1 | 3 | 5141 |
| hsa-miR-4756-3p | FAM126B | 1 | 1 | 1 | 3 | 285172 |
| hsa-miR-4728-5p | GCDH | 1 | 1 | 1 | 3 | 2639 |
| hsa-miR-3925-5p | CELF1 | 1 | 1 | 1 | 3 | 10658 |
| hsa-miR-7977 | ZNF490 | 1 | 1 | 1 | 3 | 57474 |
| hsa-miR-1343-5p | MAPRE1 | 1 | 1 | 1 | 3 | 22919 |
| hsa-miR-7106-5p | PGPEP1 | 1 | 1 | 1 | 3 | 54858 |
| hsa-miR-7977 | NCAPG2 | 1 | 1 | 1 | 3 | 54892 |
| hsa-miR-149-3p | ASXL1 | 1 | 1 | 1 | 3 | 171023 |
| hsa-miR-6825-5p | MYO1C | 1 | 1 | 1 | 3 | 4641 |
| hsa-miR-6825-5p | NAV2 | 1 | 1 | 1 | 3 | 89797 |
| hsa-miR-6785-5p | SLC29A1 | 1 | 1 | 1 | 3 | 2030 |
| hsa-miR-4728-5p | MAT1A | 1 | 1 | 1 | 3 | 4143 |
| hsa-miR-7106-5p | PAX2 | 1 | 1 | 1 | 3 | 5076 |
| hsa-miR-6851-5p | ALG8 | 1 | 1 | 1 | 3 | 79053 |
| hsa-miR-1291 | ABCC1 | 1 | 1 | 1 | 3 | 4363 |
| hsa-miR-4728-5p | CACNB1 | 1 | 1 | 1 | 3 | 782 |
| hsa-miR-7977 | UPK3BL | 1 | 1 | 1 | 3 | NA |
| hsa-miR-4283 | ARGFX | 1 | 1 | 1 | 3 | 503582 |
| hsa-miR-4728-5p | MNT | 1 | 1 | 1 | 3 | 4335 |
| hsa-miR-6851-3p | NAA15 | 1 | 1 | 1 | 3 | 80155 |
| hsa-miR-4283 | SLC27A1 | 1 | 1 | 1 | 3 | 376497 |
| hsa-miR-7106-5p | IQSEC3 | 1 | 1 | 1 | 3 | 440073 |
| hsa-miR-3123 | PRRC1 | 1 | 1 | 1 | 3 | 133619 |
| hsa-miR-6825-5p | CHST11 | 1 | 1 | 1 | 3 | 50515 |
| hsa-miR-6825-5p | FNBP1 | 1 | 1 | 1 | 3 | 23048 |
| hsa-miR-3175 | PPM1L | 1 | 1 | 1 | 3 | 151742 |
| hsa-miR-4257 | HMGA1 | 1 | 1 | 1 | 3 | 3159 |
| hsa-miR-7106-5p | XKR4 | 1 | 1 | 1 | 3 | 114786 |
| hsa-miR-6804-5p | KIF18B | 1 | 1 | 1 | 3 | 146909 |
| hsa-miR-3175 | C5orf24 | 1 | 1 | 1 | 3 | 134553 |
| hsa-miR-4512 | FAM98A | 1 | 1 | 1 | 3 | 25940 |
| hsa-miR-4257 | HMGA2 | 1 | 1 | 1 | 3 | 8091 |
| hsa-miR-6883-5p | HAP1 | 1 | 1 | 1 | 3 | 9001 |
| hsa-miR-6825-5p | NLGN2 | 1 | 1 | 1 | 3 | 57555 |
| hsa-miR-6134 | EFNB1 | 1 | 1 | 1 | 3 | 1947 |
| hsa-miR-7977 | NUP43 | 1 | 1 | 1 | 3 | 348995 |
| hsa-miR-642b-5p | RAP1B | 1 | 1 | 1 | 3 | 5908 |
| hsa-miR-7106-5p | RPRD2 | 1 | 1 | 1 | 3 | 23248 |
| hsa-miR-149-3p | SAMD10 | 1 | 1 | 1 | 3 | 140700 |
| hsa-miR-6847-5p | GPATCH8 | 1 | 1 | 1 | 3 | 23131 |
| hsa-miR-4728-5p | SCD | 1 | 1 | 1 | 3 | 6319 |
| hsa-miR-186-3p | CACNB2 | 1 | 1 | 1 | 3 | 783 |
| hsa-miR-149-3p | SCAMP4 | 1 | 1 | 1 | 3 | 113178 |
| hsa-miR-3175 | SAMD9L | 1 | 1 | 1 | 3 | 219285 |
| hsa-miR-7106-5p | KCND1 | 1 | 1 | 1 | 3 | 3750 |
| hsa-miR-6883-5p | ZFHX3 | 1 | 1 | 1 | 3 | 463 |
| hsa-miR-6785-5p | GCDH | 1 | 1 | 1 | 3 | 2639 |
| hsa-miR-6883-5p | IFFO2 | 1 | 1 | 1 | 3 | 126917 |
| hsa-miR-4728-5p | MAZ | 1 | 1 | 1 | 3 | 4150 |
| hsa-miR-149-3p | NFAT5 | 1 | 1 | 1 | 3 | 10725 |
| hsa-miR-486-3p | LY6E | 1 | 1 | 1 | 3 | 4061 |
| hsa-miR-3175 | FAM168A | 1 | 1 | 1 | 3 | 23201 |
| hsa-miR-7106-5p | ADD2 | 1 | 1 | 1 | 3 | 119 |
| hsa-miR-4467 | BRSK2 | 1 | 1 | 1 | 3 | 9024 |
| hsa-miR-6075 | KIF2C | 1 | 1 | 1 | 3 | 11004 |
| hsa-miR-7977 | PGAM5 | 1 | 1 | 1 | 3 | 192111 |
| hsa-miR-642b-5p | ZFHX3 | 1 | 1 | 1 | 3 | 463 |
| hsa-miR-6883-5p | IER5 | 1 | 1 | 1 | 3 | 51278 |
| hsa-miR-4728-5p | CALR | 1 | 1 | 1 | 3 | 811 |
| hsa-miR-6883-5p | MAZ | 1 | 1 | 1 | 3 | 4150 |
| hsa-miR-6883-5p | PARP11 | 1 | 1 | 1 | 3 | 57097 |
| hsa-miR-7977 | APOBEC3F | 1 | 1 | 1 | 3 | 200316 |
| hsa-miR-1343-5p | USF2 | 1 | 1 | 1 | 3 | 7392 |
| hsa-miR-18b-3p | CTDSPL2 | 1 | 1 | 1 | 3 | 51496 |
| hsa-miR-149-3p | CALR | 1 | 1 | 1 | 3 | 811 |
| hsa-miR-149-3p | GPR173 | 1 | 1 | 1 | 3 | 54328 |
| hsa-miR-3919 | G3BP2 | 1 | 1 | 1 | 3 | 9908 |
| hsa-miR-6799-5p | IGFBP4 | 1 | 1 | 1 | 3 | 3487 |
| hsa-miR-6883-5p | KLHL21 | 1 | 1 | 1 | 3 | 9903 |
| hsa-miR-3919 | IGF1R | 1 | 1 | 1 | 3 | 3480 |
| hsa-miR-7106-5p | DISC1 | 1 | 1 | 1 | 3 | 27185 |
| hsa-miR-4251 | TSC22D2 | 1 | 1 | 1 | 3 | 9819 |
| hsa-miR-6785-5p | KHSRP | 1 | 1 | 1 | 3 | 8570 |
| hsa-miR-149-3p | ATP2A3 | 1 | 1 | 1 | 3 | 489 |
| hsa-miR-6753-3p | LHFPL2 | 1 | 1 | 1 | 3 | 10184 |
| hsa-miR-6785-5p | RAD51B | 1 | 1 | 1 | 3 | 5890 |
| hsa-miR-6825-5p | UNC13A | 1 | 1 | 1 | 3 | 23025 |
| hsa-miR-3175 | HOXC6 | 1 | 1 | 1 | 3 | 3223 |
| hsa-miR-146b-3p | TSC22D2 | 1 | 1 | 1 | 3 | 9819 |
| hsa-miR-7977 | HAUS5 | 1 | 1 | 1 | 3 | 23354 |
| hsa-miR-6847-5p | COX6B1 | 1 | 1 | 1 | 3 | 1340 |
| hsa-miR-4728-5p | ANKRD45 | 1 | 1 | 1 | 3 | 339416 |
| hsa-miR-6883-5p | C10orf76 | 1 | 1 | 1 | 3 | NA |
| hsa-miR-7106-5p | PDE7B | 1 | 1 | 1 | 3 | 27115 |
| hsa-miR-4728-5p | PKNOX2 | 1 | 1 | 1 | 3 | 63876 |
| hsa-miR-6851-5p | C19orf43 | 1 | 1 | 1 | 3 | NA |
| hsa-miR-3123 | NUBP1 | 1 | 1 | 1 | 3 | 4682 |
| hsa-miR-6775-3p | PPM1F | 1 | 1 | 1 | 3 | 9647 |
| hsa-miR-6825-5p | PHYHIP | 1 | 1 | 1 | 3 | 9796 |
| hsa-miR-939-5p | USF2 | 1 | 1 | 1 | 3 | 7392 |
| hsa-miR-6785-5p | ASB6 | 1 | 1 | 1 | 3 | 140459 |
| hsa-miR-4728-5p | FBXO45 | 1 | 1 | 1 | 3 | 200933 |
| hsa-miR-6799-5p | PGM2L1 | 1 | 1 | 1 | 3 | 283209 |
| hsa-miR-1343-5p | MARK2 | 1 | 1 | 1 | 3 | 2011 |
| hsa-miR-4728-5p | HOXC4 | 1 | 1 | 1 | 3 | 3221 |
| hsa-miR-6895-5p | TNFAIP1 | 1 | 1 | 1 | 3 | 7126 |
| hsa-miR-4283 | TIMM8A | 1 | 1 | 1 | 3 | 1678 |
| hsa-miR-6785-5p | MINK1 | 1 | 1 | 1 | 3 | 50488 |
| hsa-miR-7977 | TOX4 | 1 | 1 | 1 | 3 | 9878 |
| hsa-miR-4710 | ZNF641 | 1 | 1 | 1 | 3 | 121274 |
| hsa-miR-7854-3p | RECK | 1 | 1 | 1 | 3 | 8434 |
| hsa-miR-7106-5p | BTF3L4 | 1 | 1 | 1 | 3 | 91408 |
| hsa-miR-6883-5p | MNT | 1 | 1 | 1 | 3 | 4335 |
| hsa-miR-3175 | PRRT2 | 1 | 1 | 1 | 3 | 112476 |
| hsa-miR-4728-5p | PLAGL2 | 1 | 1 | 1 | 3 | 5326 |
| hsa-miR-4728-5p | FEM1A | 1 | 1 | 1 | 3 | 55527 |
| hsa-miR-6785-5p | UBL4A | 1 | 1 | 1 | 3 | 8266 |
| hsa-miR-6825-5p | ANKRD45 | 1 | 1 | 1 | 3 | 339416 |
| hsa-miR-939-5p | MARK2 | 1 | 1 | 1 | 3 | 2011 |
| hsa-miR-6825-5p | BCAM | 1 | 1 | 1 | 3 | 4059 |
| hsa-miR-3123 | ARF3 | 1 | 1 | 1 | 3 | 377 |
| hsa-miR-6883-5p | SSBP2 | 1 | 1 | 1 | 3 | 23635 |
| hsa-miR-486-3p | UST | 1 | 1 | 1 | 3 | 10090 |
| hsa-miR-6785-5p | ASXL1 | 1 | 1 | 1 | 3 | 171023 |
| hsa-miR-4728-5p | SAMD10 | 1 | 1 | 1 | 3 | 140700 |
| hsa-miR-7977 | FGFBP3 | 1 | 1 | 1 | 3 | 143282 |
| hsa-miR-6825-5p | IER5 | 1 | 1 | 1 | 3 | 51278 |
| hsa-miR-4728-5p | ANKRD40 | 1 | 1 | 1 | 3 | 91369 |
| hsa-miR-7977 | ACVR1 | 1 | 1 | 1 | 3 | 90 |
| hsa-miR-486-3p | SLC4A2 | 1 | 1 | 1 | 3 | 6522 |
| hsa-miR-4756-3p | ZBTB22 | 1 | 1 | 1 | 3 | 9278 |
| hsa-miR-3919 | MAPRE1 | 1 | 1 | 1 | 3 | 22919 |
| hsa-miR-4728-5p | ARHGAP31 | 1 | 1 | 1 | 3 | 57514 |
| hsa-miR-6883-5p | MINK1 | 1 | 1 | 1 | 3 | 50488 |
| hsa-miR-6825-5p | KBTBD3 | 1 | 1 | 1 | 3 | 143879 |
| hsa-miR-149-3p | ZFP36L1 | 1 | 1 | 1 | 3 | 677 |
| hsa-miR-4728-5p | KIAA1328 | 1 | 1 | 1 | 3 | 57536 |
| hsa-miR-186-3p | ZNF462 | 1 | 1 | 1 | 3 | 58499 |
| hsa-miR-6825-5p | RASGRP3 | 1 | 1 | 1 | 3 | 25780 |
| hsa-miR-4728-5p | SMTNL2 | 1 | 1 | 1 | 3 | 342527 |
| hsa-miR-486-3p | KMT2D | 1 | 1 | 1 | 3 | 8085 |
| hsa-miR-6889-5p | CALR | 1 | 1 | 1 | 3 | 811 |
| hsa-miR-296-5p | GNB2 | 1 | 1 | 1 | 3 | 2783 |
| hsa-miR-4728-5p | KHSRP | 1 | 1 | 1 | 3 | 8570 |
| hsa-miR-6825-5p | CASZ1 | 1 | 1 | 1 | 3 | 54897 |
| hsa-miR-4728-5p | SYNGR1 | 1 | 1 | 1 | 3 | 9145 |
| hsa-miR-149-3p | SLITRK5 | 1 | 1 | 1 | 3 | 26050 |
| hsa-miR-6785-5p | DESI1 | 1 | 1 | 1 | 3 | 27351 |
| hsa-miR-6799-5p | DPH2 | 1 | 1 | 1 | 3 | 1802 |
| hsa-miR-7106-5p | SHISA6 | 1 | 1 | 1 | 3 | 388336 |
| hsa-miR-149-3p | IER5 | 1 | 1 | 1 | 3 | 51278 |
| hsa-miR-149-3p | IKZF3 | 1 | 1 | 1 | 3 | 22806 |
| hsa-miR-6785-5p | RASD1 | 1 | 1 | 1 | 3 | 51655 |
| hsa-miR-7106-5p | ATF6 | 1 | 1 | 1 | 3 | 22926 |
| hsa-miR-149-3p | DISC1 | 1 | 1 | 1 | 3 | 27185 |
| hsa-miR-149-3p | ASB6 | 1 | 1 | 1 | 3 | 140459 |
| hsa-miR-6799-5p | DDN | 1 | 1 | 1 | 3 | 23109 |
| hsa-miR-6785-5p | C10orf76 | 1 | 1 | 1 | 3 | NA |
| hsa-miR-149-3p | IQSEC3 | 1 | 1 | 1 | 3 | 440073 |
| hsa-miR-149-3p | PLEKHG2 | 1 | 1 | 1 | 3 | 64857 |
| hsa-miR-6883-5p | TP53 | 1 | 1 | 1 | 3 | 7157 |
| hsa-miR-6883-5p | ZNF491 | 1 | 1 | 1 | 3 | 126069 |
| hsa-miR-7854-3p | MTFR1 | 1 | 1 | 1 | 3 | 9650 |
| hsa-miR-149-3p | DESI1 | 1 | 1 | 1 | 3 | 27351 |
| hsa-miR-3925-5p | SKA2 | 1 | 1 | 1 | 3 | 348235 |
| hsa-miR-149-3p | FBXO45 | 1 | 1 | 1 | 3 | 200933 |
| hsa-miR-4303 | UBE2Z | 1 | 1 | 1 | 3 | 65264 |
| hsa-miR-6785-5p | BTG2 | 1 | 1 | 1 | 3 | 7832 |
| hsa-miR-146b-3p | CPEB4 | 1 | 1 | 1 | 3 | 80315 |
| hsa-miR-149-3p | PLEKHH1 | 1 | 1 | 1 | 3 | 57475 |
| hsa-miR-3919 | UNC119B | 1 | 1 | 1 | 3 | 84747 |
| hsa-miR-6883-5p | KIAA1328 | 1 | 1 | 1 | 3 | 57536 |
| hsa-miR-7977 | CCL22 | 1 | 1 | 1 | 3 | 6367 |
| hsa-miR-6134 | TRAPPC2 | 1 | 1 | 1 | 3 | 6399 |
| hsa-miR-4251 | TMEM101 | 1 | 1 | 1 | 3 | 84336 |
| hsa-miR-6883-5p | PLEKHG2 | 1 | 1 | 1 | 3 | 64857 |
| hsa-miR-3672 | CSTF2T | 1 | 1 | 1 | 3 | 23283 |
| hsa-miR-149-3p | CACNB1 | 1 | 1 | 1 | 3 | 782 |
| hsa-miR-7977 | ZBTB3 | 1 | 1 | 1 | 3 | 79842 |
| hsa-miR-6883-5p | SREBF2 | 1 | 1 | 1 | 3 | 6721 |
| hsa-miR-4728-5p | STK11 | 1 | 1 | 1 | 3 | 6794 |
| hsa-miR-6804-5p | MORC4 | 1 | 1 | 1 | 3 | 79710 |
| hsa-miR-6883-5p | RASD1 | 1 | 1 | 1 | 3 | 51655 |
| hsa-miR-6825-5p | ZBTB7A | 1 | 1 | 1 | 3 | 51341 |
| hsa-miR-6785-5p | PPFIA3 | 1 | 1 | 1 | 3 | 8541 |
| hsa-miR-6825-5p | SORCS2 | 1 | 1 | 1 | 3 | 57537 |
| hsa-miR-7106-5p | ZNF451 | 1 | 1 | 1 | 3 | 26036 |
| hsa-miR-6883-5p | FBXO45 | 1 | 1 | 1 | 3 | 200933 |
| hsa-miR-4728-5p | PLLP | 1 | 1 | 1 | 3 | 51090 |
| hsa-miR-6883-5p | NEUROD2 | 1 | 1 | 1 | 3 | 4761 |
| hsa-miR-149-3p | UBE2S | 1 | 1 | 1 | 3 | 27338 |
| hsa-miR-4728-5p | STRN4 | 1 | 1 | 1 | 3 | 29888 |
| hsa-miR-4640-5p | MLLT1 | 1 | 1 | 1 | 3 | 4298 |
| hsa-miR-6883-5p | ZCCHC8 | 1 | 1 | 1 | 3 | 55596 |
| hsa-miR-4728-5p | SLITRK5 | 1 | 1 | 1 | 3 | 26050 |
| hsa-miR-3175 | LIMD2 | 1 | 1 | 1 | 3 | 80774 |
| hsa-miR-7106-5p | MOB1B | 1 | 1 | 1 | 3 | 92597 |
| hsa-miR-589-5p | NUAK2 | 1 | 1 | 1 | 3 | 81788 |
| hsa-miR-486-3p | PKD1 | 1 | 1 | 1 | 3 | 5310 |
| hsa-miR-186-3p | ARID5B | 1 | 1 | 1 | 3 | 84159 |
| hsa-miR-3175 | TFAP2B | 1 | 1 | 1 | 3 | 7021 |
| hsa-miR-6825-5p | RAC1 | 1 | 1 | 1 | 3 | 5879 |
| hsa-miR-7977 | DYNLL2 | 1 | 1 | 1 | 3 | 140735 |
| hsa-miR-6883-5p | CHRDL1 | 1 | 1 | 1 | 3 | 91851 |
| hsa-miR-4303 | MTRNR2L6 | 1 | 1 | 1 | 3 | 100463482 |
| hsa-miR-486-3p | CAD | 1 | 1 | 1 | 3 | 790 |
| hsa-miR-3123 | BASP1 | 1 | 1 | 1 | 3 | 10409 |
| hsa-miR-193a-5p | RBBP6 | 1 | 1 | 1 | 3 | 5930 |
| hsa-miR-4251 | MRVI1 | 1 | 1 | 1 | 3 | 10335 |
| hsa-miR-149-3p | NRGN | 1 | 1 | 1 | 3 | 4900 |
| hsa-miR-3925-5p | USP25 | 1 | 1 | 1 | 3 | 29761 |
| hsa-miR-3175 | C12orf49 | 1 | 1 | 1 | 3 | 79794 |
| hsa-miR-4508 | TOR4A | 1 | 1 | 1 | 3 | 54863 |
| hsa-miR-149-3p | PLLP | 1 | 1 | 1 | 3 | 51090 |
| hsa-miR-149-3p | IGFBP4 | 1 | 1 | 1 | 3 | 3487 |
| hsa-miR-6825-5p | PHF8 | 1 | 1 | 1 | 3 | 23133 |
| hsa-miR-4728-5p | MSI1 | 1 | 1 | 1 | 3 | 4440 |
| hsa-miR-296-5p | SOX12 | 1 | 1 | 1 | 3 | 6666 |
| hsa-miR-6864-3p | ZNF443 | 1 | 1 | 1 | 3 | 10224 |
| hsa-miR-7106-5p | USB1 | 1 | 1 | 1 | 3 | 79650 |
| hsa-miR-149-3p | PRR12 | 1 | 1 | 1 | 3 | 57479 |
| hsa-miR-6883-5p | TJP3 | 1 | 1 | 1 | 3 | 27134 |
| hsa-miR-6825-5p | DLGAP3 | 1 | 1 | 1 | 3 | 58512 |
| hsa-miR-149-3p | STK35 | 1 | 1 | 1 | 3 | 140901 |
| hsa-miR-6847-5p | MPDU1 | 1 | 1 | 1 | 3 | 9526 |
| hsa-miR-7977 | HSPA1B | 1 | 1 | 1 | 3 | 3304 |
| hsa-miR-296-5p | GIGYF1 | 1 | 1 | 1 | 3 | 64599 |
| hsa-miR-4283 | HDGF | 1 | 1 | 1 | 3 | 3068 |
| hsa-miR-4251 | KLHL32 | 1 | 1 | 1 | 3 | 114792 |
| hsa-miR-486-3p | HECTD3 | 1 | 1 | 1 | 3 | 79654 |
| hsa-miR-6785-5p | TET3 | 1 | 1 | 1 | 3 | 200424 |
| hsa-miR-4640-5p | TJAP1 | 1 | 1 | 1 | 3 | 93643 |
| hsa-miR-4508 | ARHGAP40 | 1 | 1 | 1 | 3 | 343578 |
| hsa-miR-6825-5p | GPR173 | 1 | 1 | 1 | 3 | 54328 |
| hsa-miR-6785-5p | PNMA2 | 1 | 1 | 1 | 3 | 10687 |
| hsa-miR-4728-5p | NCOR2 | 1 | 1 | 1 | 3 | 9612 |
| hsa-miR-6825-5p | HDAC5 | 1 | 1 | 1 | 3 | 10014 |
| hsa-miR-6883-5p | PIGR | 1 | 1 | 1 | 3 | 5284 |
| hsa-miR-6825-5p | CBX8 | 1 | 1 | 1 | 3 | 57332 |
| hsa-miR-6847-5p | FABP3 | 1 | 1 | 1 | 3 | 2170 |
| hsa-miR-6508-3p | SFPQ | 1 | 1 | 1 | 3 | 6421 |
| hsa-miR-6825-5p | OGG1 | 1 | 1 | 1 | 3 | 4968 |
| hsa-miR-486-3p | CASKIN1 | 1 | 1 | 1 | 3 | 57524 |
| hsa-miR-186-3p | HIP1 | 1 | 1 | 1 | 3 | 3092 |
| hsa-miR-6883-5p | ATP2A3 | 1 | 1 | 1 | 3 | 489 |
| hsa-miR-7977 | ADAMTS4 | 1 | 1 | 1 | 3 | 9507 |
| hsa-miR-6825-5p | PFN1 | 1 | 1 | 1 | 3 | 5216 |
| hsa-miR-6883-5p | RPRD2 | 1 | 1 | 1 | 3 | 23248 |
| hsa-miR-6134 | FBXL18 | 1 | 1 | 1 | 3 | 80028 |
| hsa-miR-6134 | ARIH2 | 1 | 1 | 1 | 3 | 10425 |
| hsa-miR-6864-3p | CCSER2 | 1 | 1 | 1 | 3 | 54462 |
| hsa-miR-6785-5p | NAV1 | 1 | 1 | 1 | 3 | 89796 |
| hsa-miR-6761-5p | APIP | 1 | 1 | 1 | 3 | 51074 |
| hsa-miR-4303 | CAMSAP1 | 1 | 1 | 1 | 3 | 157922 |
| hsa-miR-6799-5p | ZNF70 | 1 | 1 | 1 | 3 | 7621 |
| hsa-miR-6775-3p | IQSEC3 | 1 | 1 | 1 | 3 | 440073 |
| hsa-miR-3672 | AHCYL2 | 1 | 1 | 1 | 3 | 23382 |
| hsa-miR-7106-5p | EFNA3 | 1 | 1 | 1 | 3 | 1944 |
| hsa-miR-186-3p | NUFIP2 | 1 | 1 | 1 | 3 | 57532 |
| hsa-miR-4251 | POLR1D | 1 | 1 | 1 | 3 | 51082 |
| hsa-miR-6785-5p | MKNK2 | 1 | 1 | 1 | 3 | 2872 |
| hsa-miR-4251 | MYL12B | 1 | 1 | 1 | 3 | 103910 |
| hsa-miR-6883-5p | WNT7B | 1 | 1 | 1 | 3 | 7477 |
| hsa-miR-486-3p | AP1G1 | 1 | 1 | 1 | 3 | 164 |
| hsa-miR-4710 | RNF126 | 1 | 1 | 1 | 3 | 55658 |
| hsa-miR-3123 | NRAS | 1 | 1 | 1 | 3 | 4893 |
| hsa-miR-4728-5p | SSBP2 | 1 | 1 | 1 | 3 | 23635 |
| hsa-miR-6134 | ARID1A | 1 | 1 | 1 | 3 | 8289 |
| hsa-miR-6777-5p | PLIN1 | 1 | 1 | 1 | 3 | 5346 |
| hsa-miR-6799-5p | ZNF548 | 1 | 1 | 1 | 3 | 147694 |
| hsa-miR-149-3p | MINK1 | 1 | 1 | 1 | 3 | 50488 |
| hsa-miR-4329 | NFAM1 | 1 | 1 | 1 | 3 | 150372 |
| hsa-miR-7106-5p | RPP25 | 1 | 1 | 1 | 3 | 54913 |
| hsa-miR-4640-5p | CNNM4 | 1 | 1 | 1 | 3 | 26504 |
| hsa-miR-486-3p | OGT | 1 | 1 | 1 | 3 | 8473 |
| hsa-miR-149-3p | NOVA2 | 1 | 1 | 1 | 3 | 4858 |
| hsa-miR-6785-5p | TBXA2R | 1 | 1 | 1 | 3 | 6915 |
| hsa-miR-6883-5p | MGAT5B | 1 | 1 | 1 | 3 | 146664 |
| hsa-miR-7106-5p | ASB1 | 1 | 1 | 1 | 3 | 51665 |
| hsa-miR-7977 | LRRC47 | 1 | 1 | 1 | 3 | 57470 |
| hsa-miR-4728-5p | DUSP7 | 1 | 1 | 1 | 3 | 1849 |
| hsa-miR-3123 | CALM1 | 1 | 1 | 1 | 3 | 801 |
| hsa-miR-6134 | LLGL1 | 1 | 1 | 1 | 3 | 3996 |
| hsa-miR-7106-5p | ACVR1B | 1 | 1 | 1 | 3 | 91 |
| hsa-miR-6883-5p | KRT80 | 1 | 1 | 1 | 3 | 144501 |
| hsa-miR-3919 | SET | 1 | 1 | 1 | 3 | 6418 |
| hsa-miR-6804-5p | PKM | 1 | 1 | 1 | 3 | 5315 |
| hsa-miR-6785-5p | ARL5B | 1 | 1 | 1 | 3 | 221079 |
| hsa-miR-6883-5p | STRN4 | 1 | 1 | 1 | 3 | 29888 |
| hsa-miR-7977 | MTMR14 | 1 | 1 | 1 | 3 | 64419 |
| hsa-miR-6825-5p | LENG8 | 1 | 1 | 1 | 3 | 114823 |
| hsa-miR-7160-3p | GNG3 | 1 | 1 | 1 | 3 | 2785 |
| hsa-miR-7977 | APOL6 | 1 | 1 | 1 | 3 | 80830 |
| hsa-miR-6883-5p | MLLT1 | 1 | 1 | 1 | 3 | 4298 |
| hsa-miR-6785-5p | NGFR | 1 | 1 | 1 | 3 | 4804 |
| hsa-miR-3919 | DHX36 | 1 | 1 | 1 | 3 | 170506 |
| hsa-miR-7106-5p | UBXN2B | 1 | 1 | 1 | 3 | 137886 |
| hsa-miR-6825-5p | VWA1 | 1 | 1 | 1 | 3 | 64856 |
| hsa-miR-6883-5p | ITGA3 | 1 | 1 | 1 | 3 | 3675 |
| hsa-miR-296-5p | NFIC | 1 | 1 | 1 | 3 | 4782 |
| hsa-miR-4710 | PALM2 | 1 | 1 | 1 | 3 | 114299 |
| hsa-miR-939-5p | GNAI2 | 1 | 1 | 1 | 3 | 2771 |
| hsa-miR-6825-5p | CALML3 | 1 | 1 | 1 | 3 | 810 |
| hsa-miR-6785-5p | CARM1 | 1 | 1 | 1 | 3 | 10498 |
| hsa-miR-4728-5p | CAPZB | 1 | 1 | 1 | 3 | 832 |
| hsa-miR-4756-3p | PPP2R1B | 1 | 1 | 1 | 3 | 5519 |
| hsa-miR-6847-5p | PHF19 | 1 | 1 | 1 | 3 | 26147 |
| hsa-miR-6883-5p | MARK2 | 1 | 1 | 1 | 3 | 2011 |
| hsa-miR-6761-5p | ZNF260 | 1 | 1 | 1 | 3 | 339324 |
| hsa-miR-4728-5p | AMER1 | 1 | 1 | 1 | 3 | 139285 |
| hsa-miR-6851-5p | HIRIP3 | 1 | 1 | 1 | 3 | 8479 |
| hsa-miR-6883-5p | ARL5B | 1 | 1 | 1 | 3 | 221079 |
| hsa-miR-146b-3p | CPS1 | 1 | 1 | 1 | 3 | 1373 |
| hsa-miR-4257 | GRPEL2 | 1 | 1 | 1 | 3 | 134266 |
| hsa-miR-4728-5p | MKNK2 | 1 | 1 | 1 | 3 | 2872 |
| hsa-miR-7977 | WDR55 | 1 | 1 | 1 | 3 | 54853 |
| hsa-miR-4283 | PLAGL2 | 1 | 1 | 1 | 3 | 5326 |
| hsa-miR-146b-3p | MTFR1L | 1 | 1 | 1 | 3 | 56181 |
| hsa-miR-4728-5p | TMEM63C | 1 | 1 | 1 | 3 | 57156 |
| hsa-miR-4728-5p | CARM1 | 1 | 1 | 1 | 3 | 10498 |
| hsa-miR-6785-5p | NFIX | 1 | 1 | 1 | 3 | 4784 |
| hsa-miR-4728-5p | PIGR | 1 | 1 | 1 | 3 | 5284 |
| hsa-miR-149-3p | IFFO2 | 1 | 1 | 1 | 3 | 126917 |
| hsa-miR-1343-5p | UNC13A | 1 | 1 | 1 | 3 | 23025 |
| hsa-miR-6134 | KRT80 | 1 | 1 | 1 | 3 | 144501 |
| hsa-miR-4728-5p | NAV2 | 1 | 1 | 1 | 3 | 89797 |
| hsa-miR-6825-5p | SLC4A2 | 1 | 1 | 1 | 3 | 6522 |
| hsa-miR-6785-5p | PLEKHG2 | 1 | 1 | 1 | 3 | 64857 |
| hsa-miR-4728-5p | ZFHX3 | 1 | 1 | 1 | 3 | 463 |
| hsa-miR-186-3p | ATAD2B | 1 | 1 | 1 | 3 | 54454 |
| hsa-miR-3123 | C21orf91 | 1 | 1 | 1 | 3 | 54149 |
| hsa-miR-3919 | OPHN1 | 1 | 1 | 1 | 3 | 4983 |
| hsa-miR-6883-5p | SLITRK5 | 1 | 1 | 1 | 3 | 26050 |
| hsa-miR-7977 | CSRNP3 | 1 | 1 | 1 | 3 | 80034 |
| hsa-miR-3123 | SPTY2D1 | 1 | 1 | 1 | 3 | 144108 |
| hsa-miR-4728-5p | NOVA2 | 1 | 1 | 1 | 3 | 4858 |
| hsa-miR-6825-5p | MARK2 | 1 | 1 | 1 | 3 | 2011 |
| hsa-miR-7977 | PGPEP1 | 1 | 1 | 1 | 3 | 54858 |
| hsa-miR-4251 | KCTD15 | 1 | 1 | 1 | 3 | 79047 |
| hsa-miR-6851-5p | EIF4A1 | 1 | 1 | 1 | 3 | 1973 |
| hsa-miR-6847-5p | C7orf55-LUC7L2 | 1 | 1 | 1 | 3 | 100996928 |
| hsa-miR-6799-5p | LRRC20 | 1 | 1 | 1 | 3 | 55222 |
| hsa-miR-4251 | FEN1 | 1 | 1 | 1 | 3 | 2237 |
| hsa-miR-4257 | COX10 | 1 | 1 | 1 | 3 | 1352 |
| hsa-miR-4283 | RPS6KA4 | 1 | 1 | 1 | 3 | 8986 |
| hsa-miR-486-3p | MEN1 | 1 | 1 | 1 | 3 | 4221 |
| hsa-miR-6785-5p | KIAA1328 | 1 | 1 | 1 | 3 | 57536 |
| hsa-miR-4756-3p | HCFC2 | 1 | 1 | 1 | 3 | 29915 |
| hsa-miR-6799-5p | CRY2 | 1 | 1 | 1 | 3 | 1408 |
| hsa-miR-149-3p | ARL5B | 1 | 1 | 1 | 3 | 221079 |
| hsa-miR-6799-5p | HIC2 | 1 | 1 | 1 | 3 | 23119 |
| hsa-miR-4756-3p | POLE3 | 1 | 1 | 1 | 3 | 54107 |
| hsa-miR-6134 | SCAMP3 | 1 | 1 | 1 | 3 | 10067 |
| hsa-miR-7977 | POLR2J3 | 1 | 1 | 1 | 3 | 548644 |
| hsa-miR-149-3p | ANKRD45 | 1 | 1 | 1 | 3 | 339416 |
| hsa-miR-4728-5p | ZCCHC3 | 1 | 1 | 1 | 3 | 85364 |
| hsa-miR-6864-3p | UBE2D3 | 1 | 1 | 1 | 3 | 7323 |
| hsa-miR-6883-5p | RNF111 | 1 | 1 | 1 | 3 | 54778 |
| hsa-miR-149-3p | ARL8A | 1 | 1 | 1 | 3 | 127829 |
| hsa-miR-4728-5p | ZNF491 | 1 | 1 | 1 | 3 | 126069 |
| hsa-miR-3919 | PRMT6 | 1 | 1 | 1 | 3 | 55170 |
| hsa-miR-4728-5p | ITGA3 | 1 | 1 | 1 | 3 | 3675 |
| hsa-miR-7977 | SLC25A34 | 1 | 1 | 1 | 3 | 284723 |
| hsa-miR-6825-5p | URM1 | 1 | 1 | 1 | 3 | 81605 |
| hsa-miR-149-3p | ZCCHC3 | 1 | 1 | 1 | 3 | 85364 |
| hsa-miR-6804-5p | HSPA4 | 1 | 1 | 1 | 3 | 3308 |
| hsa-miR-4728-5p | ARL5B | 1 | 1 | 1 | 3 | 221079 |
| hsa-miR-6825-5p | C5orf38 | 1 | 1 | 1 | 3 | 153571 |
| hsa-miR-7854-3p | DDN | 1 | 1 | 1 | 3 | 23109 |
| hsa-miR-6825-5p | NFIX | 1 | 1 | 1 | 3 | 4784 |
| hsa-miR-6134 | FAM161B | 1 | 1 | 1 | 3 | 145483 |
| hsa-miR-486-3p | RNF41 | 1 | 1 | 1 | 3 | 10193 |
| hsa-miR-4728-5p | MYH9 | 1 | 1 | 1 | 3 | 4627 |
| hsa-miR-149-3p | CARM1 | 1 | 1 | 1 | 3 | 10498 |
| hsa-miR-6134 | CRTAP | 1 | 1 | 1 | 3 | 10491 |
| hsa-miR-3919 | USP5 | 1 | 1 | 1 | 3 | 8078 |
| hsa-miR-7106-5p | SLC35F6 | 1 | 1 | 1 | 3 | 54978 |
| hsa-miR-146b-3p | BLCAP | 1 | 1 | 1 | 3 | 10904 |
| hsa-miR-4437 | GDE1 | 1 | 1 | 1 | 3 | 51573 |
| hsa-miR-4710 | EDN1 | 1 | 1 | 1 | 3 | 1906 |
| hsa-miR-6883-5p | DESI1 | 1 | 1 | 1 | 3 | 27351 |
| hsa-miR-186-3p | KIAA1549 | 1 | 1 | 1 | 3 | 57670 |
| hsa-miR-6825-5p | KCND1 | 1 | 1 | 1 | 3 | 3750 |
| hsa-miR-7977 | APOL2 | 1 | 1 | 1 | 3 | 23780 |
| hsa-miR-4251 | BACH1 | 1 | 1 | 1 | 3 | 571 |
| hsa-miR-6785-5p | IQSEC3 | 1 | 1 | 1 | 3 | 440073 |
| hsa-miR-6847-5p | CDK2 | 1 | 1 | 1 | 3 | 1017 |
| hsa-miR-6825-5p | NEUROD2 | 1 | 1 | 1 | 3 | 4761 |
| hsa-miR-4303 | PAFAH2 | 1 | 1 | 1 | 3 | 5051 |
| hsa-miR-486-3p | TAGLN | 1 | 1 | 1 | 3 | 6876 |
| hsa-miR-3919 | MTUS1 | 1 | 1 | 1 | 3 | 57509 |
| hsa-miR-3919 | CCDC174 | 1 | 1 | 1 | 3 | 51244 |
| hsa-miR-486-3p | NACC2 | 1 | 1 | 1 | 3 | 138151 |
| hsa-miR-7106-5p | MAPK1IP1L | 1 | 1 | 1 | 3 | 93487 |
| hsa-miR-6785-5p | ARHGAP31 | 1 | 1 | 1 | 3 | 57514 |
| hsa-miR-7977 | ZNF557 | 1 | 1 | 1 | 3 | 79230 |
| hsa-miR-4728-5p | DPYSL5 | 1 | 1 | 1 | 3 | 56896 |
| hsa-miR-149-3p | DUSP7 | 1 | 1 | 1 | 3 | 1849 |
| hsa-miR-6895-5p | ITM2C | 1 | 1 | 1 | 3 | 81618 |
| hsa-miR-6785-5p | PRICKLE1 | 1 | 1 | 1 | 3 | 144165 |
| hsa-miR-6883-5p | CTDSP2 | 1 | 1 | 1 | 3 | 10106 |
| hsa-miR-6883-5p | C20orf96 | 1 | 1 | 1 | 3 | 140680 |
| hsa-miR-4251 | SLC2A14 | 1 | 1 | 1 | 3 | 144195 |
| hsa-miR-6785-5p | ATP2A3 | 1 | 1 | 1 | 3 | 489 |
| hsa-miR-6883-5p | PITPNA | 1 | 1 | 1 | 3 | 5306 |
| hsa-miR-149-3p | CTDSP2 | 1 | 1 | 1 | 3 | 10106 |
| hsa-miR-4640-5p | ITM2C | 1 | 1 | 1 | 3 | 81618 |
| hsa-miR-3175 | RAB11B | 1 | 1 | 1 | 3 | 9230 |
| hsa-miR-7160-3p | NETO2 | 1 | 1 | 1 | 3 | 81831 |
| hsa-miR-3919 | WIPF2 | 1 | 1 | 1 | 3 | 147179 |
| hsa-miR-6825-5p | RNF157 | 1 | 1 | 1 | 3 | 114804 |
| hsa-miR-486-3p | MTMR12 | 1 | 1 | 1 | 3 | 54545 |
| hsa-miR-4728-5p | CNOT6L | 1 | 1 | 1 | 3 | 246175 |
| hsa-miR-6883-5p | TMTC1 | 1 | 1 | 1 | 3 | 83857 |
| hsa-miR-4437 | SNCG | 1 | 1 | 1 | 3 | 6623 |
| hsa-miR-3175 | C6orf106 | 1 | 1 | 1 | 3 | 64771 |
| hsa-miR-6785-5p | KLHL21 | 1 | 1 | 1 | 3 | 9903 |
| hsa-miR-6883-5p | SAMD10 | 1 | 1 | 1 | 3 | 140700 |
| hsa-miR-6895-5p | KIF5B | 1 | 1 | 1 | 3 | 3799 |
| hsa-miR-6785-5p | SYNGR1 | 1 | 1 | 1 | 3 | 9145 |
| hsa-miR-3925-5p | G3BP2 | 1 | 1 | 1 | 3 | 9908 |
| hsa-miR-4728-5p | ANGPT4 | 1 | 1 | 1 | 3 | 51378 |
| hsa-miR-6883-5p | CALR | 1 | 1 | 1 | 3 | 811 |
| hsa-miR-486-3p | MKNK2 | 1 | 1 | 1 | 3 | 2872 |
| hsa-miR-6785-5p | FAM83H | 1 | 1 | 1 | 3 | 286077 |
| hsa-miR-6883-5p | PRICKLE1 | 1 | 1 | 1 | 3 | 144165 |
| hsa-miR-149-3p | NEUROD2 | 1 | 1 | 1 | 3 | 4761 |
| hsa-miR-7106-5p | IRAK3 | 1 | 1 | 1 | 3 | 11213 |
| hsa-miR-149-3p | KHSRP | 1 | 1 | 1 | 3 | 8570 |
| hsa-miR-6825-5p | STOML3 | 1 | 1 | 1 | 3 | 161003 |
| hsa-miR-7106-5p | YWHAE | 1 | 1 | 1 | 3 | 7531 |
| hsa-miR-4437 | PLIN5 | 1 | 1 | 1 | 3 | 440503 |
| hsa-miR-4283 | NACC1 | 1 | 1 | 1 | 3 | 112939 |
| hsa-miR-4251 | URM1 | 1 | 1 | 1 | 3 | 81605 |
| hsa-miR-4728-5p | PNMA2 | 1 | 1 | 1 | 3 | 10687 |
| hsa-miR-7106-5p | TOR1AIP2 | 1 | 1 | 1 | 3 | 163590 |
| hsa-miR-6825-5p | NLRC3 | 1 | 1 | 1 | 3 | 197358 |
| hsa-miR-7106-5p | SALL2 | 1 | 1 | 1 | 3 | 6297 |
| hsa-miR-4710 | GABRR2 | 1 | 1 | 1 | 3 | 2570 |
| hsa-miR-186-3p | GPRIN3 | 1 | 1 | 1 | 3 | 285513 |
| hsa-miR-7106-5p | UBE2D4 | 1 | 1 | 1 | 3 | 51619 |
| hsa-miR-149-3p | PHYHIP | 1 | 1 | 1 | 3 | 9796 |
| hsa-miR-6889-5p | ZBTB47 | 1 | 1 | 1 | 3 | 92999 |
| hsa-miR-7854-3p | ARF6 | 1 | 1 | 1 | 3 | 382 |
| hsa-miR-4728-5p | NFAT5 | 1 | 1 | 1 | 3 | 10725 |
| hsa-miR-3123 | FAM120AOS | 1 | 1 | 1 | 3 | 158293 |
| hsa-miR-6883-5p | IKZF3 | 1 | 1 | 1 | 3 | 22806 |
| hsa-miR-7106-5p | PEX11B | 1 | 1 | 1 | 3 | 8799 |
| hsa-miR-6785-5p | DPYSL5 | 1 | 1 | 1 | 3 | 56896 |
| hsa-miR-7977 | ASB6 | 1 | 1 | 1 | 3 | 140459 |
| hsa-miR-6883-5p | PDE7A | 1 | 1 | 1 | 3 | 5150 |
| hsa-miR-18b-3p | ZNF644 | 1 | 1 | 1 | 3 | 84146 |
| hsa-miR-7854-3p | FBLN5 | 1 | 1 | 1 | 3 | 10516 |
| hsa-miR-4437 | MGST3 | 1 | 1 | 1 | 3 | 4259 |
| hsa-miR-4728-5p | C20orf96 | 1 | 1 | 1 | 3 | 140680 |
| hsa-miR-149-3p | MAT1A | 1 | 1 | 1 | 3 | 4143 |
| hsa-miR-6799-5p | DISC1 | 1 | 1 | 1 | 3 | 27185 |
| hsa-miR-6825-5p | TIGD5 | 1 | 1 | 1 | 3 | 84948 |
| hsa-miR-4728-5p | DISC1 | 1 | 1 | 1 | 3 | 27185 |
| hsa-miR-3175 | EIF5AL1 | 1 | 1 | 1 | 3 | 143244 |
| hsa-miR-4728-5p | PARP11 | 1 | 1 | 1 | 3 | 57097 |
| hsa-miR-6883-5p | DUSP7 | 1 | 1 | 1 | 3 | 1849 |
| hsa-miR-6825-5p | HEXA | 1 | 1 | 1 | 3 | 3073 |
| hsa-miR-6785-5p | HAP1 | 1 | 1 | 1 | 3 | 9001 |
| hsa-miR-4728-5p | ATP2A3 | 1 | 1 | 1 | 3 | 489 |
| hsa-miR-7106-5p | EPB41L1 | 1 | 1 | 1 | 3 | 2036 |
| hsa-miR-4728-5p | KLHL21 | 1 | 1 | 1 | 3 | 9903 |
| hsa-miR-7977 | IKZF3 | 1 | 1 | 1 | 3 | 22806 |
| hsa-miR-7977 | ZNF749 | 1 | 1 | 1 | 3 | 388567 |
| hsa-miR-4756-3p | SLC36A1 | 1 | 1 | 1 | 3 | 206358 |
| hsa-miR-7854-3p | MIDN | 1 | 1 | 1 | 3 | 90007 |
| hsa-miR-7977 | PLEKHH1 | 1 | 1 | 1 | 3 | 57475 |
| hsa-miR-3175 | MSN | 1 | 1 | 1 | 3 | 4478 |
| hsa-miR-7106-5p | TRIM66 | 1 | 1 | 1 | 3 | 9866 |
| hsa-miR-6825-5p | GLG1 | 1 | 1 | 1 | 3 | 2734 |
| hsa-miR-4728-5p | LENG8 | 1 | 1 | 1 | 3 | 114823 |
| hsa-miR-7977 | KCNE4 | 1 | 1 | 1 | 3 | 23704 |
| hsa-miR-6134 | KLHDC3 | 1 | 1 | 1 | 3 | 116138 |
| hsa-miR-6785-5p | IER5 | 1 | 1 | 1 | 3 | 51278 |
| hsa-miR-7977 | ACTR2 | 1 | 1 | 1 | 3 | 10097 |
| hsa-miR-939-5p | PGPEP1 | 1 | 1 | 1 | 3 | 54858 |
| hsa-miR-6883-5p | UBLCP1 | 1 | 1 | 1 | 3 | 134510 |
| hsa-miR-149-3p | RAD51B | 1 | 1 | 1 | 3 | 5890 |
| hsa-miR-146b-3p | FAM84B | 1 | 1 | 1 | 3 | 157638 |
| hsa-miR-6134 | TEDDM1 | 1 | 1 | 1 | 3 | 127670 |
| hsa-miR-7106-5p | C11orf54 | 1 | 1 | 1 | 3 | 28970 |
| hsa-miR-7106-5p | RPH3A | 1 | 1 | 1 | 3 | 22895 |
| hsa-miR-4756-3p | LCLAT1 | 1 | 1 | 1 | 3 | 253558 |
| hsa-miR-6799-5p | DHDDS | 1 | 1 | 1 | 3 | 79947 |
| hsa-miR-146b-3p | KLHDC8B | 1 | 1 | 1 | 3 | 200942 |
| hsa-miR-149-3p | RNF157 | 1 | 1 | 1 | 3 | 114804 |
| hsa-miR-6883-5p | RAD51B | 1 | 1 | 1 | 3 | 5890 |
| hsa-miR-4508 | BARHL1 | 1 | 1 | 1 | 3 | 56751 |
| hsa-miR-4303 | PFN2 | 1 | 1 | 1 | 3 | 5217 |
| hsa-miR-7106-5p | PFN1 | 1 | 1 | 1 | 3 | 5216 |
| hsa-miR-3175 | CCL22 | 1 | 1 | 1 | 3 | 6367 |
| hsa-miR-149-3p | SYNGR1 | 1 | 1 | 1 | 3 | 9145 |
| hsa-miR-1296-5p | TBC1D25 | 1 | 1 | 1 | 3 | 4943 |
| hsa-miR-1291 | IQSEC3 | 1 | 1 | 1 | 3 | 440073 |
| hsa-miR-381-5p | RALGAPB | 1 | 1 | 1 | 3 | 57148 |
| hsa-miR-6883-5p | CDK14 | 1 | 1 | 1 | 3 | 5218 |
| hsa-miR-4710 | MTA1 | 1 | 1 | 1 | 3 | 9112 |
| hsa-miR-149-3p | KIAA1328 | 1 | 1 | 1 | 3 | 57536 |
| hsa-miR-4257 | CEP135 | 1 | 1 | 1 | 3 | 9662 |
| hsa-miR-6883-5p | PLEKHH1 | 1 | 1 | 1 | 3 | 57475 |
| hsa-miR-1296-5p | KIF5B | 1 | 1 | 1 | 3 | 3799 |
| hsa-miR-7106-5p | CAPZB | 1 | 1 | 1 | 3 | 832 |
| hsa-miR-149-3p | MKNK2 | 1 | 1 | 1 | 3 | 2872 |
| hsa-miR-4303 | KPNA3 | 1 | 1 | 1 | 3 | 3839 |
| hsa-miR-6785-5p | RPRD2 | 1 | 1 | 1 | 3 | 23248 |
| hsa-miR-6851-5p | BCL2L13 | 1 | 1 | 1 | 3 | 23786 |
| hsa-miR-3175 | HNRNPA3 | 1 | 1 | 1 | 3 | 220988 |
| hsa-miR-4728-5p | MARK2 | 1 | 1 | 1 | 3 | 2011 |
| hsa-miR-6883-5p | PLAGL2 | 1 | 1 | 1 | 3 | 5326 |
| hsa-miR-4728-5p | ZFP36L1 | 1 | 1 | 1 | 3 | 677 |
| hsa-miR-4756-3p | SLAIN2 | 1 | 1 | 1 | 3 | 57606 |
| hsa-miR-6785-5p | CCL22 | 1 | 1 | 1 | 3 | 6367 |
| hsa-miR-6825-5p | YWHAE | 1 | 1 | 1 | 3 | 7531 |
| hsa-miR-6785-5p | CAPZB | 1 | 1 | 1 | 3 | 832 |
| hsa-miR-6785-5p | ZNF491 | 1 | 1 | 1 | 3 | 126069 |
| hsa-miR-4251 | TMTC2 | 1 | 1 | 1 | 3 | 160335 |
| hsa-miR-149-3p | NFIX | 1 | 1 | 1 | 3 | 4784 |
| hsa-miR-6825-5p | PRELP | 1 | 1 | 1 | 3 | 5549 |
| hsa-miR-1343-5p | C9orf62 | 1 | 1 | 1 | 3 | 157927 |
| hsa-miR-6777-5p | CLIP1 | 1 | 1 | 1 | 3 | 6249 |
| hsa-miR-6777-5p | ZBTB47 | 1 | 1 | 1 | 3 | 92999 |
| hsa-miR-4303 | LUZP1 | 1 | 1 | 1 | 3 | 7798 |
| hsa-miR-7854-3p | RXRB | 1 | 1 | 1 | 3 | 6257 |
| hsa-miR-6883-5p | DYRK1B | 1 | 1 | 1 | 3 | 9149 |
| hsa-miR-149-3p | MEX3A | 1 | 1 | 1 | 3 | 92312 |
| hsa-miR-7106-5p | TMEM63C | 1 | 1 | 1 | 3 | 57156 |
| hsa-miR-296-5p | CHCHD4 | 1 | 1 | 1 | 3 | 131474 |
| hsa-miR-6799-5p | MOCS3 | 1 | 1 | 1 | 3 | 27304 |
| hsa-miR-939-5p | C9orf62 | 1 | 1 | 1 | 3 | 157927 |
| hsa-miR-149-3p | TP53 | 1 | 1 | 1 | 3 | 7157 |
| hsa-miR-6851-5p | SAMD4B | 1 | 1 | 1 | 3 | 55095 |
| hsa-miR-149-3p | NAV2 | 1 | 1 | 1 | 3 | 89797 |
| hsa-miR-6883-5p | KCNA5 | 1 | 1 | 1 | 3 | 3741 |
| hsa-miR-486-3p | NSD1 | 1 | 1 | 1 | 3 | 64324 |
| hsa-miR-6825-5p | CANX | 1 | 1 | 1 | 3 | 821 |
| hsa-miR-6851-5p | MARVELD2 | 1 | 1 | 1 | 3 | 153562 |
| hsa-miR-149-3p | KLHL21 | 1 | 1 | 1 | 3 | 9903 |
| hsa-miR-7107-3p | LHFPL2 | 1 | 1 | 1 | 3 | 10184 |
| hsa-miR-6804-5p | RNF126 | 1 | 1 | 1 | 3 | 55658 |
| hsa-miR-1343-5p | HAND2 | 1 | 1 | 1 | 3 | 9464 |
| hsa-miR-186-3p | UBE4B | 1 | 1 | 1 | 3 | 10277 |
| hsa-miR-6895-5p | FOXC1 | 1 | 1 | 1 | 3 | 2296 |
| hsa-miR-4512 | ACER2 | 1 | 1 | 1 | 3 | 340485 |
| hsa-miR-149-3p | C10orf76 | 1 | 1 | 1 | 3 | NA |
| hsa-miR-4251 | ANKRD33B | 1 | 1 | 1 | 3 | 651746 |
| hsa-miR-3175 | ZNF740 | 1 | 1 | 1 | 3 | 283337 |
| hsa-miR-6785-5p | TJP3 | 1 | 1 | 1 | 3 | 27134 |
| hsa-miR-6883-5p | TMEM63C | 1 | 1 | 1 | 3 | 57156 |
| hsa-miR-6883-5p | SCAMP4 | 1 | 1 | 1 | 3 | 113178 |
| hsa-miR-6777-5p | HIC1 | 1 | 1 | 1 | 3 | 3090 |
| hsa-miR-149-3p | ZNF491 | 1 | 1 | 1 | 3 | 126069 |
| hsa-miR-6825-5p | LBX1 | 1 | 1 | 1 | 3 | 10660 |
| hsa-miR-486-3p | COPZ1 | 1 | 1 | 1 | 3 | 22818 |
| hsa-miR-3925-5p | G3BP1 | 1 | 1 | 1 | 3 | 10146 |
| hsa-miR-6825-5p | AMER1 | 1 | 1 | 1 | 3 | 139285 |
| hsa-miR-4251 | ONECUT2 | 1 | 1 | 1 | 3 | 9480 |
| hsa-miR-4728-5p | MLXIP | 1 | 1 | 1 | 3 | 22877 |
| hsa-miR-1182 | ARID1A | 1 | 1 | 1 | 3 | 8289 |
| hsa-miR-4728-5p | NLGN2 | 1 | 1 | 1 | 3 | 57555 |
| hsa-miR-7106-5p | KCNA5 | 1 | 1 | 1 | 3 | 3741 |
| hsa-miR-4251 | OTUD1 | 1 | 1 | 1 | 3 | 220213 |
| hsa-miR-4283 | TPD52L3 | 1 | 1 | 1 | 3 | 89882 |
| hsa-miR-4728-5p | IQSEC3 | 1 | 1 | 1 | 3 | 440073 |
| hsa-miR-3919 | MYC | 1 | 1 | 1 | 3 | 4609 |
| hsa-miR-642b-5p | NPTX1 | 1 | 1 | 1 | 3 | 4884 |
| hsa-miR-186-3p | GK5 | 1 | 1 | 1 | 3 | 256356 |
| hsa-miR-4728-5p | MOB3A | 1 | 1 | 1 | 3 | 126308 |
| hsa-miR-3175 | ADRA2B | 1 | 1 | 1 | 3 | 151 |
| hsa-miR-149-3p | ARHGAP31 | 1 | 1 | 1 | 3 | 57514 |
| hsa-miR-4257 | C20orf27 | 1 | 1 | 1 | 3 | 54976 |
| hsa-miR-7977 | PLEKHG2 | 1 | 1 | 1 | 3 | 64857 |
| hsa-miR-186-3p | SLC43A3 | 1 | 1 | 1 | 3 | 29015 |
| hsa-miR-4640-5p | CDKN2B | 1 | 1 | 1 | 3 | 1030 |
| hsa-miR-7106-5p | SCAMP4 | 1 | 1 | 1 | 3 | 113178 |
| hsa-miR-7977 | SP1 | 1 | 1 | 1 | 3 | 6667 |
| hsa-miR-6825-5p | HIRIP3 | 1 | 1 | 1 | 3 | 8479 |
| hsa-miR-149-3p | SLC25A34 | 1 | 1 | 1 | 3 | 284723 |
| hsa-miR-7106-5p | CBS | 1 | 1 | 1 | 3 | 875 |
| hsa-miR-642b-5p | ZDHHC5 | 1 | 1 | 1 | 3 | 25921 |
| hsa-miR-296-5p | PSMF1 | 1 | 1 | 1 | 3 | 9491 |
| hsa-miR-149-3p | RPRD2 | 1 | 1 | 1 | 3 | 23248 |
| hsa-miR-486-3p | CACNA2D2 | 1 | 1 | 1 | 3 | 9254 |
| hsa-miR-6785-5p | SLC47A1 | 1 | 1 | 1 | 3 | 55244 |
| hsa-miR-6851-5p | KIF13A | 1 | 1 | 1 | 3 | 63971 |
| hsa-miR-6883-5p | NCOR2 | 1 | 1 | 1 | 3 | 9612 |
| hsa-miR-6785-5p | IKZF3 | 1 | 1 | 1 | 3 | 22806 |
| hsa-miR-7106-5p | FKBP5 | 1 | 1 | 1 | 3 | 2289 |
| hsa-miR-4728-5p | RNF111 | 1 | 1 | 1 | 3 | 54778 |
| hsa-miR-6785-5p | ZFP36L1 | 1 | 1 | 1 | 3 | 677 |
| hsa-miR-4728-5p | AGBL5 | 1 | 1 | 1 | 3 | 60509 |
| hsa-miR-7106-5p | TGIF1 | 1 | 1 | 1 | 3 | 7050 |
| hsa-miR-486-3p | RFWD3 | 1 | 1 | 1 | 3 | 55159 |
| hsa-miR-6799-5p | ANKRD40 | 1 | 1 | 1 | 3 | 91369 |
| hsa-miR-4728-5p | PITPNA | 1 | 1 | 1 | 3 | 5306 |
| hsa-miR-3672 | TMPRSS15 | 1 | 1 | 1 | 3 | 5651 |
| hsa-miR-4257 | NEDD4L | 1 | 1 | 1 | 3 | 23327 |
| hsa-miR-6825-5p | STK35 | 1 | 1 | 1 | 3 | 140901 |
| hsa-miR-3672 | RBBP4 | 1 | 1 | 1 | 3 | 5928 |
| hsa-miR-7106-5p | HCFC1 | 1 | 1 | 1 | 3 | 3054 |
| hsa-miR-4728-5p | MGAT5B | 1 | 1 | 1 | 3 | 146664 |
| hsa-miR-6134 | NIPAL3 | 1 | 1 | 1 | 3 | 57185 |
| hsa-miR-3919 | PFDN2 | 1 | 1 | 1 | 3 | 5202 |
| hsa-miR-6883-5p | MKNK2 | 1 | 1 | 1 | 3 | 2872 |
| hsa-miR-6883-5p | ARHGAP31 | 1 | 1 | 1 | 3 | 57514 |
| hsa-miR-6785-5p | TIAL1 | 1 | 1 | 1 | 3 | 7073 |
| hsa-miR-4728-5p | RNF157 | 1 | 1 | 1 | 3 | 114804 |
| hsa-miR-6785-5p | STK35 | 1 | 1 | 1 | 3 | 140901 |
| hsa-miR-6785-5p | ZFHX3 | 1 | 1 | 1 | 3 | 463 |
| hsa-miR-6847-5p | HSP90AA1 | 1 | 1 | 1 | 3 | 3320 |
| hsa-miR-7977 | HUS1 | 1 | 1 | 1 | 3 | 3364 |
| hsa-miR-6134 | ATXN3 | 1 | 1 | 1 | 3 | 4287 |
| hsa-miR-6785-5p | RAB11B | 1 | 1 | 1 | 3 | 9230 |
| hsa-miR-939-5p | NFASC | 1 | 1 | 1 | 3 | 23114 |
| hsa-miR-6785-5p | MOB3A | 1 | 1 | 1 | 3 | 126308 |
| hsa-miR-6895-5p | SAMD4B | 1 | 1 | 1 | 3 | 55095 |
| hsa-miR-7977 | C10orf76 | 1 | 1 | 1 | 3 | NA |
| hsa-miR-7106-5p | ITGA3 | 1 | 1 | 1 | 3 | 3675 |
| hsa-miR-6883-5p | GCDH | 1 | 1 | 1 | 3 | 2639 |
| hsa-miR-149-3p | MYH14 | 1 | 1 | 1 | 3 | 79784 |
| hsa-miR-6761-5p | ATP8B4 | 1 | 1 | 1 | 3 | 79895 |
| hsa-miR-7106-5p | MBD6 | 1 | 1 | 1 | 3 | 114785 |
| hsa-miR-6825-5p | TGIF1 | 1 | 1 | 1 | 3 | 7050 |
| hsa-miR-6883-5p | IGFBP4 | 1 | 1 | 1 | 3 | 3487 |
| hsa-miR-6785-5p | STOML3 | 1 | 1 | 1 | 3 | 161003 |
| hsa-miR-3672 | ZNF443 | 1 | 1 | 1 | 3 | 10224 |
| hsa-miR-6825-5p | PGPEP1 | 1 | 1 | 1 | 3 | 54858 |
| hsa-miR-6825-5p | MGAT5B | 1 | 1 | 1 | 3 | 146664 |
| hsa-miR-6883-5p | MYH9 | 1 | 1 | 1 | 3 | 4627 |
| hsa-miR-6883-5p | PHYHIP | 1 | 1 | 1 | 3 | 9796 |
| hsa-miR-6785-5p | MEX3A | 1 | 1 | 1 | 3 | 92312 |
| hsa-miR-6883-5p | CCL22 | 1 | 1 | 1 | 3 | 6367 |
| hsa-miR-6785-5p | MAT1A | 1 | 1 | 1 | 3 | 4143 |
| hsa-miR-6883-5p | NGFR | 1 | 1 | 1 | 3 | 4804 |
| hsa-miR-6785-5p | SLC29A4 | 1 | 1 | 1 | 3 | 222962 |
| hsa-miR-6825-5p | DYRK1B | 1 | 1 | 1 | 3 | 9149 |
| hsa-miR-149-3p | C20orf96 | 1 | 1 | 1 | 3 | 140680 |
| hsa-miR-6883-5p | DISC1 | 1 | 1 | 1 | 3 | 27185 |
| hsa-miR-4710 | CFL2 | 1 | 1 | 1 | 3 | 1073 |
| hsa-miR-6785-5p | MYH14 | 1 | 1 | 1 | 3 | 79784 |
| hsa-miR-4640-5p | SLC39A1 | 1 | 1 | 1 | 3 | 27173 |
| hsa-miR-7977 | SH3BP2 | 1 | 1 | 1 | 3 | 6452 |
| hsa-miR-6883-5p | PKNOX2 | 1 | 1 | 1 | 3 | 63876 |
| hsa-miR-3919 | PHF13 | 1 | 1 | 1 | 3 | 148479 |
| hsa-miR-4251 | ZNF619 | 1 | 1 | 1 | 3 | 285267 |
| hsa-miR-7977 | ANAPC16 | 1 | 1 | 1 | 3 | 119504 |
| hsa-miR-6883-5p | STK35 | 1 | 1 | 1 | 3 | 140901 |
| hsa-miR-4756-3p | GXYLT1 | 1 | 1 | 1 | 3 | 283464 |
| hsa-miR-6847-5p | CCND2 | 1 | 1 | 1 | 3 | 894 |
| hsa-miR-6883-5p | NAV2 | 1 | 1 | 1 | 3 | 89797 |
| hsa-miR-149-3p | PRICKLE1 | 1 | 1 | 1 | 3 | 144165 |
| hsa-miR-6087 | NCOR2 | 1 | 1 | 1 | 3 | 9612 |
| hsa-miR-6785-5p | ZCCHC8 | 1 | 1 | 1 | 3 | 55596 |
| hsa-miR-149-3p | FEM1A | 1 | 1 | 1 | 3 | 55527 |
| hsa-miR-6883-5p | NFIC | 1 | 1 | 1 | 3 | 4782 |
| hsa-miR-186-3p | INO80D | 1 | 1 | 1 | 3 | 54891 |
| hsa-miR-7977 | MPRIP | 1 | 1 | 1 | 3 | 23164 |
| hsa-miR-6883-5p | STK11 | 1 | 1 | 1 | 3 | 6794 |
| hsa-miR-6825-5p | NACC2 | 1 | 1 | 1 | 3 | 138151 |
| hsa-miR-3672 | CCSER2 | 1 | 1 | 1 | 3 | 54462 |
| hsa-miR-6847-5p | M6PR | 1 | 1 | 1 | 3 | 4074 |
| hsa-miR-6825-5p | USB1 | 1 | 1 | 1 | 3 | 79650 |
| hsa-miR-186-3p | TBL1XR1 | 1 | 1 | 1 | 3 | 79718 |
| hsa-miR-3123 | BAG4 | 1 | 1 | 1 | 3 | 9530 |
| hsa-miR-4728-5p | DESI1 | 1 | 1 | 1 | 3 | 27351 |
| hsa-miR-6883-5p | PPFIA3 | 1 | 1 | 1 | 3 | 8541 |
| hsa-miR-7106-5p | TRAF3IP2 | 1 | 1 | 1 | 3 | 10758 |
| hsa-miR-149-3p | CHRDL1 | 1 | 1 | 1 | 3 | 91851 |
| hsa-miR-6785-5p | COL5A1 | 1 | 1 | 1 | 3 | 1289 |
| hsa-miR-4728-5p | PDE7A | 1 | 1 | 1 | 3 | 5150 |
| hsa-miR-7854-3p | C16orf52 | 1 | 1 | 1 | 3 | NA |
| hsa-miR-7106-5p | GLG1 | 1 | 1 | 1 | 3 | 2734 |
| hsa-miR-149-3p | TSPYL4 | 1 | 1 | 1 | 3 | 23270 |
| hsa-miR-6889-5p | NFIC | 1 | 1 | 1 | 3 | 4782 |
| hsa-miR-4257 | ATXN1L | 1 | 1 | 1 | 3 | 342371 |
| hsa-miR-7106-5p | ATXN7L3 | 1 | 1 | 1 | 3 | 56970 |
| hsa-miR-4283 | SRCIN1 | 1 | 1 | 1 | 3 | 80725 |
| hsa-miR-6851-5p | CIAPIN1 | 1 | 1 | 1 | 3 | 57019 |
| hsa-miR-6883-5p | UBE2S | 1 | 1 | 1 | 3 | 27338 |
| hsa-miR-4437 | UBE2D2 | 1 | 1 | 1 | 3 | 7322 |
| hsa-miR-4251 | SLC43A2 | 1 | 1 | 1 | 3 | 124935 |
| hsa-miR-4728-5p | CHRDL1 | 1 | 1 | 1 | 3 | 91851 |
| hsa-miR-296-5p | RNF44 | 1 | 1 | 1 | 3 | 22838 |
| hsa-miR-6785-5p | PARP11 | 1 | 1 | 1 | 3 | 57097 |
| hsa-miR-6825-5p | MEX3A | 1 | 1 | 1 | 3 | 92312 |
| hsa-miR-149-3p | PKNOX2 | 1 | 1 | 1 | 3 | 63876 |
| hsa-miR-4437 | CC2D1B | 1 | 1 | 1 | 3 | 200014 |
| hsa-miR-7106-5p | TBXA2R | 1 | 1 | 1 | 3 | 6915 |
| hsa-miR-149-3p | CPM | 1 | 1 | 1 | 3 | 1368 |
| hsa-miR-3123 | EIF1AX | 1 | 1 | 1 | 3 | 1964 |
| hsa-miR-7106-5p | PDPR | 1 | 1 | 1 | 3 | 55066 |
| hsa-miR-149-3p | PLAGL2 | 1 | 1 | 1 | 3 | 5326 |
| hsa-miR-4728-5p | ZCCHC8 | 1 | 1 | 1 | 3 | 55596 |
| hsa-miR-149-3p | RASD1 | 1 | 1 | 1 | 3 | 51655 |
| hsa-miR-186-3p | DUSP3 | 1 | 1 | 1 | 3 | 1845 |
| hsa-miR-1343-5p | GNAI2 | 1 | 1 | 1 | 3 | 2771 |
| hsa-miR-6785-5p | SLITRK5 | 1 | 1 | 1 | 3 | 26050 |
| hsa-miR-6851-5p | DNAJC11 | 1 | 1 | 1 | 3 | 55735 |
| hsa-miR-7106-5p | SIX3 | 1 | 1 | 1 | 3 | 6496 |
| hsa-miR-6785-5p | ARL8A | 1 | 1 | 1 | 3 | 127829 |
| hsa-miR-6864-3p | CSTF2T | 1 | 1 | 1 | 3 | 23283 |
| hsa-miR-6777-5p | NFIC | 1 | 1 | 1 | 3 | 4782 |
| hsa-miR-6785-5p | PKNOX2 | 1 | 1 | 1 | 3 | 63876 |
| hsa-miR-149-3p | SREBF1 | 1 | 1 | 1 | 3 | 6720 |
| hsa-miR-6785-5p | SREBF2 | 1 | 1 | 1 | 3 | 6721 |
| hsa-miR-4728-5p | STK35 | 1 | 1 | 1 | 3 | 140901 |
| hsa-miR-6785-5p | RNF111 | 1 | 1 | 1 | 3 | 54778 |
| hsa-miR-6883-5p | ZCCHC3 | 1 | 1 | 1 | 3 | 85364 |
| hsa-miR-6825-5p | RPS6KA2 | 1 | 1 | 1 | 3 | 6196 |
| hsa-miR-3919 | CNBP | 1 | 1 | 1 | 3 | 7555 |
| hsa-miR-149-3p | MAZ | 1 | 1 | 1 | 3 | 4150 |
| hsa-miR-6785-5p | STRN4 | 1 | 1 | 1 | 3 | 29888 |
| hsa-miR-6785-5p | KCNA5 | 1 | 1 | 1 | 3 | 3741 |
| hsa-miR-6889-5p | PLIN1 | 1 | 1 | 1 | 3 | 5346 |
| hsa-miR-186-3p | SPIB | 1 | 1 | 1 | 3 | 6689 |
| hsa-miR-149-3p | TIAL1 | 1 | 1 | 1 | 3 | 7073 |
| hsa-miR-4728-5p | CPM | 1 | 1 | 1 | 3 | 1368 |
| hsa-miR-6825-5p | HIC2 | 1 | 1 | 1 | 3 | 23119 |
| hsa-miR-6851-5p | NACC1 | 1 | 1 | 1 | 3 | 112939 |
| hsa-miR-7977 | GPATCH8 | 1 | 1 | 1 | 3 | 23131 |
| hsa-miR-4728-5p | PSMD11 | 1 | 1 | 1 | 3 | 5717 |
| hsa-miR-7106-5p | NAV2 | 1 | 1 | 1 | 3 | 89797 |
| hsa-miR-3123 | SERPINB3 | 1 | 1 | 1 | 3 | 6317 |
| hsa-miR-6825-5p | ARHGAP31 | 1 | 1 | 1 | 3 | 57514 |
| hsa-miR-6825-5p | IDS | 1 | 1 | 1 | 3 | 3423 |
| hsa-miR-7106-5p | FOSL2 | 1 | 1 | 1 | 3 | 2355 |
| hsa-miR-4710 | TMED10 | 1 | 1 | 1 | 3 | 10972 |
| hsa-miR-7106-5p | NAV1 | 1 | 1 | 1 | 3 | 89796 |
| hsa-miR-486-3p | TXLNA | 1 | 1 | 1 | 3 | 200081 |
| hsa-miR-4303 | KIF1B | 1 | 1 | 1 | 3 | 23095 |
| hsa-miR-149-3p | ITGA3 | 1 | 1 | 1 | 3 | 3675 |
| hsa-miR-6825-5p | CASKIN1 | 1 | 1 | 1 | 3 | 57524 |
| hsa-miR-149-3p | COL5A1 | 1 | 1 | 1 | 3 | 1289 |
| hsa-miR-486-3p | SYNGR1 | 1 | 1 | 1 | 3 | 9145 |
| hsa-miR-4437 | SETD5 | 1 | 1 | 1 | 3 | 55209 |
| hsa-miR-149-3p | SSBP2 | 1 | 1 | 1 | 3 | 23635 |
| hsa-miR-7977 | ZKSCAN3 | 1 | 1 | 1 | 3 | 80317 |
| hsa-miR-6134 | S100A11 | 1 | 1 | 1 | 3 | 6282 |
| hsa-miR-6785-5p | NRGN | 1 | 1 | 1 | 3 | 4900 |
| hsa-miR-6825-5p | RPP25 | 1 | 1 | 1 | 3 | 54913 |
| hsa-miR-6851-5p | RANGAP1 | 1 | 1 | 1 | 3 | 5905 |
| hsa-miR-7106-5p | NACC2 | 1 | 1 | 1 | 3 | 138151 |
| hsa-miR-4728-5p | TJP3 | 1 | 1 | 1 | 3 | 27134 |
| hsa-miR-6775-3p | ZNF148 | 1 | 1 | 1 | 3 | 7707 |
| hsa-miR-149-3p | NFIC | 1 | 1 | 1 | 3 | 4782 |
| hsa-miR-149-3p | STRN4 | 1 | 1 | 1 | 3 | 29888 |
| hsa-miR-4303 | FAM168B | 1 | 1 | 1 | 3 | 130074 |
| hsa-miR-3672 | UBE2D3 | 1 | 1 | 1 | 3 | 7323 |
| hsa-miR-6825-5p | ABL1 | 1 | 1 | 1 | 3 | 25 |
| hsa-miR-7106-5p | SORCS2 | 1 | 1 | 1 | 3 | 57537 |
| hsa-miR-6883-5p | MYH14 | 1 | 1 | 1 | 3 | 79784 |
| hsa-miR-6895-5p | KMT2A | 1 | 1 | 1 | 3 | 4297 |
| hsa-miR-6883-5p | SYNGR1 | 1 | 1 | 1 | 3 | 9145 |
| hsa-miR-6864-3p | AGXT2 | 1 | 1 | 1 | 3 | 64902 |
| hsa-miR-3175 | CALR | 1 | 1 | 1 | 3 | 811 |
| hsa-miR-6785-5p | PHYHIP | 1 | 1 | 1 | 3 | 9796 |
| hsa-miR-6825-5p | HCFC1 | 1 | 1 | 1 | 3 | 3054 |
| hsa-miR-6785-5p | MYH9 | 1 | 1 | 1 | 3 | 4627 |
| hsa-miR-486-3p | DNAJC6 | 1 | 1 | 1 | 3 | 9829 |
| hsa-miR-4710 | NEDD4L | 1 | 1 | 1 | 3 | 23327 |
| hsa-miR-149-3p | RFT1 | 1 | 1 | 1 | 3 | 91869 |
| hsa-miR-4728-5p | RAB11B | 1 | 1 | 1 | 3 | 9230 |
| hsa-miR-4283 | BARHL1 | 1 | 1 | 1 | 3 | 56751 |
| hsa-miR-3175 | SHISA6 | 1 | 1 | 1 | 3 | 388336 |
| hsa-miR-6883-5p | KHSRP | 1 | 1 | 1 | 3 | 8570 |
| hsa-miR-6883-5p | NLGN2 | 1 | 1 | 1 | 3 | 57555 |
| hsa-miR-4728-5p | IER5 | 1 | 1 | 1 | 3 | 51278 |
| hsa-miR-6785-5p | PLAGL2 | 1 | 1 | 1 | 3 | 5326 |
| hsa-miR-4303 | ZNF107 | 1 | 1 | 1 | 3 | 51427 |
| hsa-miR-6825-5p | RAB11FIP4 | 1 | 1 | 1 | 3 | 84440 |
| hsa-miR-4728-5p | IKZF3 | 1 | 1 | 1 | 3 | 22806 |
| hsa-miR-3175 | DYRK1B | 1 | 1 | 1 | 3 | 9149 |
| hsa-miR-149-3p | NAV1 | 1 | 1 | 1 | 3 | 89796 |
| hsa-miR-6785-5p | FEM1A | 1 | 1 | 1 | 3 | 55527 |
| hsa-miR-6825-5p | CSNK1G2 | 1 | 1 | 1 | 3 | 1455 |
| hsa-miR-149-3p | TET3 | 1 | 1 | 1 | 3 | 200424 |
| hsa-miR-4728-5p | PHYHIP | 1 | 1 | 1 | 3 | 9796 |
| hsa-miR-6785-5p | NRBP1 | 1 | 1 | 1 | 3 | 29959 |
| hsa-miR-6851-5p | KRT80 | 1 | 1 | 1 | 3 | 144501 |
| hsa-miR-6825-5p | EVC | 1 | 1 | 1 | 3 | 2121 |
| hsa-miR-4728-5p | TP53 | 1 | 1 | 1 | 3 | 7157 |
| hsa-miR-6825-5p | KLK10 | 1 | 1 | 1 | 3 | 5655 |
| hsa-miR-6777-5p | CASKIN1 | 1 | 1 | 1 | 3 | 57524 |
| hsa-miR-3175 | HIC1 | 1 | 1 | 1 | 3 | 3090 |
| hsa-miR-3123 | SPIRE1 | 1 | 1 | 1 | 3 | 56907 |
| hsa-miR-6804-5p | TLN2 | 1 | 1 | 1 | 3 | 83660 |
| hsa-miR-6785-5p | PLLP | 1 | 1 | 1 | 3 | 51090 |
| hsa-miR-7977 | CPSF2 | 1 | 1 | 1 | 3 | 53981 |
| hsa-miR-7106-5p | COPS7B | 1 | 1 | 1 | 3 | 64708 |
| hsa-miR-4728-5p | MLLT1 | 1 | 1 | 1 | 3 | 4298 |
| hsa-miR-4728-5p | SREBF2 | 1 | 1 | 1 | 3 | 6721 |
| hsa-miR-3123 | GIGYF1 | 1 | 1 | 1 | 3 | 64599 |
| hsa-miR-6785-5p | DISC1 | 1 | 1 | 1 | 3 | 27185 |
| hsa-miR-6799-5p | SAMD4B | 1 | 1 | 1 | 3 | 55095 |
| hsa-miR-6883-5p | TET3 | 1 | 1 | 1 | 3 | 200424 |
| hsa-miR-4728-5p | IGFBP4 | 1 | 1 | 1 | 3 | 3487 |
| hsa-miR-6761-5p | TMCC1 | 1 | 1 | 1 | 3 | 23023 |
| hsa-miR-6825-5p | SHISA6 | 1 | 1 | 1 | 3 | 388336 |
| hsa-miR-4640-5p | ITPRIP | 1 | 1 | 1 | 3 | 85450 |
| hsa-miR-6785-5p | NFIC | 1 | 1 | 1 | 3 | 4782 |
| hsa-miR-6785-5p | MAZ | 1 | 1 | 1 | 3 | 4150 |
| hsa-miR-6785-5p | NCOR2 | 1 | 1 | 1 | 3 | 9612 |
| hsa-miR-149-3p | CNOT6L | 1 | 1 | 1 | 3 | 246175 |
| hsa-miR-4251 | NCOA3 | 1 | 1 | 1 | 3 | 8202 |
| hsa-miR-6883-5p | PRR12 | 1 | 1 | 1 | 3 | 57479 |
| hsa-miR-6851-5p | TOR1AIP2 | 1 | 1 | 1 | 3 | 163590 |
| hsa-miR-4283 | GRID1 | 1 | 1 | 1 | 3 | 2894 |
| hsa-miR-4283 | GIGYF1 | 1 | 1 | 1 | 3 | 64599 |
| hsa-miR-7854-3p | AKT3 | 1 | 1 | 1 | 3 | 10000 |
| hsa-miR-7106-5p | RAD51B | 1 | 1 | 1 | 3 | 5890 |
| hsa-miR-4728-5p | NFIX | 1 | 1 | 1 | 3 | 4784 |
| hsa-miR-6883-5p | TBXA2R | 1 | 1 | 1 | 3 | 6915 |
| hsa-miR-589-5p | ATP13A3 | 1 | 1 | 1 | 3 | 79572 |
| hsa-miR-6785-5p | ANKRD45 | 1 | 1 | 1 | 3 | 339416 |
| hsa-miR-6851-5p | EFNB1 | 1 | 1 | 1 | 3 | 1947 |
| hsa-miR-6785-5p | CPM | 1 | 1 | 1 | 3 | 1368 |
| hsa-miR-7107-3p | GOLGA5 | 1 | 1 | 1 | 3 | 9950 |
| hsa-miR-6883-5p | RAB11B | 1 | 1 | 1 | 3 | 9230 |
| hsa-miR-4508 | LYPLA2 | 1 | 1 | 1 | 3 | 11313 |
| hsa-miR-7106-5p | PFKFB3 | 1 | 1 | 1 | 3 | 5209 |
| hsa-miR-4728-5p | SLC29A1 | 1 | 1 | 1 | 3 | 2030 |
| hsa-miR-186-3p | POGZ | 1 | 1 | 1 | 3 | 23126 |
| hsa-miR-6825-5p | CLSTN1 | 1 | 1 | 1 | 3 | 22883 |
| hsa-miR-6785-5p | CALR | 1 | 1 | 1 | 3 | 811 |
| hsa-miR-4728-5p | NRGN | 1 | 1 | 1 | 3 | 4900 |
| hsa-miR-149-3p | MARK2 | 1 | 1 | 1 | 3 | 2011 |
| hsa-miR-7854-3p | ARID1A | 1 | 1 | 1 | 3 | 8289 |
| hsa-miR-486-3p | DLGAP4 | 1 | 1 | 1 | 3 | 22839 |
| hsa-miR-7977 | PLXNA2 | 1 | 1 | 1 | 3 | 5362 |
| hsa-miR-7160-3p | FAM83F | 1 | 1 | 1 | 3 | 113828 |
| hsa-miR-6825-5p | NXN | 1 | 1 | 1 | 3 | 64359 |
| hsa-miR-486-3p | ATXN7L3 | 1 | 1 | 1 | 3 | 56970 |
| hsa-miR-6825-5p | COPS7B | 1 | 1 | 1 | 3 | 64708 |
| hsa-miR-4728-5p | NAV1 | 1 | 1 | 1 | 3 | 89796 |
| hsa-miR-4251 | CTBP1 | 1 | 1 | 1 | 3 | 1487 |
| hsa-miR-186-3p | FBXL17 | 1 | 1 | 1 | 3 | 64839 |
| hsa-miR-7977 | TRAPPC2 | 1 | 1 | 1 | 3 | 6399 |
| hsa-miR-4251 | HLA-DOA | 1 | 1 | 1 | 3 | 3111 |
| hsa-miR-6785-5p | SSBP2 | 1 | 1 | 1 | 3 | 23635 |
| hsa-miR-7106-5p | PCYT1A | 1 | 1 | 1 | 3 | 5130 |
| hsa-miR-4251 | MIER1 | 1 | 1 | 1 | 3 | 57708 |
| hsa-miR-6785-5p | TMTC1 | 1 | 1 | 1 | 3 | 83857 |
| hsa-miR-149-3p | NGFR | 1 | 1 | 1 | 3 | 4804 |
| hsa-miR-4728-5p | SAMD14 | 1 | 1 | 1 | 3 | 201191 |
| hsa-miR-486-3p | MDM4 | 1 | 1 | 1 | 3 | 4194 |
| hsa-miR-7977 | TMEM120B | 1 | 1 | 1 | 3 | 144404 |
| hsa-miR-7106-5p | PRELP | 1 | 1 | 1 | 3 | 5549 |
| hsa-miR-6825-5p | NRBP1 | 1 | 1 | 1 | 3 | 29959 |
| hsa-miR-7854-3p | ARIH2 | 1 | 1 | 1 | 3 | 10425 |
| hsa-miR-4728-5p | TIAL1 | 1 | 1 | 1 | 3 | 7073 |
| hsa-miR-6847-5p | RNF185 | 1 | 1 | 1 | 3 | 91445 |
| hsa-miR-6883-5p | TIAL1 | 1 | 1 | 1 | 3 | 7073 |
| hsa-miR-4728-5p | PPFIA3 | 1 | 1 | 1 | 3 | 8541 |
| hsa-miR-6785-5p | MLLT1 | 1 | 1 | 1 | 3 | 4298 |
| hsa-miR-6799-5p | DNAJC24 | 1 | 1 | 1 | 3 | 120526 |
| hsa-miR-4710 | LCE1A | 1 | 1 | 1 | 3 | 353131 |
| hsa-miR-7106-5p | NRBP1 | 1 | 1 | 1 | 3 | 29959 |
| hsa-miR-6847-5p | LUC7L2 | 1 | 1 | 1 | 3 | 51631 |
| hsa-miR-4728-5p | BTG2 | 1 | 1 | 1 | 3 | 7832 |
| hsa-miR-149-3p | ZNF503 | 1 | 1 | 1 | 3 | 84858 |
| hsa-miR-4251 | CHAC1 | 1 | 1 | 1 | 3 | 79094 |
| hsa-miR-6851-5p | DDN | 1 | 1 | 1 | 3 | 23109 |
| hsa-miR-296-5p | SYT2 | 1 | 1 | 1 | 3 | 127833 |
| hsa-miR-6825-5p | ZNF740 | 1 | 1 | 1 | 3 | 283337 |
| hsa-miR-149-3p | PITPNA | 1 | 1 | 1 | 3 | 5306 |
| hsa-miR-7854-3p | OVOL1 | 1 | 1 | 1 | 3 | 5017 |
| hsa-miR-7106-5p | GLYR1 | 1 | 1 | 1 | 3 | 84656 |
| hsa-miR-186-3p | FOXO1 | 1 | 1 | 1 | 3 | 2308 |
| hsa-miR-4303 | USP46 | 1 | 1 | 1 | 3 | 64854 |
| hsa-miR-6883-5p | BTG2 | 1 | 1 | 1 | 3 | 7832 |
| hsa-miR-3925-5p | MLEC | 1 | 1 | 1 | 3 | 9761 |
| hsa-miR-4251 | PRKAB2 | 1 | 1 | 1 | 3 | 5565 |
| hsa-miR-486-3p | RALY | 1 | 1 | 1 | 3 | 22913 |
| hsa-miR-589-5p | SHCBP1 | 1 | 1 | 1 | 3 | 79801 |
| hsa-miR-149-3p | AGBL5 | 1 | 1 | 1 | 3 | 60509 |
| hsa-miR-146b-3p | KCTD21 | 1 | 1 | 1 | 3 | 283219 |
| hsa-miR-6785-5p | ZNF503 | 1 | 1 | 1 | 3 | 84858 |
| hsa-miR-4728-5p | COL5A1 | 1 | 1 | 1 | 3 | 1289 |
| hsa-miR-7106-5p | DNAJC8 | 1 | 1 | 1 | 3 | 22826 |
| hsa-miR-149-3p | HOXC4 | 1 | 1 | 1 | 3 | 3221 |
| hsa-miR-642b-5p | SH3BP5L | 1 | 1 | 1 | 3 | 80851 |
| hsa-miR-149-3p | RAB7A | 1 | 1 | 1 | 3 | 7879 |
| hsa-miR-3175 | BARHL1 | 1 | 1 | 1 | 3 | 56751 |
| hsa-miR-6883-5p | GPR173 | 1 | 1 | 1 | 3 | 54328 |
| hsa-miR-186-3p | VPS53 | 1 | 1 | 1 | 3 | 55275 |
| hsa-miR-6825-5p | PACSIN1 | 1 | 1 | 1 | 3 | 29993 |
| hsa-miR-4728-5p | CTDSP2 | 1 | 1 | 1 | 3 | 10106 |
| hsa-miR-149-3p | ANKRD40 | 1 | 1 | 1 | 3 | 91369 |
| hsa-miR-4756-3p | PDZD2 | 1 | 1 | 1 | 3 | 23037 |
| hsa-miR-6883-5p | ASXL1 | 1 | 1 | 1 | 3 | 171023 |
| hsa-miR-6753-3p | GOLGA5 | 1 | 1 | 1 | 3 | 9950 |
| hsa-miR-6825-5p | XPO6 | 1 | 1 | 1 | 3 | 23214 |
| hsa-miR-3123 | TMEM47 | 1 | 1 | 1 | 3 | 83604 |
| hsa-miR-296-5p | TOM1 | 1 | 1 | 1 | 3 | 10043 |
| hsa-miR-149-3p | RNF111 | 1 | 1 | 1 | 3 | 54778 |
| hsa-miR-6134 | RAB5B | 1 | 1 | 1 | 3 | 5869 |
| hsa-miR-6864-3p | TMPRSS15 | 1 | 1 | 1 | 3 | 5651 |
| hsa-miR-6825-5p | GPHA2 | 1 | 1 | 1 | 3 | 170589 |
| hsa-miR-6134 | PLAGL2 | 1 | 1 | 1 | 3 | 5326 |
| hsa-miR-486-3p | SRCIN1 | 1 | 1 | 1 | 3 | 80725 |
| hsa-miR-7977 | ZNF582 | 1 | 1 | 1 | 3 | 147948 |
| hsa-miR-6825-5p | CELSR2 | 1 | 1 | 1 | 3 | 1952 |
| hsa-miR-149-3p | TMTC1 | 1 | 1 | 1 | 3 | 83857 |
| hsa-miR-7106-5p | TRIM67 | 1 | 1 | 1 | 3 | 440730 |
| hsa-miR-149-3p | FAM83H | 1 | 1 | 1 | 3 | 286077 |
| hsa-miR-149-3p | TMEM63C | 1 | 1 | 1 | 3 | 57156 |
| hsa-miR-6785-5p | MNT | 1 | 1 | 1 | 3 | 4335 |
| hsa-miR-6883-5p | ZNF503 | 1 | 1 | 1 | 3 | 84858 |
| hsa-miR-6883-5p | NFIX | 1 | 1 | 1 | 3 | 4784 |
| hsa-miR-4728-5p | NFIC | 1 | 1 | 1 | 3 | 4782 |
| hsa-miR-149-3p | NCOR2 | 1 | 1 | 1 | 3 | 9612 |
| hsa-miR-3123 | ORC4 | 1 | 1 | 1 | 3 | 5000 |
| hsa-miR-4728-5p | RAD51B | 1 | 1 | 1 | 3 | 5890 |
| hsa-miR-7106-5p | CIAPIN1 | 1 | 1 | 1 | 3 | 57019 |
| hsa-miR-3919 | MAP3K9 | 1 | 1 | 1 | 3 | 4293 |
| hsa-miR-4257 | ACER2 | 1 | 1 | 1 | 3 | 340485 |
| hsa-miR-6825-5p | ANK1 | 1 | 1 | 1 | 3 | 286 |
| hsa-miR-6825-5p | PRR12 | 1 | 1 | 1 | 3 | 57479 |
| hsa-miR-149-3p | ZFHX3 | 1 | 1 | 1 | 3 | 463 |
| hsa-miR-3925-5p | NUAK2 | 1 | 1 | 1 | 3 | 81788 |
| hsa-miR-6883-5p | CNOT6L | 1 | 1 | 1 | 3 | 246175 |
| hsa-miR-6883-5p | SLC29A1 | 1 | 1 | 1 | 3 | 2030 |
| hsa-miR-3175 | UBE4B | 1 | 1 | 1 | 3 | 10277 |
| hsa-miR-4508 | RGS6 | 1 | 1 | 1 | 3 | 9628 |
| hsa-miR-186-3p | CPT1A | 1 | 1 | 1 | 3 | 1374 |
| hsa-miR-6785-5p | STK11 | 1 | 1 | 1 | 3 | 6794 |
| hsa-miR-149-3p | STK11 | 1 | 1 | 1 | 3 | 6794 |
| hsa-miR-4728-5p | MYH14 | 1 | 1 | 1 | 3 | 79784 |
| hsa-miR-6889-5p | CLIP1 | 1 | 1 | 1 | 3 | 6249 |
| hsa-miR-6883-5p | CACNB1 | 1 | 1 | 1 | 3 | 782 |
| hsa-miR-149-3p | BTG2 | 1 | 1 | 1 | 3 | 7832 |
| hsa-miR-6825-5p | CST9 | 1 | 1 | 1 | 3 | 128822 |
| hsa-miR-6785-5p | RFT1 | 1 | 1 | 1 | 3 | 91869 |
| hsa-miR-4728-5p | RFT1 | 1 | 1 | 1 | 3 | 91869 |
| hsa-miR-6825-5p | TULP1 | 1 | 1 | 1 | 3 | 7287 |
| hsa-miR-6825-5p | FAM83H | 1 | 1 | 1 | 3 | 286077 |
| hsa-miR-3123 | PAPOLG | 1 | 1 | 1 | 3 | 64895 |
| hsa-miR-6785-5p | PIGR | 1 | 1 | 1 | 3 | 5284 |
| hsa-miR-6883-5p | FEM1A | 1 | 1 | 1 | 3 | 55527 |
| hsa-miR-6883-5p | MSI1 | 1 | 1 | 1 | 3 | 4440 |
| hsa-miR-149-3p | MYH9 | 1 | 1 | 1 | 3 | 4627 |
| hsa-miR-6895-5p | AHCYL2 | 1 | 1 | 1 | 3 | 23382 |
| hsa-miR-3123 | KDM5A | 1 | 1 | 1 | 3 | 5927 |
| hsa-miR-6134 | CNNM3 | 1 | 1 | 1 | 3 | 26505 |
| hsa-miR-4728-5p | ASXL1 | 1 | 1 | 1 | 3 | 171023 |
| hsa-miR-6825-5p | CBS | 1 | 1 | 1 | 3 | 875 |
| hsa-miR-6883-5p | DPYSL5 | 1 | 1 | 1 | 3 | 56896 |
| hsa-miR-4283 | SLC35E2 | 1 | 1 | 1 | 3 | NA |
| hsa-miR-149-3p | PNMA2 | 1 | 1 | 1 | 3 | 10687 |
| hsa-miR-6804-5p | IGF1R | 1 | 1 | 1 | 3 | 3480 |
| hsa-miR-6785-5p | ZCCHC3 | 1 | 1 | 1 | 3 | 85364 |
| hsa-miR-4728-5p | FAM83H | 1 | 1 | 1 | 3 | 286077 |
| hsa-miR-6883-5p | MAT1A | 1 | 1 | 1 | 3 | 4143 |
| hsa-miR-4728-5p | PLEKHH1 | 1 | 1 | 1 | 3 | 57475 |
| hsa-miR-149-3p | CDK14 | 1 | 1 | 1 | 3 | 5218 |
| hsa-miR-7977 | CLEC7A | 1 | 1 | 1 | 3 | 64581 |
| hsa-miR-6775-3p | VWA5A | 1 | 1 | 1 | 3 | 4013 |
| hsa-miR-149-3p | CAPZB | 1 | 1 | 1 | 3 | 832 |
| hsa-miR-6895-5p | SFPQ | 1 | 1 | 1 | 3 | 6421 |
| hsa-miR-149-3p | TJP3 | 1 | 1 | 1 | 3 | 27134 |
| hsa-miR-4756-3p | CLSPN | 1 | 1 | 1 | 3 | 63967 |
| hsa-miR-4728-5p | TET3 | 1 | 1 | 1 | 3 | 200424 |
| hsa-miR-6883-5p | ARL8A | 1 | 1 | 1 | 3 | 127829 |
| hsa-miR-1291 | VWA5A | 1 | 1 | 1 | 3 | 4013 |
| hsa-miR-3123 | PNISR | 1 | 1 | 1 | 3 | 25957 |
| hsa-miR-939-5p | MAPRE1 | 1 | 1 | 1 | 3 | 22919 |
| hsa-miR-6785-5p | PLEKHH1 | 1 | 1 | 1 | 3 | 57475 |
| hsa-miR-6825-5p | SALL2 | 1 | 1 | 1 | 3 | 6297 |
| hsa-miR-4283 | ASB6 | 1 | 1 | 1 | 3 | 140459 |
| hsa-miR-6785-5p | GPR173 | 1 | 1 | 1 | 3 | 54328 |
| hsa-miR-6753-3p | KLHL3 | 1 | 1 | 1 | 3 | 26249 |
| hsa-miR-6785-5p | MLXIP | 1 | 1 | 1 | 3 | 22877 |
| hsa-miR-5090 | HNRNPC | 1 | 1 | 1 | 3 | 3183 |
| hsa-miR-7106-5p | HECTD3 | 1 | 1 | 1 | 3 | 79654 |
| hsa-miR-3919 | CREBL2 | 1 | 1 | 1 | 3 | 1389 |
| hsa-miR-4792 | COX6A1 | 1 | 1 | 1 | 3 | 1337 |
| hsa-miR-7106-5p | SLC29A2 | 1 | 1 | 1 | 3 | 3177 |
| hsa-miR-7160-3p | IGF2BP1 | 1 | 1 | 1 | 3 | 10642 |
| hsa-miR-6851-3p | GTPBP2 | 1 | 1 | 1 | 3 | 54676 |
| hsa-miR-6851-5p | TMEM81 | 1 | 1 | 1 | 3 | 388730 |
| hsa-miR-4728-5p | TBXA2R | 1 | 1 | 1 | 3 | 6915 |
| hsa-miR-6847-5p | KPNA2 | 1 | 1 | 1 | 3 | 3838 |
| hsa-miR-4640-5p | ORAI2 | 1 | 1 | 1 | 3 | 80228 |
| hsa-miR-6785-5p | FBXO45 | 1 | 1 | 1 | 3 | 200933 |
| hsa-miR-4437 | STEAP3 | 1 | 1 | 1 | 3 | 55240 |
| hsa-miR-7854-3p | ABHD2 | 1 | 1 | 1 | 3 | 11057 |
| hsa-miR-149-3p | MGAT5B | 1 | 1 | 1 | 3 | 146664 |
| hsa-miR-4728-5p | IFFO2 | 1 | 1 | 1 | 3 | 126917 |
| hsa-miR-3123 | TM9SF4 | 1 | 1 | 1 | 3 | 9777 |
| hsa-miR-149-3p | FGFR1 | 1 | 1 | 1 | 3 | 2260 |
| hsa-miR-6851-5p | DMWD | 1 | 1 | 1 | 3 | 1762 |
| hsa-miR-3925-5p | FKBP1A | 1 | 1 | 1 | 3 | 2280 |
| hsa-miR-7106-5p | URM1 | 1 | 1 | 1 | 3 | 81605 |
| hsa-miR-149-3p | MLLT1 | 1 | 1 | 1 | 3 | 4298 |
| hsa-miR-6883-5p | NFAT5 | 1 | 1 | 1 | 3 | 10725 |
| hsa-miR-6785-5p | WNT7B | 1 | 1 | 1 | 3 | 7477 |
| hsa-miR-6785-5p | HOXC4 | 1 | 1 | 1 | 3 | 3221 |
| hsa-miR-4283 | TEX261 | 1 | 1 | 1 | 3 | 113419 |
| hsa-miR-6785-5p | C20orf96 | 1 | 1 | 1 | 3 | 140680 |
| hsa-miR-4512 | TSTD2 | 1 | 1 | 1 | 3 | 158427 |
| hsa-miR-6883-5p | MOB3A | 1 | 1 | 1 | 3 | 126308 |
| hsa-miR-6825-5p | ANKRD52 | 1 | 1 | 1 | 3 | 283373 |
| hsa-miR-3919 | ARHGAP1 | 1 | 1 | 1 | 3 | 392 |
| hsa-miR-4303 | TP53INP2 | 1 | 1 | 1 | 3 | 58476 |
| hsa-miR-6785-5p | CACNB1 | 1 | 1 | 1 | 3 | 782 |
| hsa-miR-6825-5p | MRPL18 | 1 | 1 | 1 | 3 | 29074 |
| hsa-miR-3123 | CYP51A1 | 1 | 1 | 1 | 3 | 1595 |
| hsa-miR-381-5p | CTPS1 | 1 | 1 | 1 | 3 | 1503 |
| hsa-miR-186-3p | UBN2 | 1 | 1 | 1 | 3 | 254048 |
| hsa-miR-149-3p | CASZ1 | 1 | 1 | 1 | 3 | 54897 |
| hsa-miR-4728-5p | WNT7B | 1 | 1 | 1 | 3 | 7477 |
| hsa-miR-3175 | LPCAT3 | 1 | 1 | 1 | 3 | 10162 |
| hsa-miR-186-3p | SOCS7 | 1 | 1 | 1 | 3 | 30837 |
| hsa-miR-6825-5p | ZBTB7B | 1 | 1 | 1 | 3 | 51043 |
| hsa-miR-6825-5p | NLRP2 | 1 | 1 | 1 | 3 | 55655 |
| hsa-miR-186-3p | KIAA0930 | 1 | 1 | 1 | 3 | 23313 |
| hsa-miR-6087 | TLN2 | 1 | 1 | 1 | 3 | 83660 |
| hsa-miR-7854-3p | SP2 | 1 | 1 | 1 | 3 | 6668 |
| hsa-miR-7977 | PPP1R16B | 1 | 1 | 1 | 3 | 26051 |
| hsa-miR-4283 | ZNF689 | 1 | 1 | 1 | 3 | 115509 |
| hsa-miR-486-3p | MRPL34 | 1 | 1 | 1 | 3 | 64981 |
| hsa-miR-4257 | PIM3 | 1 | 1 | 1 | 3 | 415116 |
| hsa-miR-486-3p | SHMT1 | 1 | 1 | 1 | 3 | 6470 |
| hsa-miR-3123 | SERPINB4 | 1 | 1 | 1 | 3 | 6318 |
| hsa-miR-6825-5p | CRY2 | 1 | 1 | 1 | 3 | 1408 |
| hsa-miR-6785-5p | ANKRD40 | 1 | 1 | 1 | 3 | 91369 |
| hsa-miR-4257 | PDP2 | 1 | 1 | 1 | 3 | 57546 |
| hsa-miR-6134 | SLC5A6 | 1 | 1 | 1 | 3 | 8884 |
| hsa-miR-4728-5p | GPR173 | 1 | 1 | 1 | 3 | 54328 |
| hsa-miR-6785-5p | MGAT5B | 1 | 1 | 1 | 3 | 146664 |
| hsa-miR-6825-5p | SAP18 | 1 | 1 | 1 | 3 | 10284 |
| hsa-miR-6785-5p | PRR12 | 1 | 1 | 1 | 3 | 57479 |
| hsa-miR-7106-5p | MAT2A | 1 | 1 | 1 | 3 | 4144 |
| hsa-miR-6889-5p | HIC1 | 1 | 1 | 1 | 3 | 3090 |
| hsa-miR-149-3p | PARP11 | 1 | 1 | 1 | 3 | 57097 |
| hsa-miR-6889-5p | CASKIN1 | 1 | 1 | 1 | 3 | 57524 |
| hsa-miR-6825-5p | MKNK2 | 1 | 1 | 1 | 3 | 2872 |
| hsa-miR-6883-5p | ANKRD40 | 1 | 1 | 1 | 3 | 91369 |
| hsa-miR-7977 | ATP9A | 1 | 1 | 1 | 3 | 10079 |
| hsa-miR-6785-5p | CNOT6L | 1 | 1 | 1 | 3 | 246175 |
| hsa-miR-4303 | CYB561 | 1 | 1 | 1 | 3 | 1534 |
| hsa-miR-7106-5p | NXN | 1 | 1 | 1 | 3 | 64359 |
| hsa-miR-3919 | TMEM47 | 1 | 1 | 1 | 3 | 83604 |
| hsa-miR-4728-5p | DYRK1B | 1 | 1 | 1 | 3 | 9149 |
| hsa-miR-4512 | DDX3X | 1 | 1 | 1 | 3 | 1654 |
| hsa-miR-149-3p | ZCCHC8 | 1 | 1 | 1 | 3 | 55596 |
| hsa-miR-3175 | PSME3 | 1 | 1 | 1 | 3 | 10197 |
| hsa-miR-1343-5p | SLC9A3R2 | 1 | 1 | 1 | 3 | 9351 |
| hsa-miR-3123 | ZWILCH | 1 | 1 | 1 | 3 | 55055 |
| hsa-miR-6851-5p | SLC19A3 | 1 | 1 | 1 | 3 | 80704 |
| hsa-miR-7106-5p | PLAGL2 | 1 | 1 | 1 | 3 | 5326 |
| hsa-miR-7106-5p | PARP11 | 1 | 1 | 1 | 3 | 57097 |
| hsa-miR-3925-5p | NPAT | 1 | 1 | 1 | 3 | 4863 |
| hsa-miR-4283 | PUM1 | 1 | 1 | 1 | 3 | 9698 |
| hsa-miR-296-5p | ZDHHC8 | 1 | 1 | 1 | 3 | 29801 |
| hsa-miR-6864-3p | KLLN | 1 | 1 | 1 | 3 | 100144748 |
| hsa-miR-296-5p | KCTD12 | 1 | 1 | 1 | 3 | 115207 |
| hsa-miR-1296-5p | ANKRD24 | 1 | 1 | 1 | 3 | 170961 |
| hsa-miR-149-3p | FN3K | 1 | 1 | 1 | 3 | 64122 |
| hsa-miR-3175 | RAPGEF1 | 1 | 1 | 1 | 3 | 2889 |
| hsa-miR-6895-5p | FLOT2 | 1 | 1 | 1 | 3 | 2319 |
| hsa-miR-149-3p | WNT7B | 1 | 1 | 1 | 3 | 7477 |
| hsa-miR-7106-5p | TEX261 | 1 | 1 | 1 | 3 | 113419 |
| hsa-miR-6883-5p | MEX3A | 1 | 1 | 1 | 3 | 92312 |
| hsa-miR-3925-5p | IGFBP5 | 1 | 1 | 1 | 3 | 3488 |
| hsa-miR-6825-5p | SCAMP4 | 1 | 1 | 1 | 3 | 113178 |
| hsa-miR-6785-5p | CBS | 1 | 1 | 1 | 3 | 875 |
| hsa-miR-6785-5p | ITGA3 | 1 | 1 | 1 | 3 | 3675 |
| hsa-miR-6785-5p | DYRK1B | 1 | 1 | 1 | 3 | 9149 |
| hsa-miR-4257 | CNNM4 | 1 | 1 | 1 | 3 | 26504 |
| hsa-miR-6134 | HAX1 | 1 | 1 | 1 | 3 | 10456 |
| hsa-miR-6851-5p | TMED10 | 1 | 1 | 1 | 3 | 10972 |
| hsa-miR-4728-5p | PLEKHG2 | 1 | 1 | 1 | 3 | 64857 |
| hsa-miR-6883-5p | ASB6 | 1 | 1 | 1 | 3 | 140459 |
| hsa-miR-7854-3p | HOXB6 | 1 | 1 | 1 | 3 | 3216 |
| hsa-miR-7160-3p | SLC7A5 | 1 | 1 | 1 | 3 | 8140 |
| hsa-miR-6825-5p | ARRB1 | 1 | 1 | 1 | 3 | 408 |
| hsa-miR-4728-5p | ZNF503 | 1 | 1 | 1 | 3 | 84858 |
| hsa-miR-4728-5p | ARL8A | 1 | 1 | 1 | 3 | 127829 |
| hsa-miR-4728-5p | IQSEC2 | 1 | 1 | 1 | 3 | 23096 |
| hsa-miR-4728-5p | SCAMP4 | 1 | 1 | 1 | 3 | 113178 |
| hsa-miR-4710 | ATG9A | 1 | 1 | 1 | 3 | 79065 |
| hsa-miR-6825-5p | REG4 | 1 | 1 | 1 | 3 | 83998 |
| hsa-miR-6785-5p | NEUROD2 | 1 | 1 | 1 | 3 | 4761 |
| hsa-miR-6883-5p | RFT1 | 1 | 1 | 1 | 3 | 91869 |
| hsa-miR-6825-5p | THY1 | 1 | 1 | 1 | 3 | 7070 |
| hsa-miR-4640-5p | LMBR1L | 1 | 1 | 1 | 3 | 55716 |
| hsa-miR-3672 | KLLN | 1 | 1 | 1 | 3 | 100144748 |
| hsa-miR-4728-5p | UBE2S | 1 | 1 | 1 | 3 | 27338 |
| hsa-miR-6785-5p | PITPNA | 1 | 1 | 1 | 3 | 5306 |
| hsa-miR-1291 | ZNF148 | 1 | 1 | 1 | 3 | 7707 |
| hsa-miR-3175 | FOXC1 | 1 | 1 | 1 | 3 | 2296 |
| hsa-miR-6847-5p | PPP2R5E | 1 | 1 | 1 | 3 | 5529 |
| hsa-miR-4728-5p | HAP1 | 1 | 1 | 1 | 3 | 9001 |
| hsa-miR-3123 | ID4 | 1 | 1 | 1 | 3 | 3400 |
| hsa-miR-4728-5p | RASD1 | 1 | 1 | 1 | 3 | 51655 |
| hsa-miR-149-3p | PDE7A | 1 | 1 | 1 | 3 | 5150 |
| hsa-miR-6851-5p | WNT7B | 1 | 1 | 1 | 3 | 7477 |
| hsa-miR-6785-5p | CDK14 | 1 | 1 | 1 | 3 | 5218 |
| hsa-miR-7106-5p | SLC2A4 | 1 | 1 | 1 | 3 | 6517 |
| hsa-miR-6799-5p | ZCCHC24 | 1 | 1 | 1 | 3 | 219654 |
| hsa-miR-4437 | AMOTL2 | 1 | 1 | 1 | 3 | 51421 |
| hsa-miR-7106-5p | IDS | 1 | 1 | 1 | 3 | 3423 |
| hsa-miR-7977 | SNTN | 1 | 1 | 1 | 3 | 132203 |
| hsa-miR-4728-5p | CCL22 | 1 | 1 | 1 | 3 | 6367 |
| hsa-miR-6825-5p | CCL22 | 1 | 1 | 1 | 3 | 6367 |
| hsa-miR-6785-5p | KRT80 | 1 | 1 | 1 | 3 | 144501 |
| hsa-miR-6785-5p | NFAT5 | 1 | 1 | 1 | 3 | 10725 |
| hsa-miR-6785-5p | SCAMP4 | 1 | 1 | 1 | 3 | 113178 |
| hsa-miR-6825-5p | ADD2 | 1 | 1 | 1 | 3 | 119 |
| hsa-miR-6825-5p | ICK | 1 | 1 | 1 | 3 | 22858 |
| hsa-miR-7977 | MINOS1 | 1 | 1 | 1 | 3 | 440574 |
| hsa-miR-4728-5p | TMTC1 | 1 | 1 | 1 | 3 | 83857 |
| hsa-miR-6895-5p | ANKRD24 | 1 | 1 | 1 | 3 | 170961 |
