# Supplementary material for: Up-Regulation of hsa_circ_0000517 Predicts Adverse Prognosis of Hepatocellular Carcinoma
Source: Front Oncol. 2019 Oct 22;9:1105. doi: 10.3389/fonc.2019.01105 (PMC6842961; doi:10.3389/fonc.2019.01105)
Supplement: Supplementary file 7 [file Table_7.docx]

**Table S7. The GO terms of 742 potential genes.**

| ID | Description | GeneRatio | BgRatio | pvalue | p.adjust | qvalue | geneID | Count |
| --- | --- | --- | --- | --- | --- | --- | --- | --- |
| GO:0042826 | histone deacetylase binding | 18/693 | 109/17548 | 2.52E-07 | 0.000207715 | 0.000193408 | TP53/SFPQ/RAC1/HSPA1B/NCOR2/HDAC5/DHX36/NACC2/YWHAE/HIC1/SP1/RBBP4/HSP90AA1/SIX3/MIER1/KCTD21/KPNA2/SP2 | 18 |
| GO:0000982 | transcription factor activity, RNA polymerase II proximal promoter sequence-specific DNA binding | 34/693 | 415/17548 | 5.03E-05 | 0.014360726 | 0.01337163 | PLAGL2/TP53/NFAT5/BTG2/HAND2/ZFHX3/SREBF2/GCFC2/POU2F3/HMGA2/USF2/SSBP2/ATF6/DDN/ZBTB7A/TFAP2B/SOX12/NFIC/BACH1/NACC2/BARHL1/ONECUT2/MYC/SP1/TGIF1/CC2D1B/SREBF1/FOSL2/ZKSCAN3/ZNF148/OVOL1/FOXO1/ZBTB7B/SP2 | 34 |
| GO:0000987 | proximal promoter sequence-specific DNA binding | 36/693 | 451/17548 | 5.23E-05 | 0.014360726 | 0.01337163 | TP53/UBTF/NFAT5/HNRNPC/AGO1/HAND2/ZFHX3/NEUROD2/SREBF2/TBR1/GCFC2/POU2F3/TCF7L2/PAX2/HMGA2/USF2/SSBP2/ATF6/DDN/ZBTB7A/NFIC/NACC2/NSD1/ONECUT2/MYC/SP1/TGIF1/RBBP4/CC2D1B/SREBF1/FOSL2/ZKSCAN3/ZNF148/OVOL1/ZBTB7B/SP2 | 36 |
| GO:0000978 | RNA polymerase II proximal promoter sequence-specific DNA binding | 34/693 | 436/17548 | 0.000131799 | 0.02711773 | 0.025249994 | TP53/NFAT5/HNRNPC/AGO1/HAND2/ZFHX3/NEUROD2/SREBF2/TBR1/GCFC2/POU2F3/TCF7L2/HMGA2/USF2/SSBP2/ATF6/DDN/ZBTB7A/NFIC/NACC2/NSD1/ONECUT2/MYC/SP1/TGIF1/RBBP4/CC2D1B/SREBF1/FOSL2/ZKSCAN3/ZNF148/OVOL1/ZBTB7B/SP2 | 34 |
| GO:0001228 | transcriptional activator activity, RNA polymerase II transcription regulatory region sequence-specific DNA binding | 32/693 | 417/17548 | 0.000274502 | 0.044657302 | 0.041581525 | PLAGL2/TP53/NFAT5/RXRB/HOXC4/MLXIP/HAND2/NEUROD2/HMGA1/POU2F3/HMGA2/USF2/SSBP2/IKZF3/ATF6/TFAP2B/SOX12/NFIC/CSRNP3/BACH1/SALL2/BARHL1/FOXC1/ONECUT2/MYC/SP1/HCFC1/SIX3/SREBF1/SPIB/FOSL2/ZBTB7B | 32 |
| GO:0003680 | AT DNA binding | 4/693 | 10/17548 | 0.000418524 | 0.044657302 | 0.041581525 | HAND2/HMGA1/HMGA2/KMT2A | 4 |
| GO:0001078 | transcriptional repressor activity, RNA polymerase II proximal promoter sequence-specific DNA binding | 15/693 | 141/17548 | 0.000463349 | 0.044657302 | 0.041581525 | BTG2/ZFHX3/SREBF2/GCFC2/HMGA2/ZBTB7A/BACH1/NACC2/TGIF1/CC2D1B/ZKSCAN3/ZNF148/OVOL1/FOXO1/SP2 | 15 |
| GO:0001047 | core promoter binding | 15/693 | 142/17548 | 0.000499631 | 0.044657302 | 0.041581525 | TP53/UBTF/AGO1/ZFHX3/SFPQ/PRDM10/HMGA2/MAZ/TFAP2B/HDAC5/DHX36/MTA1/SP1/KMT2A/KDM5A | 15 |
| GO:0061631 | ubiquitin conjugating enzyme activity | 6/693 | 27/17548 | 0.000539652 | 0.044657302 | 0.041581525 | UBE2S/UBE2G1/UBE2Z/UBE2D3/UBE2D4/UBE2D2 | 6 |
| GO:0051015 | actin filament binding | 17/693 | 174/17548 | 0.000542616 | 0.044657302 | 0.041581525 | TAGLN/CAPZB/WIPF2/BLOC1S6/MYO1C/CACNB2/ADD2/PKNOX2/HIP1/MYH9/ACTR2/MYH14/CFL2/ABL1/TLN2/SAMD14/TULP1 | 17 |
| GO:0061650 | ubiquitin-like protein conjugating enzyme activity | 6/693 | 28/17548 | 0.000664124 | 0.049688573 | 0.046266267 | UBE2S/UBE2G1/UBE2Z/UBE2D3/UBE2D4/UBE2D2 | 6 |
