# Supplementary figures and images for: Up-Regulation of hsa_circ_0000517 Predicts Adverse Prognosis of Hepatocellular Carcinoma
Source: Front Oncol. 2019 Oct 22;9:1105. doi: 10.3389/fonc.2019.01105 (PMC6842961; doi:10.3389/fonc.2019.01105)

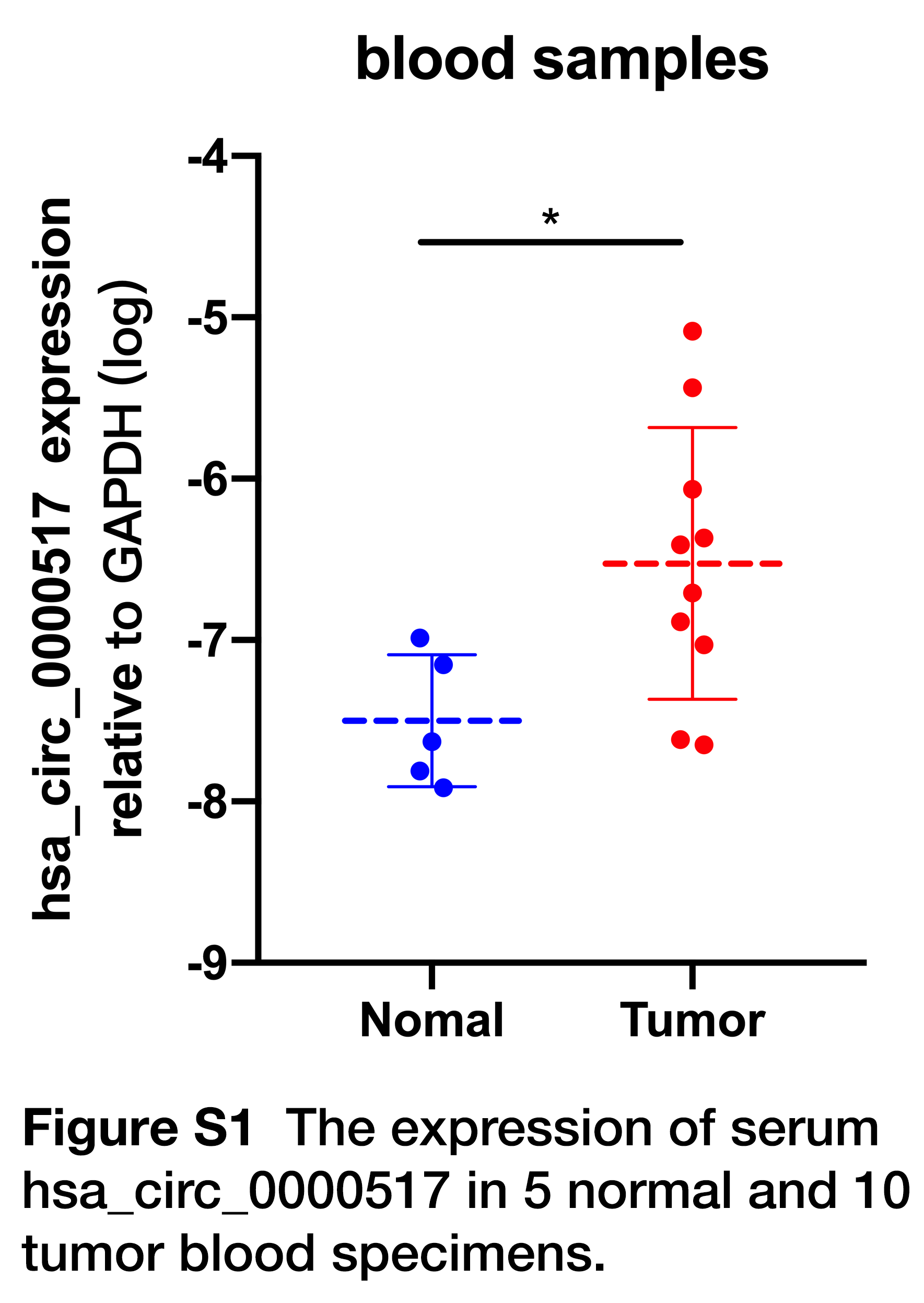

Supplement: Supplementary file 8 [file Image_1.TIFF]
